# Continuous Radar Tracking Illustrates the Development of Multi-destination Routes of Bumblebees

**DOI:** 10.1038/s41598-017-17553-1

**Published:** 2017-12-11

**Authors:** Joseph L. Woodgate, James C. Makinson, Ka S. Lim, Andrew M. Reynolds, Lars Chittka

**Affiliations:** 1 Department of Biological and Experimental Psychology, School of Biological and Chemical Sciences, Queen Mary University of London, Mile End Road, London, E1 4NS UK; 2 Rothamsted Research, West Common, Harpenden, Hertfordshire, AL5 2JQ UK; 3 Wissenschaftskolleg zu Berlin Institute for Advanced Study, Wallotstrasse 19, Berlin, D-14193 Germany

## Abstract

Animals that visit multiple foraging sites face a problem, analogous to the Travelling Salesman Problem, of finding an efficient route. We explored bumblebees’ route development on an array of five artificial flowers in which minimising travel distances between individual feeders conflicted with minimising overall distance. No previous study of bee spatial navigation has been able to follow animals’ movement during learning; we tracked bumblebee foragers continuously, using harmonic radar, and examined the process of route formation in detail for a small number of selected individuals. On our array, bees did not settle on visit sequences that gave the shortest overall path, but prioritised movements to nearby feeders. Nonetheless, flight distance and duration reduced with experience. This increased efficiency was attributable mainly to experienced bees reducing exploration beyond the feeder array and flights becoming straighter with experience, rather than improvements in the sequence of feeder visits. Flight paths of all legs of a flight stabilised at similar rates, whereas the first few feeder visits became fixed early while bees continued to experiment with the order of later visits. Stabilising early sections of a route and prioritising travel between nearby destinations may reduce the search space, allowing rapid adoption of efficient routes.

## Introduction

Central place foragers, like bees, that visit multiple feeding destinations on a single foraging trip, often show evidence of ‘trapline’ routes, returning to the same locations in a repeatable order, like hunters checking a series of traps^1–8^. Traplining ensures animals do not need to search afresh on each trip^9,10^ and reduces the variance in the frequency of returns to each patch^11,12^. Traplines might also be advantageous by increasing the probability of finding resources like slowly accumulating nectar before competitors, preventing build-ups of resources that might attract more competitors; and ensuring optimal rates of visitation to each patch^13,14^.

The development of trapline routes in bees has been examined by recording the sequence of visits to flowers to infer the rules underlying route formation^3,9–13,15–19^. Examining route formation at the level of visit sequences means prior studies have been unable to examine in detail the flight paths by which animals move around the feeder array. Lihoreau *et al*.^20^ recorded the flight paths of experienced bees but could not study how bees first found the feeders, how or whether they explored in search of alternative food sources, how their movements changed as they refined their visit sequence or whether movements between flowers improved and stabilised in similar ways to the sequence of visits to the flowers themselves. In this study, we used harmonic radar to record the flight of bumblebee workers continuously, for up to two days, as they foraged on an artificial feeder array. We were thus able to examine in detail their movements between feeders in parallel with analysis of the visit sequences, throughout the entire period of route learning. We developed new methods to compare flight paths, by estimating the probability that the bee passed over the same parts of the landscape in different flights, using methods derived from Brownian Bridge Movement Models^21^, allowing us to use this unprecedentedly detailed spatial data to understand how bees’ movements develop and stabilise with experience.

Current technology only allows us to track one individual at a time and technical challenges mean that continuous tracking is more difficult than tracking individuals for a single flight each, necessitating a trade-off between the number of individuals tracked and the number of flights recorded from each. We have chosen to record the full histories of 6 bees rather than fragments of behaviour from a larger number of individuals. Although unconventional, this technique gives unprecedented insight into the route formation process for these bees, although caution must be exercised in extrapolating from these bees to the larger population.

The problem faced by traplining bees is analogous to the Travelling Salesman Problem (TSP), an extensively studied mathematical problem^22^, for which there is no solution short of testing every possible route. Bees seldom develop completely stereotyped routes, but show significantly non-random patterns of visitation and often follow preferred sequences of feeder visits^11–13,23^. In developing efficient routes, bees might minimise either the distance travelled between pairs of locations^3,10,12^ or total distance travelled on each journey^15–17,20^; these are often correlated, and can be difficult to disentangle. Additionally, there is evidence for a trade-off between finding the shortest route and prioritising visits to more rewarding food sources^15^, and between accuracy of route following and travel speed^18,19^. Ohashi *et al*.^12^ found bees preferred to minimise feeder-to-feeder distances over forming a straight path in an indoor array with feeders spaced ~1 m apart, preventing them from taking the optimal path which would minimise the overall travel distance. It has been suggested that bees may be more motivated to optimise routes at large spatial scales^15,20^. Lihoreau *et al*. developed a traplining heuristic model using a process of iterative improvement based on the overall length of each route tried^20,24^, which reproduced a number of results seen in the behaviour of real bees^24^, but predicted there are certain spatial configurations for which bees cannot form stable traplines that minimise travel distance, including the conflicting array used by Ohashi *et al*.^12^.

**Figure 1:**
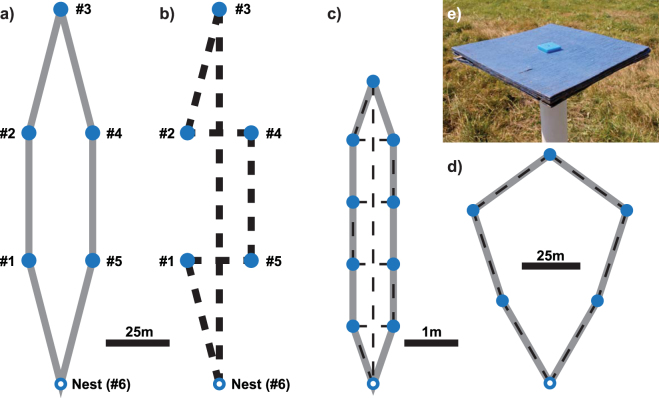
Set-up of feeder array. (**a**) Diagram of feeder array showing optimal route (the path that visits all 5 feeders and returns to the nest in the shortest distance; solid grey line). The feeders (filled blue circles) are referred to by the numbers #1-#5 throughout the text, and the nest location (empty blue circle) by the number #6. (**b**) Diagram of feeder array showing nearest neighbour route (the path described by an algorithm which, at each step, visits the nearest feeder that has not yet been visited; dashed black line). (**c**) Diagram of negative feeder array used by Ohashi *et al*.^12^, showing optimal (solid grey line) and nearest-neighbour routes (dashed black line). In common with the array used in our experiment, there is a conflict between the shortest overall route and the shortest feeder-to-feeder distances. (**d**) Diagram of feeder array used by Lihoreau *et al*.^20^. Note that optimal and nearest-neighbour routes are identical. (**e**) Photograph of a feeder platform as used in our array, showing acrylic chip with well for sucrose solution.

In the only study of trapline foraging at a large spatial scale to date, Lihoreau *et al*.^20^ found that bees developed a repeatable trapline on a pentagonal feeder array, following the route which required the shortest possible flight path; however this was also the route that would result from use of a nearest-neighbour heuristic, where an agent always travels to the closest as-yet-unvisited location (see Fig. 1). In this study, we continuously tracked bees foraging on a feeder array at a large spatial scale to investigate the process of route development when minimising the distance between individual pairs of feeders would conflict with minimising the overall travel distance. Our array, like that of Ohashi *et al*.^12^ had the property that following a nearest-neighbour rule between individual feeders would lead to a larger than optimal travel distance (see Fig. 1). In common with Lihoreau *et al*.^20^, our array involved five feeders and the length of the shortest possible path was identical. Moreover, in both arrays, to minimise overall route length, a bee should take a path around the edge of the array (Fig. 1). Because the distances and number of feeders were the same as those used by^20^, any differences in performance must be attributable to the spatial configuration of the feeders.

## Results

### Flight structure and development

Tracking bees over multiple consecutive flights presents formidable challenges and no previous study has tracked bees as they learn any spatial navigation task. Despite these difficulties we managed to track six bees continuously through every foraging bout they made on the experimental feeder array for up to 61 foraging bouts each (summarised in supplementary Table S1; the radar tracks for every bout can be seen in Supplementary Figs S1-S201 and supplementary videos S1-S6). The two feeders closest to the nest (#1 and #5, see Fig. 1) were discovered on the first foraging bout by all bees. We estimated that feeders should not be visible to bees from further than ~18 m (although we did not test this empirically; see Material & Methods), so feeders #1 and #5 should not be visible from the nest (distance = 50 m) although they would be visible from the location the bees were familiar with from training (halfway between feeders #1 and #5, which were 25 m from each other). Two bees flew directly to this middle point before turning at right angles to fly toward a feeder (see supplementary Figs S48, S196), but others seemed to fly to their first-visited feeder in a fairly straight line (supplementary Figs S1, S187). Possibly these bees noticed the feeders earlier, while heading toward the middle point, and adjusted their course straight away. Bees 1, 2, 4 and 5 also discovered the next closest pair of feeders (#2 and #4) on their first bouts, while bee 3 discovered them during its second bout. (Bee 6 discovered feeder #2 on its second bout but never visited #4). No bees discovered the furthest feeder (#3) on their first bout, but it was located by bees 1–3 and 5 within 4–6 bouts. Bees 4 and 6 never visited feeder #3. The first bout in which bees 1–3 and 5 visited all five feeders also occurred within 4–6 bouts, but bees 4 and 6 never visited every feeder.

Figure 2 (and Supplementary Fig. S202) shows the cumulative probabilities that each bee visited different parts of the landscape over their first five foraging bouts, the last five bouts of the first day of foraging and the last five bouts of the second day of foraging (where applicable). The figures show how bees’ routes develop and stabilise with experience. Route development is demonstrated graphically in supplementary videos S1-S3. In the first five foraging bouts (Fig. 2a–d), bees’ movements within the feeder array were quite diffuse, indicating that they were not following stereotyped routes. However, the radar tracks show, perhaps surprisingly, that the majority of feeder-to-feeder flights were relatively straight, even in the earliest bouts, suggesting that feeder locations were quickly memorised and that there was little searching for feeders within the array (see supplementary Figs S1–S201).

**Figure 2:**
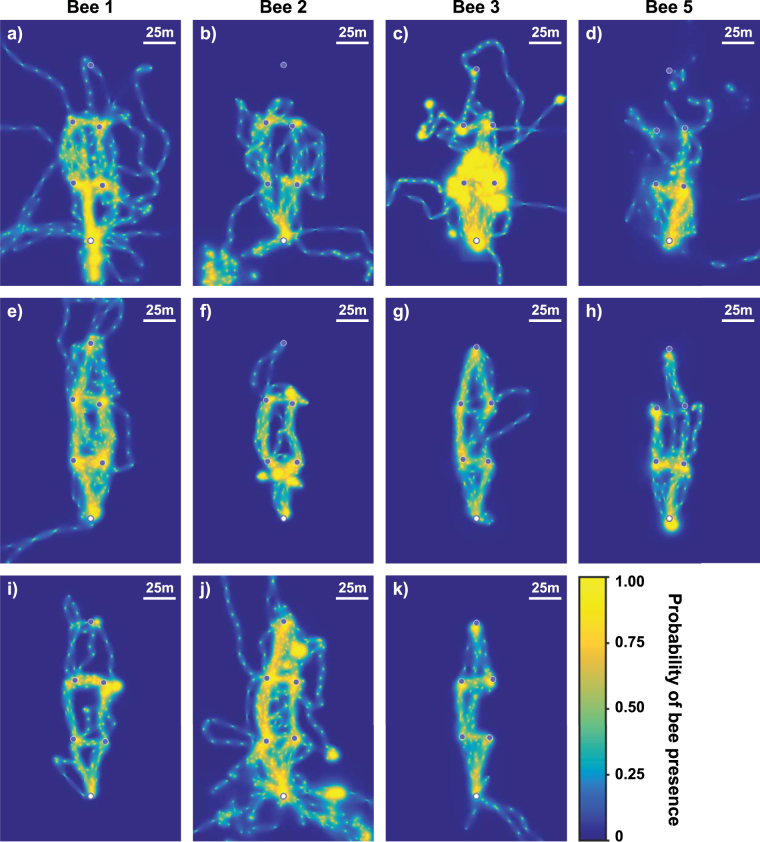
Heat maps showing the development and stabilisation of routes between feeders. The nest position is represented by an open circle and the positions of the feeders by closed circles. The colour of each pixel represents the probability that the bee passed over that point in the landscape as estimated from the radar tracks using a Brownian bridges technique (described in Methods). Each pixel represents an area 5 × 5 m. Scale bars represent a distance of 25 m. Each panel represents the cumulative probability map of one bee’s location over 5 consecutive bouts. The first column shows the activity of bee 1 at three levels of foraging experience; the other columns show bees 2, 3 and 5 respectively. Bees 4 and 6 did not complete enough foraging bouts to investigate how their routes developed with experience; their initial foraging bouts are shown in Supplementary Fig. S202. (**a**–**d**) Cumulative activity of bees 1–3 and 5 respectively, over the course of their first 5 foraging bouts on the experimental array. All bees travelled widely over the area bounded by the nest and four closest feeders, and made many exploratory flights beyond the area of the array; (**e**–**h**) Cumulative activity of bees 1–3 and 5 respectively, over their last 5 foraging bouts on the first day of tracking. The bees’ activity within the area of the array was now largely restricted to narrow corridors between feeders and flights beyond the array were much reduced. The furthest feeder was visited more regularly than during the initial bouts. (**i**–**k**) Cumulative activity of bees 1–3 respectively, over their last 5 foraging bouts on the second day of tracking; bee 5 was only recorded for one day. Routes within the array were further refined. Bee 2 showed a resurgence of flights beyond the area of the array.

Revisits to empty feeders were common throughout the experiment (mean revisits per bout across all bees, 4.46 ± 4.75; means are reported ± s.d. throughout the text). We attempted to deduce whether bees landed on the feeders during these visits or merely overflew them, by looking for occasions when the bee moved less than 2.5 m between consecutive radar datapoints. The mean proportion of first visits to a full feeder within each bout in which the bee met this criterion of stillness was 0.92 ± 0.17. That of revisits was only 0.58 ± 0.35, but this is likely to be an underestimate since this method only detects stops longer than 4.5 s on average (due to the rotation of the radar) and we have previously observed bees landing, sampling an empty feeder and taking to the air again in only 1–2 s.

Early flights also show numerous loops far beyond the feeder array. In the case of bee 2, several flights beyond the array ended at a known patch of wild flowers and the radar data show the bee remaining fairly still for a period of time, suggesting that it had stopped to feed (see supplementary Figs S48, S54). There was nothing to suggest the other bees stopped to feed during these explorations beyond the array, but they may have been searching for more feeders or alternative food sources. Flight paths were subdivided into *legs*, sections of flight between one feeder (or nest) and another. We characterised any leg in which the bees flew mores than 50 m from all previously discovered feeders as exploration legs. These were observed to originate at all locations but most commonly started from the nest (supplementary Fig. S203a). Most occurred not upon first leaving the nest but after consuming the sucrose solution at several (but rarely all five) feeders (supplementary Fig. S203b) and returning to within 5 m of the nest without entering it. Exploration legs nearly always happened before any revisits to empty feeders (supplementary Fig. S203c). Exploration flights were observed to head in all directions suggesting the bees were not drawn to particular parts of the landscape, but the radar tracks do suggest that individual bees may have had idiosyncratic preferred directions, making similar explorations on multiple feeding bouts (e.g. bee 3: supplementary Figs S104, S105, S111, S116; bee 4: S162-S165).

By the time of the last bouts made on the first day of foraging (Fig. 2e–h) the movement patterns show considerable changes: flights beyond the array almost ceased and movement within the array had become largely confined to more direct, narrow corridors. Some pairs of feeders were very strongly connected, suggesting stable, stereotyped segments of the route, while others had weaker connections, indicating less common transitions. For bees 1–3 we also examine the last five bouts on their second day of foraging (Fig. 2i–l). Their movements were again largely confined to straight connecting flights between feeder locations, but the shape of the routes was different, demonstrating that their routes continued to develop. Bee 2 (Fig. 2j) showed a resurgence of exploratory flight outside the feeder array but its movements within the array were still concentrated on a few straight transitions between feeders, so exploration flights were added to habitual routes. These late explorations took the bee to parts of the landscape it visited during its first bout on the array, but did not closely resemble its other early exploration legs. In particular the bee did not revisit the flower patch it likely foraged at during its first day on the experimental array.

### Distance and duration of flight

**Figure 3:**
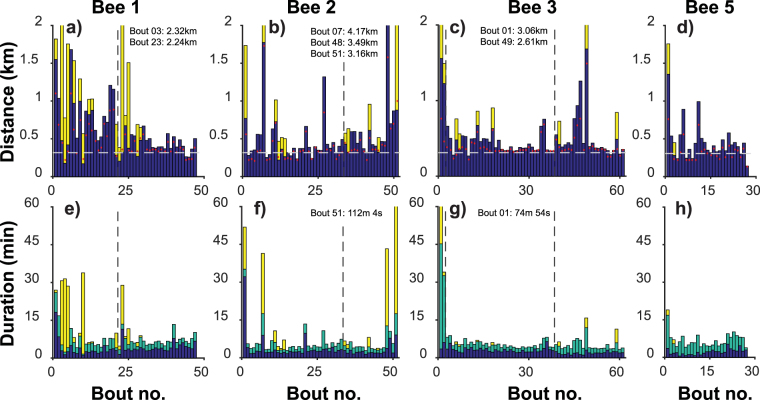
Path length and duration of foraging bouts within and outside array. X-axis of each plot shows the cumulative number of foraging bouts experienced by one bee. Vertical dashed lines indicate overnight breaks. (**a**–**d**) Path length of each bout by bees 1–3 and 5 respectively. Path lengths are the cumulative length of a series of straight lines joining every radar observation of a bee’s position. Total distance flown is broken down into two categories: blue bars show distance flown in legs that remained within 50 m of a feeder; yellow bars show distance flown during ‘exploration’ legs (in which the bee travels further than 50 m from all previously visited feeders). The horizontal dashed grey line indicates the minimum possible distance of an optimal route that visits all feeders. Note that the actual distance flown can be less than this theoretical minimum in cases where the bee did not visit all 5 feeders. Red dots indicate straight-line path length, the theoretical distance of a route composed of straight lines that visits the feeders in the order observed in each bout. Exploration legs were largely confined to the first few bouts each day. The total distance flown in each bout showed a decreasing trend with experience. The y-axis is clipped at 2 km for clarity. The total distance travelled on bouts that exceeded 2 km is given in text on the figure. (**e**–**h**) Duration of each bout. Total duration is broken down into three categories: blue bars show the time spent within 5 m of a feeder; green bars show the duration of legs that remained within 50 m of a feeder; yellow bars show the total duration of ‘exploration’ legs. The y-axis is clipped at 60 minutes for clarity. The total duration of bouts that exceeded 60 minutes is given in text on the figure. Time spent on feeders was high during the first few bouts by each bee and may represent time spent learning the feeder locations; this time fell in subsequent bouts and remained largely unchanged, likely reflecting the time required to ingest food.

**Figure 4:**
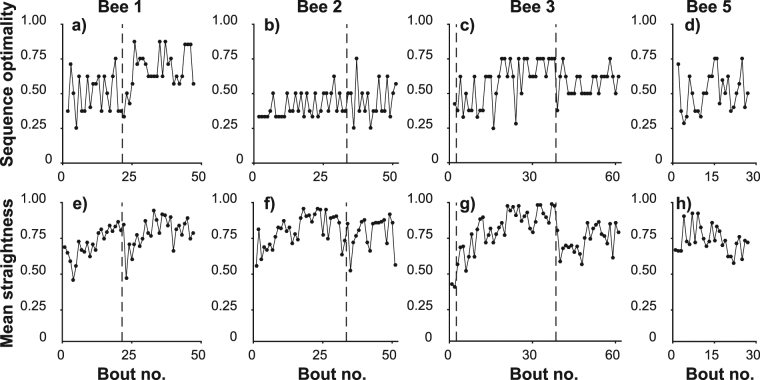
Feeder visit sequence and flight path become more efficient with experience. Each panel represents every foraging bout flown by one bee. The x-axis of each plot shows the cumulative number of foraging bouts experienced by each bee. The vertical dashed lines indicate overnight breaks between foraging bouts. (**a–d**) Optimality score for the feeder visit sequence in each bout by bees 1–3 and 5 respectively (similarity of the feeder visit sequence in each bout to the theoretically optimal visit sequence). Visit sequences became more similar to the optimal sequence as bees gained experience. (**e–h**) Mean straightness of the flight paths of all legs in each bout by bees 1–3 and 5 respectively. Flights between feeders tended to become straighter with experience.

Both flight path length and duration decreased significantly from the beginning to the end of the first day’s foraging (Table 1, Fig. 3, supplementary Fig. S204), suggesting a tendency for routes to become more efficient with experience. Even after a day’s foraging, however, the mean path length was 538.9 m ± 280.1, far in excess of the minimum necessary travel distance (300 m; Table 1). Path length can be subdivided into legs within the feeder array or exploration legs. Exploration legs accounted for very large travel distances (Fig. 3a–d) and very high proportions of the total path length (supplementary Fig. 205) and largely occurred during the first few foraging bouts of each day (the proportion of total path length attributable to exploration legs was 0.21 ± 0.30 over the first five foraging bouts but just 0.03 ± 0.13 over the last five bouts). Reduction in exploration legs accounts for the majority of the reduction in path length over the course of a day (proportion of total distance reduction between first and last five bouts during the first day attributable to exploration legs: 0.69 ± 0.37). The mean straightness of the flight paths between feeders tended to increase with experience (Table 1, Fig. 4e–h), also contributing to reduction in path length.

**Table 1:** Descriptive statistics and results of ANOVAs comparing first five foraging bouts with the last five performed on the first day of foraging.

Dependent variable	Mean ± s.d. First bouts	Mean ± s.d. Last bouts	N (Bees)	d.f.	*F*	*P*
Path length, total	1016.3 ± 785.4	538.9 ± 280.1	4	1,35	7.12	**0.0115**
Straight-line path length	418.4 ± 240.2	428.6 ± 211.7	4	1,35	0.10	0.7515
Straight-line/observed path length	0.53 ± 0.21	0.82 ± 0.14	4	1,35	31.2	**<0.001**
Duration, total	1140.7 ± 1132.6	402.6 ± 120.4	4	1,35	9.93	**0.0033**
Visit sequence optimality	0.44 ± 0.15	0.55 ± 0.16	4	1,35	7.02	**0.0120**
Straightness	0.63 ± 0.12	0.82 ± 0.11	4	1,35	23.9	**<0.001**
Visit sequence self-similarity	0.65 ± 0.16	0.69 ± 0.15	4	1,35	0.923	**0.3434**
Flight path self-similarity	0.46 ± 0.14	0.56 ± 0.17	4	1,35	4.97	**0.0324**
Mean flight path leg similarity	0.43 ± 0.10	0.52 ± 0.08	4	1,35	10.5	**0.0027**

Bout duration was subdivided into time spent within 5 m of feeder locations, flight time within the array and flight time during exploration legs. Time spent near feeders (which will include feeding and food-handling time as well any time spent learning the appearance or location of the feeders) stayed largely constant (first five bouts, 319.8 s ± 443.9; last five bouts, 200.8 s ± 62.4). Reduction in time spent on exploration legs explains around half the observed reduction in total flight duration (0.53 ± 0.22).

### Visitation sequences and route optimization

All except bees 4 and 6 met the established definition of route stabilisation (identical visitation sequence, excluding revisits to emptied feeders, over three consecutive bouts^20^) after 17–34 bouts (supplementary Table S1), comparable to the 30 bouts reported by Lihoreau *et al*.^20^, but all went on to make further bouts with differing sequences. If revisits to empty feeders were included, no bee would ever have met the criterion of three (or even two) identical consecutive bouts. This means that the true routes taken by the bees never became fully stereotyped.

Optimality scores measured how similar the visitation sequence was to the optimal sequence (minimal overall travel distance), and increased significantly with time and experience (Table 1, Fig. 4a-d), although only one bee ever used the shortest route (bee 1, on 15 of 47 foraging bouts; see supplementary Figs S13, S15, S18, S19, S25, S26, S29, S31, S32, S34, S35, S36, S37, S41, S43). By contrast, all bees tested in a regular pentagon used the shortest route^20^. Despite the visitation sequences becoming closer to the optimal sequence, this did not lead to visit sequences with a shorter straight-line path length (Euclidean distance between feeders in the order visited; Table 1). However, by flying straighter between feeders, bees were able to bring the distance they actually flew closer to the straight-line path length, after a day’s foraging (Table 1, supplementary Fig. S205). By the last five bouts of day one, the mean proportion of the total distance flown which was attributable to the straight-line path length was 0.82 ± 0.14 showing that feeder visitation sequence became the main determinant of total distance flown.

Given that improvement in visitation sequence was not an important contributor to increasing route efficiency, what rules were involved in determining sequence choice? One easily computed rule is the nearest-neighbour heuristic in which a bee should always travel to the nearest unvisited feeder (Fig. 1b). Two bees made multiple trips whose visit sequences only involved nearest-neighbour transitions (bees 3 and 4; see supplementary Figs S141, S142, S143, S146, S148, S149, S150, S151, S152, S153, S157, S159 and S171, S180, S181, S183, S184, S185, S186, S187, S189, S190). Although the sequences of the other bees did not use nearest-neighbour transitions exclusively, across all bees, the mean proportion of legs in each bout in which the bee flew to the closest location was 0.56 ± 0.12. The mean distance rank (which quantified whether the legs within a bout involved moving to close or distant locations) across all bees was 1.45 ± 0.17. In other words, most transitions were to the closest or second closest feeder from the starting point, mixed in roughly equal proportions. There was no obvious change in distance rank over time (supplementary Fig. S207). Our feeder array was designed so that following a nearest-neighbour rule would lead to unnecessarily long path lengths, which explains why changes in the visitation sequence in this experiment failed to reduce the straight-line path length. In many natural situations, a preference for nearby locations would result in a close-to-minimal path length.

### Route repeatability

**Figure 5:**
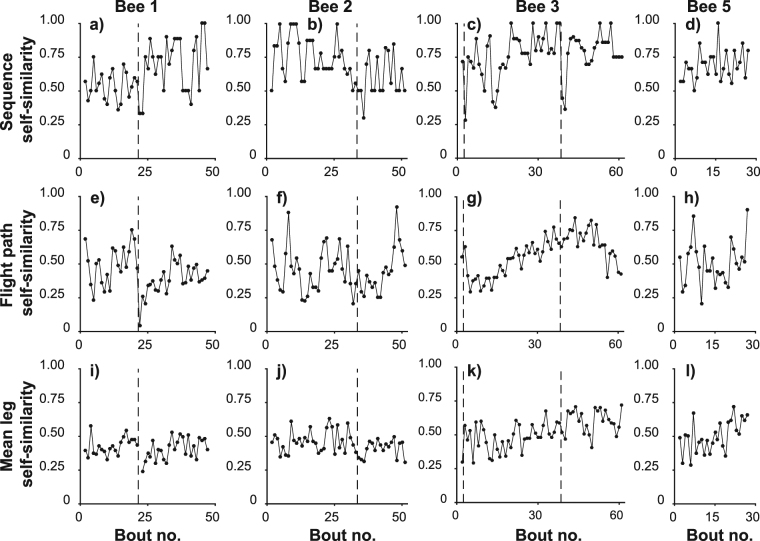
Feeder visit sequence and flight path become more repeatable with experience. Each panel represents every foraging bout flown by one bee. The x-axis of each plot shows the cumulative number of foraging bouts experienced by each bee. The vertical dashed lines indicate overnight breaks between foraging bouts. (**a**–**d**) Self-similarity score for the feeder visit sequence in each bout by bees 1–3 and 5 respectively (similarity of the sequence of feeder visits in each bout to that of the previous bout undertaken by the same bee). Visit sequences increased in similarity with experience. (**e**–**h**) Self-similarity score comparing the probability map of the bee’s location during each bout to that of the previous bout by bees 1–3 and 5 respectively. Flight paths became more similar to one another with experience. (**i**–**l**) For bees 1–3 and 5 respectively, the mean similarity of the individual legs of each bout to the most recent occasion on which the same bee made the same feeder-to-feeder transition. Even after thus accounting for the increasing likelihood of legs to share the same origin and destination, the probability maps describing the flight path showed a trend toward increasing self-similarity over time.

A similarity score which compared each bout’s visitation sequence to that of the previous bout showed no significant change over day 1 (Table 1, Fig. 5a–d). We also developed a similarity score to compare actual flight paths. We multiply two probability maps to compute the probability of bees having visited the same location in each bout and compute a score to express the total similarity as a proportion of the similarity of one path to itself. These scores showed that flight paths became more similar with experience (Table 1, Fig. 5e–h). Theoretically, flight paths could evolve by small incremental changes such that each path would be similar to its predecessor and yet large changes could accumulate over time. To investigate this possibility we also compared each bout’s flight path to that occurring 2, 3 or 4 bouts previously. Supplementary Fig. S209 shows that the same trend toward increasing similarity occurs across longer scales than just consecutive bouts, confirming that the rate of change in flight paths decreased as bees’ gained experience.

An increase in similarity across an entire bout could reflect either an increasing tendency to fly between locations along a similar path or an increasing number of transitions between the same start and end points, meaning that the bee would be more likely to be in the same general areas of the landscape. To control for this we calculated the similarity of each individual leg within a bout to the most recent occasion on which that bee had flown from the same start point to the same end point. The mean self-similarity of all the legs in each bout also increased with experience (Table 1, Fig. 5i–l), demonstrating that flight paths stabilised independently of the feeder visitation sequence.

**Figure 6:**
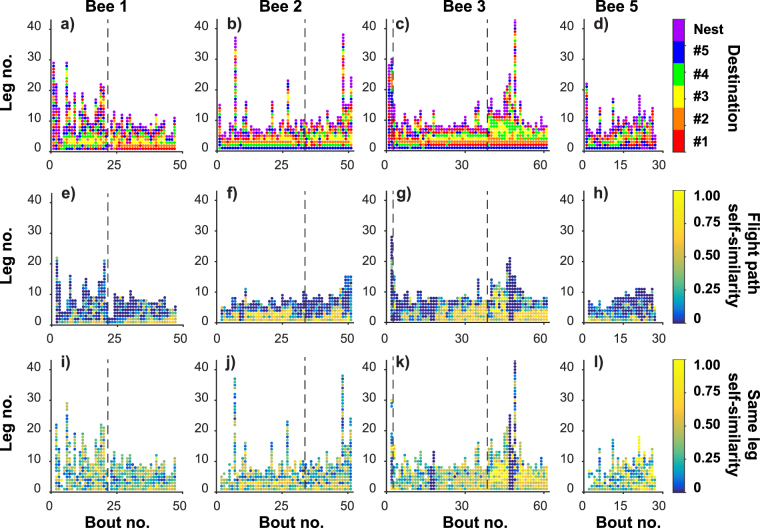
Individual legs of a foraging route stabilise at different rates. Each panel represents every individual leg (transitions from any feeder to another) of every foraging bout flown by one bee. The x-axis shows the cumulative number of foraging bouts. The y-axis shows the cumulative number of legs within each bout. Vertical dashed lines indicate overnight breaks between foraging bouts. (**a**–**d**) For bees 1–3 and 5 respectively, the colour of each dot represents the identity of the destination of each leg of the route (feeders #1-#5 or the nest). Horizontal rows of the same colour demonstrate that a bee visited the same destination at the same stage of several consecutive bouts. Such patterns appeared early during the second day of foraging by bee 1 and during the first days of foraging by the other bees. These stable elements of the feeder visit sequence were more common in the earlier legs of each bout (lower rows on the y-axis). (**e**–**h**) For bees 1–3 and 5 respectively, the colour of each dot represents the self-similarity score comparing the probability map of the bee’s location during each leg of the bout to that of the same leg in the previous bout. (**i**–**l**) For bees 1–3 and 5 respectively, the colour of each dot represents the similarity of each leg within each bout to the most recent occasion on which the same bee made the same feeder-to-feeder transition.

The traplining heuristic model^24^ suggested that foraging routes do not change or improve all at once, but as a consequence of altering the probabilities of individual feeder-to-feeder transitions (legs). Figure 6 visualises this process and investigates whether some legs of the route stabilize more quickly than others. Figure 6a–d show the destination of each leg of each bout. Horizontal rows of the same colour indicate that the same feeder was visited at the same stage of several consecutive bouts and illustrate repeatable visit sequences. Although our sample size is too low to permit detailed statistical analysis, we believe that two general trends can be discerned: individual legs are more likely to stabilise on the same destination as the bees gain experience, and early legs within each bout are more likely to have stable destinations than later ones.

To examine the plasticity of segments of foraging routes, we broke down each bout into segments of two, three or four consecutive feeder visits. Supplementary Fig. S211 plots the probability of each segment of flight occurring within a foraging bout, on a log-linear scale, against their rank order of likeliness. The majority of segments fit an exponential distribution, which is a hallmark of random sequences^25^ and suggests that these parts of a visitation sequence were generated in a purely stochastic way. However, the most commonly occurring segments, toward the left of each plot, deviate greatly from an exponential distribution. This indicates that approximately the 20 most common sections of flight were not randomly chosen and presumably resulted from the bees’ memory and decision making, while a random element in the choice of rarer segments allowed them to experiment with new sequences.

Figure 6e–h show how similar the flight path of each leg was to that of the leg occurring at the same stage of the previous bout. Again, two trends are suggested: leg similarity increased with experience within each day of tracking (there was a sharp drop off between days for bees 1 and 2); and leg similarity may decrease with increasing leg number, possibly indicating that, within each bout, early legs stabilised faster than later legs. We also calculated the similarity of each leg to the most recent occasion on which the bee flew from the same starting point to the same end point (Fig. 6i–l), but there was no evident pattern of earlier legs having higher scores than later legs in the same bout. Individual legs sharing the same start and end position become more similar with experience largely because they converge on straight-line routes, and our data suggest that this occurs at the same rate for all destinations. If the early legs in a bout stabilise faster, then, this is largely the result of the destinations of early legs becoming fixed while later legs did not.

### Effects of overnight breaks

Three bees were recorded foraging over two full days. Bees 2 and 3 were confined to the nest overnight between days. Bee 1 was confined to the nest for four days and five nights between the first and second day of tracking (bee 2 was tracked during the intervening period). To investigate whether this break between bouts affected foraging routes we tested for a difference between the final five bouts of the first day and the initial five bouts of the second day’s foraging in all variables that had showed a significant effect of experience over the first day, plus the distance flown during exploration legs (Table 2). While there was no significant change in the total flight path length or duration, there was a significant increase in the distance flown in exploration bouts between days (Table 2, Fig. 3). There was also a significant decrease in the proportion of the total path length that could be accounted for by the visitation sequence (Table 2, supplementary Fig. S205), mean straightness of legs within a bout (Table 2, Fig. 4) and flight path self-similarity (Table 2, Fig. 5). Periods without active foraging, even overnight breaks, led to less straight and repeatable flights. It is not clear whether this resulted from degradation of route or location memories, or from a resurgence of exploration behaviour.

**Table 2:** Descriptive statistics and results of ANOVAs comparing last five foraging bouts performed on the first day of foraging with the first five on the second day.

Dependent variable	Mean ± s.d. End day 1	Mean ± s.d. Start day 2	N (Bees)	d.f.	*F*	*P*
Path length, total	603.1 ± 289.8	715.4 ± 474.3	3	1,26	1.04	0.3174
Path length, exploration legs	27.3 ± 105.8	266.8 ± 512.2	3	1,26	7.97	**0.0090**
Straight-line/observed path length	0.81 ± 0.16	0.65 ± 0.22	3	1,26	8.70	**0.0067**
Duration, total	382.2 ± 110.5	444.2 ± 378.0	3	1,26	0.0148	0.9040
Visit sequence optimality	0.56 ± 0.17	0.53 ± 0.17	3	1,26	0.211	0.6497
Straightness	0.86 ± 0.09	0.70 ± 0.11	3	1,26	18.8	**0.0002**
Flight path self-similarity	0.55 ± 0.17	0.43 ± 0.22	3	1,26	5.37	**0.0286**
Mean flight path leg similarity	0.49 ± 0.07	0.44 ± 0.15	3	1,24	2.50	**0.1267**

## Discussion

Previous studies into multi-destination route formation have focussed on sequences of feeder visits but researchers have not been able to track the ontogeny of actual flight paths (routes taken between feeding stations) of their subjects^3,9–12,15–20^. We used harmonic radar to track the flight paths of bees continuously for the entire duration of testing and examined the development of flights, as well as visit sequences, for the first time. We tested whether bumblebee foragers were able to find the shortest route on a large-scale feeder array, designed so that consistently minimising the travel distance between individual pairs of feeders would lead to suboptimal overall route lengths^12^. None of our bees regularly managed to choose a route that minimised overall travel distance, instead showing behaviour consistent with a preference for shorter inter-feeder travel distances. Analysis of the actual flight paths taken by our subject bees suggested that the flight paths between feeders became both straighter and more repeatable with experience and that this process was independent of changes to the feeder visit sequence. Travel distance and duration reduced with experience and these efficiency gains had two main causes: a reduction in the distance and time spent flying outside the area of the feeder array, and an increasing tendency to take straighter routes between feeders. Changes in visit sequence did not lead to a significant reduction in travel distance, although it is likely that in other spatial arrangements of feeders, similar sequence refinement would reduce distances.

Inexperienced bees made frequent looping flights beyond the feeder array, but reduced the incidence of these exploration flights as they gained experience, reducing the distance and duration of foraging bouts (although exploration legs were seen again on the second morning of tracking). In a previous study^26^ we tracked free-foraging bumblebees throughout their lives and identified a class of long, circuitous exploration flights which largely occurred during the first few flights ever made by bumblebee workers. The exploration legs we identified in this study bear some resemblance to those of new foragers, being long, highly circuitous and apparently using the nest as a central point from which to explore. It is not clear what caused bees to make exploration legs in this study. The subject bees had previous experience of the field site, accrued during the training stage of the experiment, so exploration legs were unlikely to serve a similar purpose to orientation flights. They typically occurred after bees had visited several feeders but were less common after all five rewards had been consumed, so it is possible that bees were triggered to search for other food sources if they had visited all the feeders whose location they remembered, but were left unsated. Under this hypothesis, decreasing distance and duration of exploration legs would reflect increasing certainty about the position of all five feeders. Alternatively, Woodgate *et al*.^26^ suggest that bees may simply explore the landscape during the early stages of their foraging career, subsequently switching to efficient exploitation of the best foraging sources discovered. Thus, changes in exploration in this experiment might reflect a typical switch from exploration to exploitation behaviour. Finally, bees may explore in search of reserve food sources in case of changing resource distributions^27^. Foraging bumblebees switch flexibly between flower species^28,29^, so may explore for other flowers even when trained to a single feeder. We conducted the experiment in the autumn to limit the availability of natural flowers so reduced exploration might reflect the bees’ increasing certainty that there were no alternative food sources to be discovered.

We developed new techniques to compare flights in terms of their use of space and showed that flight paths became more similar to one another as bees gained experience. Much of this increasing similarity can be attributed to the pattern of increasing flight straightness over the same timescale but there is also evidence of repeatable, idiosyncratic flight paths (see for example, the crooked yet repeatable first leg of bee 2’s foraging bouts between supplementary Figs S66-S80). Similarity scores should be taken to indicate rates of change: increasing similarity demonstrates that the rate of change slows dramatically over the course of a day, but our bees’ routes continued to develop, and did not become completely stereotyped even after 50 or 60 foraging bouts. It is unclear whether they would have done so over longer timescales but the flights we recorded in this experiment probably represent a significant proportion of the number of bouts most bumblebees would undertake in a lifetime^26^, so there may not be any time for further stabilisation to occur under natural conditions.

One reason why path lengths remained longer than necessary and routes never became fully stereotyped was the phenomenon of frequent revisits to empty feeders. This may occur because bees rechecked familiar feeders in case they had refilled; our analysis suggested that 58% of approaches to recently depleted feeders involved landings on the platform and that is likely an underestimate since bees can check a feeder for food too fast for the radar to pick up. It is also possible that bees recapitulate familiar segments of route, using the feeders and landmarks to aid navigation. It is unlikely that bees could see one feeder from another (see Material & Methods) but without further experimentation it is not possible to be certain at what distance they can detect them. It is possible that stochasticity in when a bee catches sight of familiar but hard-to-see targets like our feeders, and consequent variation between visually-guided and memory-driven processes of navigation, may explain some of the variation we observed in route structure.

The majority of sub-sequences of feeder visits closely fitted an exponential distribution (supplementary Fig S211), a hallmark of random processes, strongly suggesting that these segments of route were chosen by rules that were at least partly stochastic. However, the most commonly seen segments of route were a poor fit for an exponential distribution, consistent with the idea that these common movements were determined by memory while stochastic choice of rarer segments generated continual variation, allowing bees to search for more efficient visitation sequences. This is consistent with a tenet of the traplining heuristic model^24^ - that routes develop by holding some feeder-to-feeder transitions constant while others continue to be swapped around - as demonstrated graphically in the supplementary videos (S1–S3) where certain segments of path were continually reinforced while other segments of the route gradually fade, since they were not repeated in subsequent bouts. Interestingly, the repeatable and rarer path segments were not distributed evenly throughout the visit sequence: destinations of the earliest legs in each bout became highly repeatable, while later parts of the visitation sequence continue to change. Potentially, memorising the beginning of a route while continuing to experiment with later visits might allow a bee to search for more efficient routes while exploiting the increased speed possible on familiar routes.

None of our bees continued to visit feeders in the order that they were discovered, supporting previous observations^16,20^. Only one ever visited the feeders in the order necessary to minimise overall travel distance, broadly agreeing with Reynolds *et al*.^24^, who predicted that bees would be unable to form a stable trapline of minimal travel distance on a similarly structured array of 10 feeders. By contrast, when Lihoreau *et al*.^20^ tested bees on a regular pentagonal arrangement of feeders, all 7 of their subjects developed a trapline route which minimised both the overall travel distance and the individual feeder-to-feeder distances. Their routes were also more repeatable (sequence similarity score between the last two foraging bouts on the first day of tracking for each bee: Lihoreau *et al*.^20^, 0.89 ± 0.07; this study, 0.73 ± 0.21). Our array had an identical number of feeders, total path length of the minimal-distance route and the same minimal-distance solution (travel around the outside of the array), to that used by^20^. While our relatively small sample size makes it impossible to conclude with certainty that bees can never choose the minimal distance solution on our array, it seems clear that such a solution is found more rarely than on the array used by Lihoreau *et al*.^20^. We suggest that the observed differences in route structure and repeatability are attributable to the different spatial relationships between feeders, specifically the fact that in our array the minimal distance sequence required bees to ignore the short nearest-neighbour transitions that were possible between feeders #1 and #5, and between #2 and #4.

The nearest-neighbour route between feeders in the array used by Lihoreau *et al*.^20^ was identical to the minimal overall distance route, so the two strategies could not be distinguished. In our study most bees did not use full nearest-neighbour routes, but it is possible to prioritise low travel distances without the entire feeder visit sequence being determined solely by nearest-neighbour transitions. Over half of all legs on our array were nearest-neighbour movements and the mean distance rank was 1.45, suggesting that our bees prioritised low between-feeder distances. This preference for closer feeders was remarkably consistent across all bees and mirrors the results of a similarly structured array on a smaller spatial scale^12^. The traplining heuristic model^24^ proposed that bees may use an iterative improvement process of route development whereby the probability of flying between any pair of locations is enhanced or decreased as a function of the length of past routes using the same transitions. If probabilities were also influenced by the length of individual legs (with a higher weighting given to short travel distances) this would lead to routes in which low between-feeder travel distances were prioritised but which were flexible enough to also include non-nearest-neighbour transitions. This hypothesis is consistent with the observation that the bees in Lihoreau *et al*.^20^ did not exclusively make nearest neighbour transitions, the fact bees were less likely to hit on the minimal-distance sequence in our array than^20^, and the fact that our bees’ routes were less stable than theirs (because of the conflict between minimising travel distance in each leg and minimising overall route length).

One striking pattern in the flights of those bees we tracked over two days was an increase in the distance flown during exploration flights and a corresponding drop in flight path straightness and repeatability, between days of tracking. This reflects an increase in exploration behaviour on the morning of the second day and, intriguingly, the changes were more noticeable in the flights of bee 1, which had a four-day gap between the first and second days of tracking. It is unclear whether this resurgence of exploration was caused by degradation in memory, preventing bees from resuming the previous day’s route or whether bumblebees have a general tendency to explore at the beginning of each day. Such a strategy might allow them to respond effectively to changes in the distribution of floral resources over the course of their foraging career (although there is little support for this hypothesis in the flight paths recorded by Woodgate *et al*.^26^).

Care must be taken in extrapolating our results to the population as a whole since the tracks are derived from only 6 bees. Understanding how the habitual routes of central place foragers develop with experience requires long-term tracking of individuals throughout the entire duration of the experiment, which no previous study of spatial orientation and learning has accomplished. The complete dataset is among the largest harmonic radar datasets ever recorded (exceeded only by^26,30,31^, which are limited by the lack of a comprehensive record of the flights of any individual). Radar tracking individual bees over multiple consecutive flights presents a significantly greater challenge than tracking individuals for one flight each so there was an unavoidable trade-off between the depth of data we could acquire on the development of individual bees’ routes and the total number of individuals we were able to track. We believe that the insights our data provide into the development of the individual bees we tracked justify that trade-off and could not have been obtained any other way.

One limitation of the relatively low number of individual bees we were able to track in this study is that we cannot investigate whether individual differences in their behaviours reflect different strategies for solving the Travelling Salesman Problem: the feeder visit sequences adopted by each bee became more repeatable with experience but did not converge on a single solution as did the bees in^20^. Were several different stable solutions reached by different strategies or does the sequence preferred by each bee simply reflect the stochastic nature of a single heuristic strategy? Bumblebees show consistent individual differences in their fidelity to particular trapline routes and in the speed and accuracy of following favoured sequences^23^, but that study did not examine the structure or efficiency of their habitual routes, nor could it analyse the flight paths of the bees. In addition to idiosyncratic differences in behaviour, it is conceivable that space use strategies change with age or condition, for which we have no data on our focal bees. Further individual idiosyncrasies include the very digressive, lengthy exploration legs of bee 2 toward the end of its second day; or Bee 4 whose flight paths did not straighten (Fig. 4h), yet showed similar levels of flight path similarity to the other bees, suggesting the development of stable yet inefficient flight paths.

Our study supports the idea that there are some spatial arrangements of foraging resources for which bumblebee foragers are unable to develop a stable, efficient trapline route. Crystallisation of early legs of the route and a preference for short between-feeder travel distances both have the effect of greatly reducing the vast number of potential route options available and may, under many circumstances, allow bees to alight on a close-to-optimal route in a short space of time. These same heuristics can, however, lead to suboptimal routes under certain conditions such as the array we designed for this experiment. Nonetheless, bees are able to adopt efficient routes rapidly by refining their flight paths, as well as their visit sequences.

## Material and Methods

### Study site

Field work took place from July to October 2014, April 2015, September to October 2015 and May to June 2017, at Rothamsted Research, Hertfordshire, UK (51°48′35"N 0°21′23"W). Attempts to train bees to visit our feeders in July and August 2014, April 2015, and May and June 2017 were unsuccessful as the bees showed a strong preference for natural forage sources. Consequently, data collection could only take place late in the season when there were few naturally occurring sources of food in the area. Bees were tracked in two locations: In location 1, the nest was situated at the East margin of a recently harvested wheat field (approx. 250 × 175 m), with the feeder array extending West into the field and the radar at the North-western corner, approximately 220 m from the nest. In location 2, the nest was placed at the edge of a field of mown grass (approx. 700 × 300 m), with the feeders extending roughly North-west into the field and the radar at the Northern edge of the field, approximately 300 m from the nest. In both cases there were linear landscape features such as paths, field edges and hedgerows, but the feeder arrays were placed in locally homogenous areas.

### Bees and initial training

Our study subjects were *Bombus terrestris audax* from commercially sourced colonies (Biobest NV, Westerlo, Belgium). Colonies were kept in wooden nest boxes (30 × 21 × 16 cm high), which were kept in a wooden shed to provide shelter and further insulation. A Perspex tunnel (26 × 4 × 4 cm high) allowed access to the outside world through a hole in the shed wall (Ø = 50 mm). The colony was fed with pollen directly in the nestbox every day and with 40% sucrose solution (w/w) in a gravity feeder every few days to ensure that the colony never ran out of food in the honey pots.

During the experiment, focal bees fed from artificial feeders consisting of a blue painted wooden platform (20 × 20 cm) mounted on top of a 92 cm white plastic pole (Ø = 40 mm; Fig. 1a). Precisely measured volumes of 40% sucrose solution were placed in a well drilled into a small blue acrylic chip (2.5 × 2.5 × 0.5 cm high) on the platform, using an electronic pipette (HandyStep, BrandTech Scientific Inc., Essex, CT, US).

To train bees at these artificial feeders, the colony was placed inside a flight room (390 × 285 × 230 cm high), with three feeders which were kept constantly topped up to allow *ad libitum* feeding. Feeding workers were marked using numbered tags (Opalith Zeichenplättchen Leuchtfarben, Bienen-Voigt & Warnholz, Ellerau, Germany). After three days, the colony was transferred to the field and two feeders set up directly outside the nest entrance. These feeders were gradually moved away from the nest over the course of a day until they were 50 m from the colony entrance. The bee that visited the feeders most regularly was chosen as our focal bee. We used tunnel gates to confine all other bees to the nest and only allowed the focal bee to forage. We had no information on the ages of our focal bees. No bees had any flight experience prior to training and all experienced similar amounts of training but, since the experiment involved free-flying bees in an outdoor field site, we were unable to control for any differences in how they explored the landscape during training.

We measured the functional crop volume of each focal bee by allowing it to forage at a row of five feeders placed next to each other, 50 m from the nest. In 2014 we attached transponders to the focal bees before measuring the crop volume; in 2015 and 2017 we measured the crop volume first and only attached transponders to bees whose crops had been measured successfully. We adjusted the volume of sucrose between visits by the bee until we found the maximum volume at which the bee would consume all five reward droplets before returning to the nest. During the experiment all feeders contained this volume of sugar solution (1/5 of the crop capacity per feeder) to ensure that the bee was motivated to visit all five feeders but would then be sufficiently full to return to the nest without foraging further. A further adjustment of the reward volume was necessary for one bee, which showed a tendency to continue to explore the field after consuming the contents of all feeders (see supplementary Table S1).

There was a high rate of attrition at every step of training. We estimate that no more than 5–10% of bees that fed at our feeders in the flight room continued to do so in the field during September and October (and considerably fewer earlier in the summer). Approximately 50 (±20%) of bees that foraged on the feeders immediately in front of the shed were still regular visitors once the feeders had been moved to 50 m. No more than 4–6 regular foragers were ever available at 50 m. Non-focal bees were confined to the nest during the time we measured the crop capacity of the focal bee. If the focal bee could not be tracked, it was rare for any previously regular foragers to still be motivated to visit the feeders so training typically had to begin again. Over the course of the study we attempted to measure crop volumes for 24 focal bees. Crop measurement could not be completed for 9 bees as they stopped foraging and remained in the nest; after crop measurement, but before they could be tracked on the experimental array, five more bees appeared to lose motivation and stopped foraging, while two died. We attempted to track the remaining 8 bees but two stopped visiting the feeder at the training point, presumably visiting wild flowers instead, so could not be tracked on the array and were abandoned after 4 and 7 unsuccessful flights, respectively. Six focal bees were successfully tracked as they foraged on the experimental array.

We continuously monitored these bees and tracked every foraging bout they made. Bees 1 and 2 were tracked at location 1 over two full days of foraging (supplementary Table S1). Bee 1 had a four day break between the first and second day of tracking due to difficulty locating it at the start of the second day of tracking. Bees 3–6 were tracked at location 2. Bee 3 was tracked for two bouts toward the end of one day and then for a subsequent two full days. Bees 4 and 6 performed only 6 and 9 foraging bouts respectively, subsequently remaining in the nest and never emerging to forage again. Bee 5 was tracked for 27 bouts over the course of one full day but could not be tracked on the following two days due to bad weather and died of natural causes before tracking could be resumed.

### Experimental procedure

Once the focal bee had completed at least five foraging bouts visiting the feeders in their start position, we moved the feeders into the experimental positions. The experimental array comprised five feeders and the nest, forming a hexagon in which the distances between feeders forming the edges of the hexagon were 50 m, but the distance across the body of the hexagon was only 25 m (Fig. 1). The shortest route to visit all five feeders and return to the nest was around the edges of the hexagon (total length: 300 m; Fig. 1a). A route dominated by nearest-neighbour movements between feeding stations would include two transitions across the short axis of the array and a long final leg (total distance: 346.8 m; Fig. 1b). Bumblebees’ failure to detect non-self-luminant objects subtending less than 3° of visual arc^32^, suggests the entire feeder apparatus should not be detectable from further away than 17.9 m (although recent results suggest insect vision may be more acute than previously believed^33^). If true, no feeder should be visible from any other although it is likely that both feeders 1 and 5 would be visible to a bee that returned to the position the feeders had been in during training. The bees were confined to the nest for approximately 4–5 minutes between bouts while an experimenter refilled the feeders.

### Harmonic radar tracking

All movements of the focal bees outside the nest were tracked using 32 mm harmonic radar^34^. The radar returned distance and direction coordinates of the bees’ position every 3 s while the bees remained in line-of-sight within a radius of about 800 m (accuracy ≈ ± 2 m). Radar transponders weigh around 15 mg, consist of a 16 mm vertical dipole and were attached to the numbered tag on the thorax of the focal bees using superglue (Loctite Power Flex Gel, Henkel Ltd., Hemel Hempstead, UK). Bumblebees can carry nectar loads of up to 90% of their body mass^28^, and the transponder represents only 8–10% of a typical worker’s mass of 175–200 mg.

### Data analysis

A number of variables were derived from the radar track data. The five feeders and the nest will be referred to as *locations*. Bees were considered to have visited a location if they were detected within a 5 m radius. We also tried to deduce from the flight paths whether the bees had landed by identifying pairs of consecutive radar signals that were within 2.5 m of each other. The proportion of visits in which the bee was still for at least 2 consecutive rotations of the radar was 0.88 ± 0.16. Each path was broken down into a number of *legs,* each of which was defined as the segment of a track beginning when the bee left the vicinity of any location and ending when it next approached any location. Legs in which the bee remained within 50 m of at least one location were categorised as *within-array* and those in which it travelled more than 50 m from every location that that bee had previously discovered (i.e. it was further from any known location than the locations are from one another) were categorised as *exploration*.

*Path length* was the cumulative length of a straight-line path between every positional fix over the entire foraging bout and could be further broken down into distance travelled on within-array or exploration legs. *Straight-line path length* was calculated as the straight-line distance joining all the feeders visited in each bout in the order they were visited; this is the shortest distance a bee could travel while maintaining the observed visit sequence. *Bout duration* was the total time elapsed between the bee’s departure from the nest and its return. The *straightness* of each leg of a track was defined as the distance component of the vector sum of the straight lines connecting every pair of positional fixes within that leg and can range from 0 (datapoints form a circle) to 1 (the path forms a straight line). The straightness of an entire bout was the mean of the straightness score for all legs; a score of 1 would indicate that the route was composed of perfect straight lines between locations, although there may be changes of direction from one leg to the next.

The radar data tells us the bees’ position every 3 s on average but not where they were between radar fixes. We used a method derived from Brownian bridge movement models^21^ to estimate the probability of the bees’ location between radar positional fixes. The landscape was broken down into 5 × 5 m pixels and the time interval between each pair of consecutive datapoints into 50 *timeslices* of equal duration. The probability density function of the bee being at location *x, y* at time *t* is given by

1 $$P(x,y,t|{x}_{n},{y}_{n},{x}_{n+1},{y}_{n+1})=\frac{1}{2\pi {\sigma }^{2}}\exp (\frac{{(x-\overline{x})}^{2}+{(y-\overline{y})}^{2}}{2{\sigma }^{2}})$$

where:

2 $$\overline{x}(t)={x}_{n}+({x}_{n+1}-{x}_{n})\frac{t}{T}$$

3 $$\overline{y}(t)={y}_{n}+({y}_{n+1}-{y}_{n})\frac{t}{T}$$

4 $${\sigma }^{2}={s}^{2}\frac{{t}^{2}}{{T}^{2}}{(T-t)}^{2}$$

*T* represents the time elapsed between two consecutive radar fixes while *x*_*n*_, *y*_*n*_ and *x*_*n+1*_, *y*_*n+1*_ represent the coordinates of the two fixes (i.e. the bee’s position at *t* = 0 and *t* = *T*). Flight speed *s* was estimated for each bout from the radar data: datapoints that were less than 5 m apart were assumed to result from the bee being stationary rather than in flight and were excluded, then the total distance covered by the remaining datapoints was divided by the total duration. This procedure results in an estimate of the likelihood of the bee having passed through each pixel in the time between the two radar fixes, for which we know its position with certainty. The probability maps thus generated for each timeslice can be summed across all timeslices in a bout (or several bouts) to calculate the probability that the bee passed through any given pixel at any point during the foraging bout (Fig. 2, supplementary Figs S1-S201). The probability maps for two foraging bouts (or legs of foraging bouts) can be multiplied to give the probability that the bee passed through the same pixels in both bouts. A *flight self-similarity* score was derived from the resulting probability map by summing the probability at each pixel and standardising by dividing that sum by the sum of the probability map produced by multiplying the bout by itself. The resulting score can range from 0 for two flight paths that did not intersect at all, to 1 for two identical flights. To investigate the repeatability of flight paths while controlling for the fact that the bees did not always visit the feeders in the same order, we compared the probability map for each leg of each bout to that of the most recent occasion on which the bee flew between the same two locations. We took the mean of these similarity scores for each leg to produce the *mean leg similarity score* for each bout.

*Feeder visitation sequence* was the order in which visits to the feeders or nest occurred. The number of revisits to empty feeders was calculated as the total number of feeder visits minus the number of unique feeders visited. We followed Lihoreau *et al*.^20^ in defining a trapline as a visitation sequence (excluding revisits to emptied feeders) that was repeated on at least three consecutive bouts. If more than one such sequence occurred, the trapline was the most commonly occurring sequence. We investigated whether visitation sequences have a tendency to stabilise with experience using a similarity index (SI), as described by Saleh and Chittka^10^, which quantifies the similarity between pairs of visitation sequences. SI scores range from 0 for completely different sequences to 1 for identical sequences. The sequence *self-similarity score* was the SI generated by comparing the full sequence for each bout (including revisits to empty feeders) with the previous bout, and the *optimality score* was calculated by comparing each bout sequence to the ‘optimal’ sequence, defined as the order of visits that allows a bee to visit all five feeders with the shortest possible total travel distance (Fig. 1a). To establish whether two sequences of flower visits were significantly more similar than expected by chance, we computed 1,000 similarity indices from 1,001 visitation sequences, randomly generated with replacement (so that revisits to ‘empty’ feeders could occur). Because 95% of the randomly generated similarity indices fall below a threshold of 0.70, two sequences were significantly more similar than expected by chance (at the 1% level) if the similarity index is greater than this threshold. All bees exceeded this threshold multiple times (see Fig. 5a-d) demonstrating that feeder visit sequences were not random. We assigned a *distance rank* to each leg, where moving to the closest feeder was given a rank of 1, moving to the second closest feeder was given a rank of 2 and so on.

Complete feeder visitation sequences,

5 $$\{{n}_{(1)},{n}_{(2)},{n}_{(3)},\mathrm{..}.{n}_{(m)}\}$$

where *n* is any of the 5 feeders or the nest, were partitioned into *flight segments:* sub-sequences of length 2,

6 $$\{{n}_{(1)},{n}_{(2)}\},\{{n}_{(2)},{n}_{(3)}\},\mathrm{..}.\{{n}_{(m-1)},{n}_{(m)}\}$$

each of these sub-sequences were assigned a unique identifying number,

7 $$6{n}_{(i)}+{n}_{(i+1)}$$

Some of these flight segments occur with high probability while the majority occur with relatively low probability. The occurrence probabilities were ranked from highest to lowest, and plotted as histograms on log-linear scales (see supplementary Fig. S211). A straight-line relationship indicates an exponential distribution. This procedure was repeated for *flight segments* of length 3 and 4 by assigning unique numbers,

8 $${6}^{2}{n}_{(i)}+6{n}_{(i+1)}+{n}_{(i+2)}$$

9 $${6}^{3}{n}_{(i)}+{6}^{2}{n}_{(i+1)}+6{n}_{(i+2)}+{n}_{(i+3)}$$

To test whether the bees routes changed with experience, we tested for a difference in route characteristics between the first and last five bouts flown by bees 1–3 and 5 over the first day of tracking, using a series of ANOVAs (using the *anovan* function of Matlab (Mathworks Inc., Natick, USA)). Bees 4 and 6 were excluded because they did not perform enough bouts to gauge their improvement with experience. Dependent variables were path length, straight-line path length, straight-line/observed path length, bout duration, optimality score, straightness, sequence self-similarity score, flight self-similarity score and the mean leg similarity score. Experience level (first five bouts = 0; last five bouts = 1) was the predictor and bee ID was included as a random factor. To investigate whether an overnight break affected behaviour, another set of ANOVAs were used to compare the performance of bees 1–3 over the last five bouts flown on the first day with the first five bouts flown on the second day of tracking. Dependent variables were the total length of exploration legs in each bout and all variables that showed a significant effect of experience over the first day (path length, straight-line/observed path length, bout duration, optimality score, straightness, flight self-similarity score and the mean leg similarity score). All tests were two-tailed and significance was determined at α = 0.05.

### Data availability

All harmonic radar tracks of bee flights recorded in this study are available in graphical form as Supplementary Figs S1-S201. All data used in statistical analysis are available as Supplementary Dataset S1.

## Electronic supplementary material

Supplementary material

Supplementary video S1

Supplementary video S2

Supplementary video S3

Supplementary video S4

Supplementary video S5

Supplementary video S6

Supplementary Dataset 1
