# Supplementary material for: Continuous Radar Tracking Illustrates the Development of Multi-destination Routes of Bumblebees
Source: Sci Rep. 2017 Dec 11;7:17323. doi: 10.1038/s41598-017-17553-1 (PMC5725577; doi:10.1038/s41598-017-17553-1)
Supplement: Supplementary file 1 — Supplementary material [file 41598_2017_17553_MOESM1_ESM.pdf]

Supplementary information

Continuous Radar Tracking Illustrates the Development of Multi-destination Routes of Bumblebees

Joseph L Woodgate[1,2]*, James C Makinson[1,2], Ka S Lim[2], Andrew M Reynolds[2], Lars Chittka[1]

*Corresponding author. Email: j.woodgate@qmul.ac.uk

[1] Department of Biological and Experimental Psychology, School of Biological and Chemical Sciences, Queen Mary University of London, Mile End Road, London E1 4NS, UK

[2] Rothamsted Research, West Common, Harpenden, Hertfordshire AL5 2JQ, UK

Supplementary table

Supplementary Table S1: Details of six bees

	**Bee 1**	**Bee 2**	**Bee 3**	**Bee 4**	**Bee 5**	**Bee 6**
Dates tracked	16/09/14 21/09/14	17/09/14 18/09/14	25/09/14 26/09/14	28/09/14	03/10/14	28/09/15
No foraging bouts	47	51	61	6	27	9
Location of feeder array	1	1	2	2	2	2
Volume sucrose solution per feeder (µl)	18 (bouts 1-13)<br>19 (bouts 14-21)<br>20 (bouts 22-47	20	16	20	19.5	14
Trapline sequence	1-2-3-4-5	5-4-2-3-1	5-1-2-3-4	N/A	5-1-2-4	N/A
First bout in which trapline appeared	34	24	17	N/A	20	N/A
Largest number of consecutive trapline bouts	4	5	12	N/A	3	N/A
Total number of trapline bouts	15	17	26	N/A	6	N/A

Supplementary figures

Supplementary Figs S1-S201: Flight paths of every flight made at feeder array by six bees. Each figure represents a single foraging bout by one bee. Bee ID, flight number, date of recording and the duration of each flight are shown on each figure. Nest position is represented in each panel by an open circle and the positions of the feeders by closed circles. Scale bars represent a distance of 25m. a) Flight path of the bee during each bout. Consecutive radar observations of the bee's location are joined by straight lines. The foraging bout is broken down into 'legs', each of which encompasses the portion of the flight path which began when the bee left a feeder or nest and ended when it came within 5m of another feeder or the nest. Each leg is represented by a different colour. Any leg during which the bee travels further than 50m from all the feeders it has previously visited is defined as 'exploration' and represented by a dashed line. b) Heat map showing the probability that the bee passed over each part of the landscape during each bout, as estimated from the radar tracks using a Brownian bridges technique (described in Methods). Each pixel represents an area 5 x 5m.

Fig S1

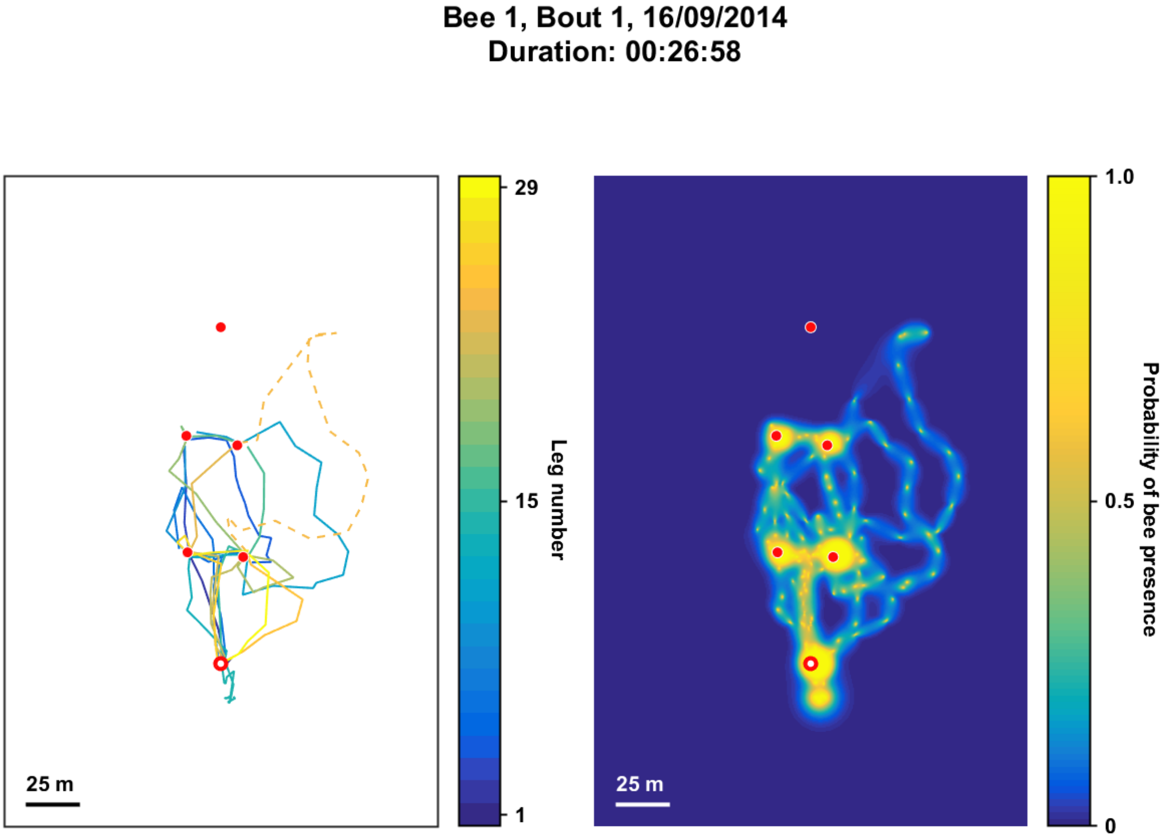

Bee 1, Bout 1, 16/09/2014
Duration: 00:26:58
29
15
1
Leg number
25 m
1.0
0.5
0
Probability of bee presence
25 m

Fig S2

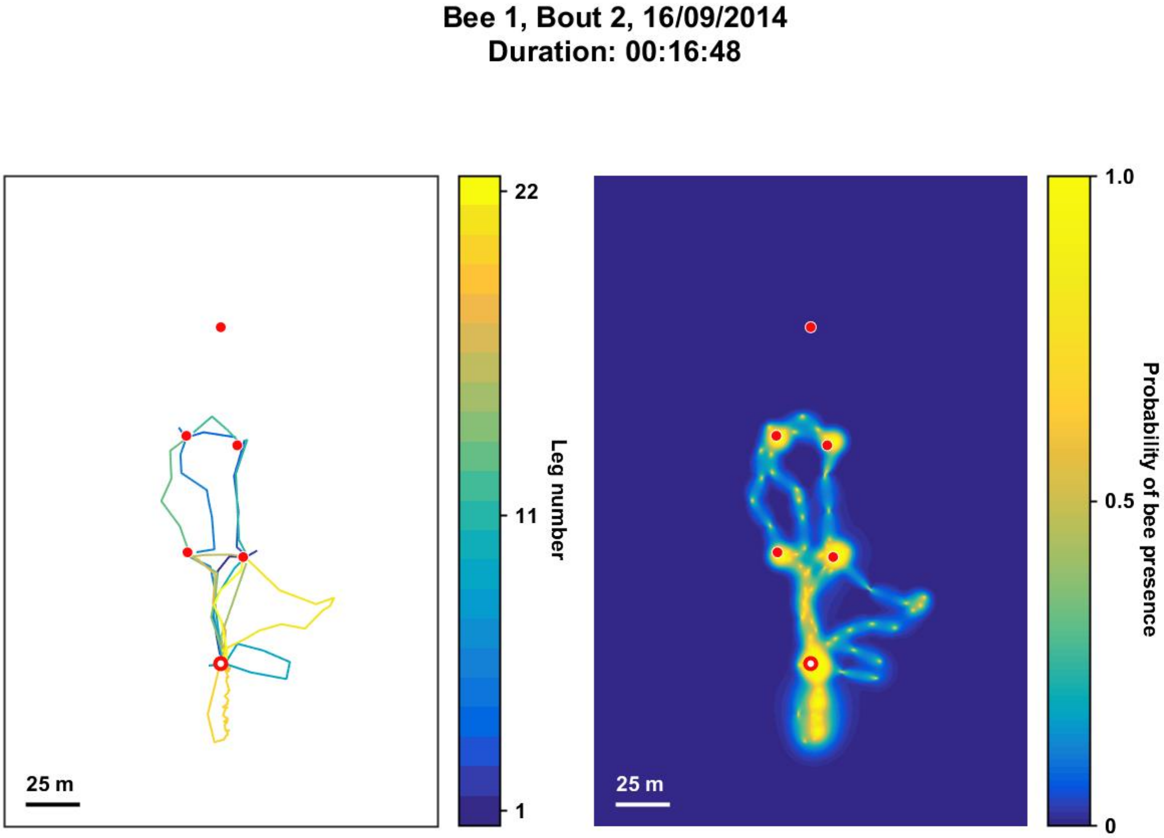
Bee 1, Bout 2, 16/09/2014
Duration: 00:16:48
22
11
1
Leg number
25 m
1.0
0.5
0
Probability of bee presence
25 m

Fig S3

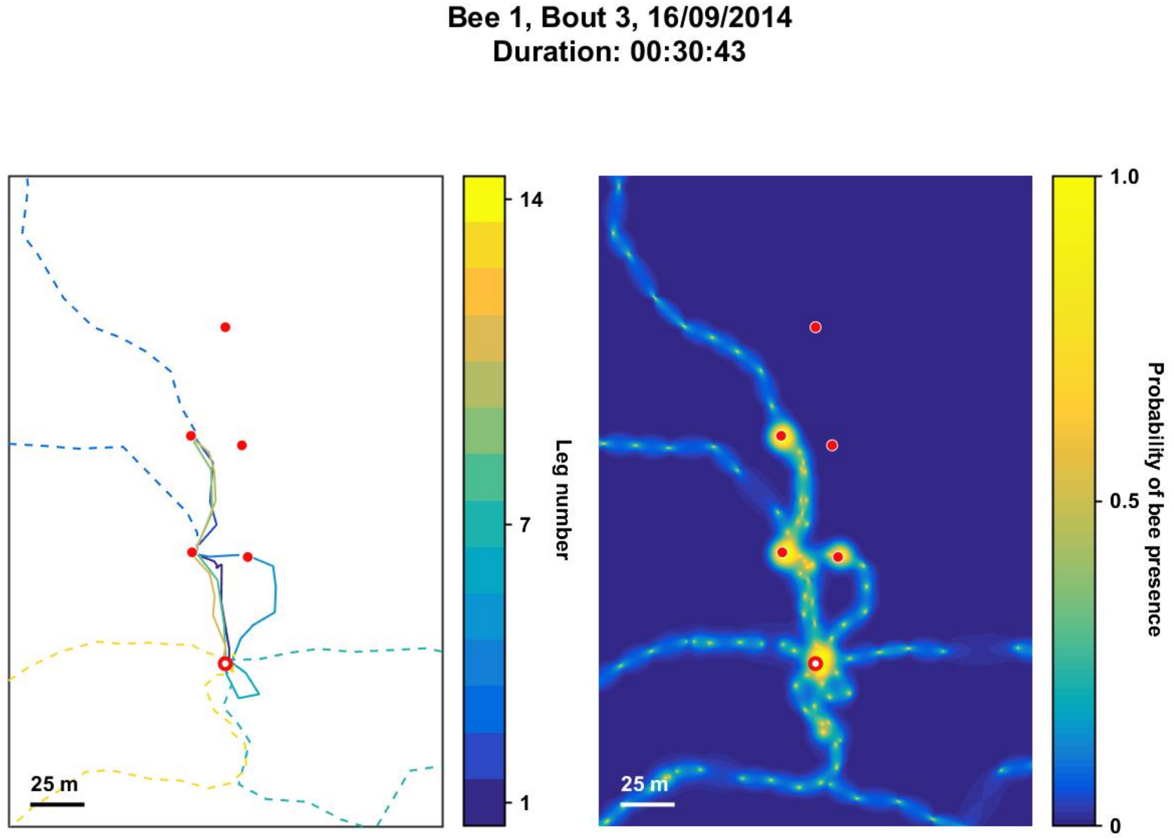
Bee 1, Bout 3, 16/09/2014
Duration: 00:30:43
25 m
Leg number
14
7
1
25 m
Probability of bee presence
1.0
0.5
0

Fig S4

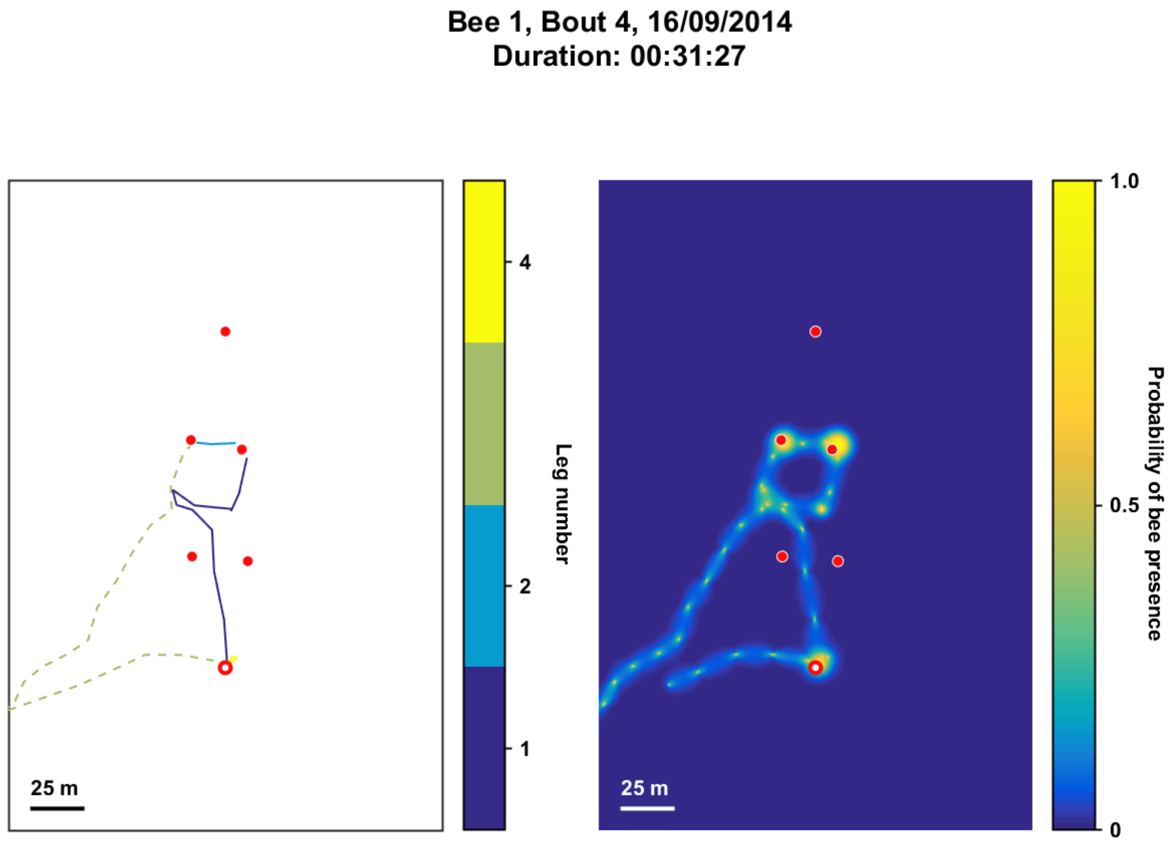

Bee 1, Bout 4, 16/09/2014
Duration: 00:31:27
25 m
Leg number
1
2
4
25 m
Probability of bee presence
0
0.5
1.0

Fig S5

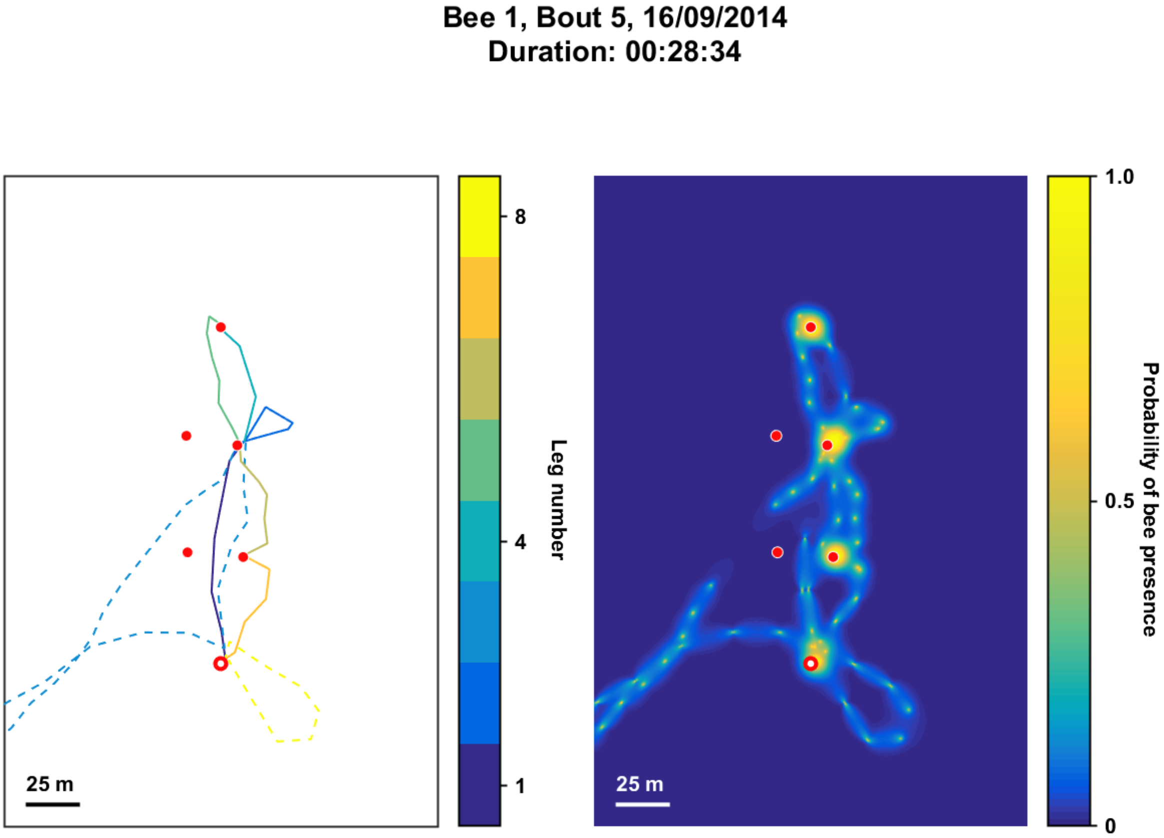
Bee 1, Bout 5, 16/09/2014
Duration: 00:28:34
25 m
8
4
1
Leg number
25 m
1.0
0.5
0
Probability of bee presence

Fig S6

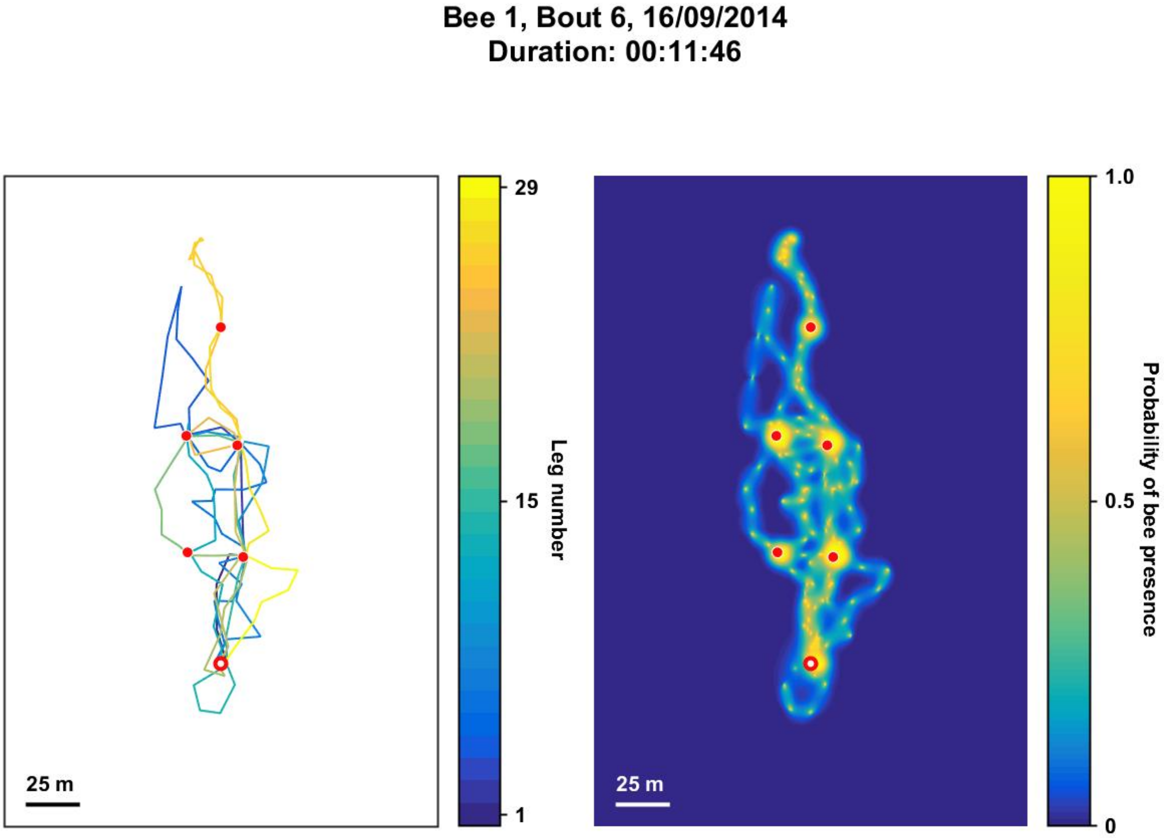
Bee 1, Bout 6, 16/09/2014
Duration: 00:11:46
25 m
29
15
1
Leg number
25 m
1.0
0.5
0
Probability of bee presence

Fig S7

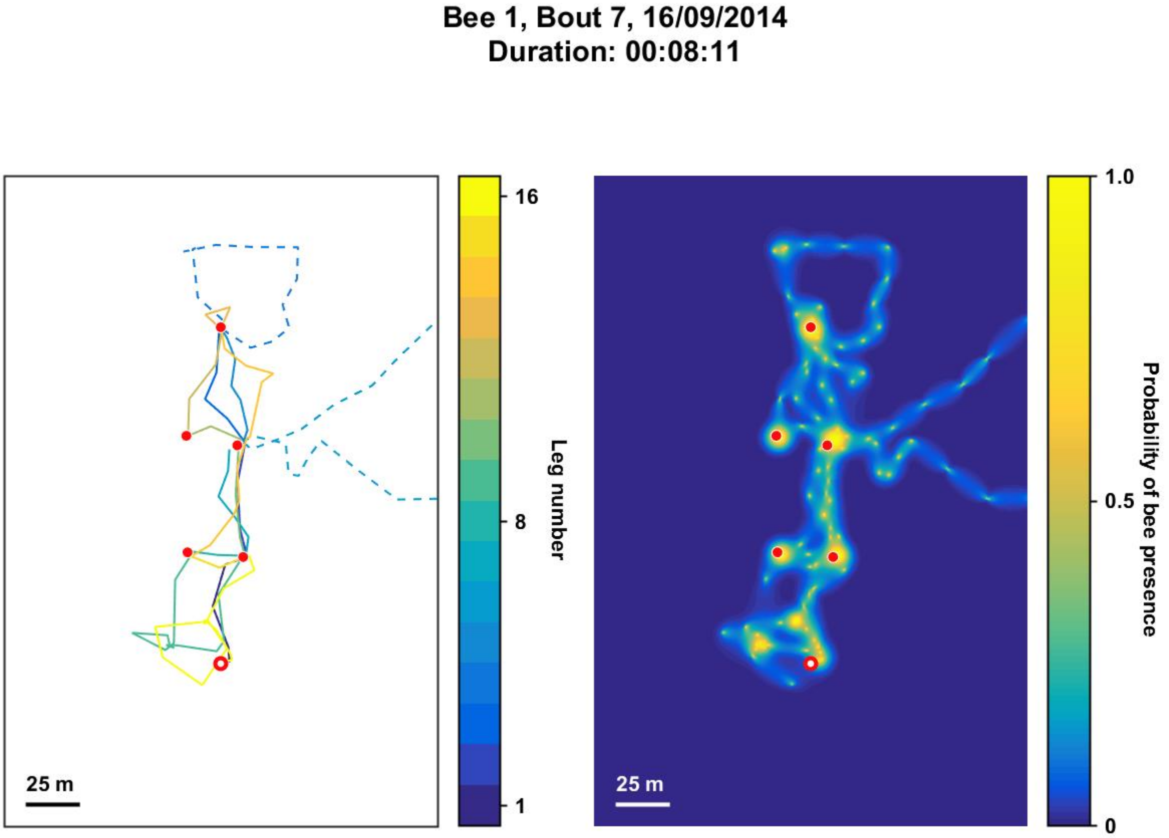

Bee 1, Bout 7, 16/09/2014
Duration: 00:08:11
25 m
Leg number
16
8
1
25 m
Probability of bee presence
1.0
0.5
0

Fig S8

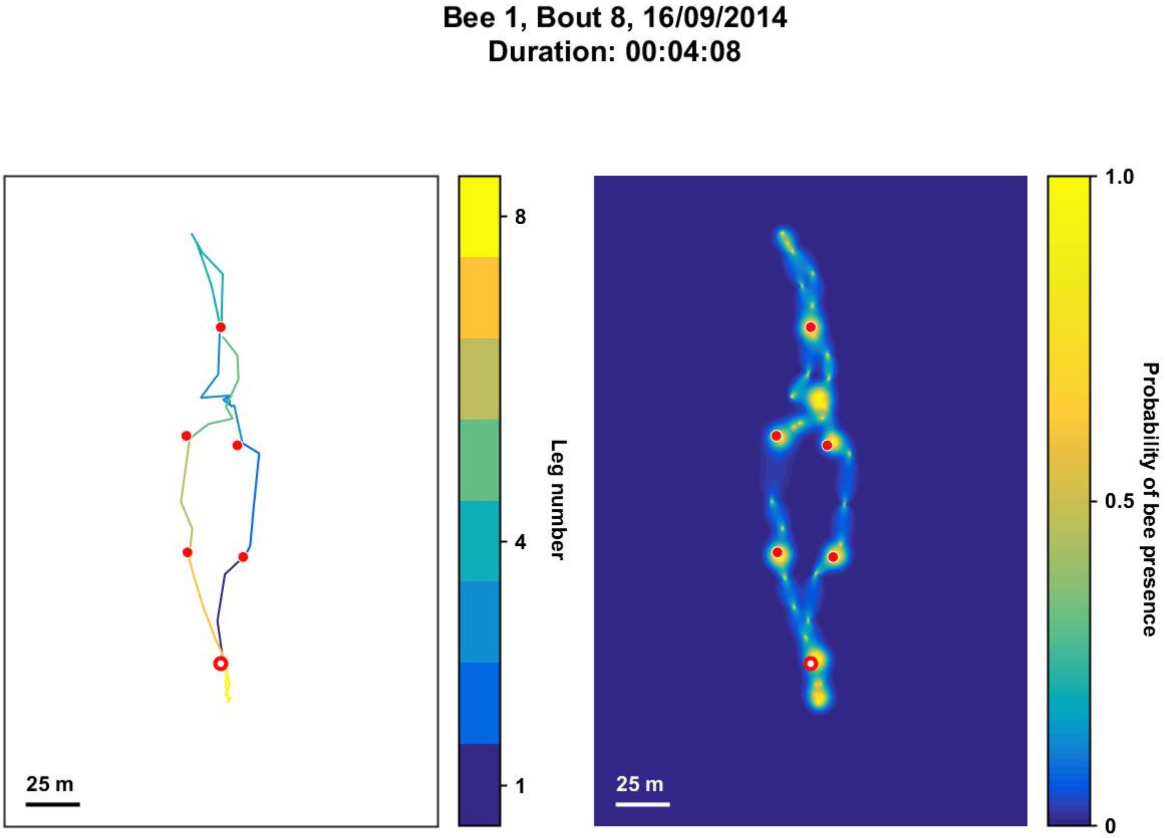

Bee 1, Bout 8, 16/09/2014
Duration: 00:04:08
25 m
Leg number
8
4
1
25 m
Probability of bee presence
1.0
0.5
0

Fig S9

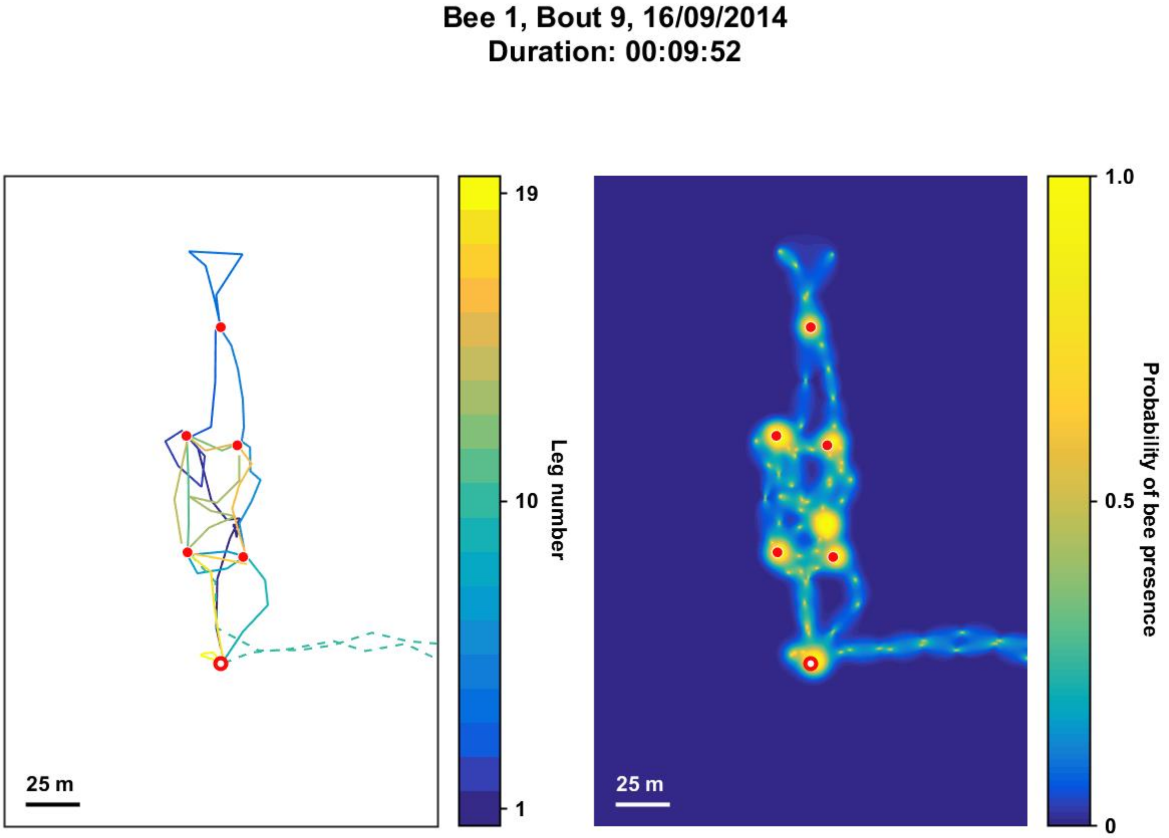
Bee 1, Bout 9, 16/09/2014
Duration: 00:09:52
25 m
19
10
1
Leg number
25 m
1.0
0.5
0
Probability of bee presence

Fig S10

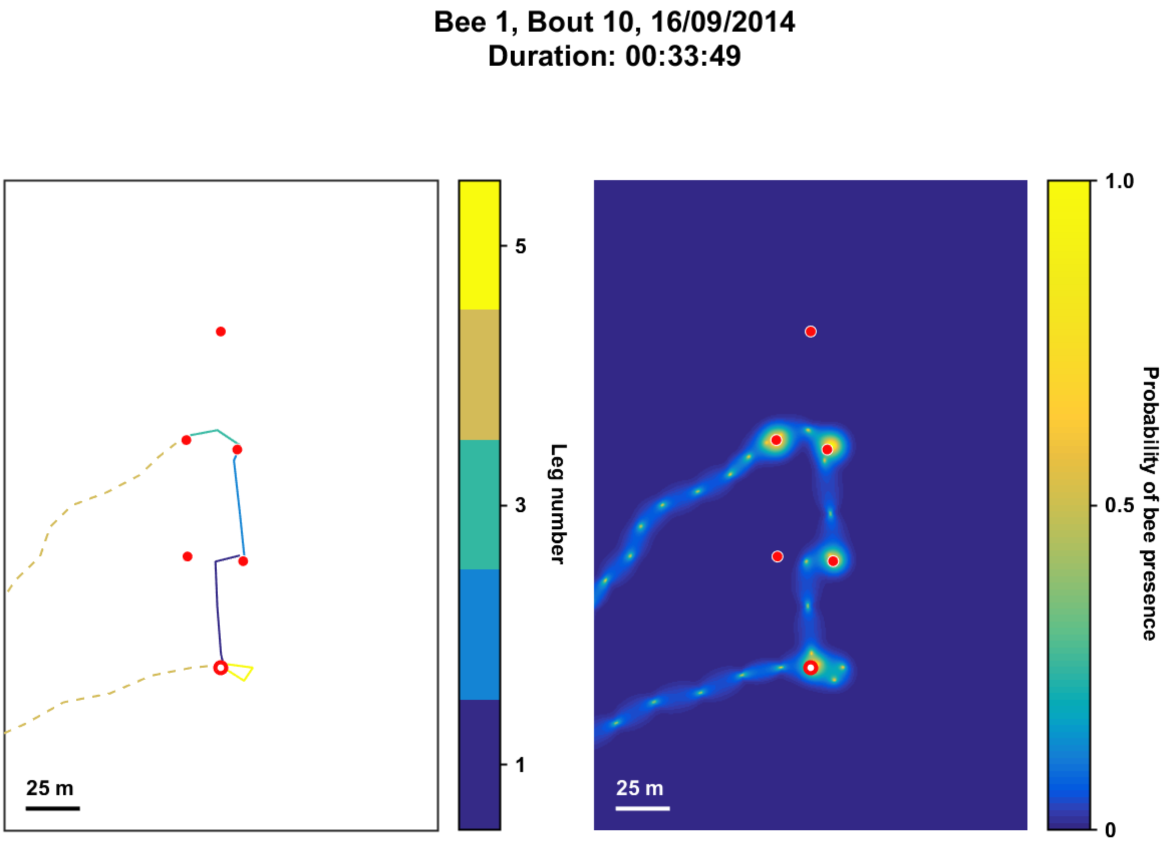
Bee 1, Bout 10, 16/09/2014
Duration: 00:33:49
25 m
5
3
1
Leg number
25 m
1.0
0.5
0
Probability of bee presence

Fig S11

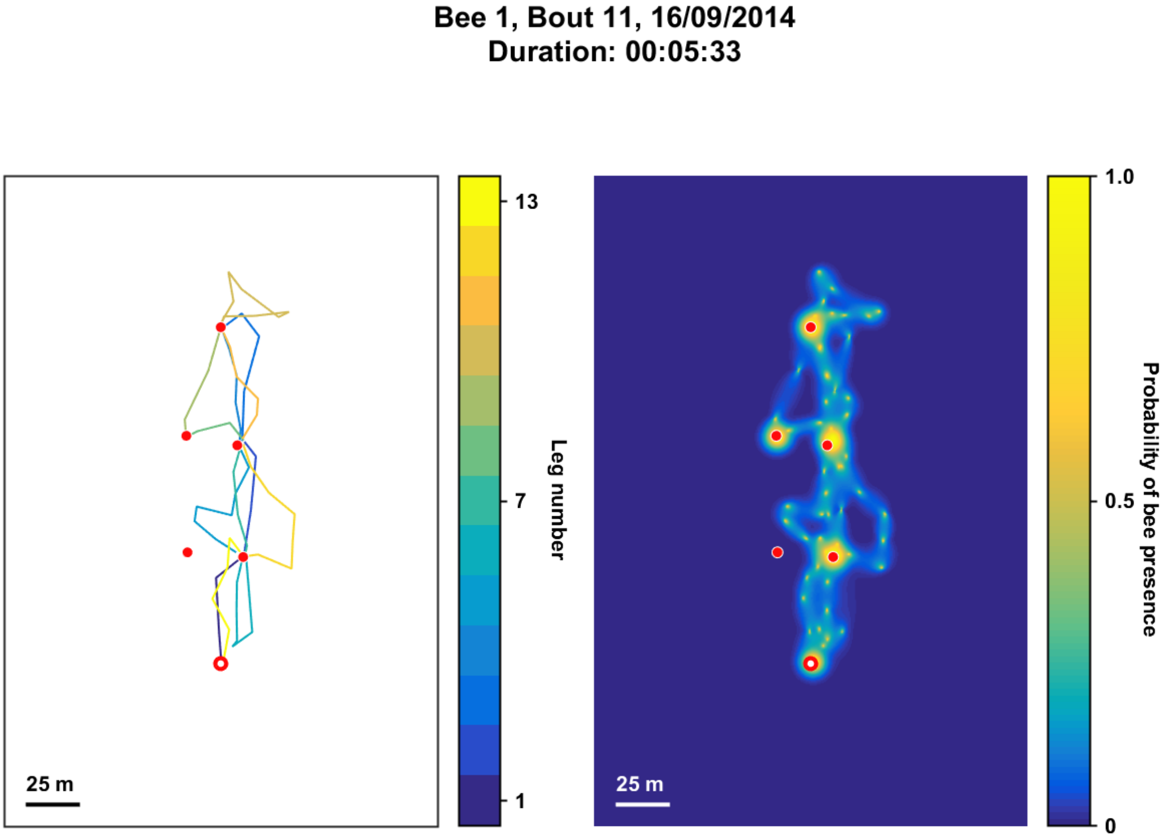

Bee 1, Bout 11, 16/09/2014
Duration: 00:05:33
25 m
13
7
1
Leg number
25 m
1.0
0.5
0
Probability of bee presence

Fig S12

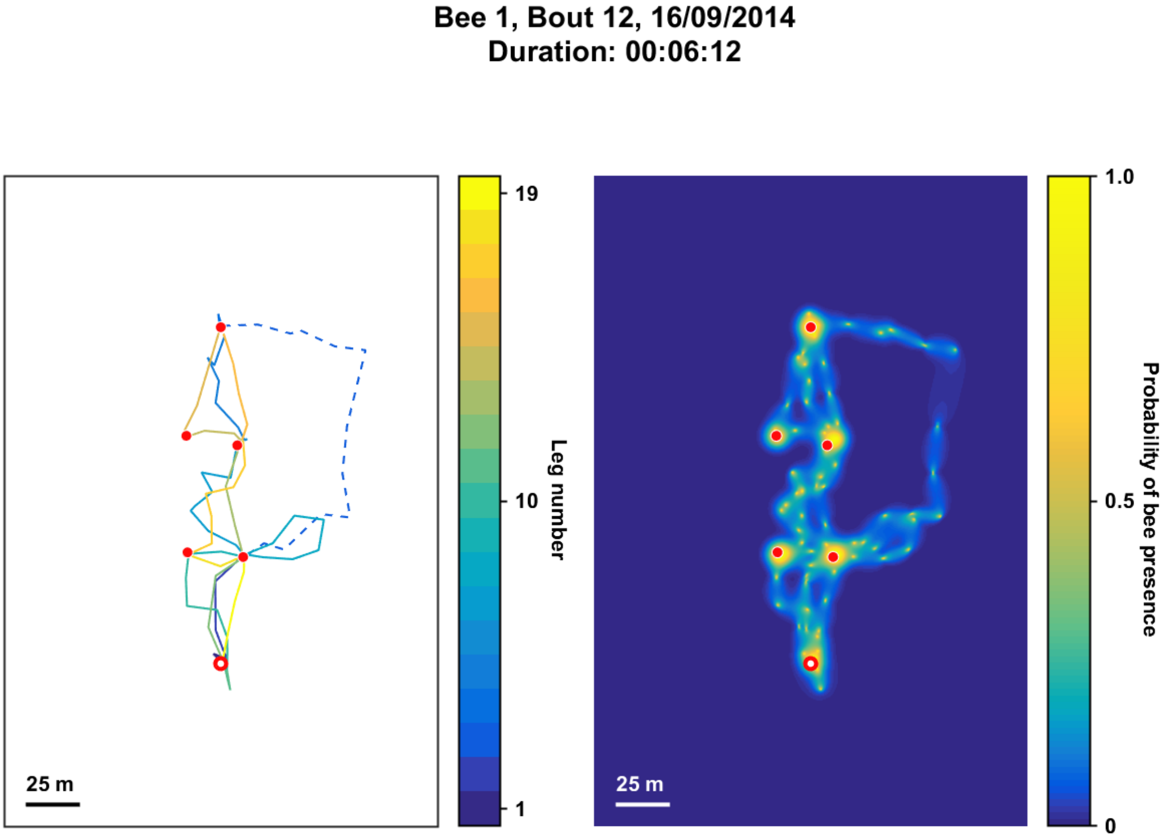
Bee 1, Bout 12, 16/09/2014
Duration: 00:06:12
25 m
19
10
1
Leg number
25 m
1.0
0.5
0
Probability of bee presence

Fig S13

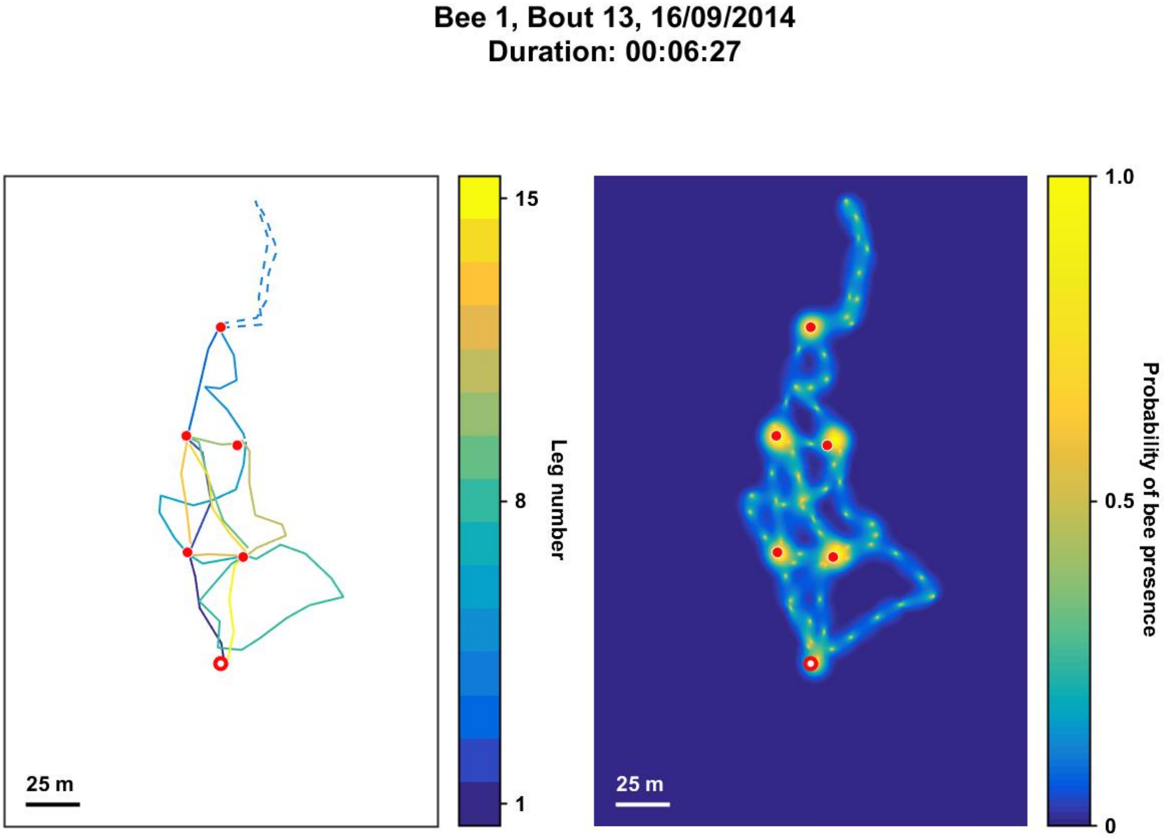

Bee 1, Bout 13, 16/09/2014
Duration: 00:06:27
25 m
15
8
1
Leg number
25 m
1.0
0.5
0
Probability of bee presence

Fig S14

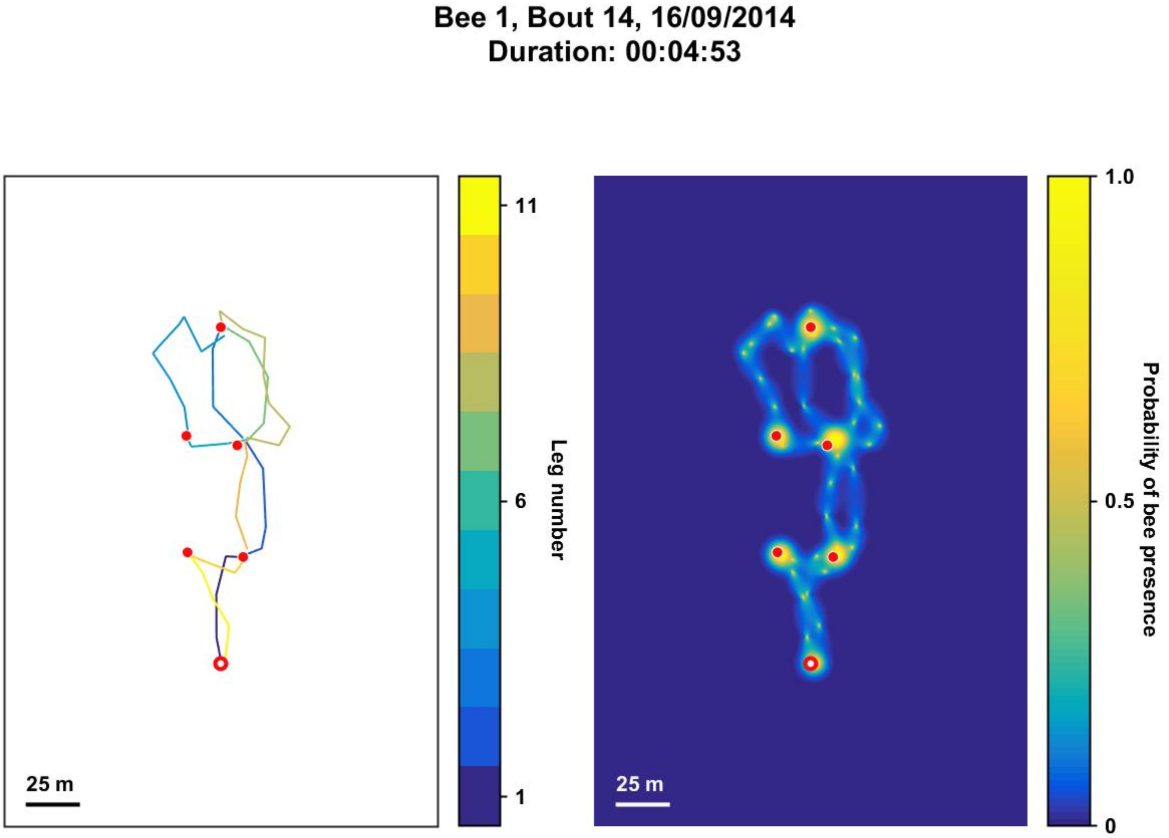

Bee 1, Bout 14, 16/09/2014
Duration: 00:04:53
25 m
Leg number
11
6
1
25 m
Probability of bee presence
1.0
0.5
0

Fig S15

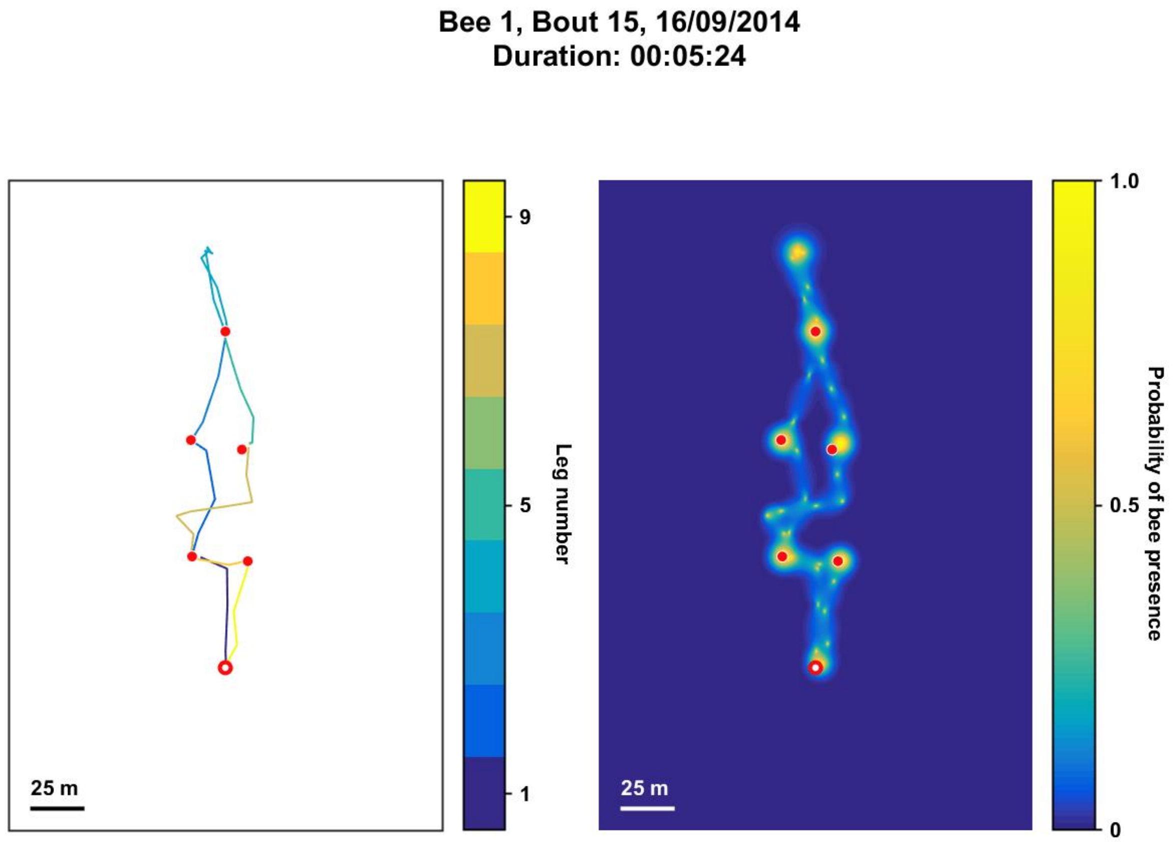

Bee 1, Bout 15, 16/09/2014
Duration: 00:05:24
25 m
9
5
1
Leg number
25 m
1.0
0.5
0
Probability of bee presence

Fig S16

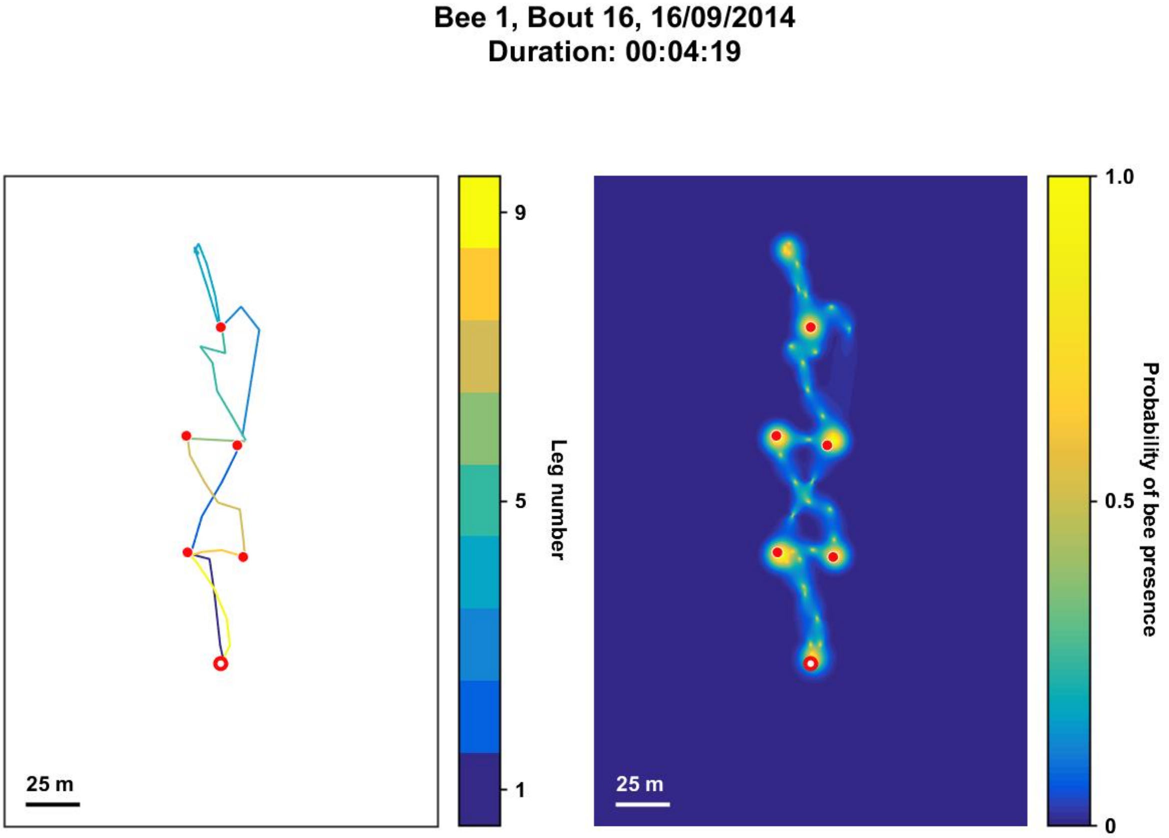

Bee 1, Bout 16, 16/09/2014
Duration: 00:04:19
25 m
9
5
1
Leg number
25 m
1.0
0.5
0
Probability of bee presence

Fig S17

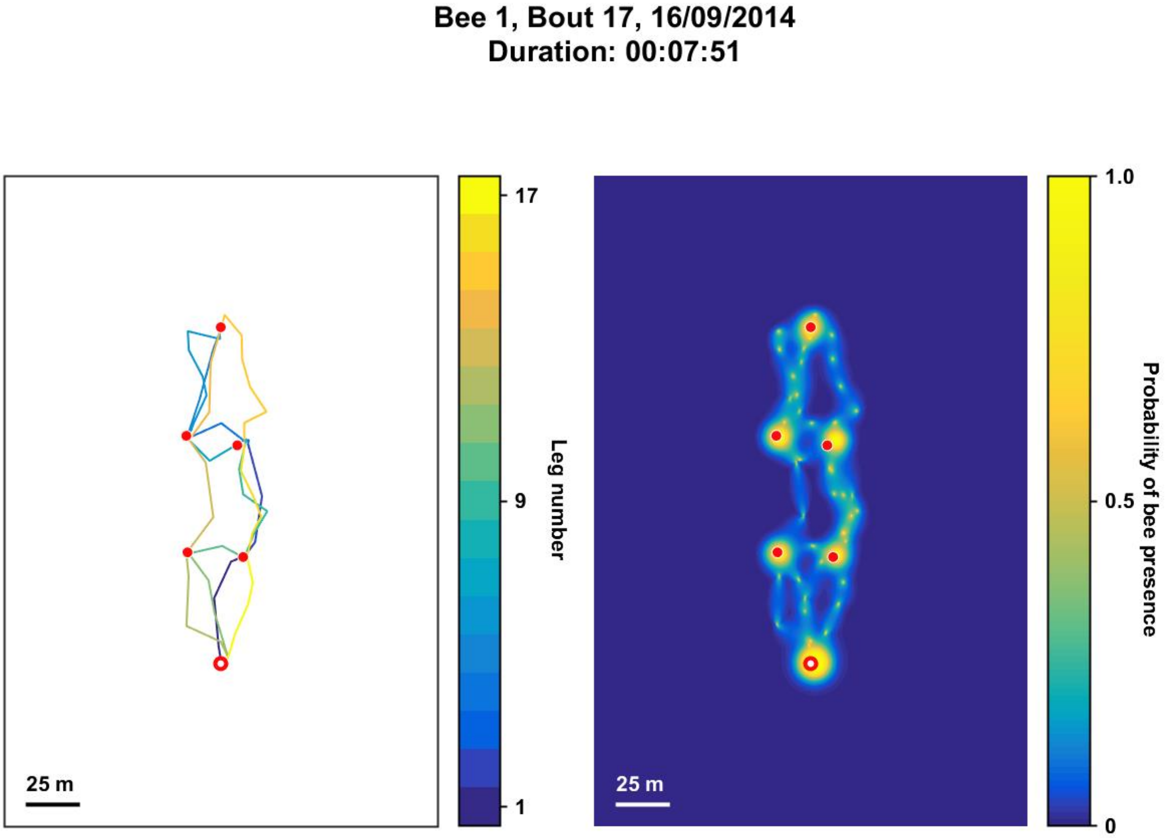
Bee 1, Bout 17, 16/09/2014
Duration: 00:07:51
25 m
17
9
1
Leg number
25 m
1.0
0.5
0
Probability of bee presence

Fig S18

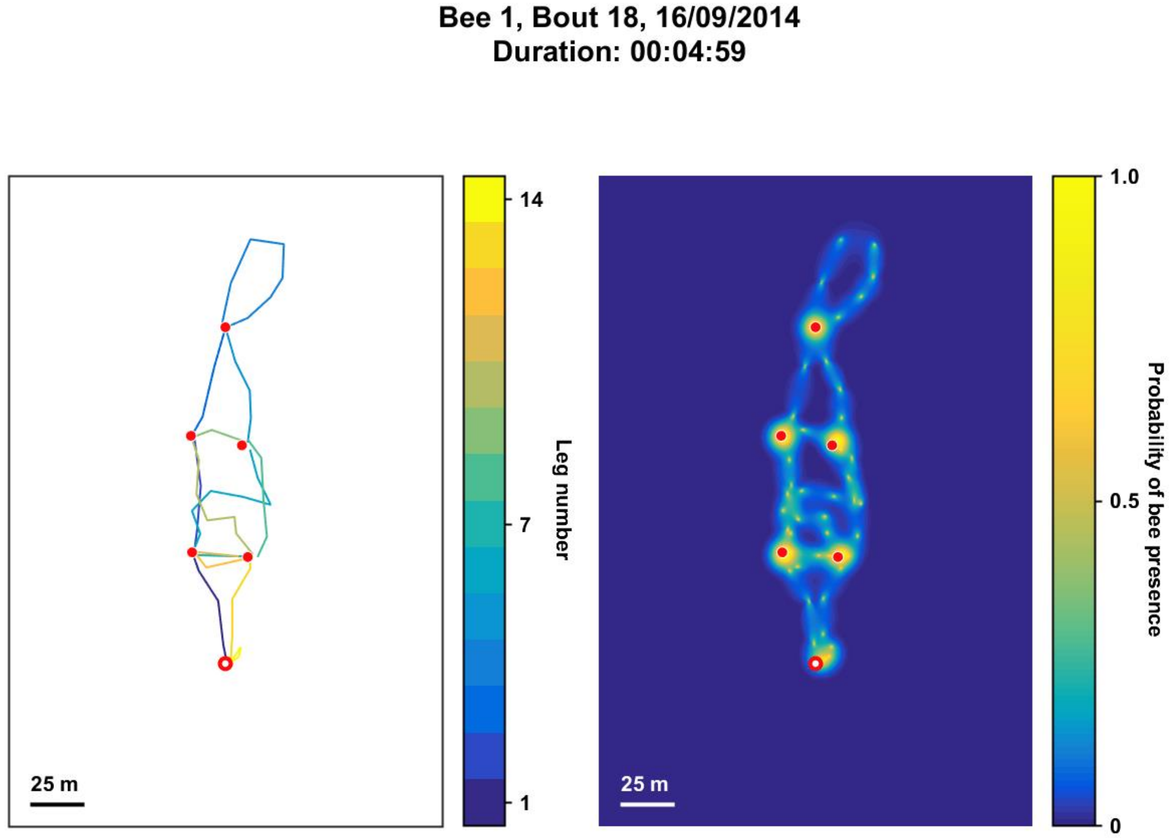
Bee 1, Bout 18, 16/09/2014
Duration: 00:04:59
25 m
14
7
1
Leg number
25 m
1.0
0.5
0
Probability of bee presence

Fig S19

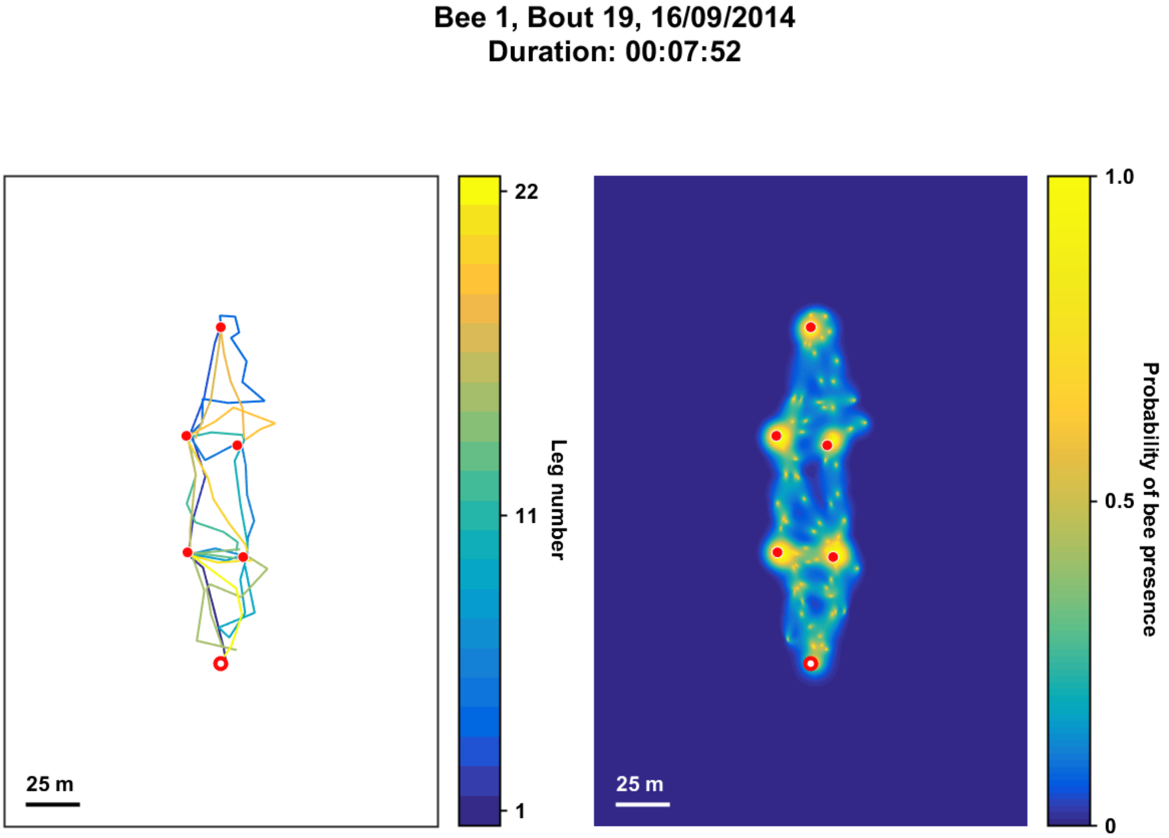
Bee 1, Bout 19, 16/09/2014
Duration: 00:07:52
25 m
22
11
1
Leg number
25 m
1.0
0.5
0
Probability of bee presence

Fig S20

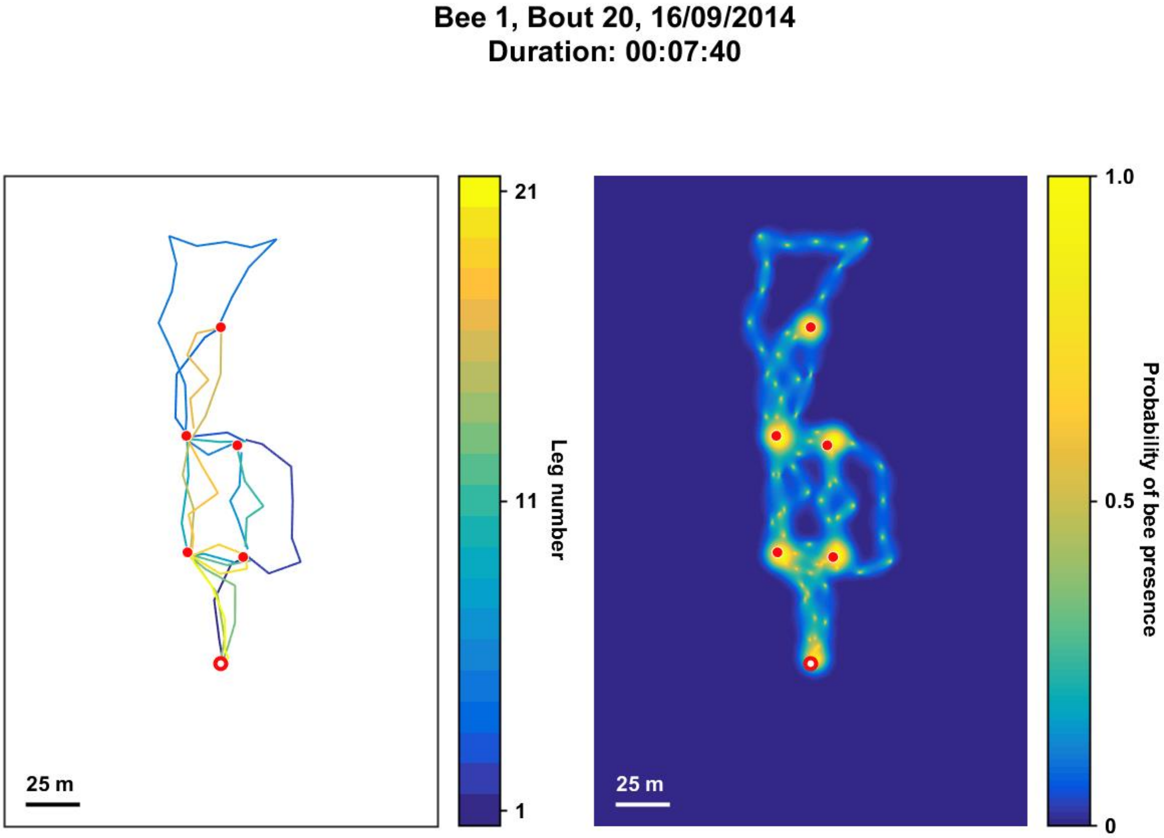
Bee 1, Bout 20, 16/09/2014
Duration: 00:07:40
25 m
21
11
1
Leg number
25 m
1.0
0.5
0
Probability of bee presence

Fig S21

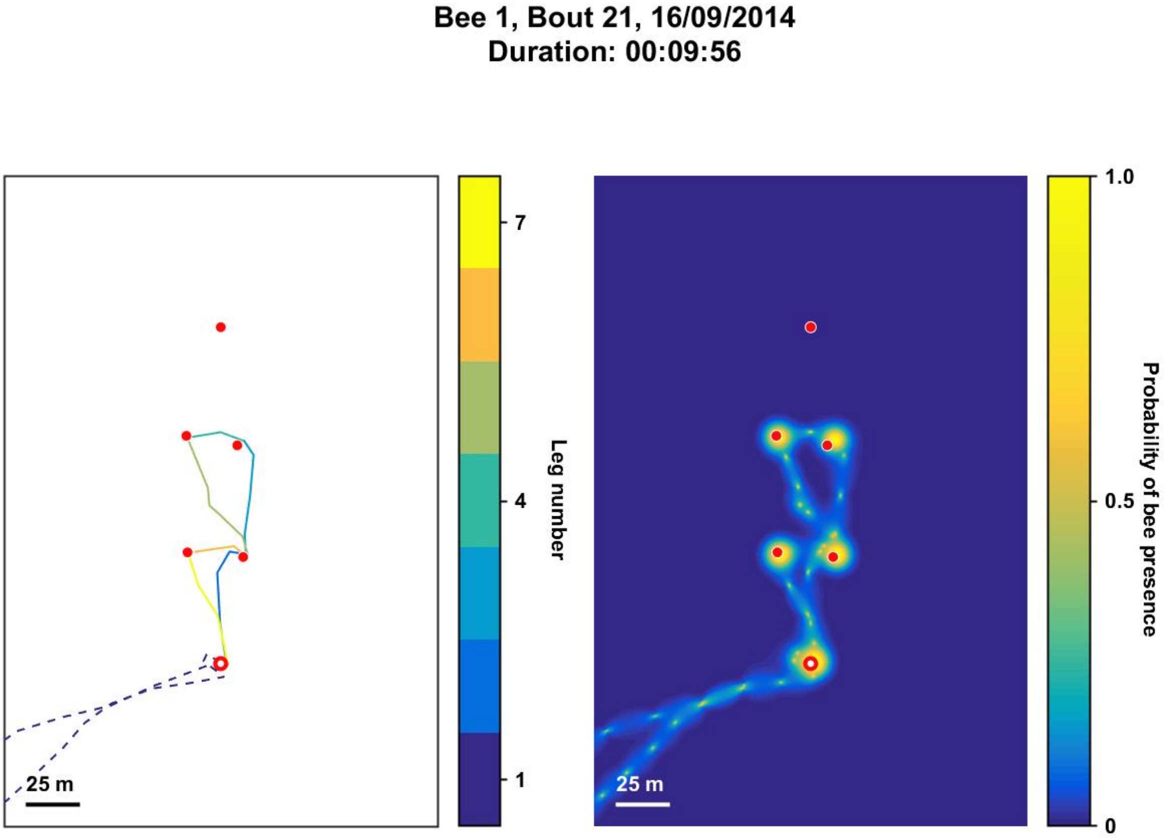

Bee 1, Bout 21, 16/09/2014
Duration: 00:09:56
25 m
7
4
1
Leg number
25 m
1.0
0.5
0
Probability of bee presence

Fig S22

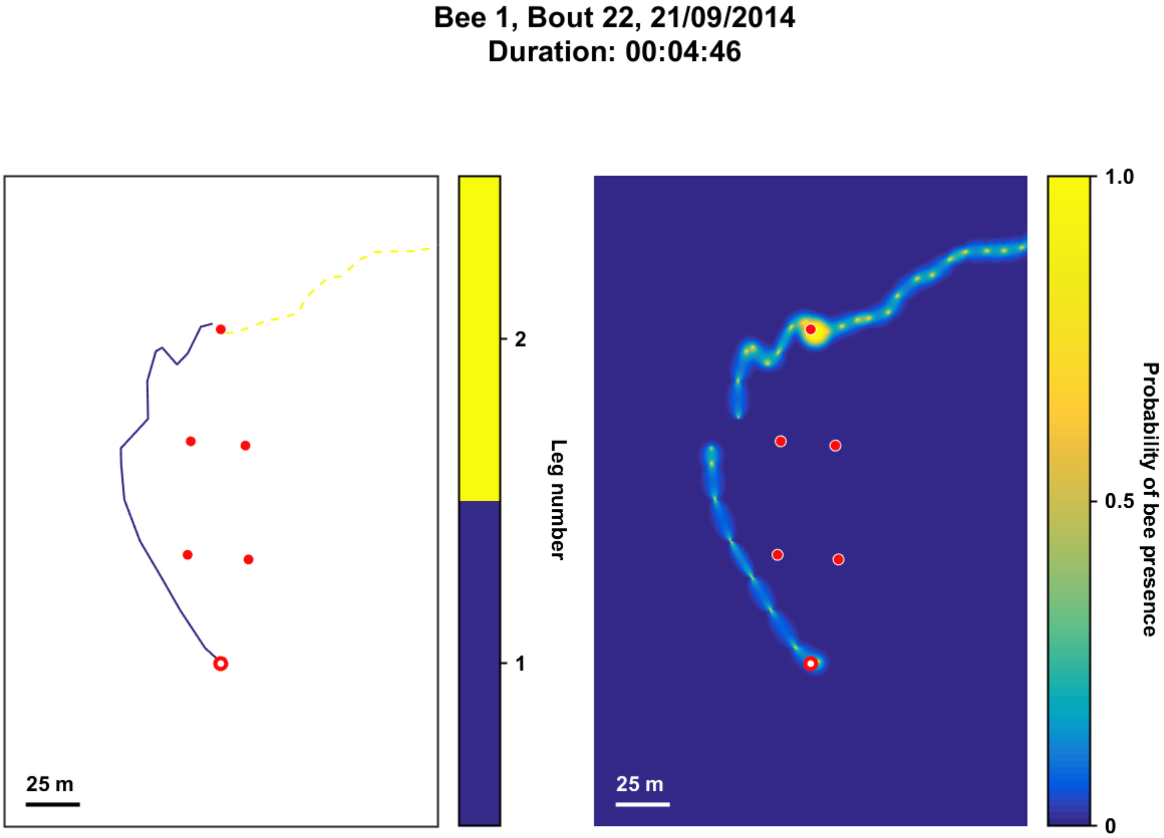

Bee 1, Bout 22, 21/09/2014
Duration: 00:04:46
25 m
2
1
Leg number
25 m
1.0
0.5
0
Probability of bee presence

Fig S23

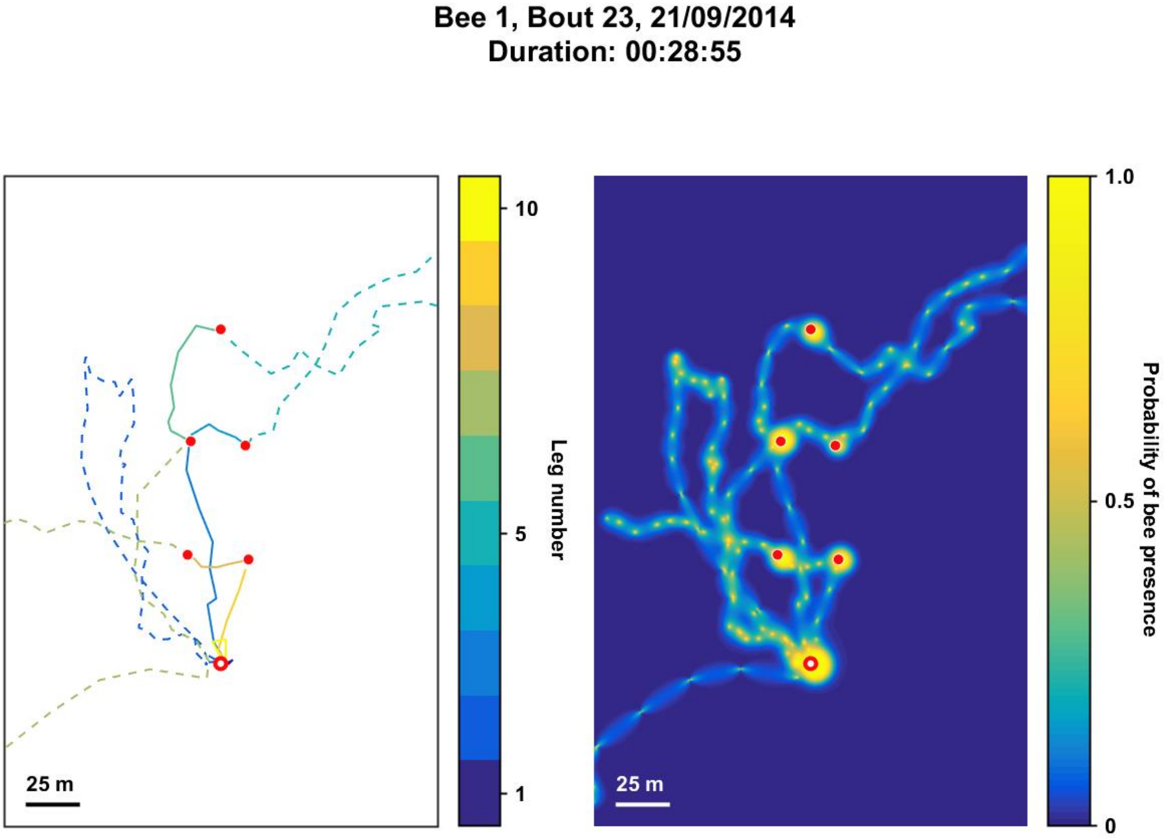
Bee 1, Bout 23, 21/09/2014
Duration: 00:28:55
25 m
10
5
1
Leg number
25 m
1.0
0.5
0
Probability of bee presence

Fig S24

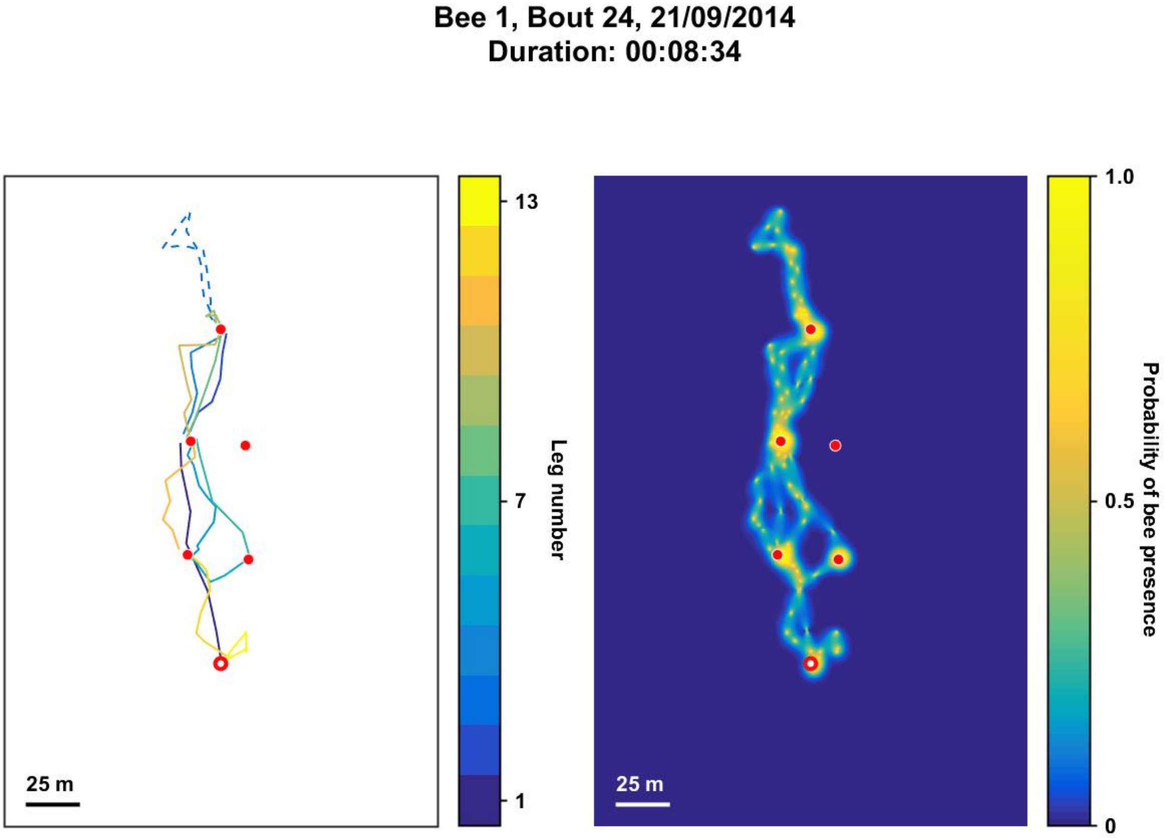

Bee 1, Bout 24, 21/09/2014
Duration: 00:08:34
25 m
13
7
1
Leg number
25 m
1.0
0.5
0
Probability of bee presence

Fig S25

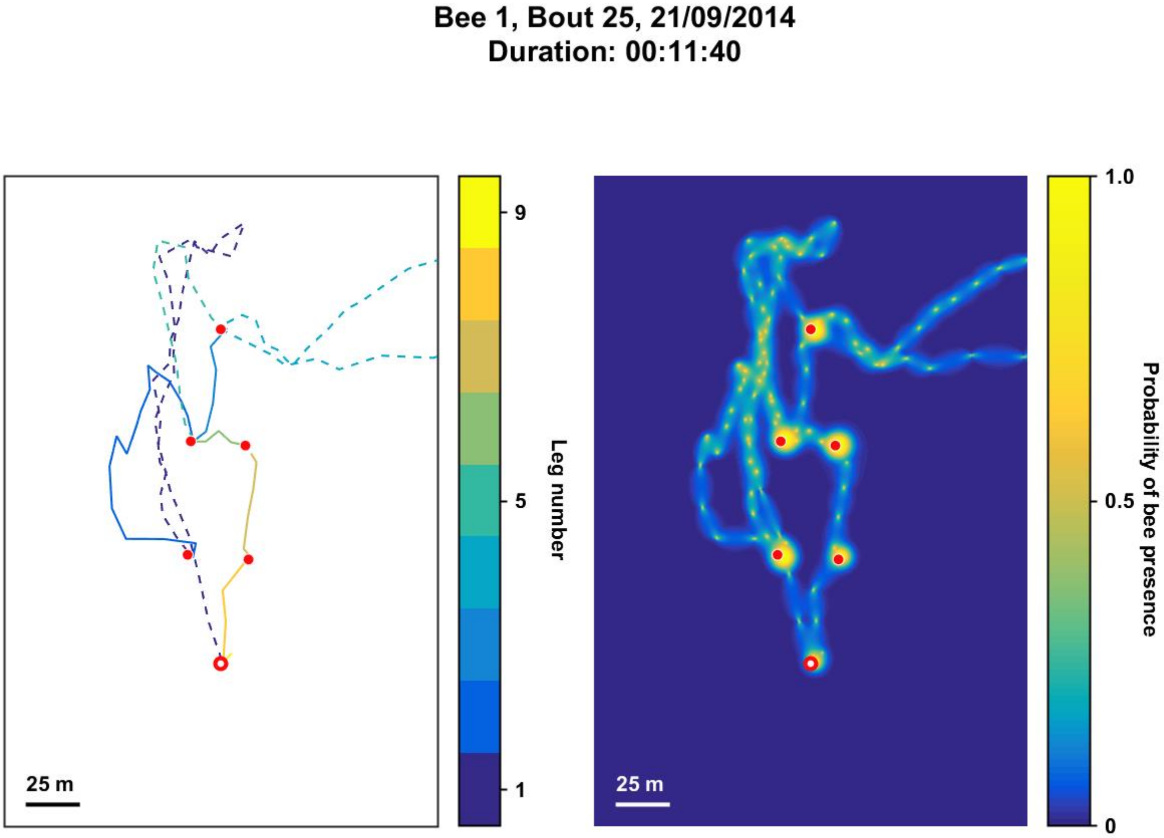

Bee 1, Bout 25, 21/09/2014
Duration: 00:11:40
25 m
Leg number
9
5
1
25 m
Probability of bee presence
1.0
0.5
0

Fig S26

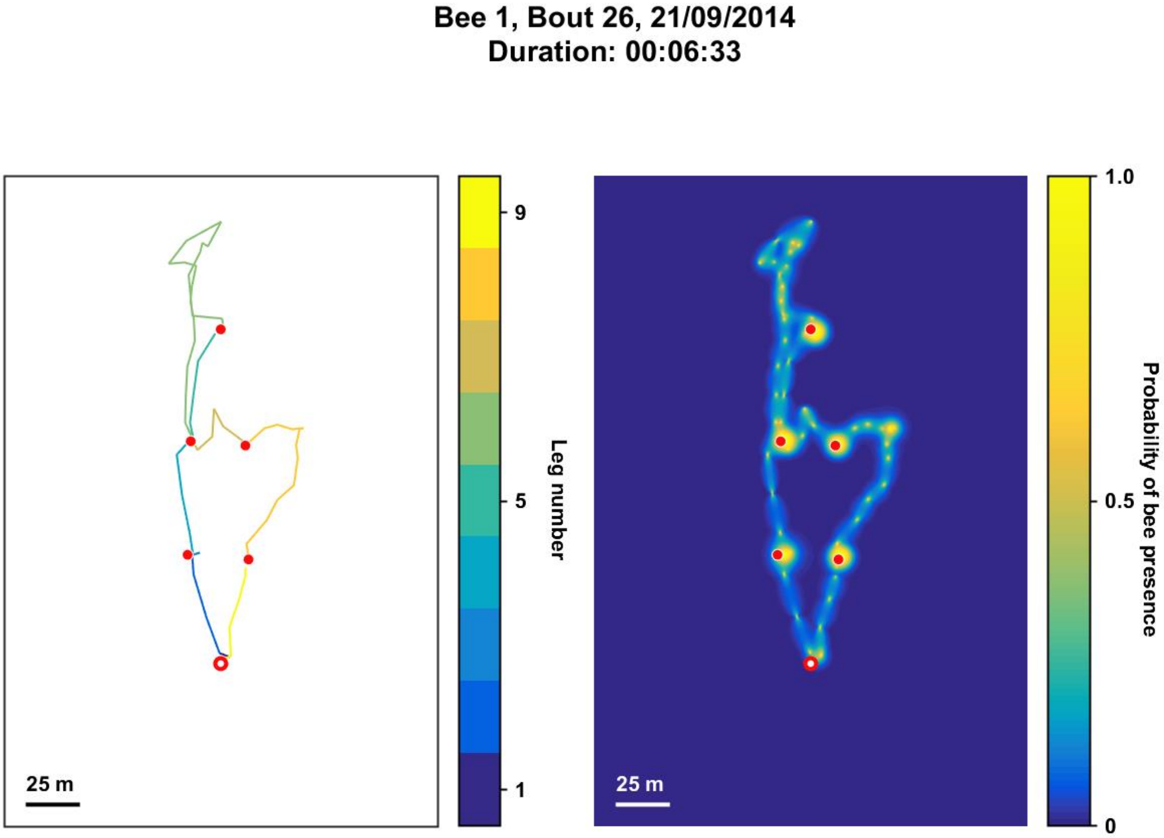

Bee 1, Bout 26, 21/09/2014
Duration: 00:06:33
25 m
Leg number
9
5
1
25 m
Probability of bee presence
1.0
0.5
0

Fig S27

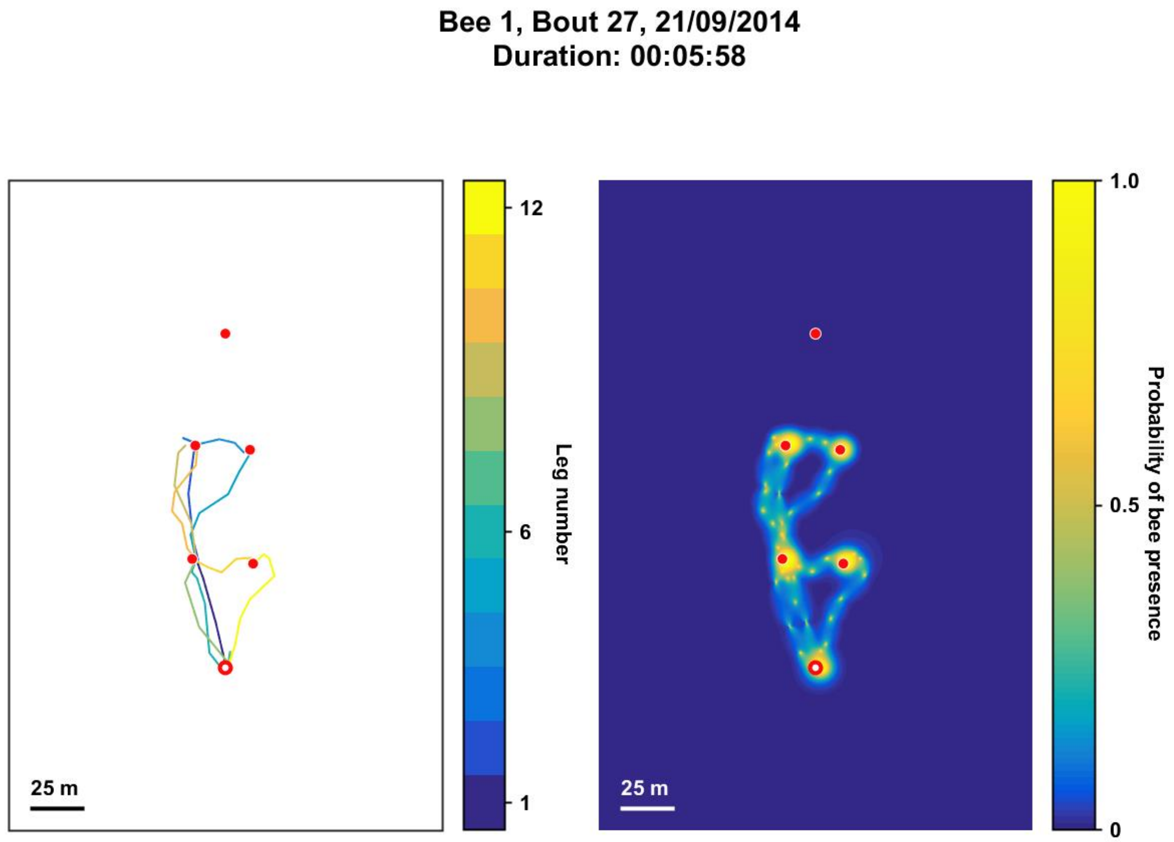
Bee 1, Bout 27, 21/09/2014
Duration: 00:05:58
25 m
12
6
1
Leg number
25 m
1.0
0.5
0
Probability of bee presence

Fig S28

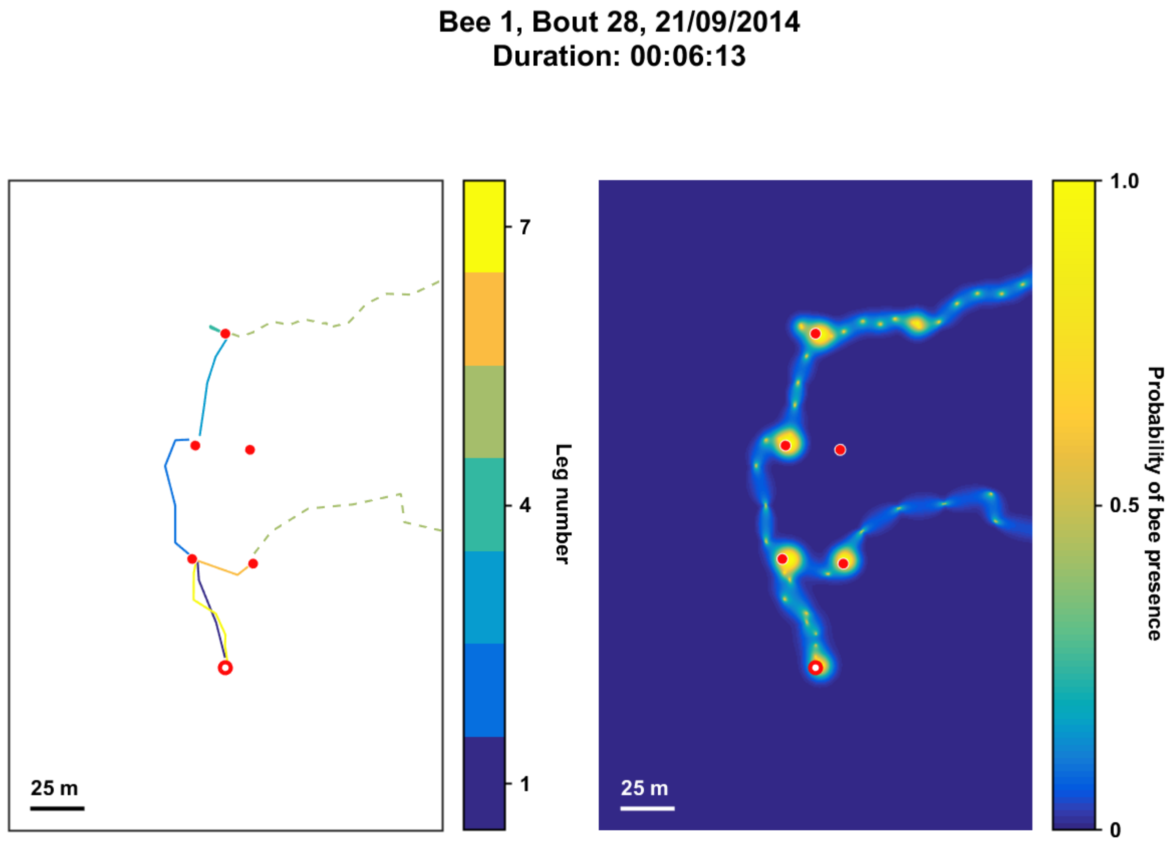
Bee 1, Bout 28, 21/09/2014
Duration: 00:06:13
25 m
7
4
1
Leg number
25 m
1.0
0.5
0
Probability of bee presence

Fig S29

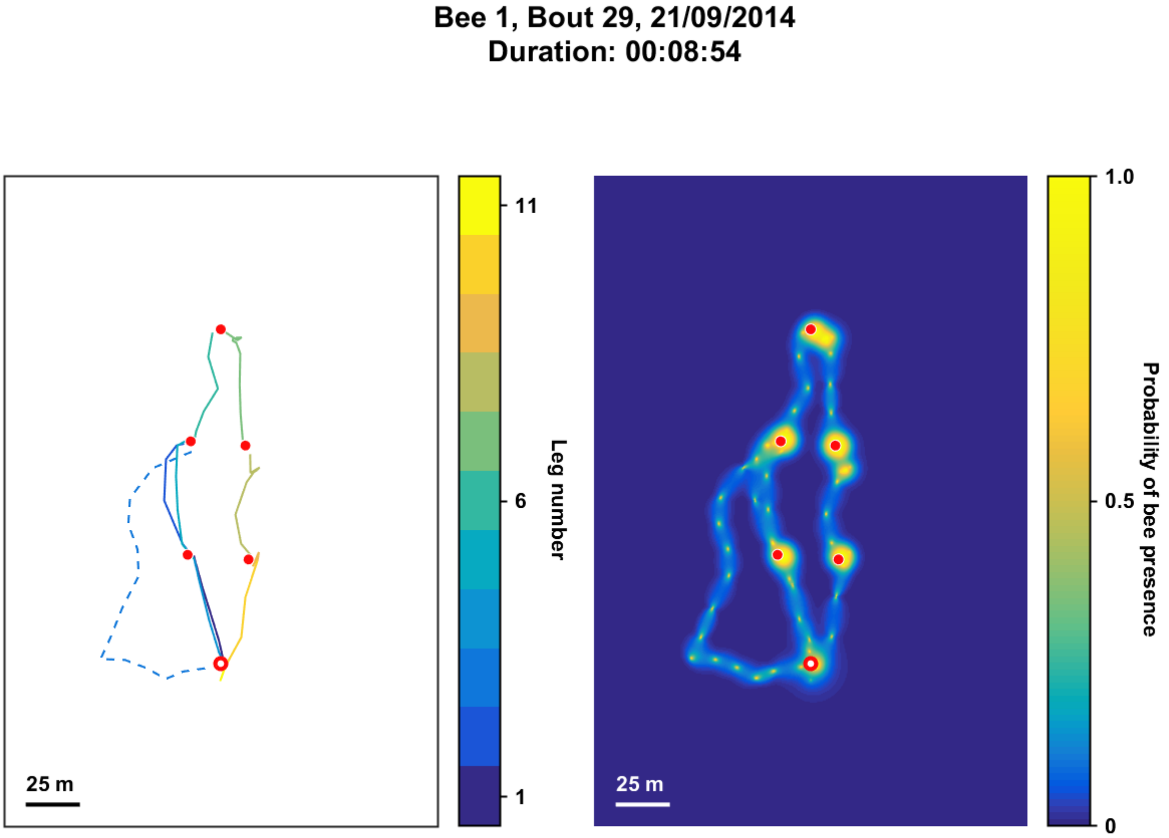

Bee 1, Bout 29, 21/09/2014
Duration: 00:08:54
25 m
11
6
1
Leg number
25 m
1.0
0.5
0
Probability of bee presence

Fig S30

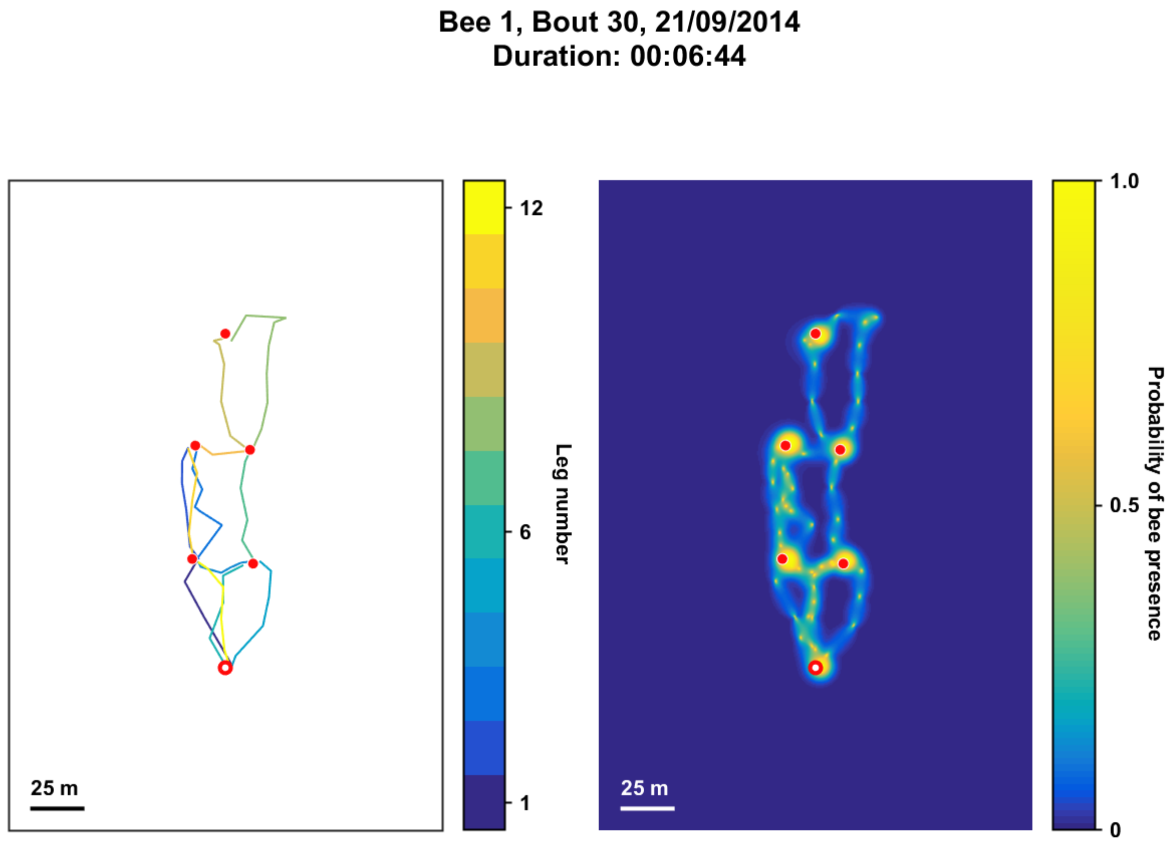

Bee 1, Bout 30, 21/09/2014
Duration: 00:06:44
25 m
12
6
1
Leg number
25 m
1.0
0.5
0
Probability of bee presence

Fig S31

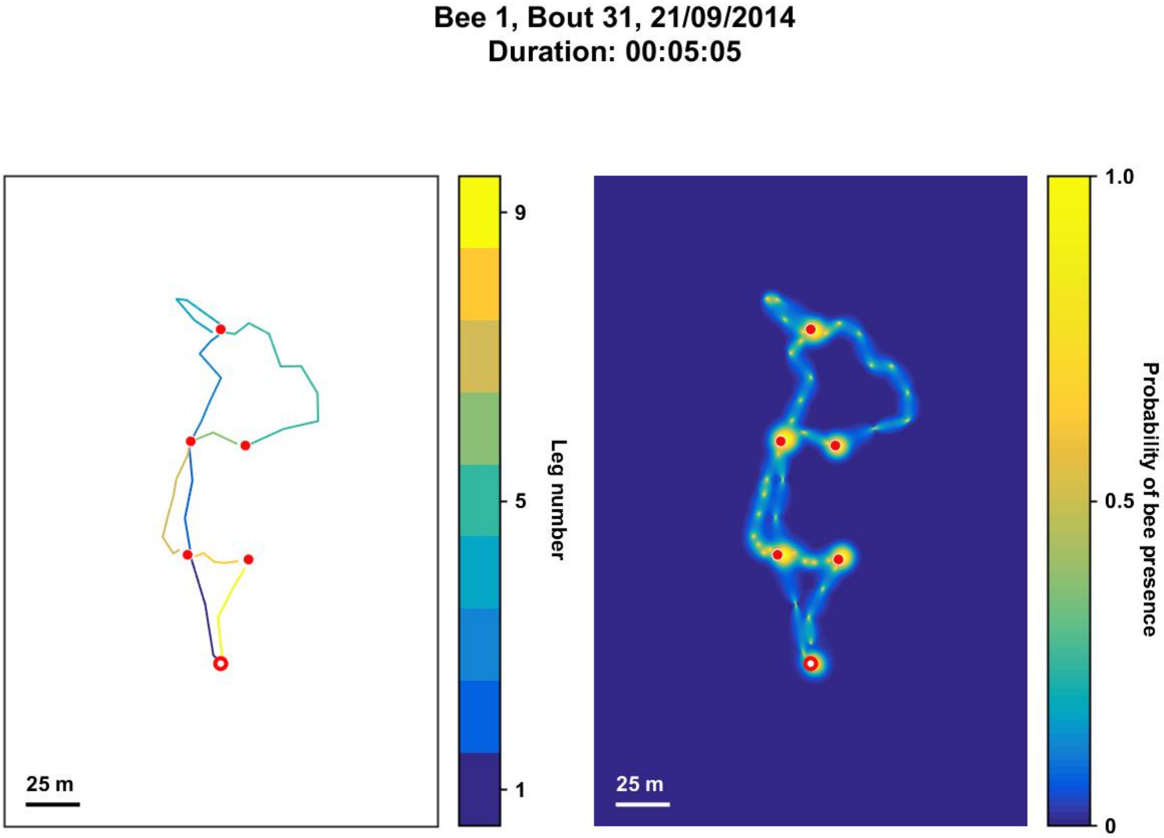

Bee 1, Bout 31, 21/09/2014
Duration: 00:05:05
25 m
9
5
1
Leg number
25 m
1.0
0.5
0
Probability of bee presence

Fig S32

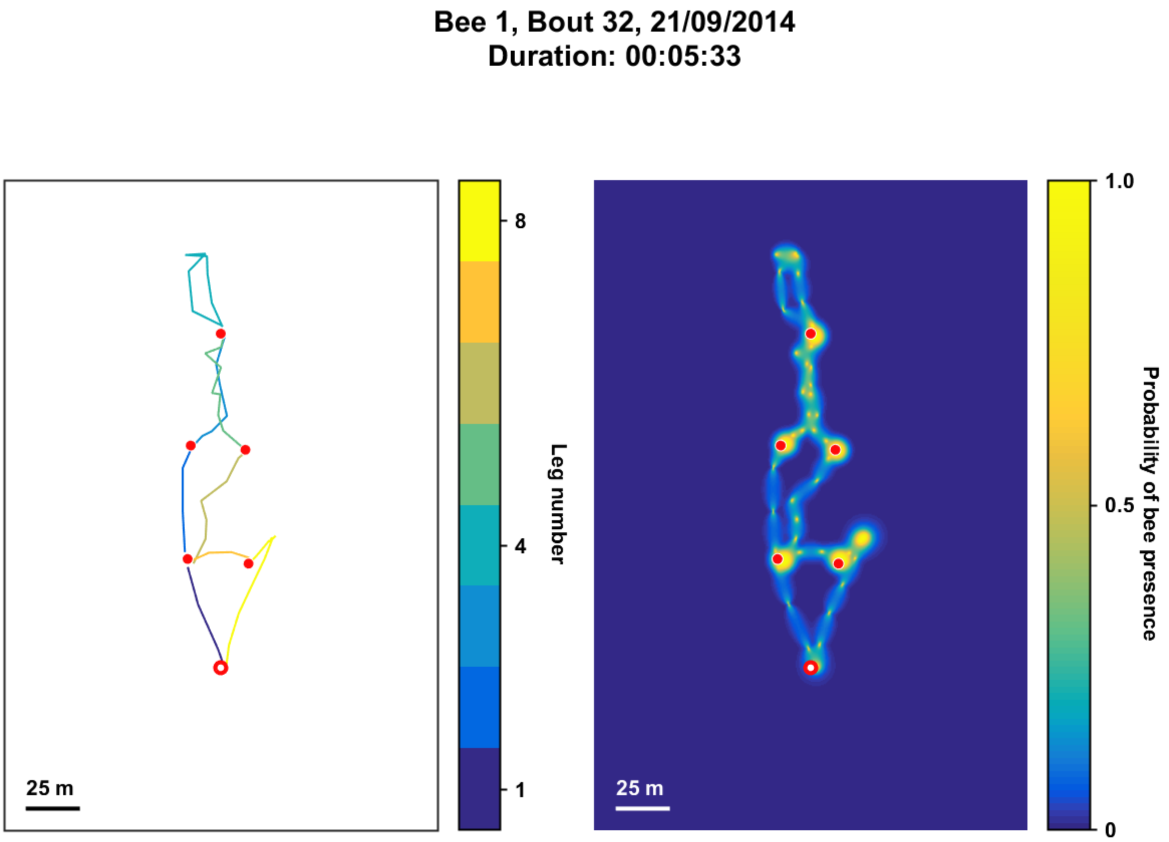

Bee 1, Bout 32, 21/09/2014
Duration: 00:05:33
25 m
Leg number
8
4
1
25 m
Probability of bee presence
1.0
0.5
0

Fig S33

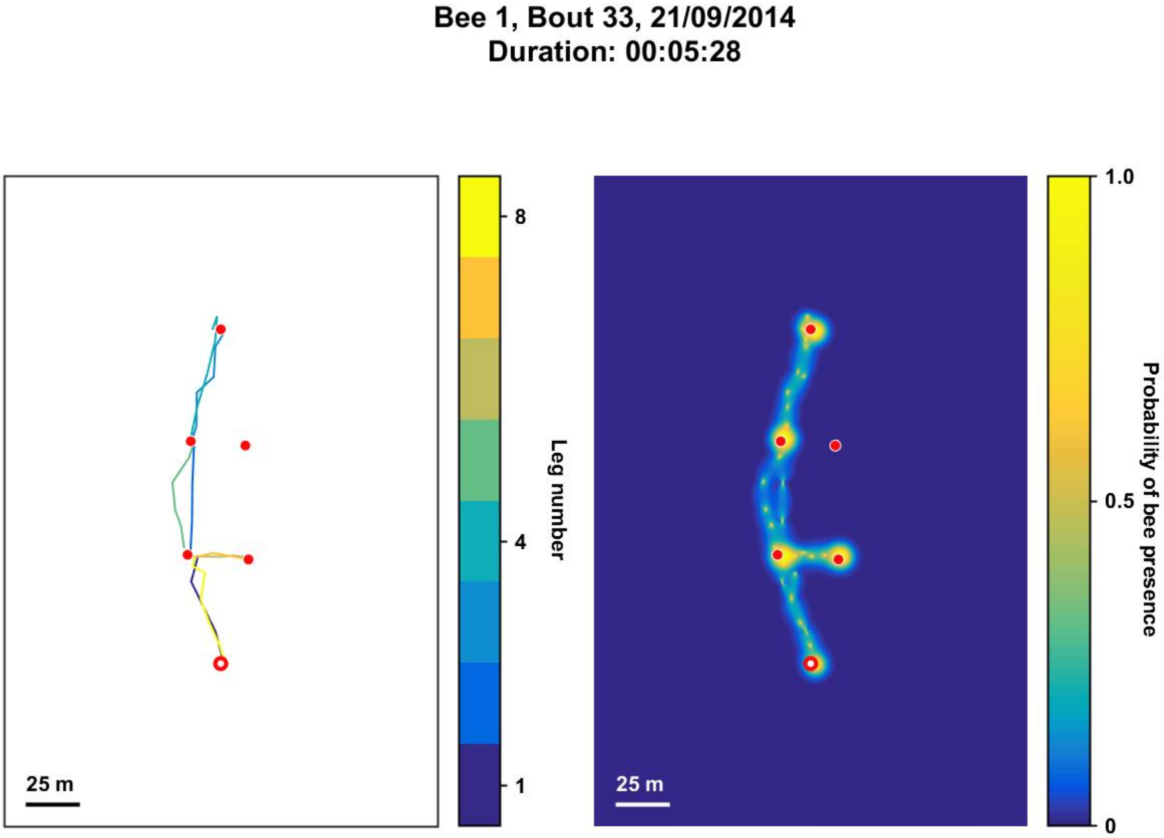

Bee 1, Bout 33, 21/09/2014
Duration: 00:05:28
25 m
Leg number
8
4
1
25 m
Probability of bee presence
1.0
0.5
0

Fig S34

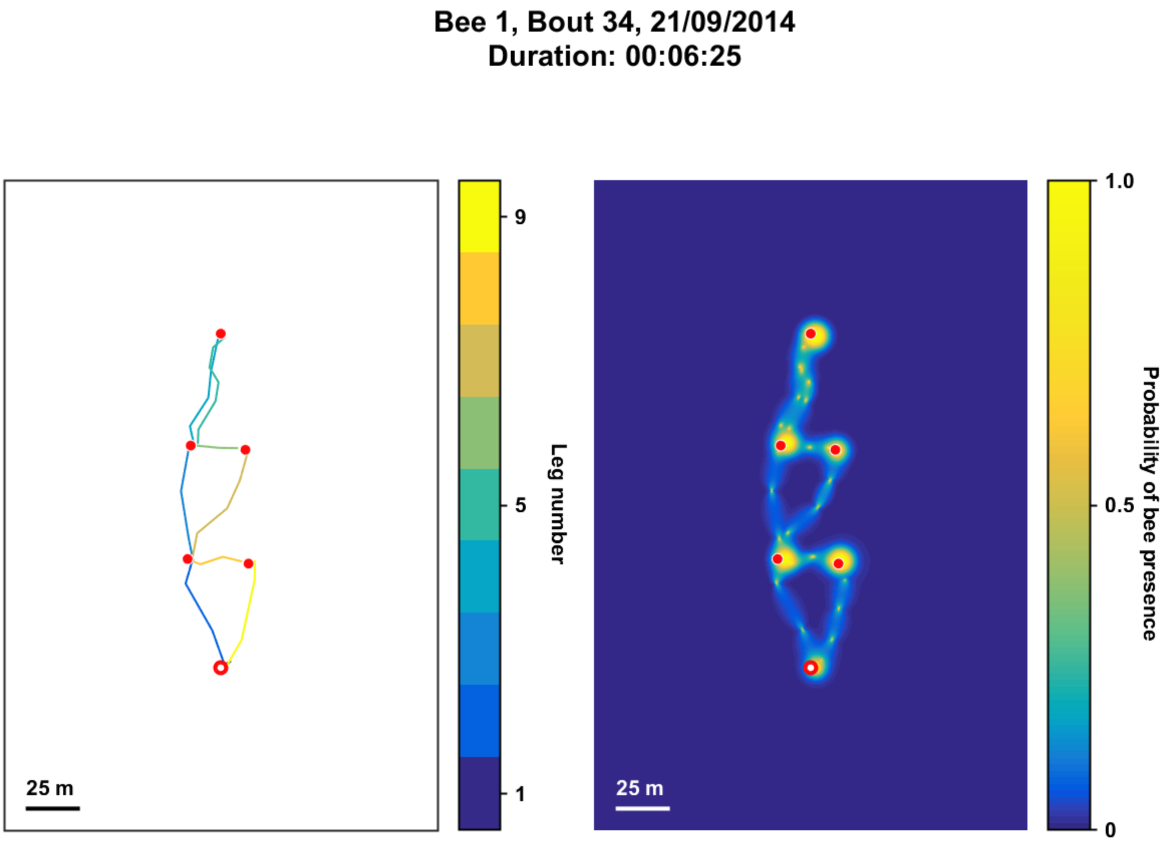
Bee 1, Bout 34, 21/09/2014
Duration: 00:06:25
25 m
9
5
1
Leg number
25 m
1.0
0.5
0
Probability of bee presence

Fig S35

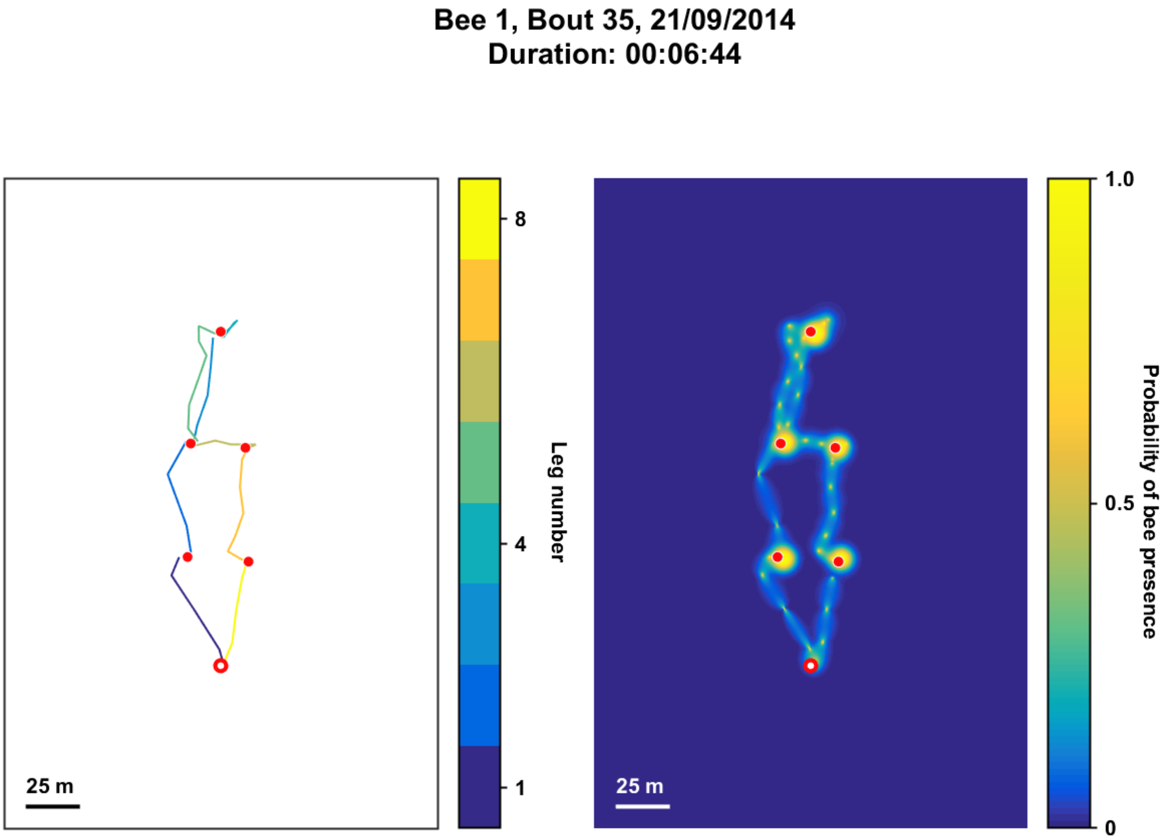
Bee 1, Bout 35, 21/09/2014
Duration: 00:06:44
25 m
8
4
1
Leg number
25 m
1.0
0.5
0
Probability of bee presence

Fig S36

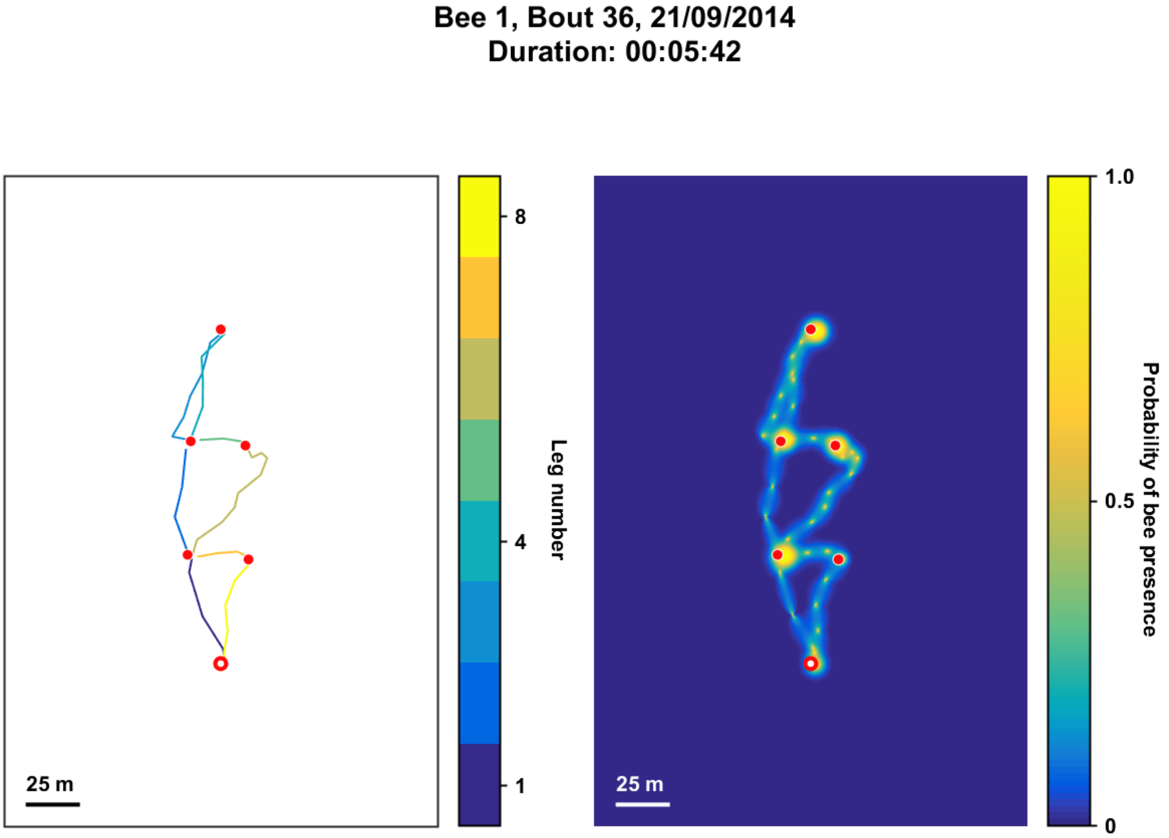

Bee 1, Bout 36, 21/09/2014
Duration: 00:05:42
25 m
8
4
1
Leg number
25 m
1.0
0.5
0
Probability of bee presence

Fig S37

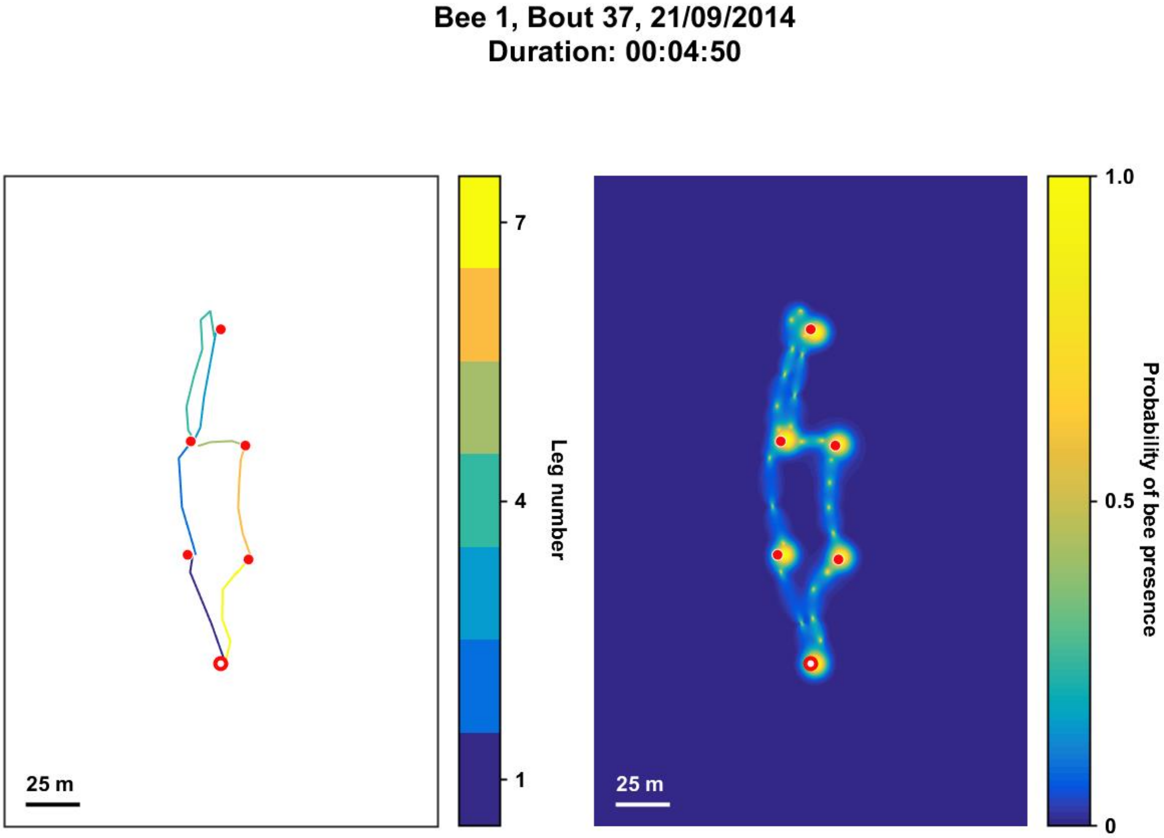

Bee 1, Bout 37, 21/09/2014
Duration: 00:04:50
25 m
7
4
1
Leg number
25 m
1.0
0.5
0
Probability of bee presence

Fig S38

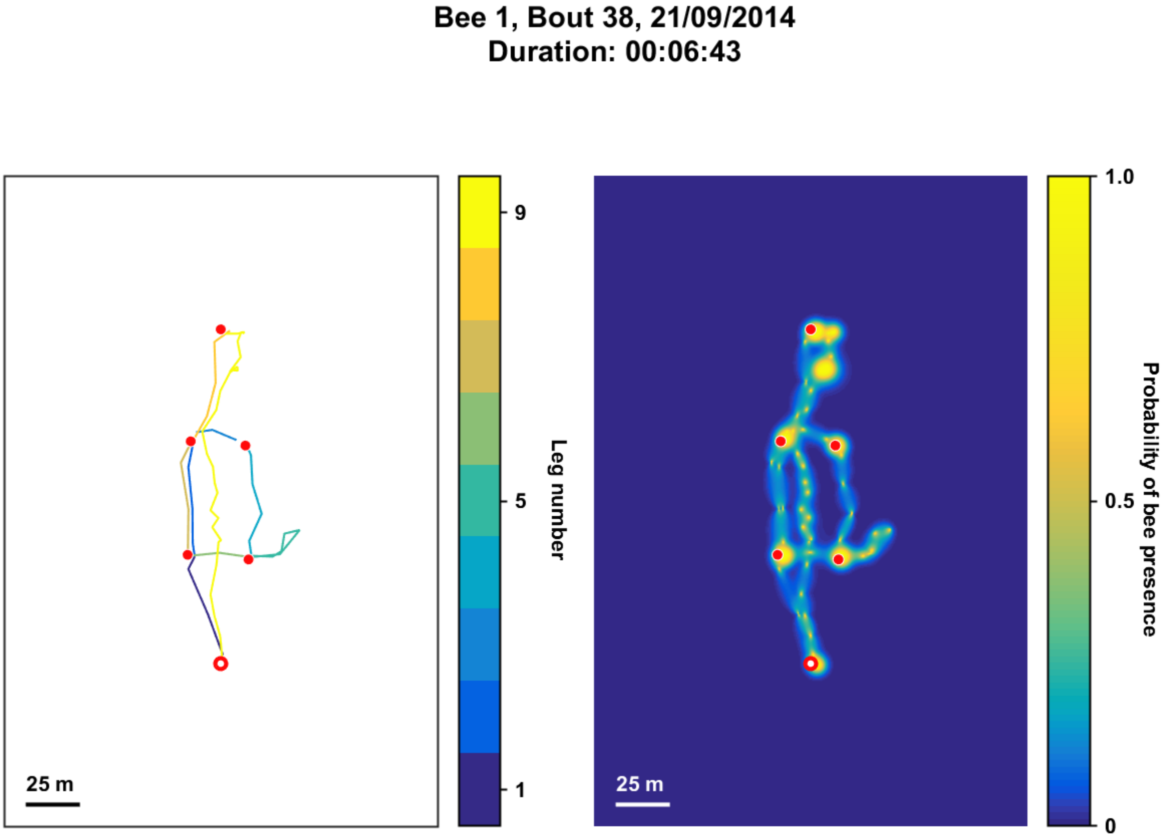
Bee 1, Bout 38, 21/09/2014
Duration: 00:06:43
25 m
Leg number
9
5
1
25 m
Probability of bee presence
1.0
0.5
0

Fig S39

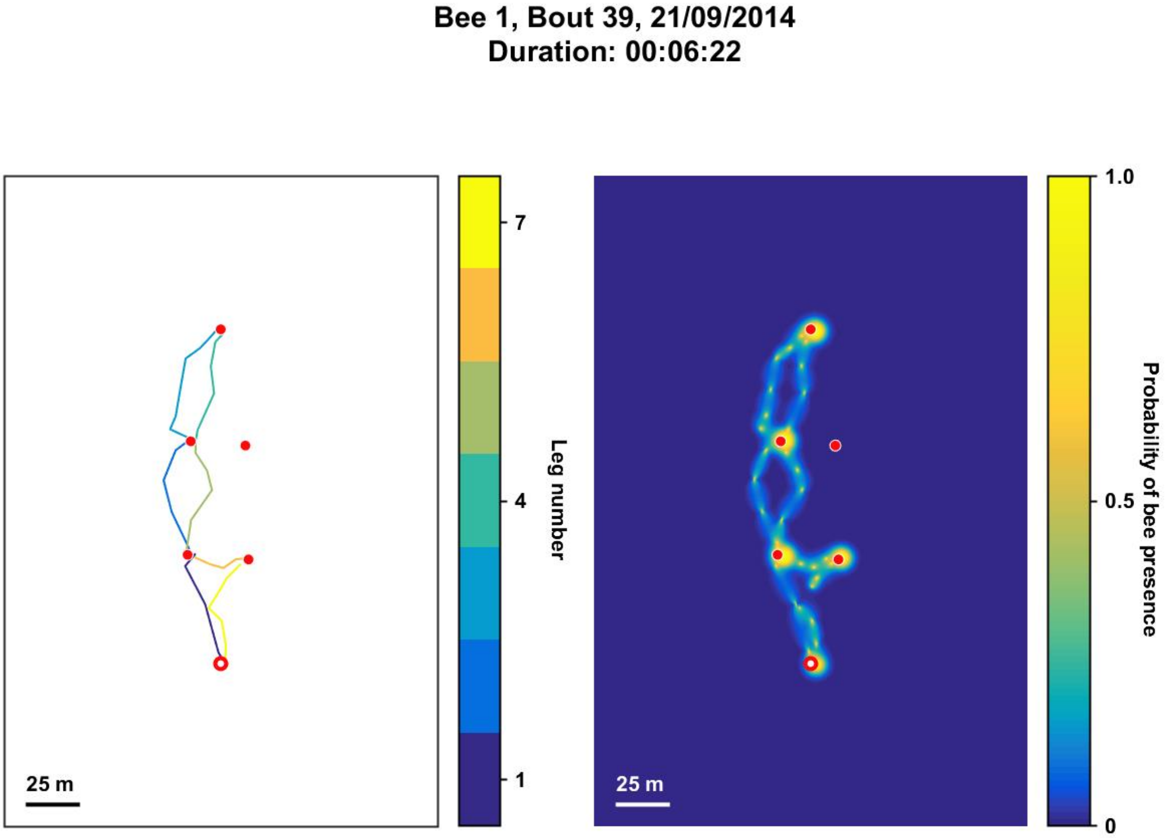

Bee 1, Bout 39, 21/09/2014
Duration: 00:06:22
25 m
Leg number
1
4
7
25 m
Probability of bee presence
0
0.5
1.0

Fig S40

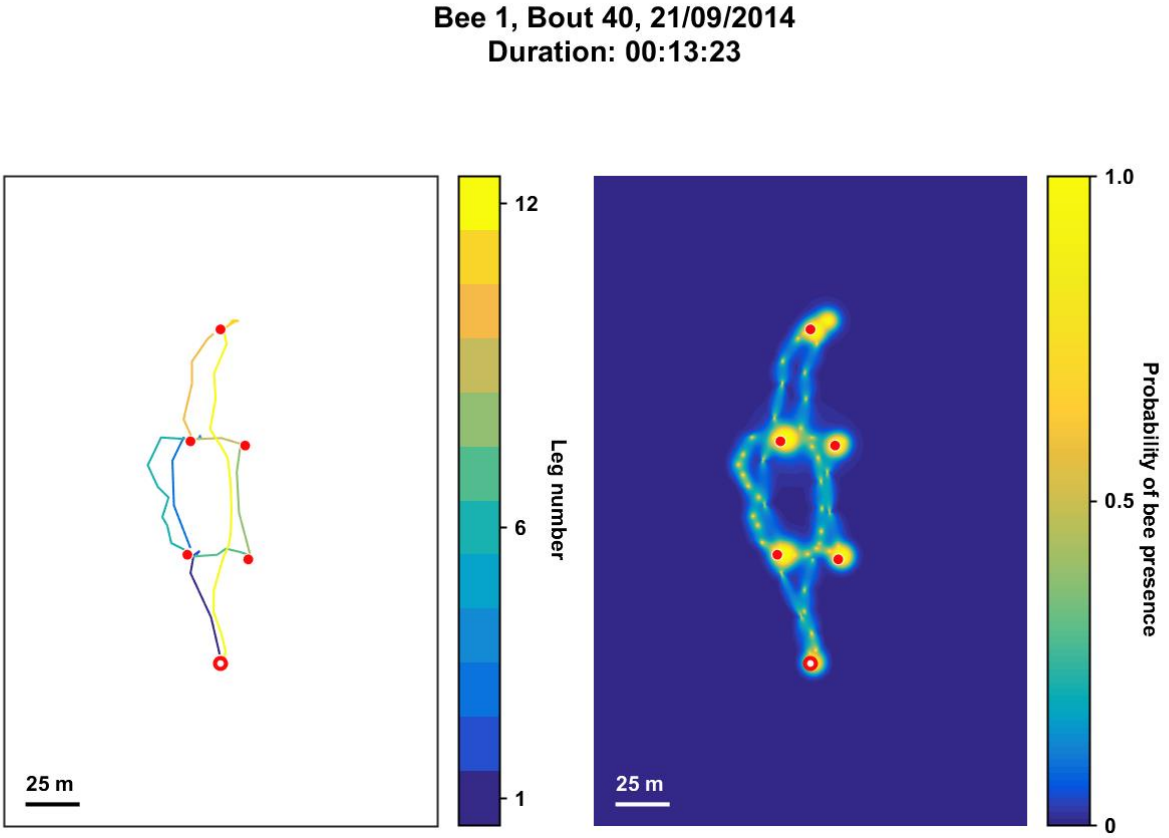

Bee 1, Bout 40, 21/09/2014
Duration: 00:13:23
25 m
12
6
1
Leg number
25 m
1.0
0.5
0
Probability of bee presence

Fig S41

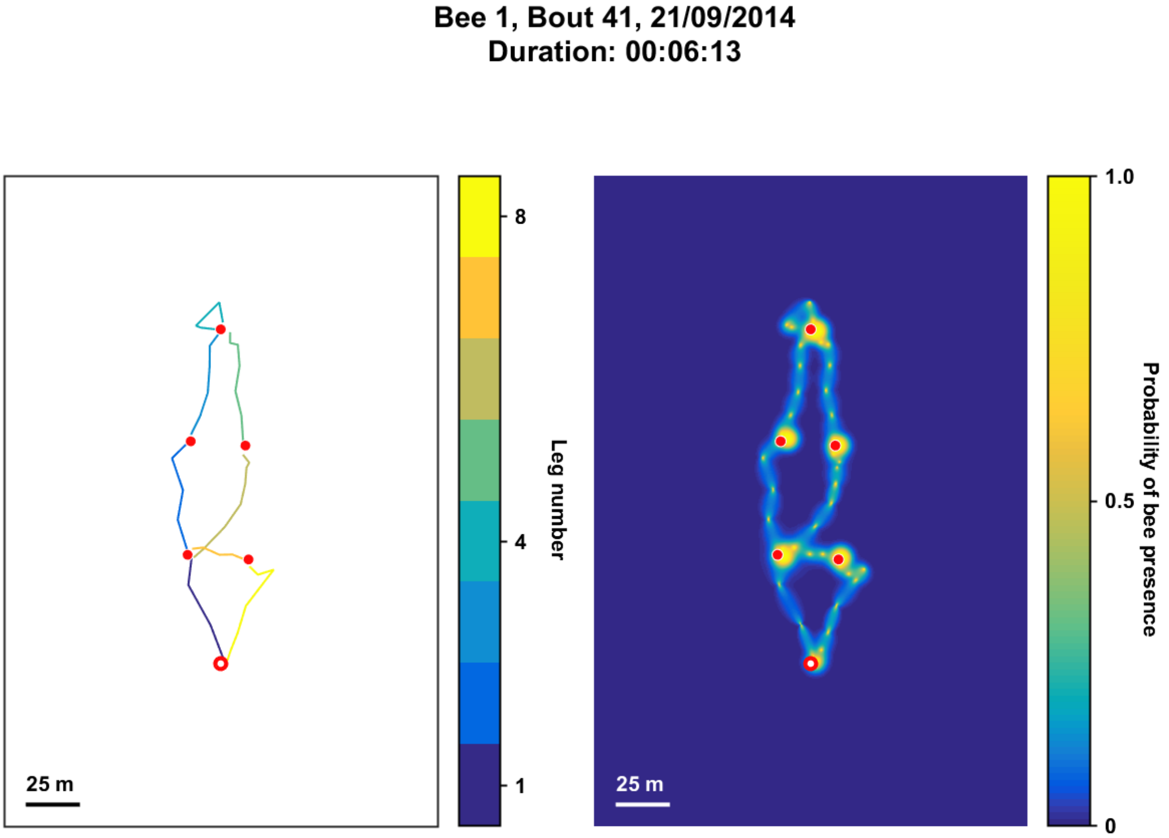

Bee 1, Bout 41, 21/09/2014
Duration: 00:06:13
25 m
8
4
1
Leg number
25 m
1.0
0.5
0
Probability of bee presence

Fig S42

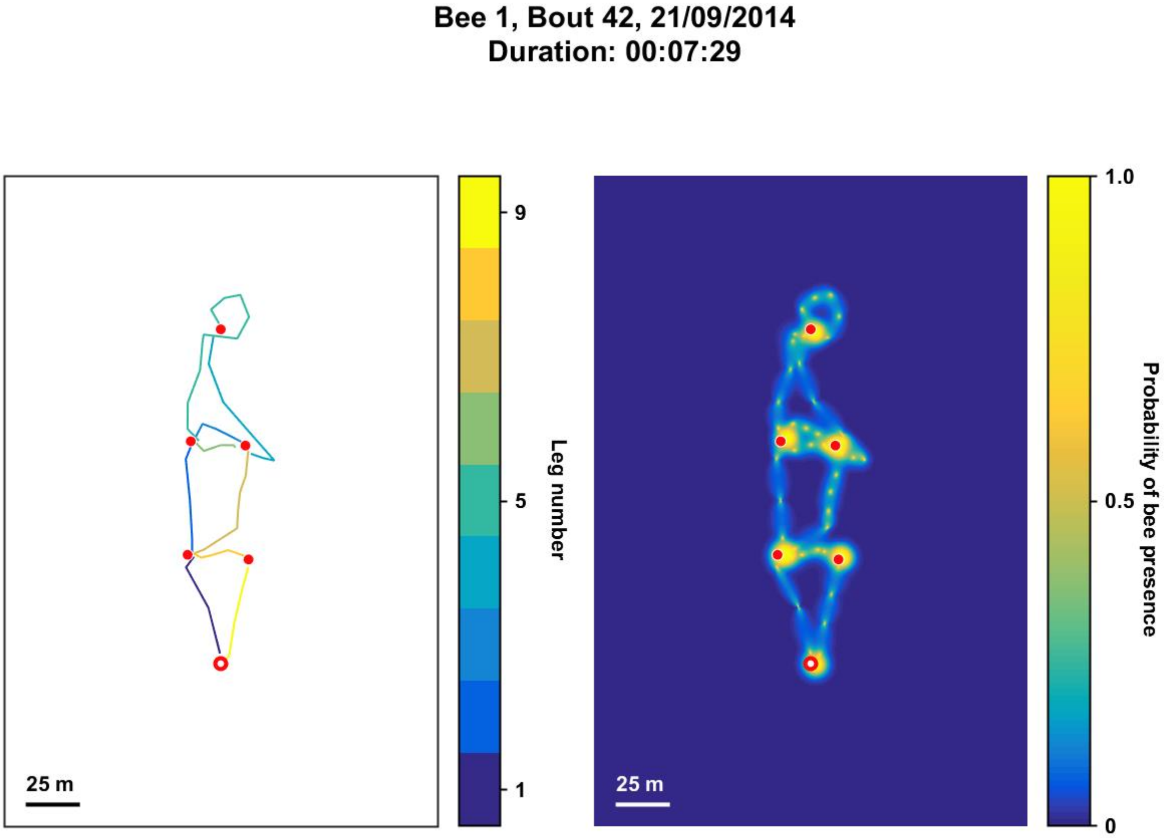

Bee 1, Bout 42, 21/09/2014
Duration: 00:07:29
25 m
Leg number
9
5
1
25 m
Probability of bee presence
1.0
0.5
0

Fig S43

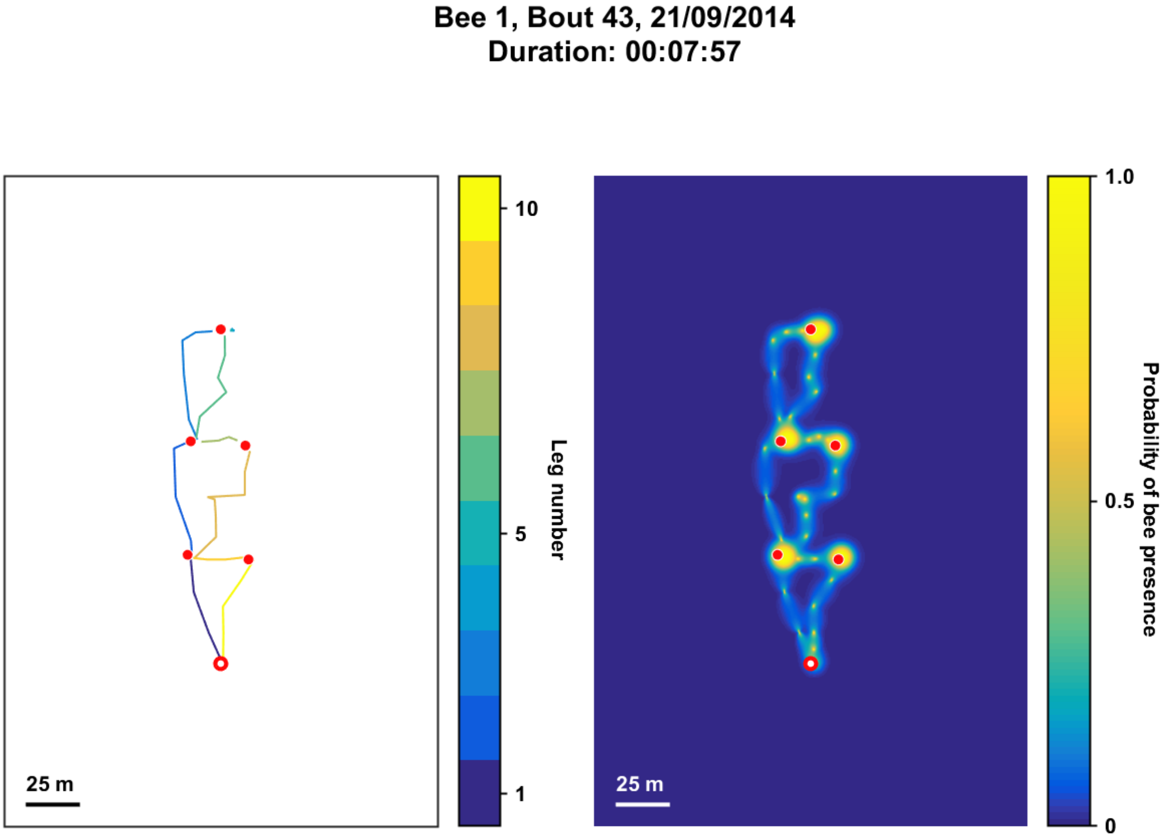

Bee 1, Bout 43, 21/09/2014
Duration: 00:07:57
25 m
10
5
1
Leg number
25 m
1.0
0.5
0
Probability of bee presence

Fig S44

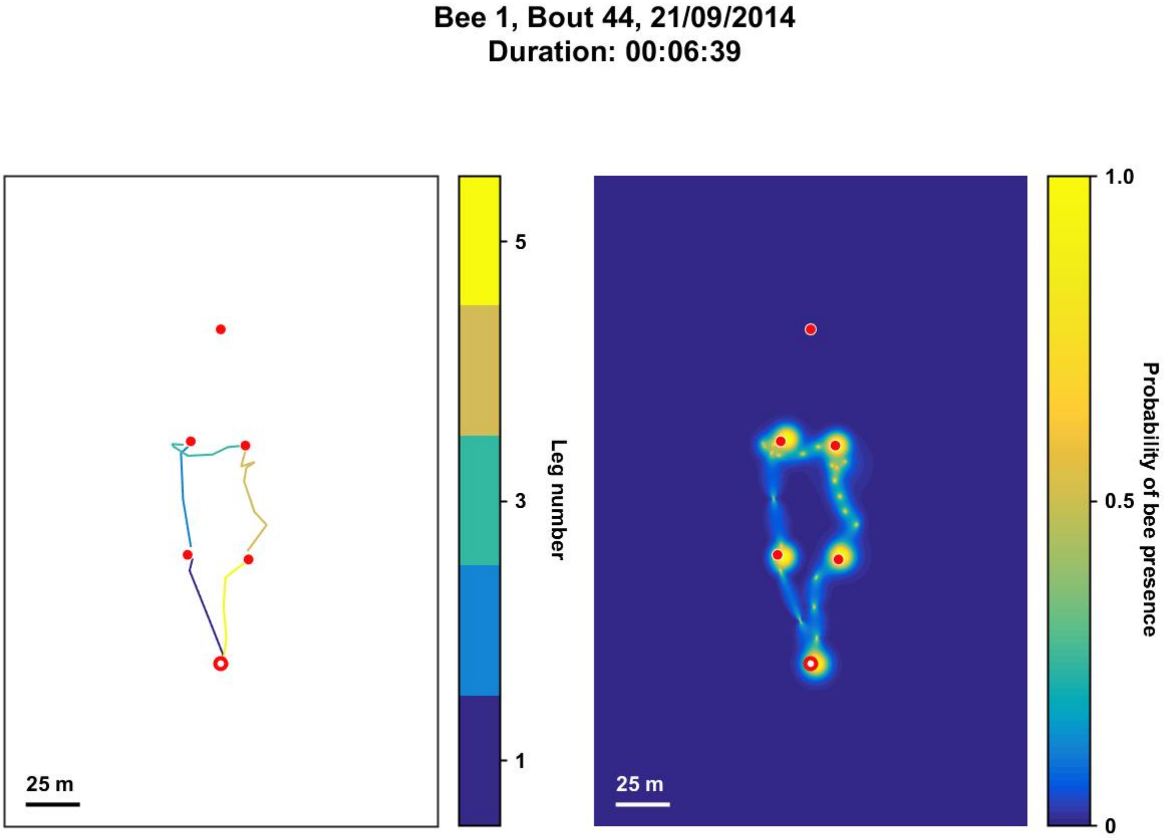

Bee 1, Bout 44, 21/09/2014
Duration: 00:06:39
25 m
Leg number
1
3
5
25 m
Probability of bee presence
0
0.5
1.0

Fig S45

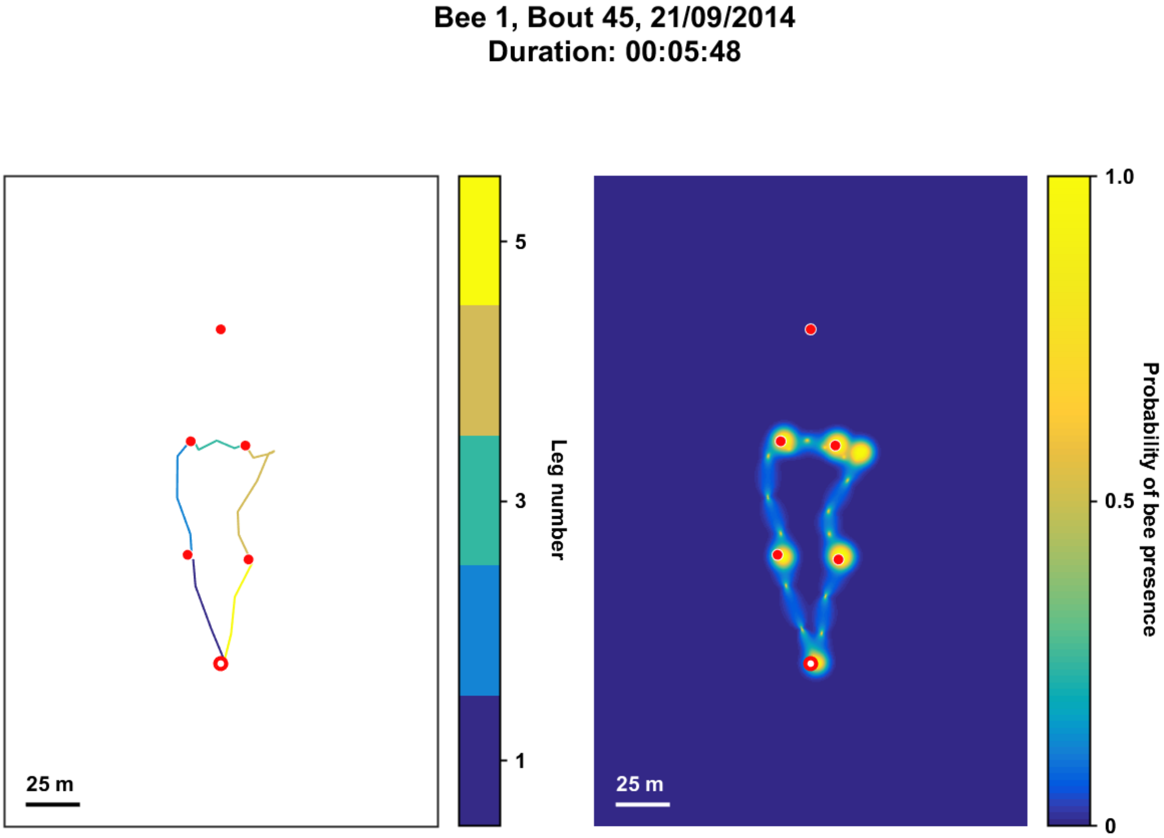

Bee 1, Bout 45, 21/09/2014
Duration: 00:05:48
25 m
5
3
1
Leg number
25 m
1.0
0.5
0
Probability of bee presence

Fig S46

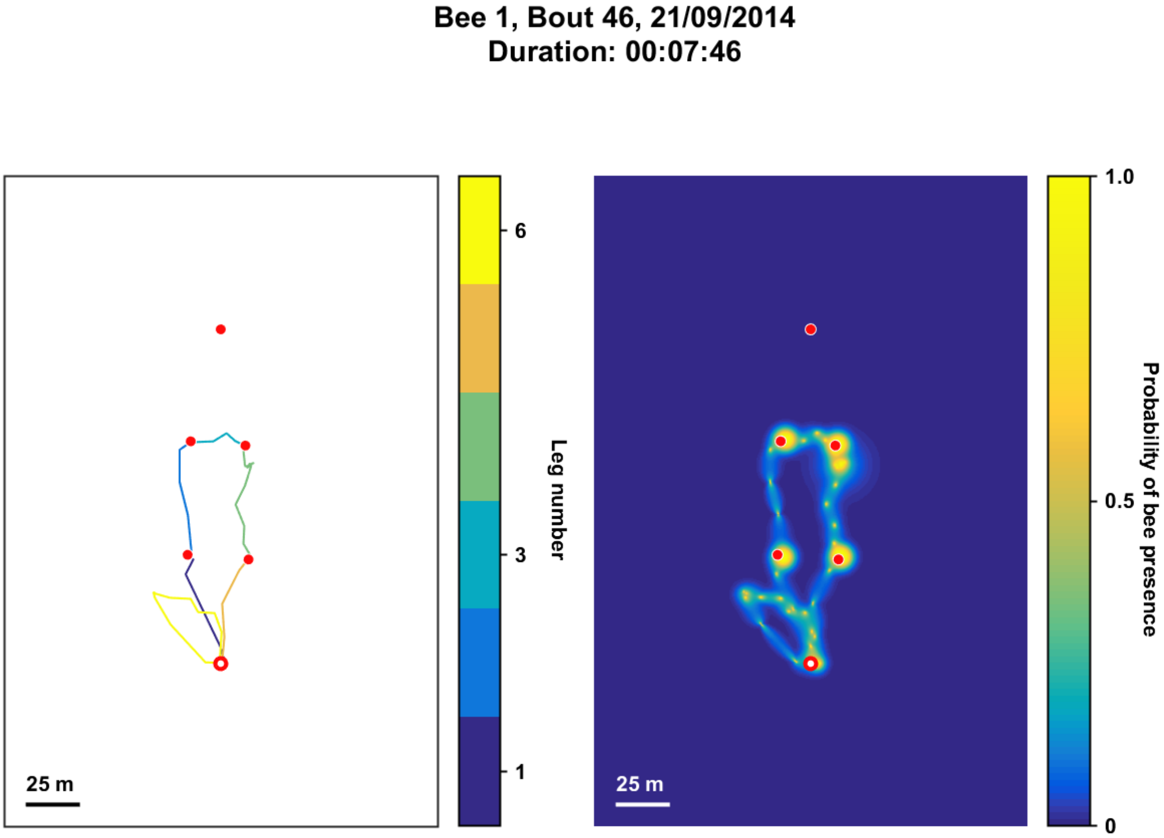

Bee 1, Bout 46, 21/09/2014
Duration: 00:07:46
25 m
6
3
1
Leg number
25 m
1.0
0.5
0
Probability of bee presence

Fig S47

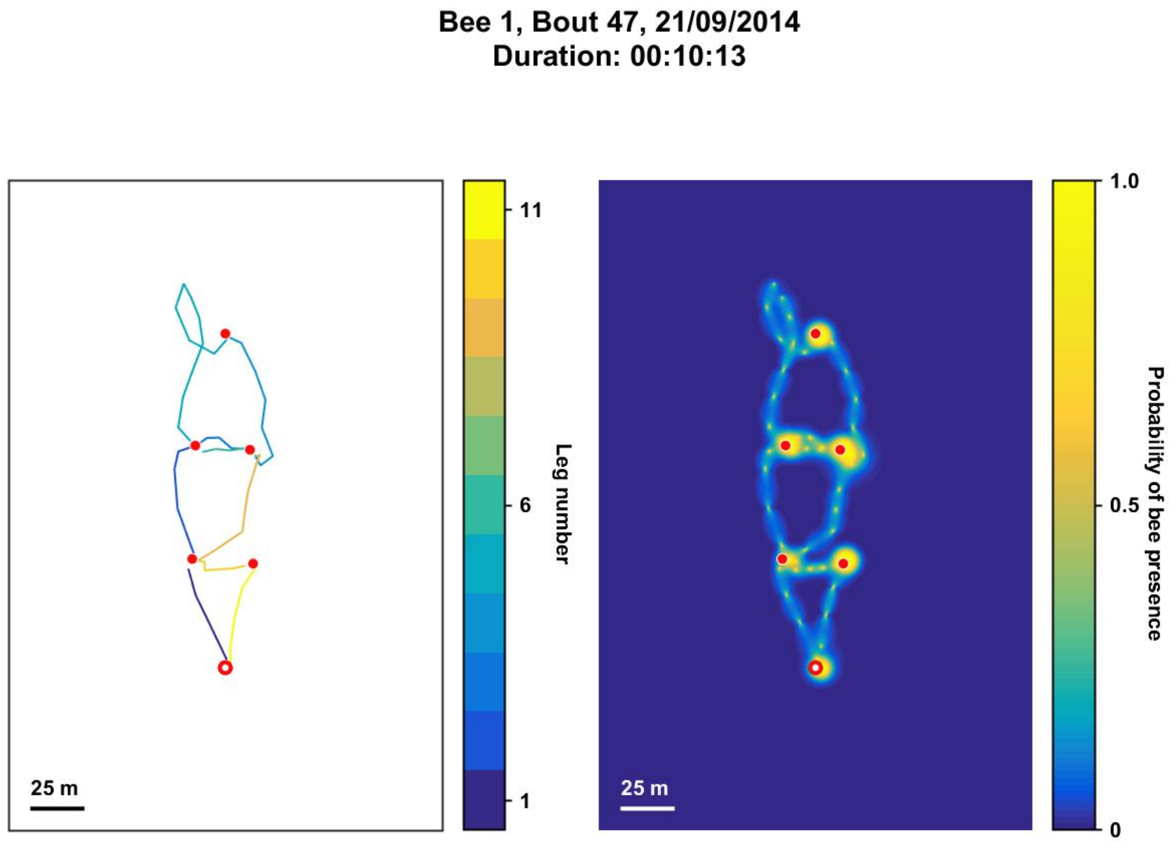

Bee 1, Bout 47, 21/09/2014
Duration: 00:10:13
25 m
Leg number
11
6
1
25 m
Probability of bee presence
1.0
0.5
0

Fig S48

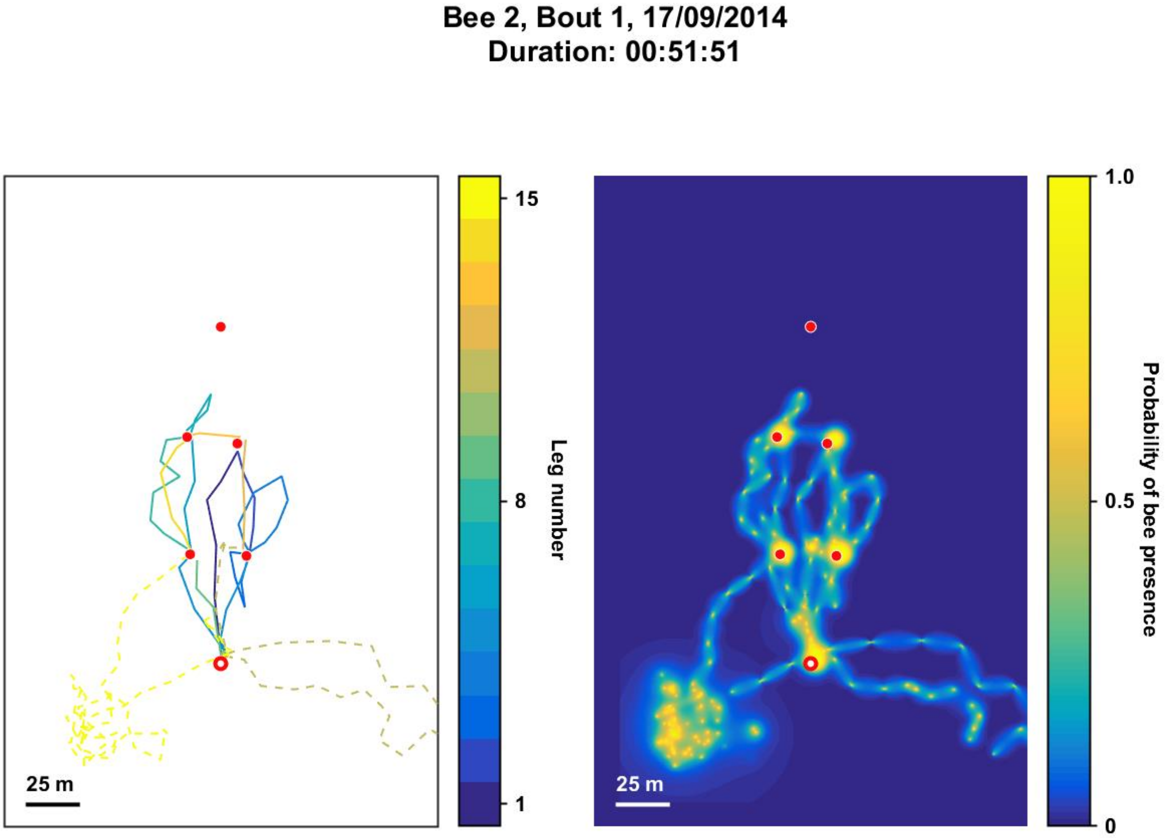

Bee 2, Bout 1, 17/09/2014
Duration: 00:51:51
25 m
15
8
1
Leg number
25 m
1.0
0.5
0
Probability of bee presence

Fig S49

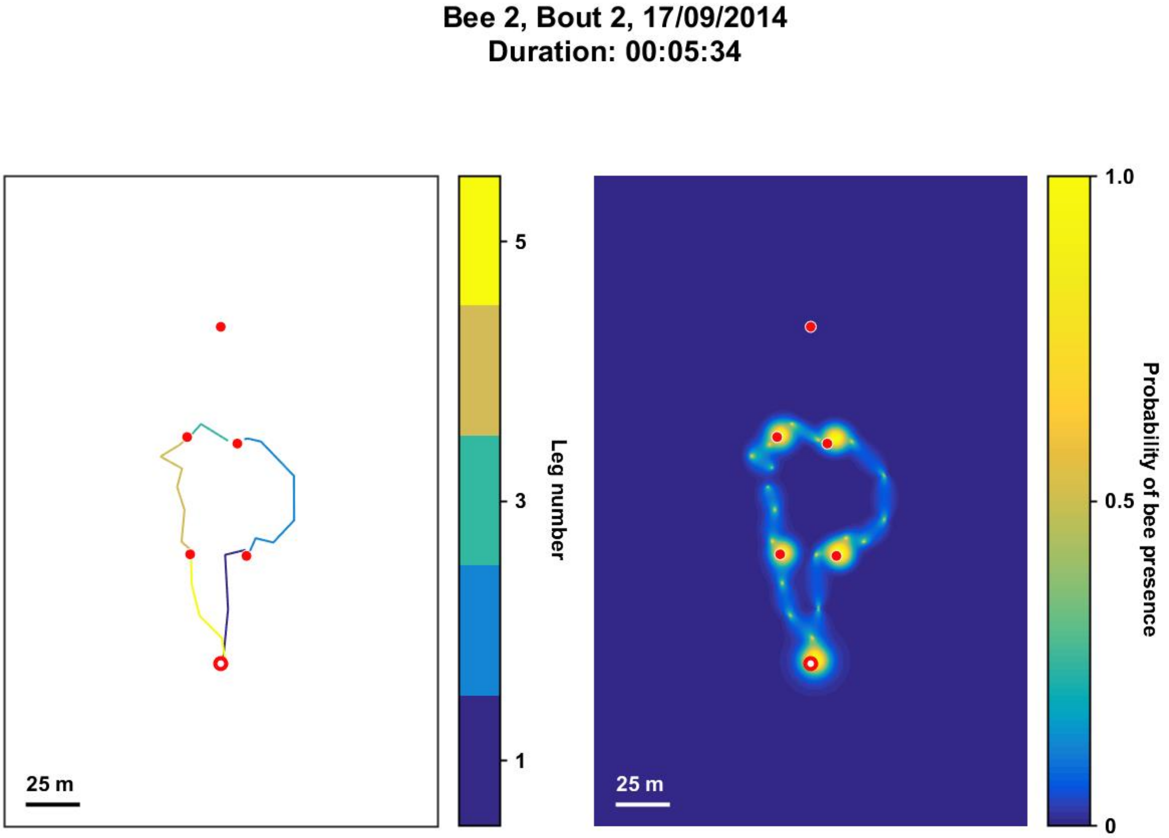

Bee 2, Bout 2, 17/09/2014
Duration: 00:05:34
25 m
5
3
1
Leg number
25 m
1.0
0.5
0
Probability of bee presence

Fig S50

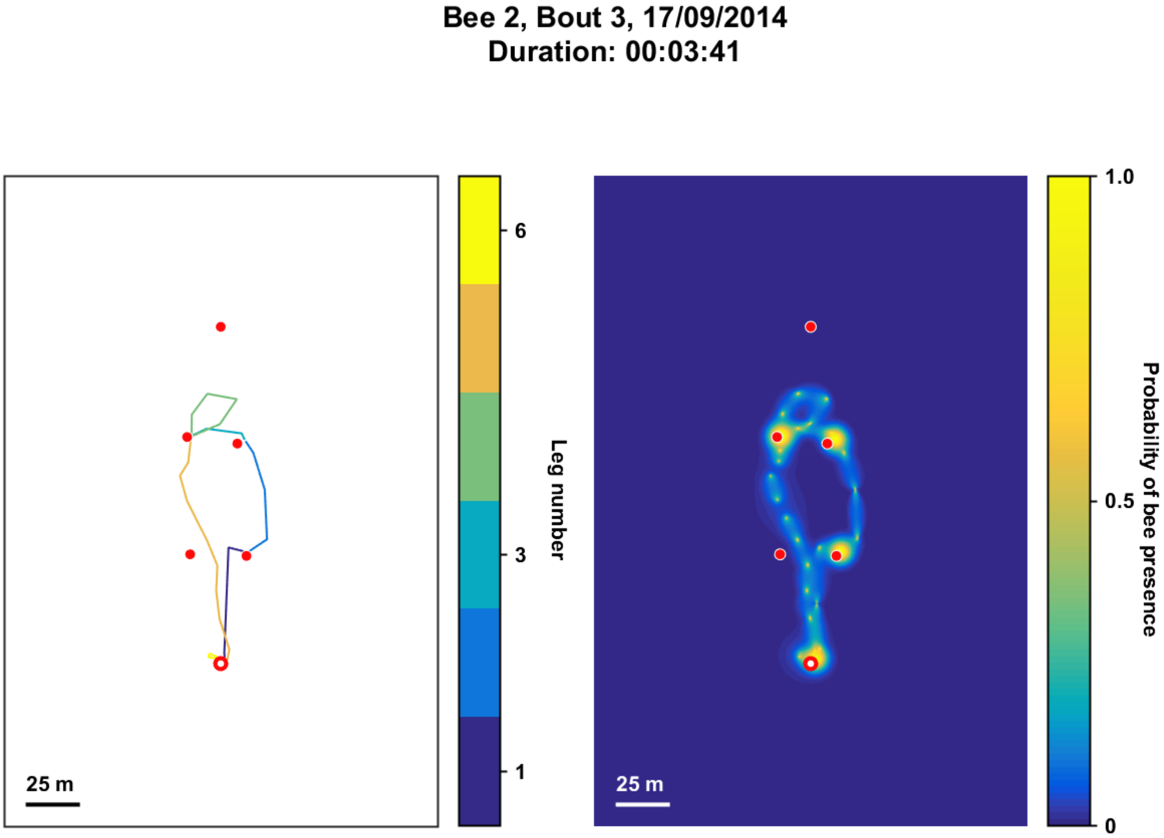
Bee 2, Bout 3, 17/09/2014
Duration: 00:03:41
25 m
6
3
1
Leg number
25 m
1.0
0.5
0
Probability of bee presence

Fig S51

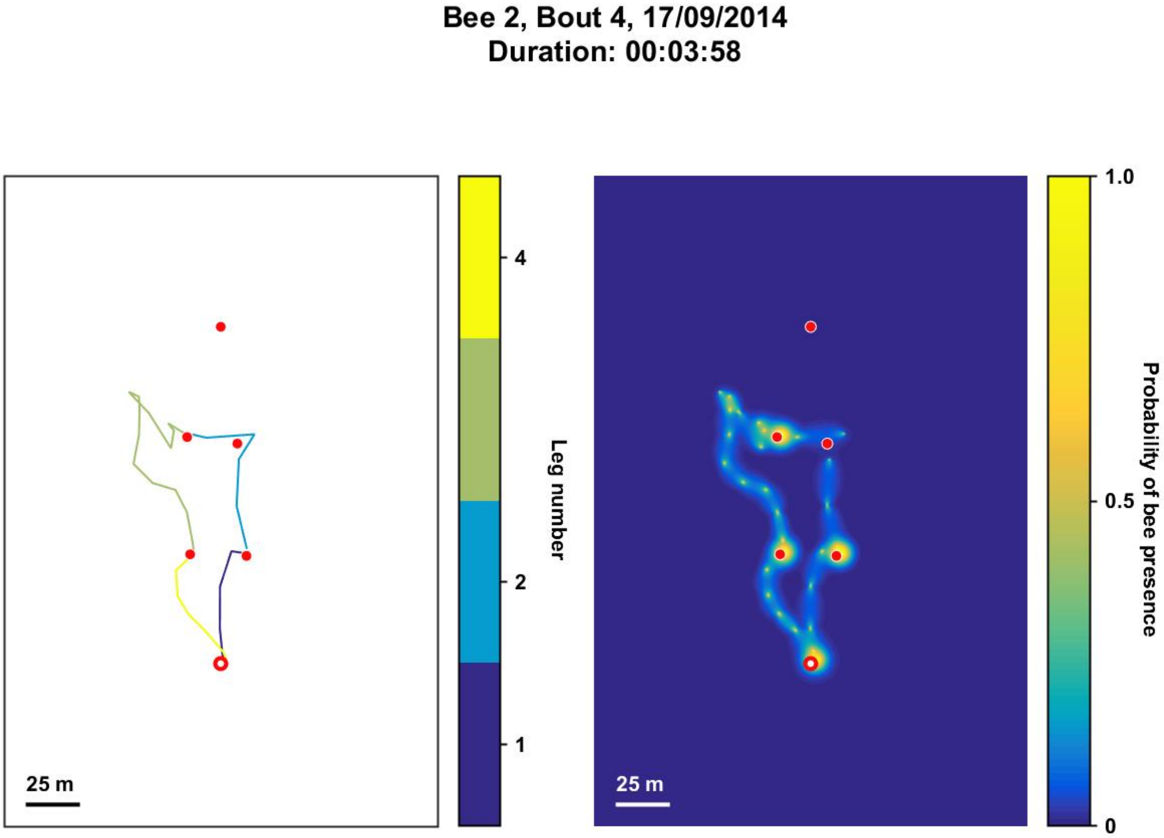
Bee 2, Bout 4, 17/09/2014
Duration: 00:03:58
25 m
4
2
1
Leg number
25 m
1.0
0.5
0
Probability of bee presence

Fig S52

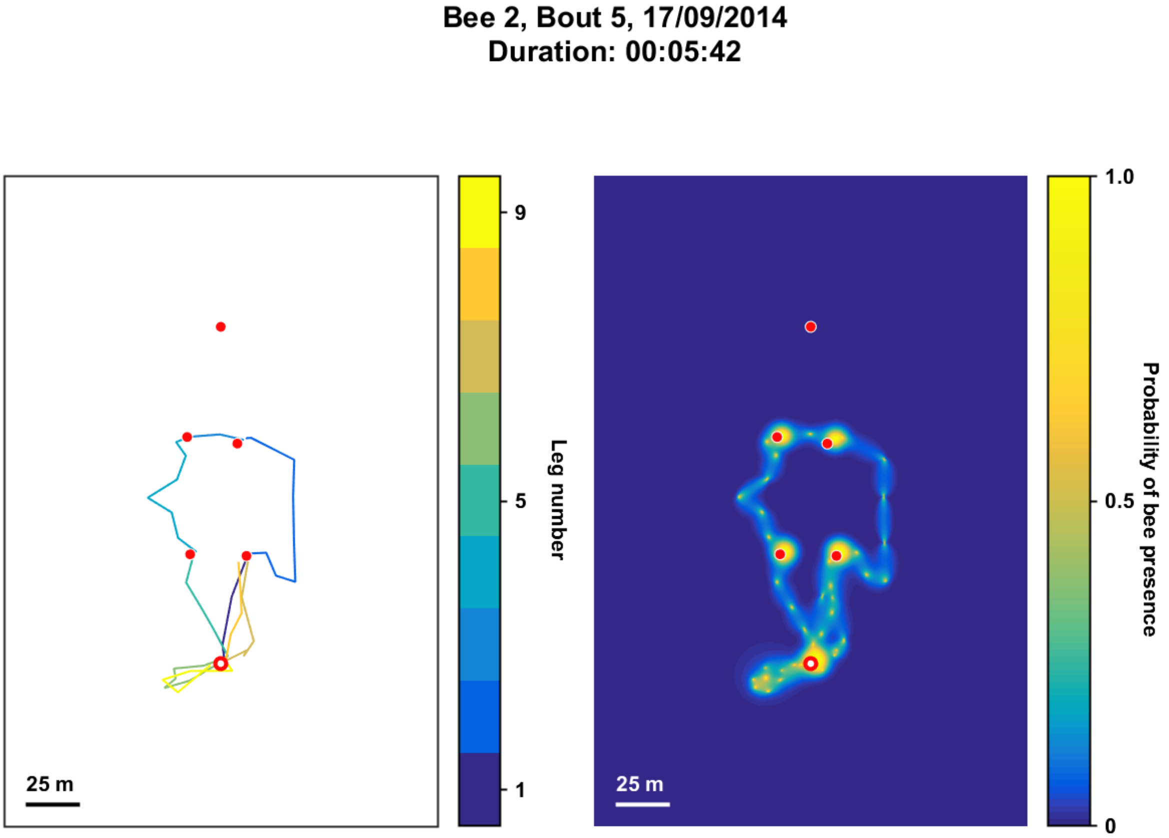

Bee 2, Bout 5, 17/09/2014
Duration: 00:05:42
25 m
9
5
1
Leg number
25 m
1.0
0.5
0
Probability of bee presence

Fig S53

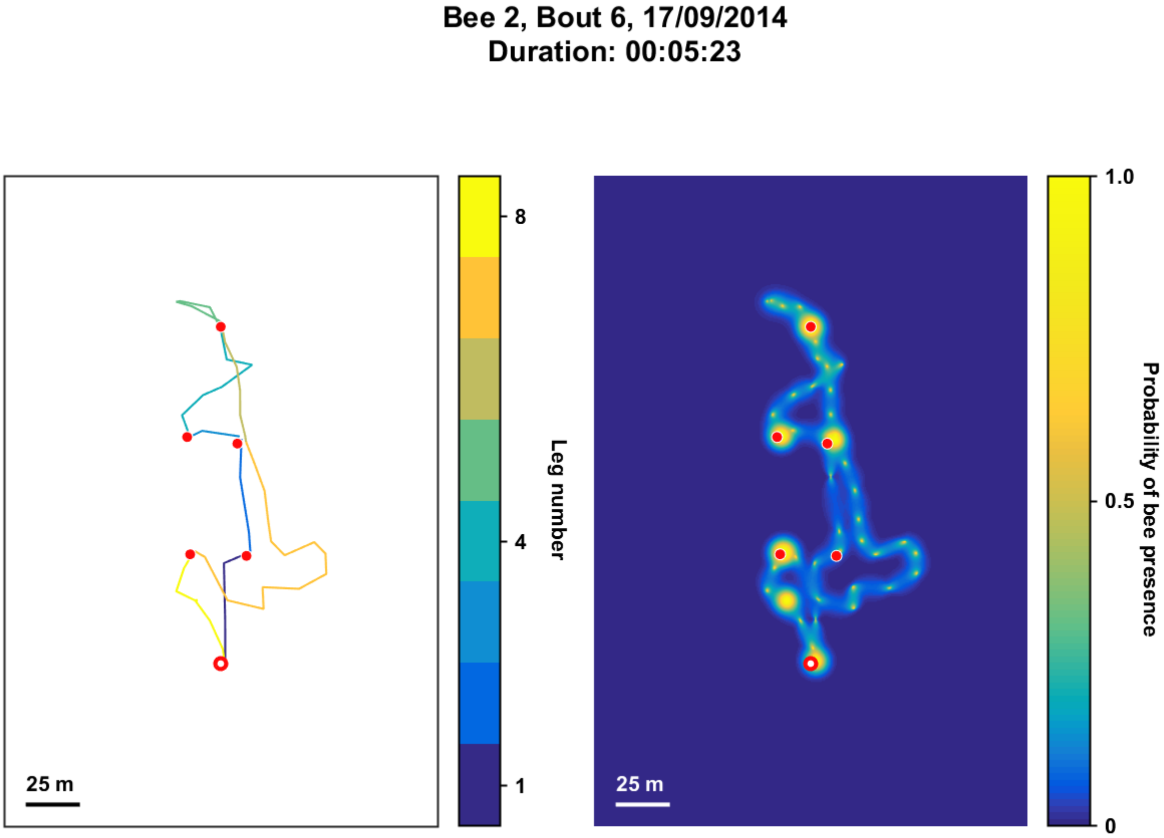

Bee 2, Bout 6, 17/09/2014
Duration: 00:05:23
25 m
8
4
1
Leg number
25 m
1.0
0.5
0
Probability of bee presence

Fig S54

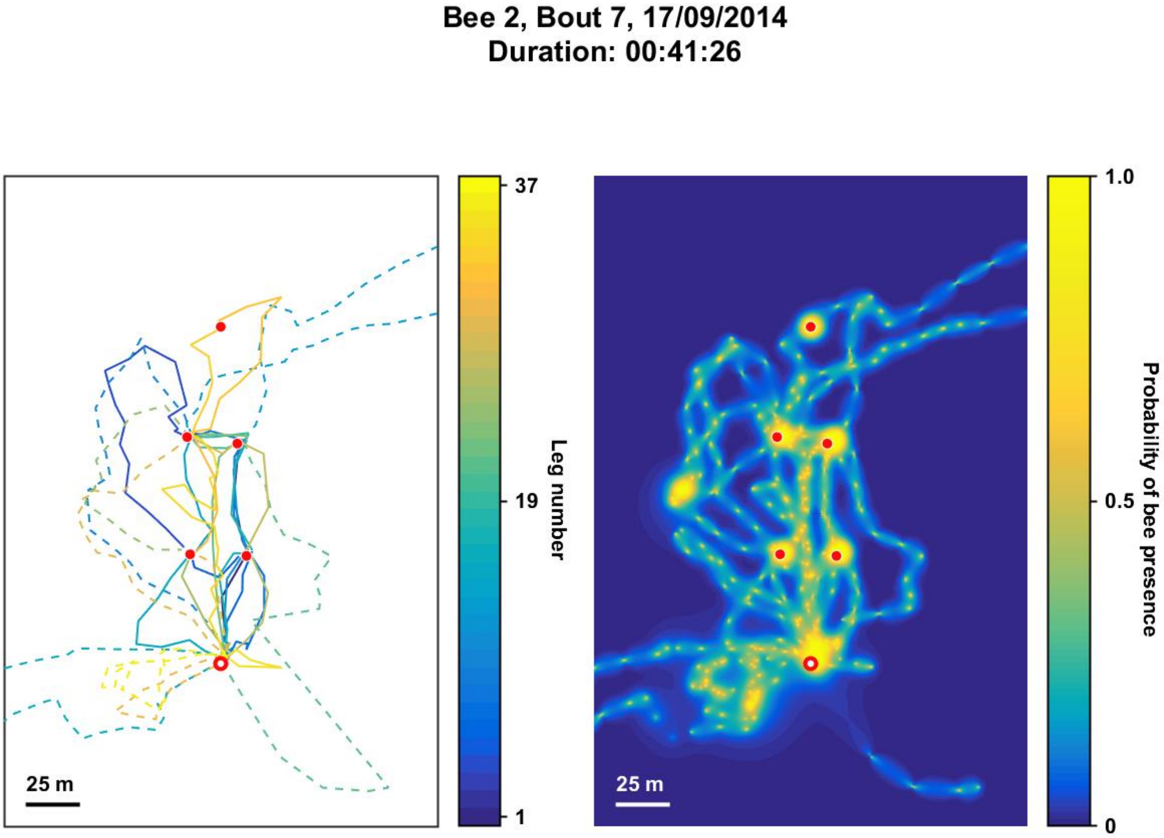
Bee 2, Bout 7, 17/09/2014
Duration: 00:41:26
25 m
37
19
1
Leg number
25 m
1.0
0.5
0
Probability of bee presence

Fig S55

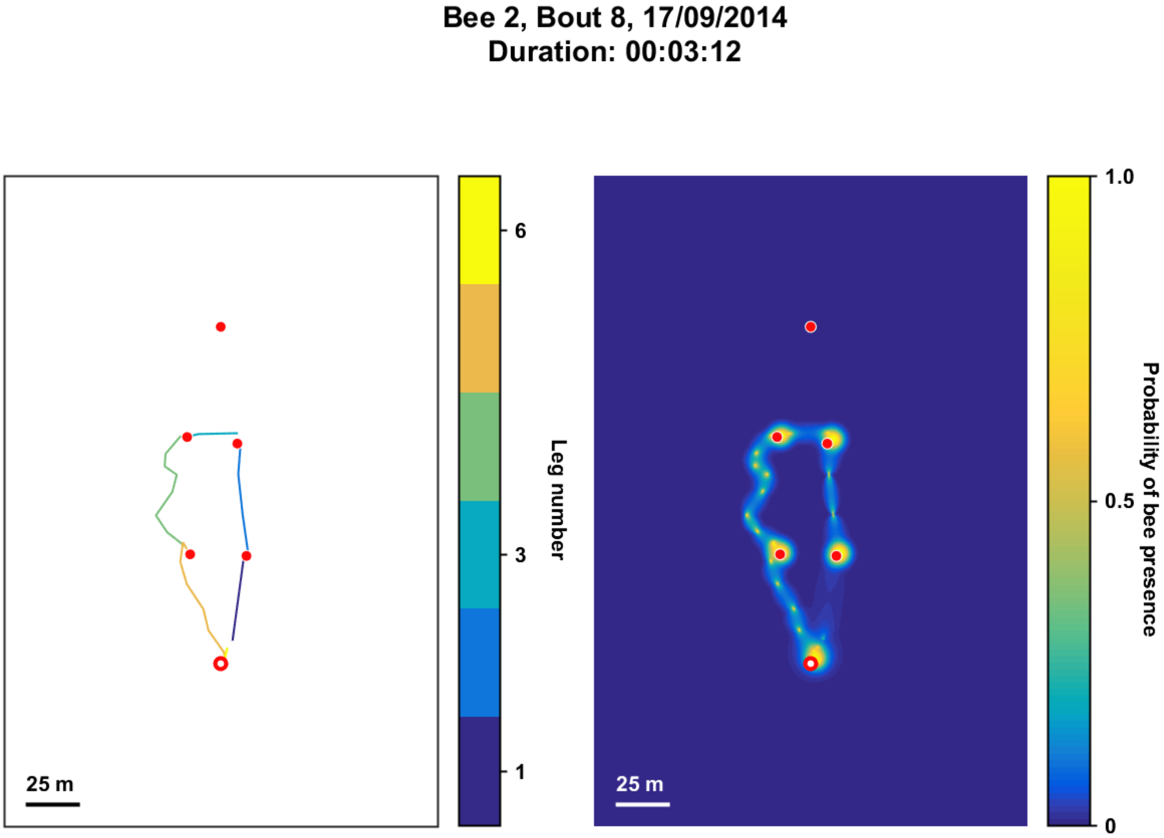
Bee 2, Bout 8, 17/09/2014
Duration: 00:03:12
25 m
Leg number
6
3
1
25 m
Probability of bee presence
1.0
0.5
0

Fig S56

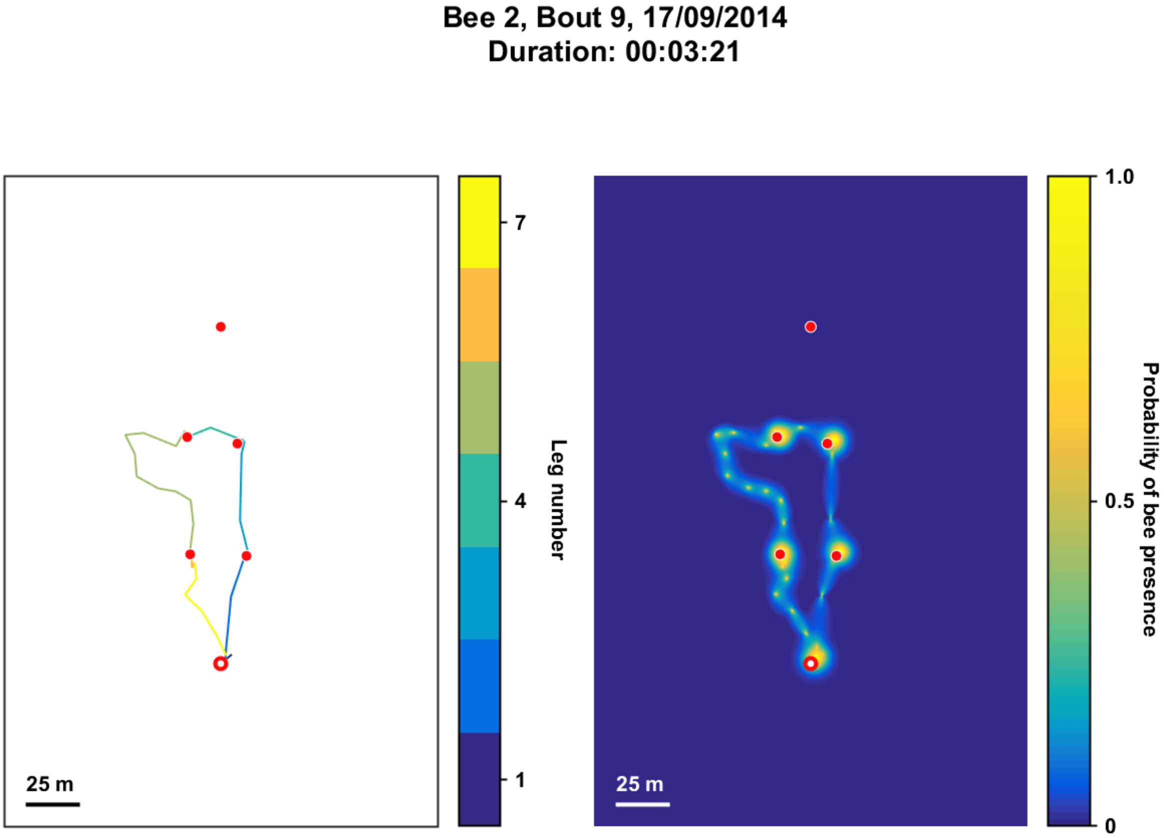

Bee 2, Bout 9, 17/09/2014
Duration: 00:03:21
25 m
7
4
1
Leg number
25 m
1.0
0.5
0
Probability of bee presence

Fig S57

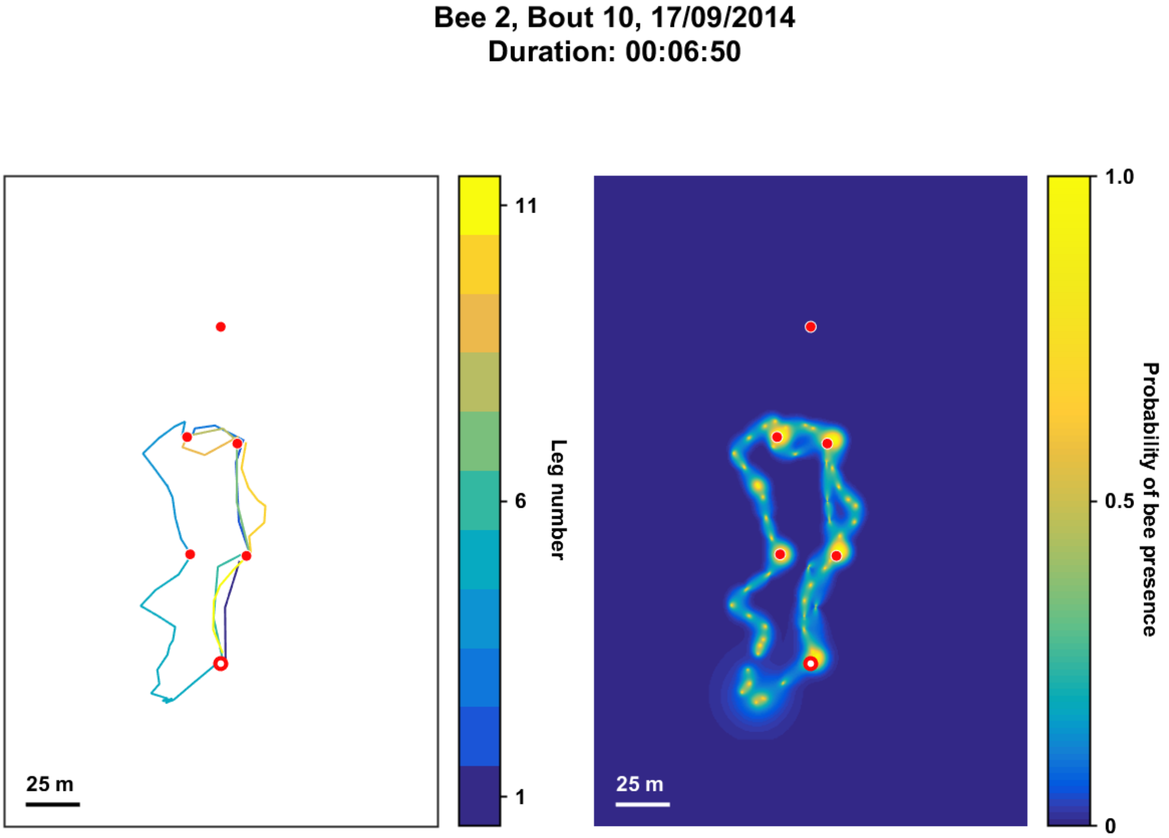

Bee 2, Bout 10, 17/09/2014
Duration: 00:06:50
25 m
11
6
1
Leg number
25 m
1.0
0.5
0
Probability of bee presence

Fig S58

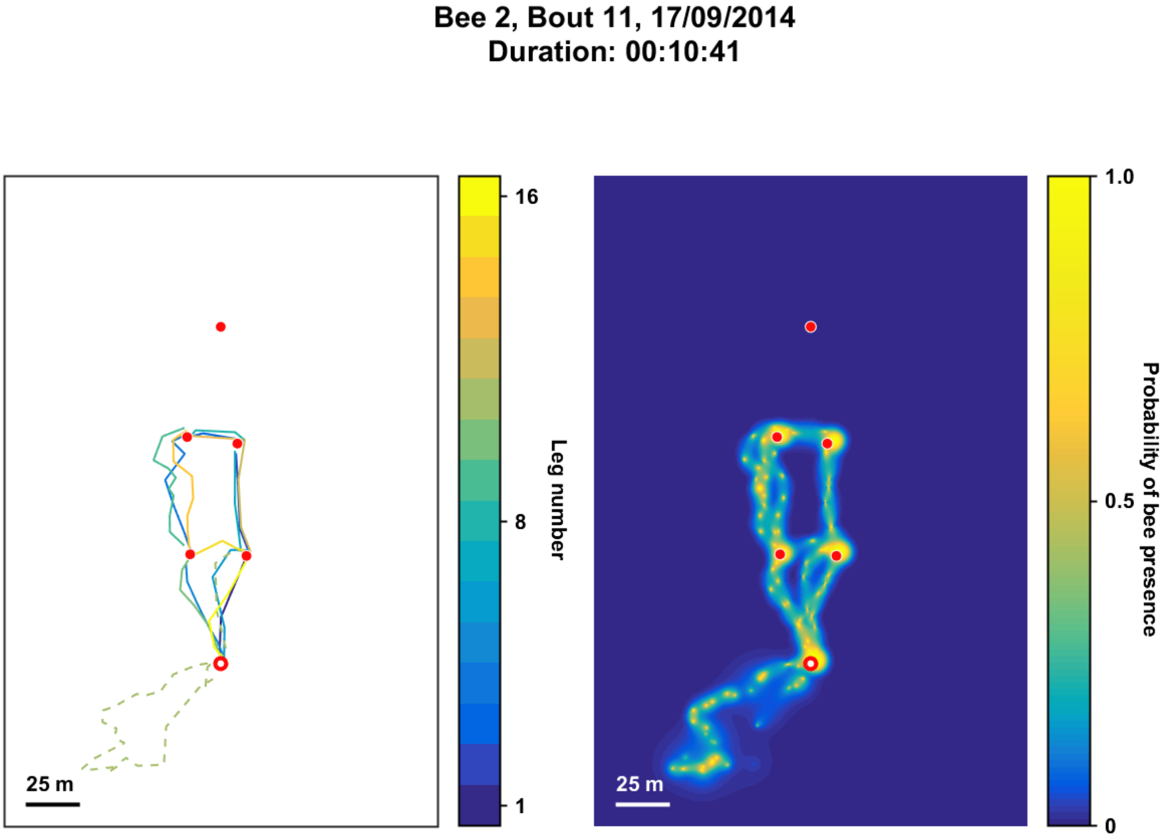

Bee 2, Bout 11, 17/09/2014
Duration: 00:10:41
25 m
16
8
1
Leg number
25 m
1.0
0.5
0
Probability of bee presence

Fig S59

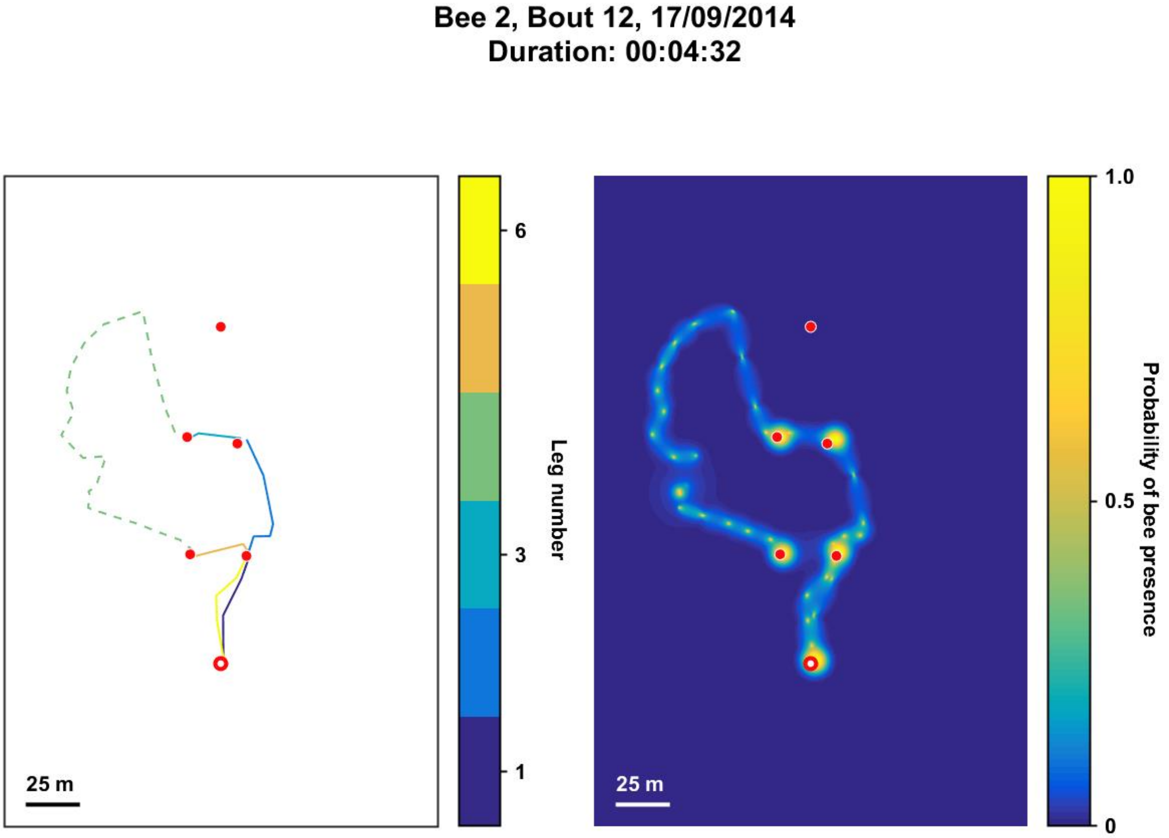

Bee 2, Bout 12, 17/09/2014
Duration: 00:04:32
25 m
Leg number
6
3
1
25 m
Probability of bee presence
1.0
0.5
0

Fig S60

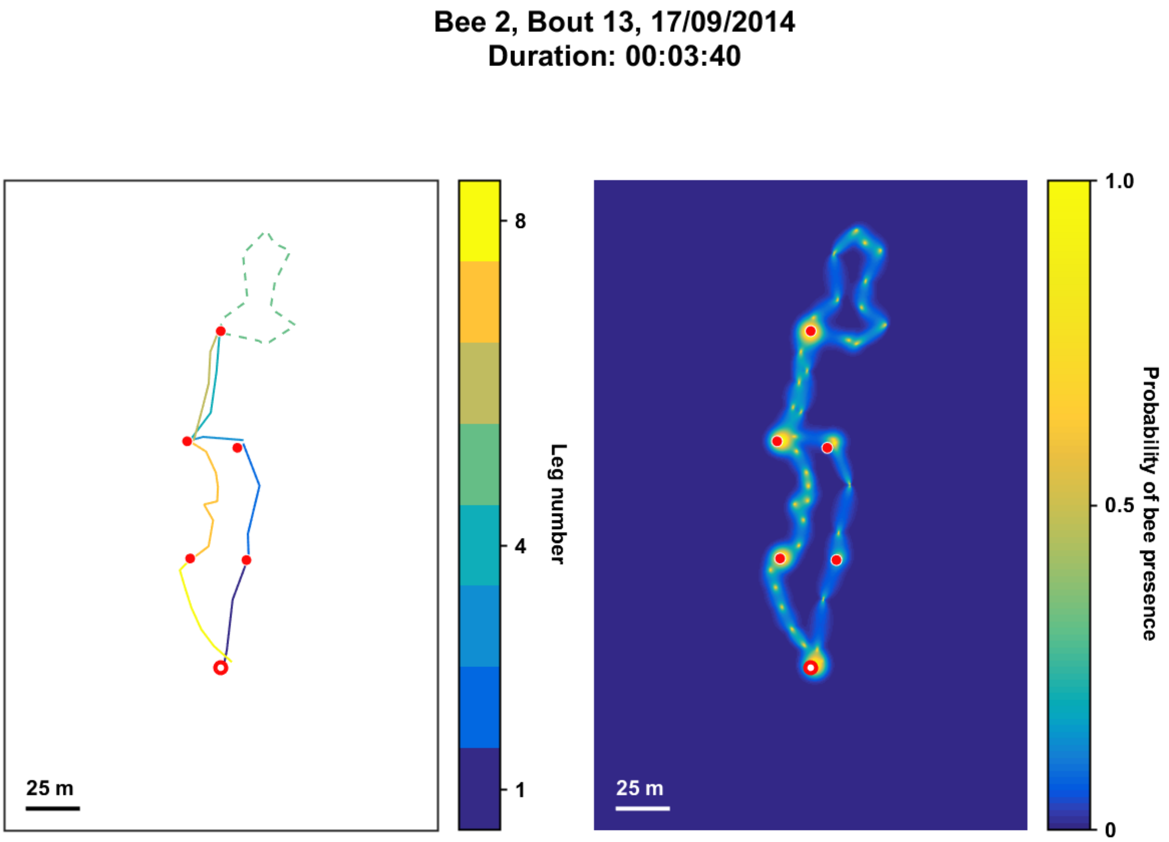
Bee 2, Bout 13, 17/09/2014
Duration: 00:03:40
25 m
8
4
1
Leg number
25 m
1.0
0.5
0
Probability of bee presence

Fig S61

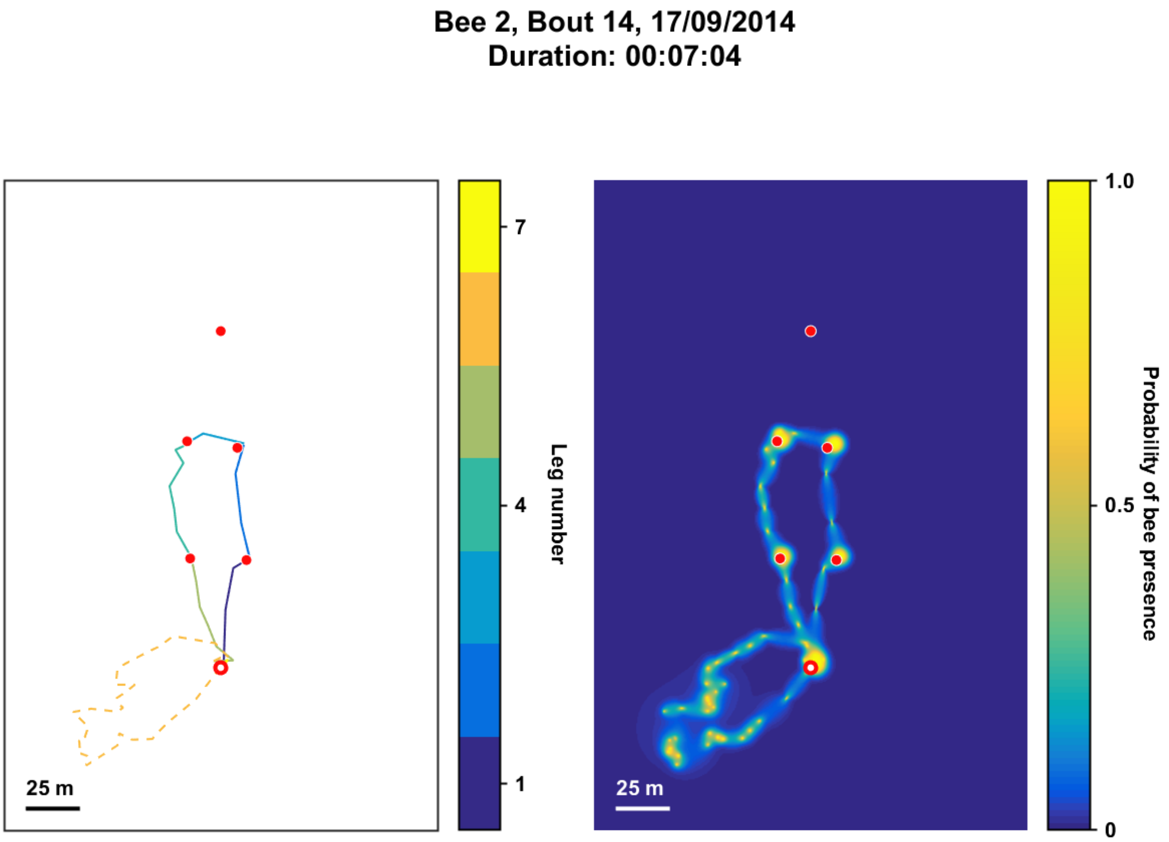
Bee 2, Bout 14, 17/09/2014
Duration: 00:07:04
25 m
7
4
1
Leg number
25 m
1.0
0.5
0
Probability of bee presence

Fig S62

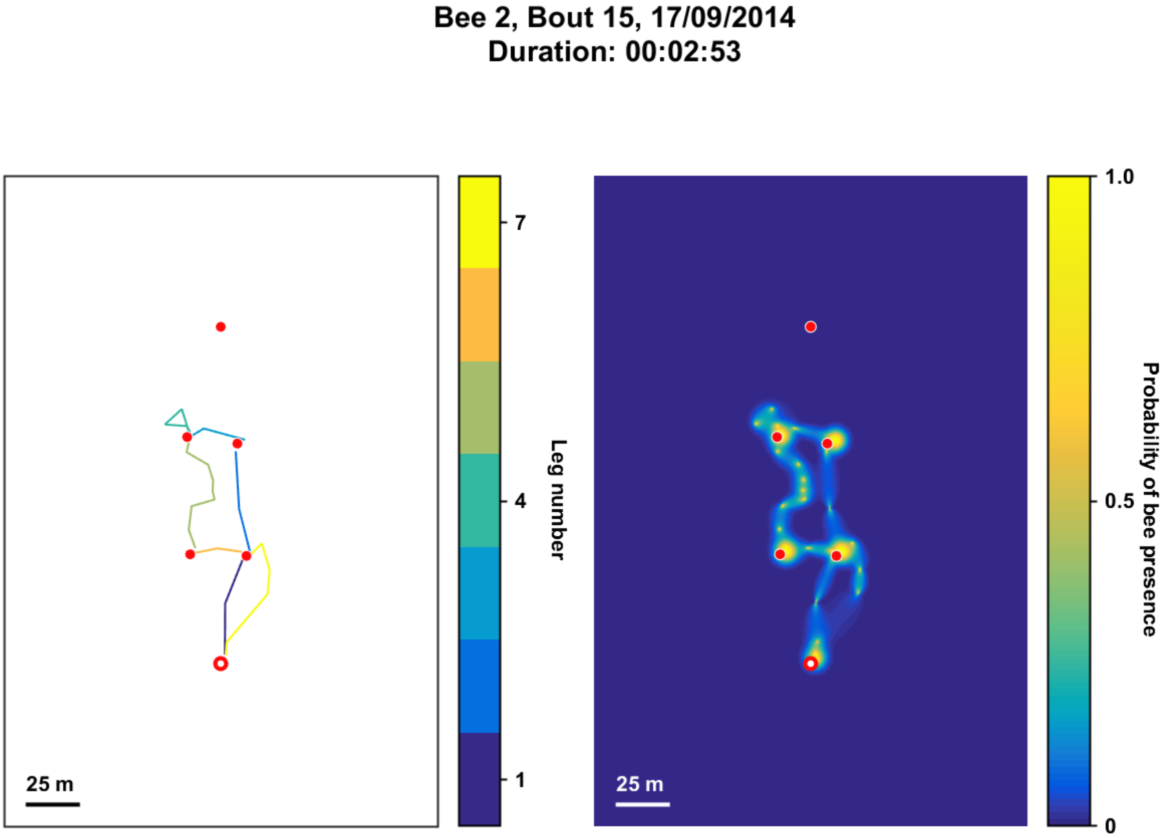
Bee 2, Bout 15, 17/09/2014
Duration: 00:02:53
25 m
7
4
1
Leg number
25 m
1.0
0.5
0
Probability of bee presence

Fig S63

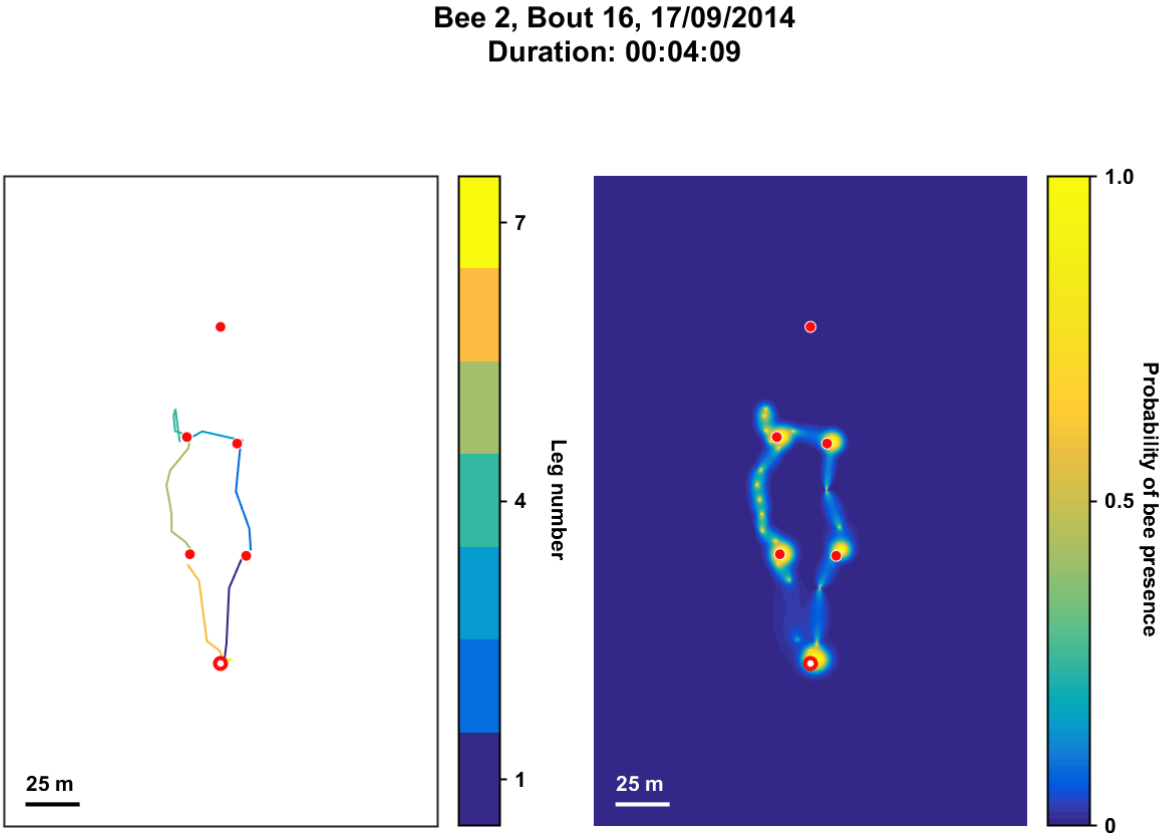
Bee 2, Bout 16, 17/09/2014
Duration: 00:04:09
25 m
Leg number
7
4
1
25 m
Probability of bee presence
1.0
0.5
0

Fig S64

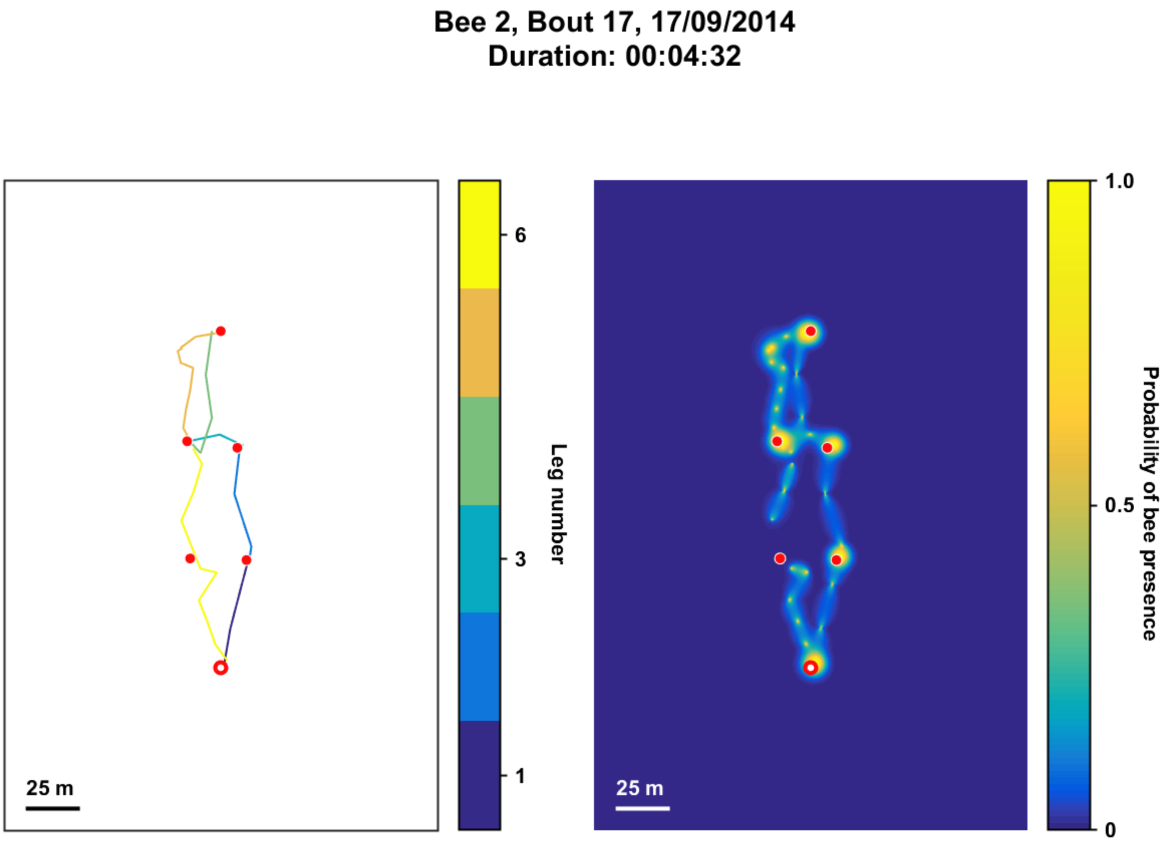

Bee 2, Bout 17, 17/09/2014
Duration: 00:04:32
25 m
6
3
1
Leg number
25 m
1.0
0.5
0
Probability of bee presence

Fig S65

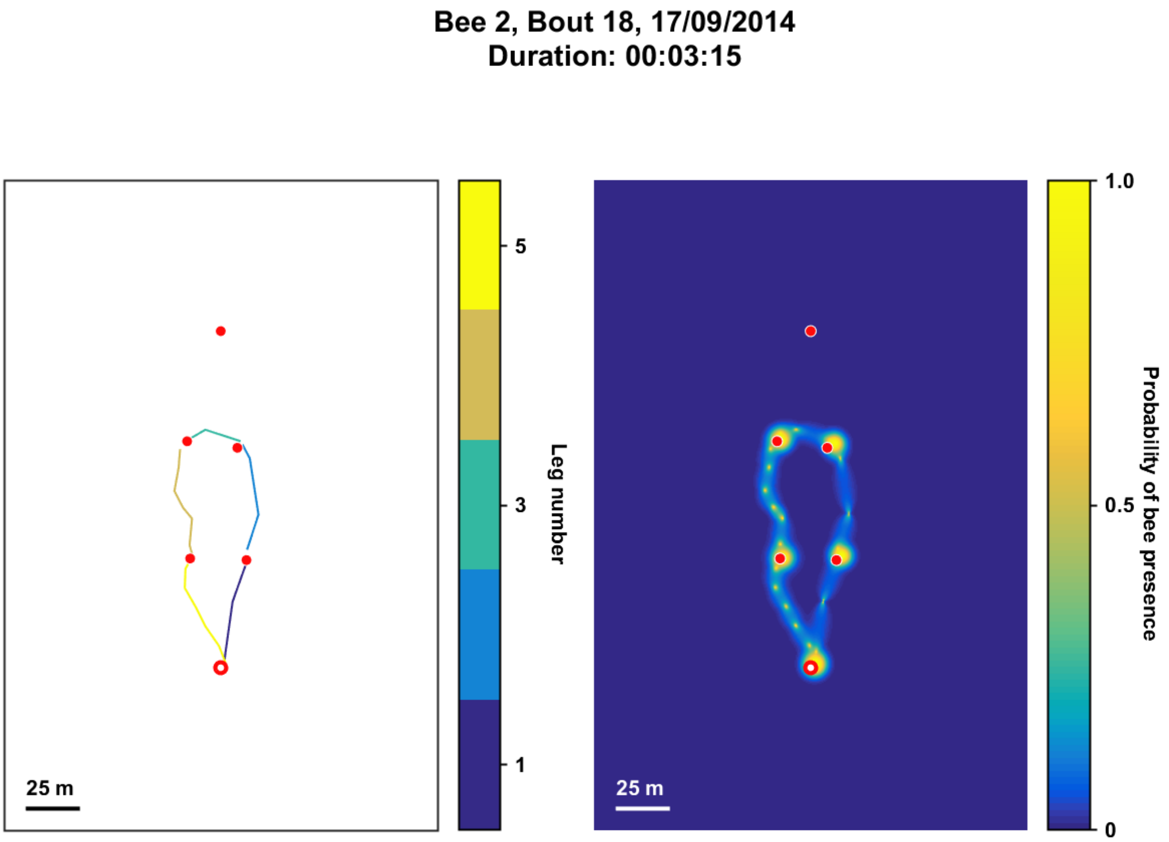
Bee 2, Bout 18, 17/09/2014
Duration: 00:03:15
25 m
5
3
1
Leg number
25 m
1.0
0.5
0
Probability of bee presence

Fig S66

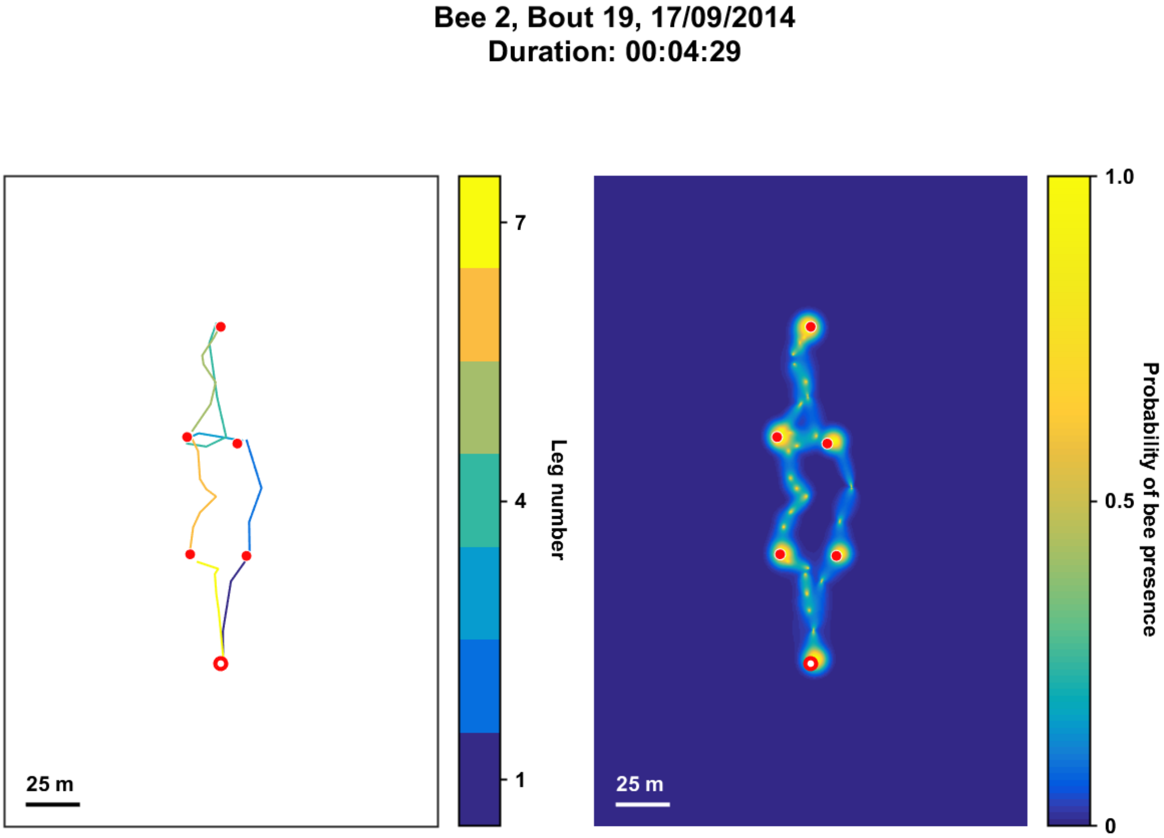
Bee 2, Bout 19, 17/09/2014
Duration: 00:04:29
25 m
Leg number
1
4
7
25 m
Probability of bee presence
0
0.5
1.0

Fig S67

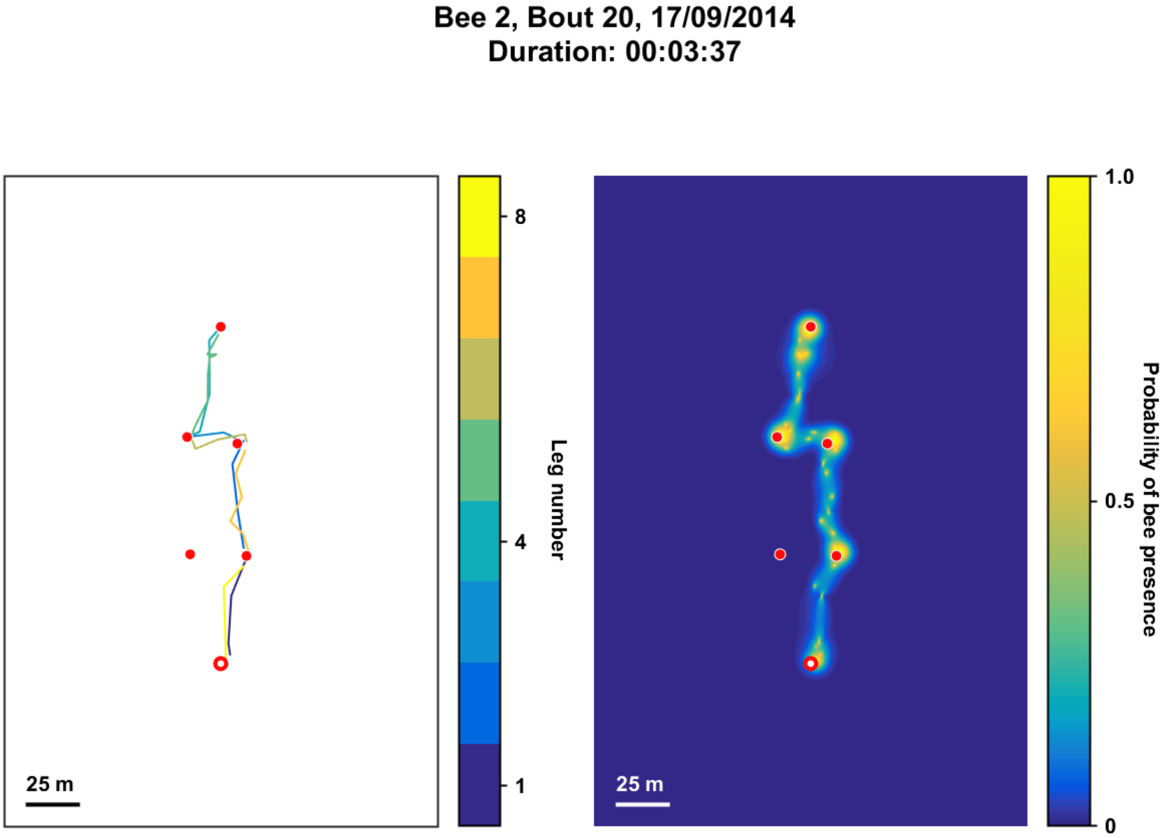

Bee 2, Bout 20, 17/09/2014
Duration: 00:03:37
25 m
8
4
1
Leg number
25 m
1.0
0.5
0
Probability of bee presence

Fig S68

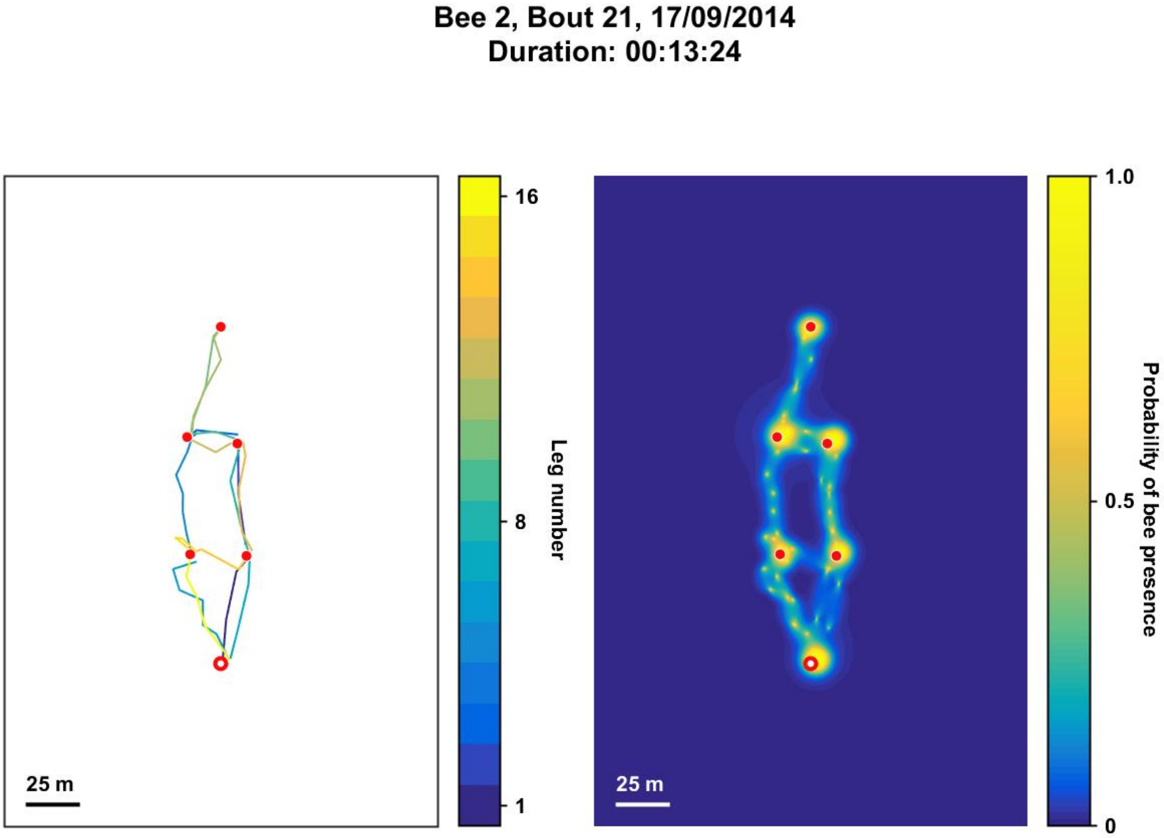
Bee 2, Bout 21, 17/09/2014
Duration: 00:13:24
25 m
16
8
1
Leg number
25 m
1.0
0.5
0
Probability of bee presence

Fig S69

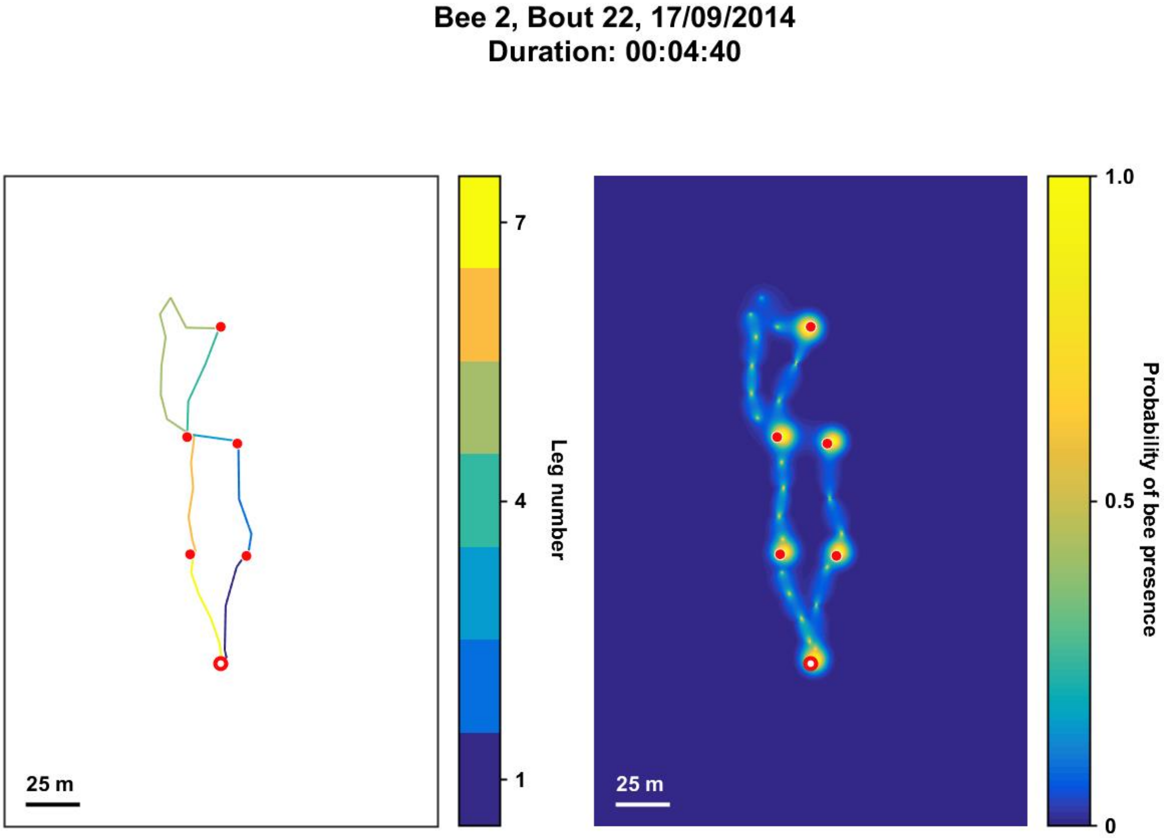

Bee 2, Bout 22, 17/09/2014
Duration: 00:04:40
25 m
7
4
1
Leg number
25 m
1.0
0.5
0
Probability of bee presence

Fig S70

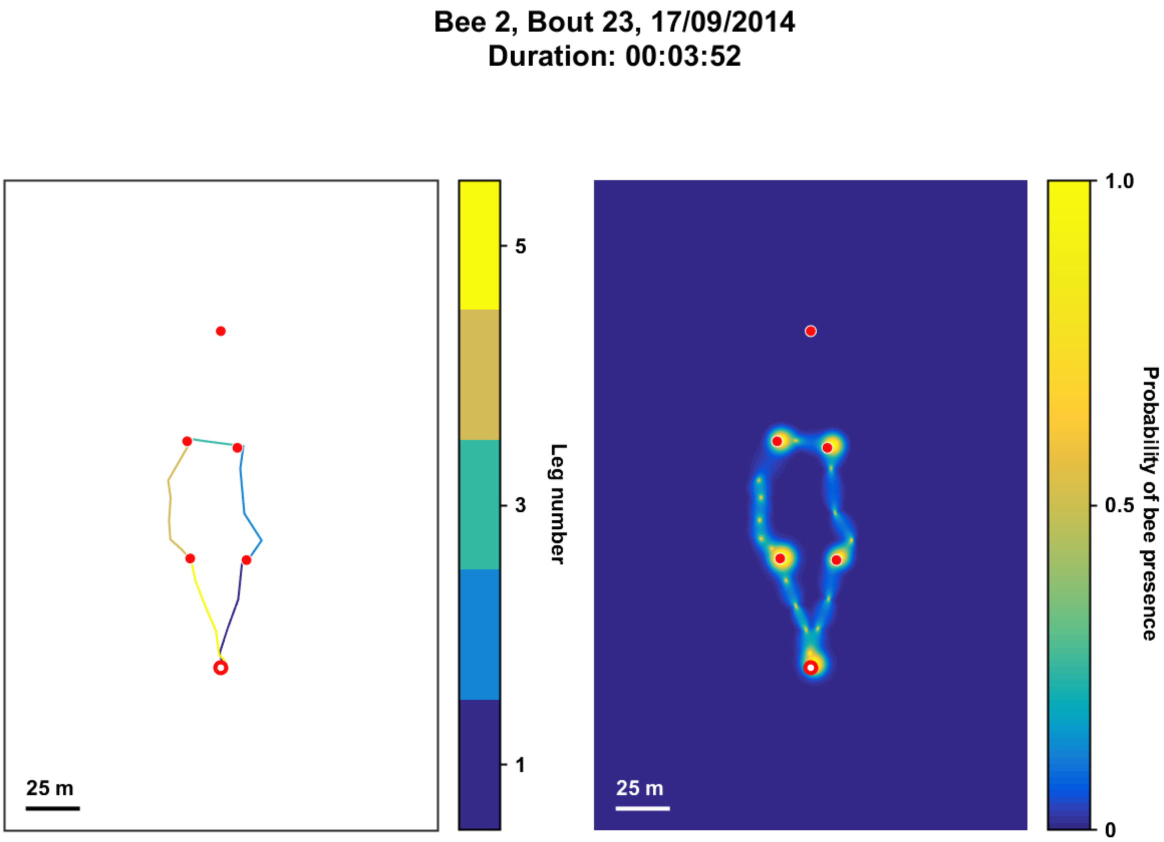

Bee 2, Bout 23, 17/09/2014
Duration: 00:03:52
25 m
5
3
1
Leg number
25 m
1.0
0.5
0
Probability of bee presence

Fig S71

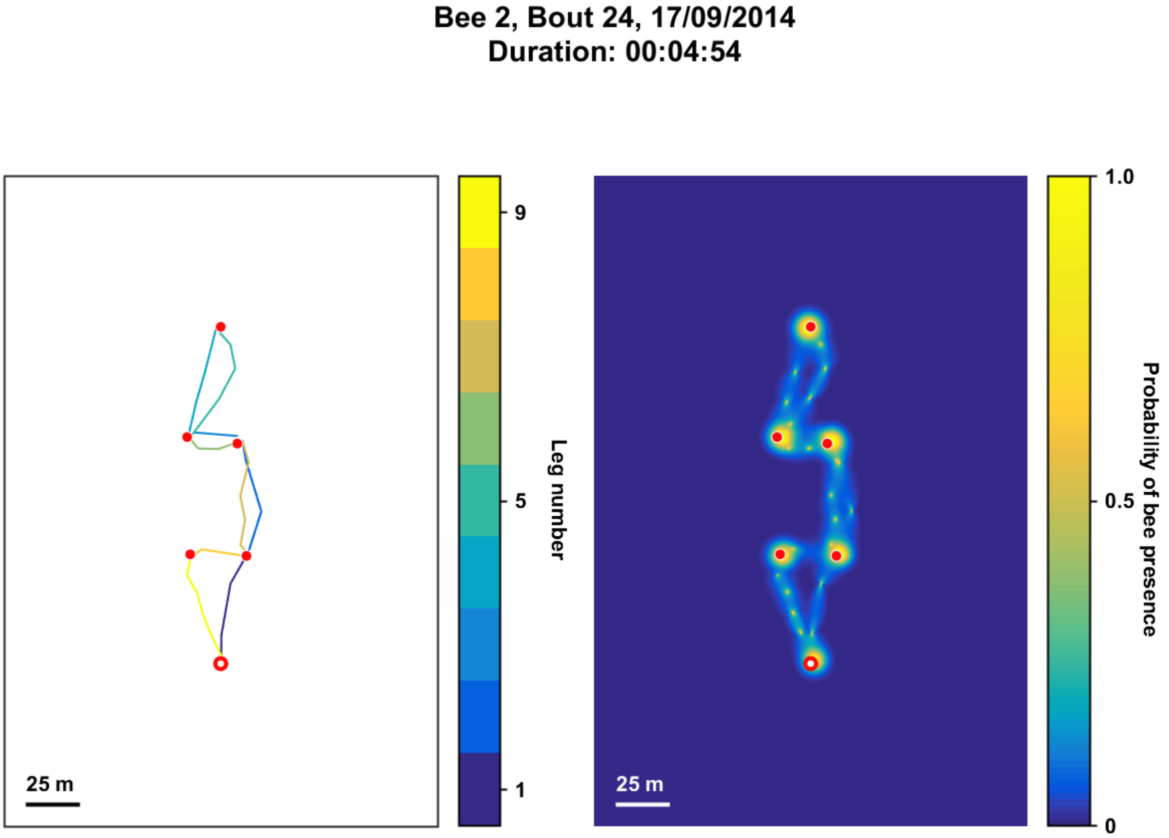

Bee 2, Bout 24, 17/09/2014
Duration: 00:04:54
25 m
9
5
1
Leg number
25 m
1.0
0.5
0
Probability of bee presence

Fig S72

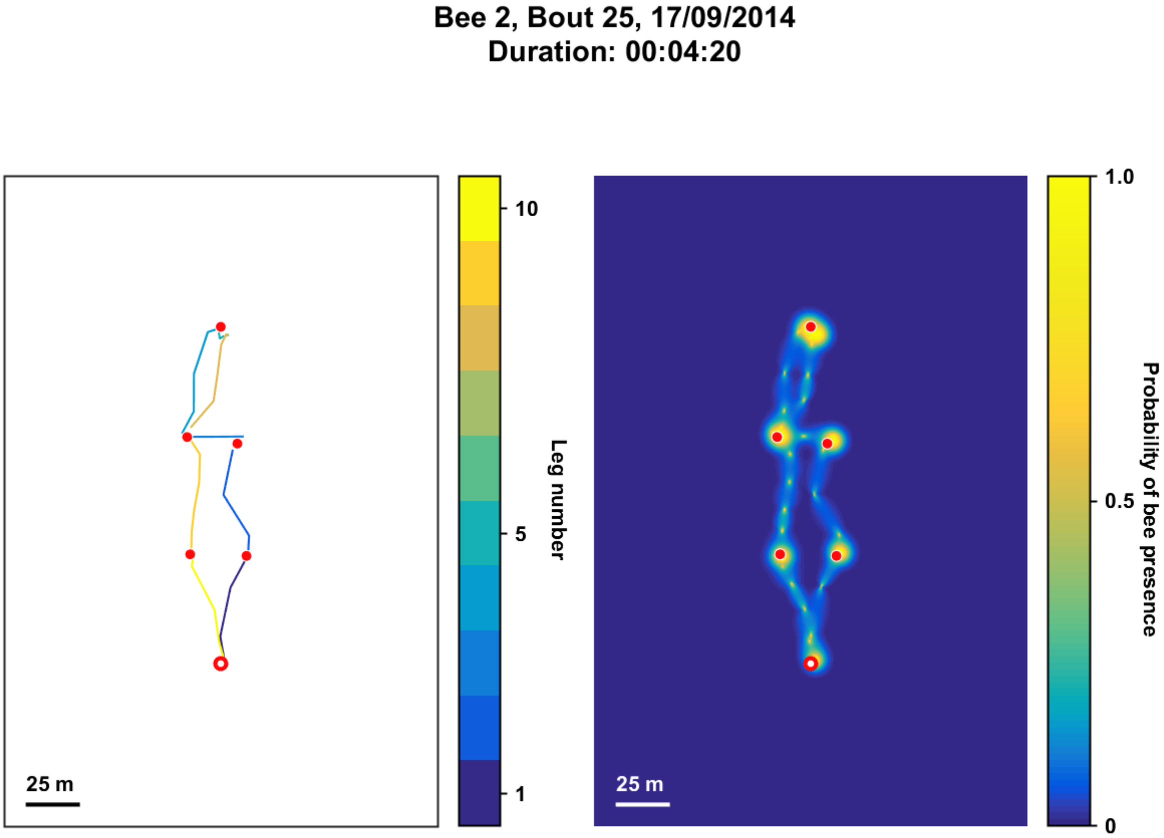

Bee 2, Bout 25, 17/09/2014
Duration: 00:04:20
25 m
10
5
1
Leg number
25 m
1.0
0.5
0
Probability of bee presence

Fig S73

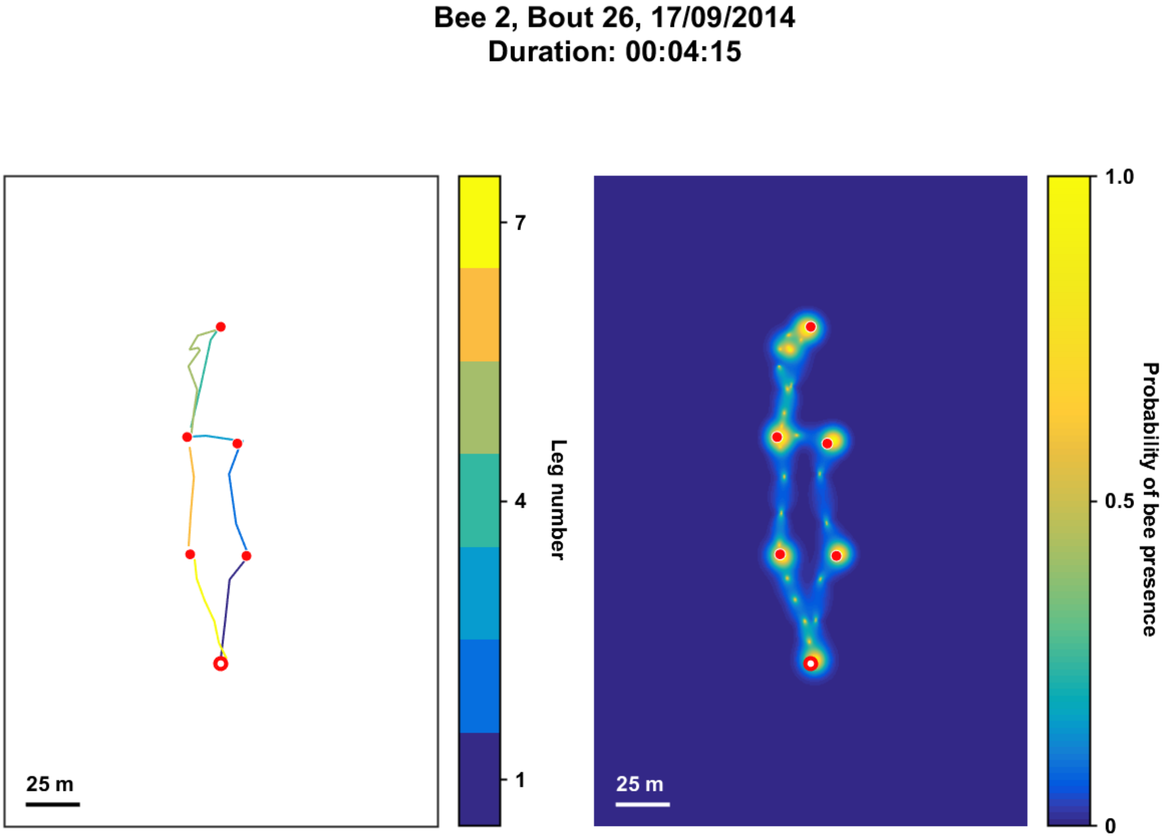
Bee 2, Bout 26, 17/09/2014
Duration: 00:04:15
25 m
7
4
1
Leg number
25 m
1.0
0.5
0
Probability of bee presence

Fig S74

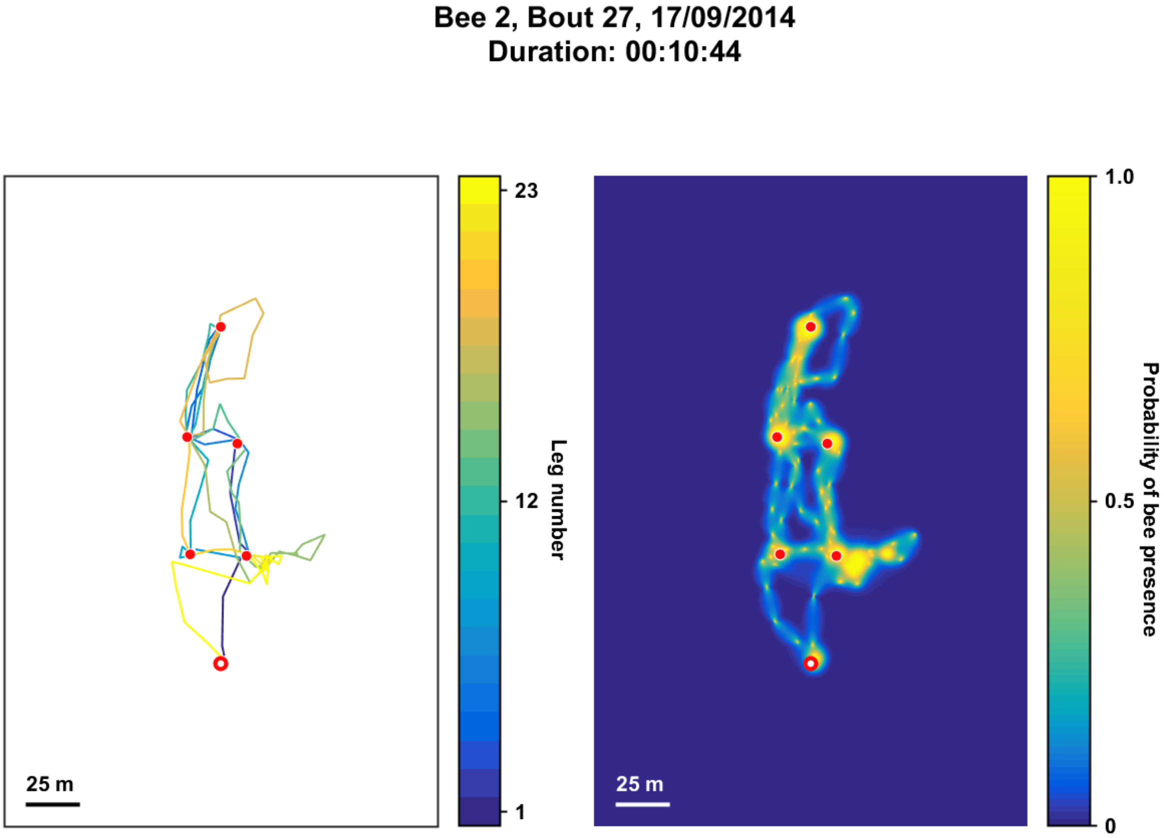
Bee 2, Bout 27, 17/09/2014
Duration: 00:10:44
25 m
23
12
1
Leg number
25 m
1.0
0.5
0
Probability of bee presence

Fig S75

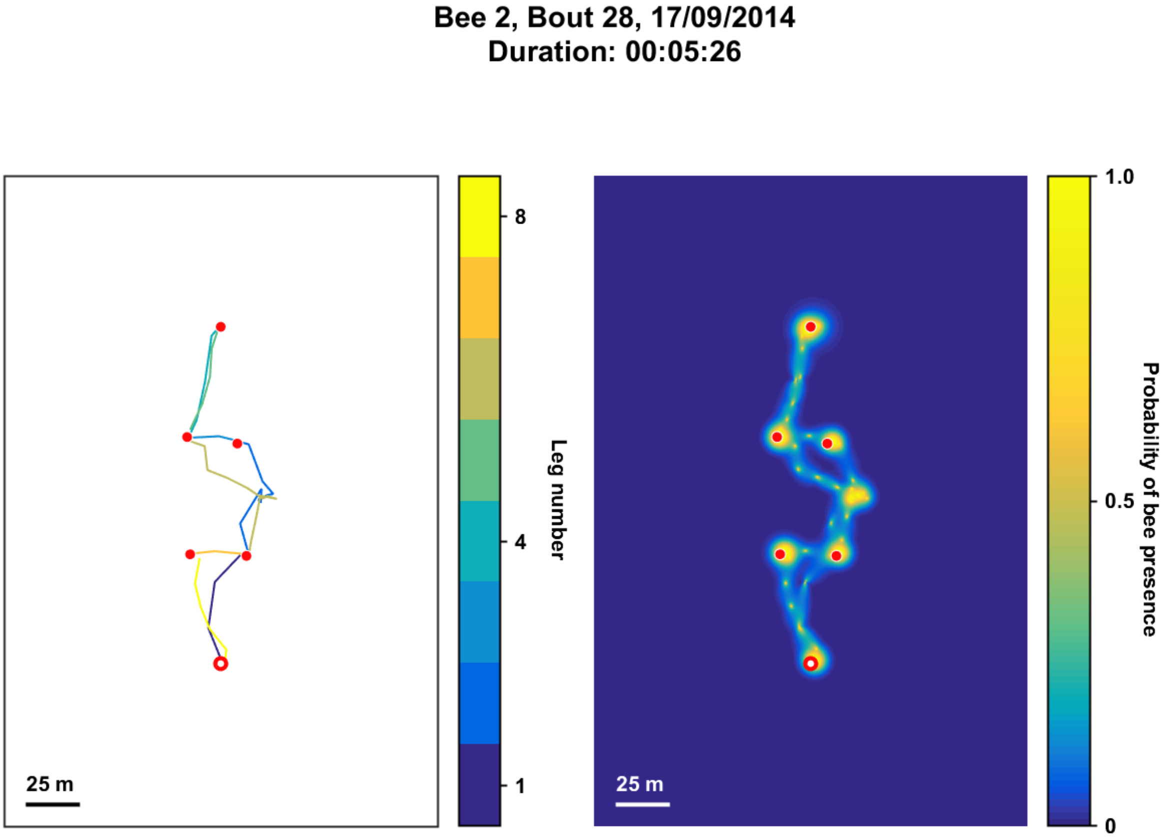

Bee 2, Bout 28, 17/09/2014
Duration: 00:05:26
25 m
8
4
1
Leg number
25 m
1.0
0.5
0
Probability of bee presence

Fig S76

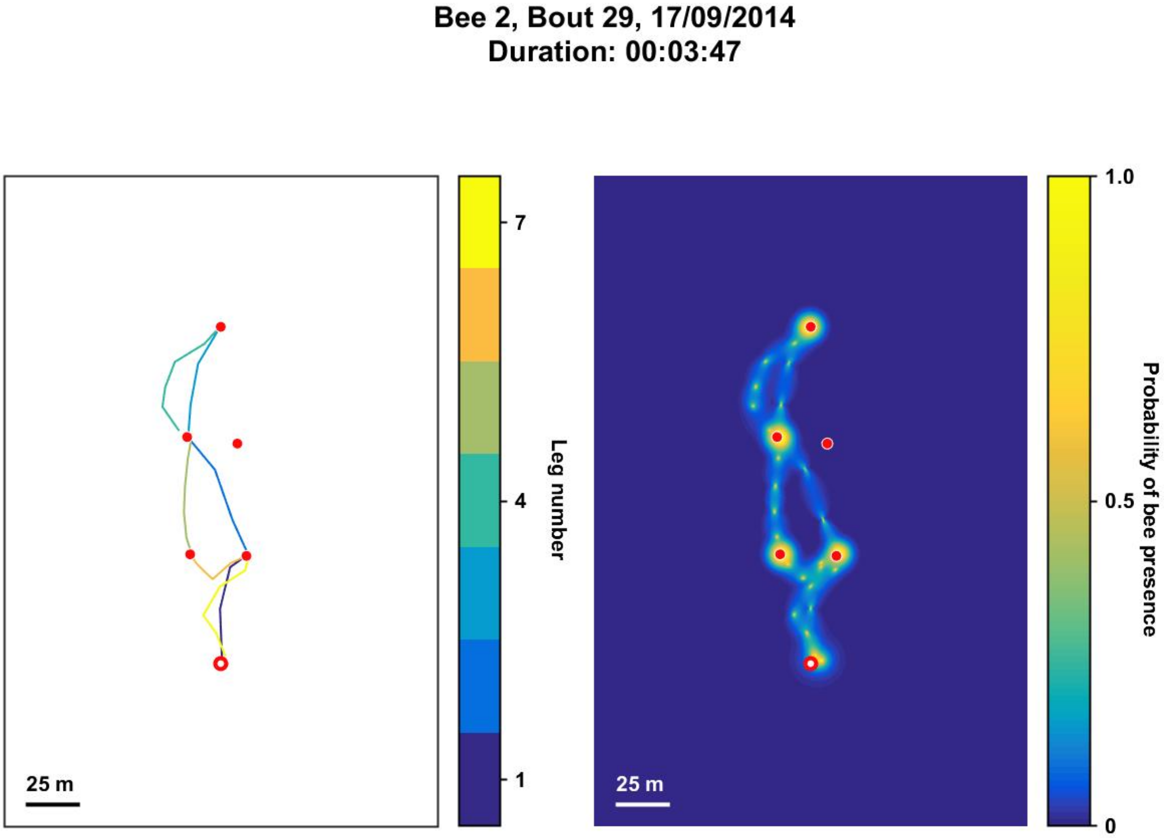

Bee 2, Bout 29, 17/09/2014
Duration: 00:03:47
25 m
7
4
1
Leg number
25 m
1.0
0.5
0
Probability of bee presence

Fig S77

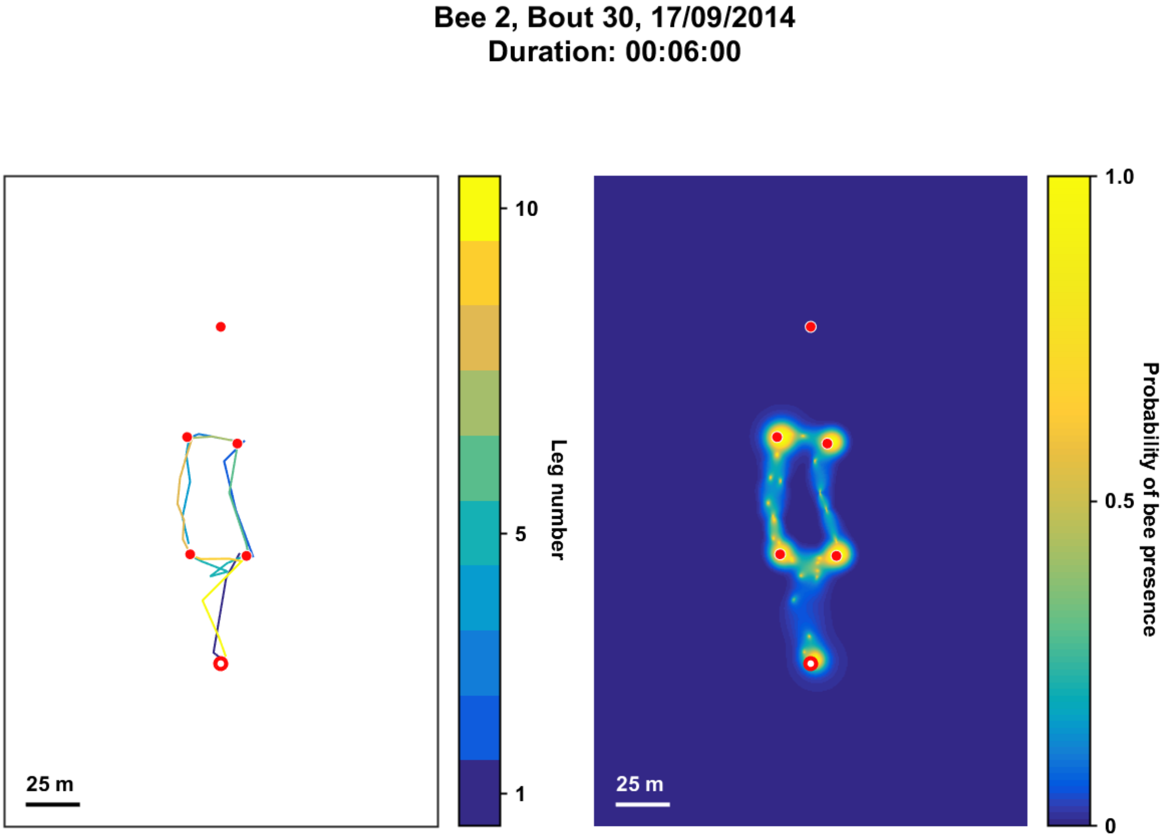

Bee 2, Bout 30, 17/09/2014
Duration: 00:06:00
25 m
Leg number
10
5
1
25 m
Probability of bee presence
1.0
0.5
0

Fig S78

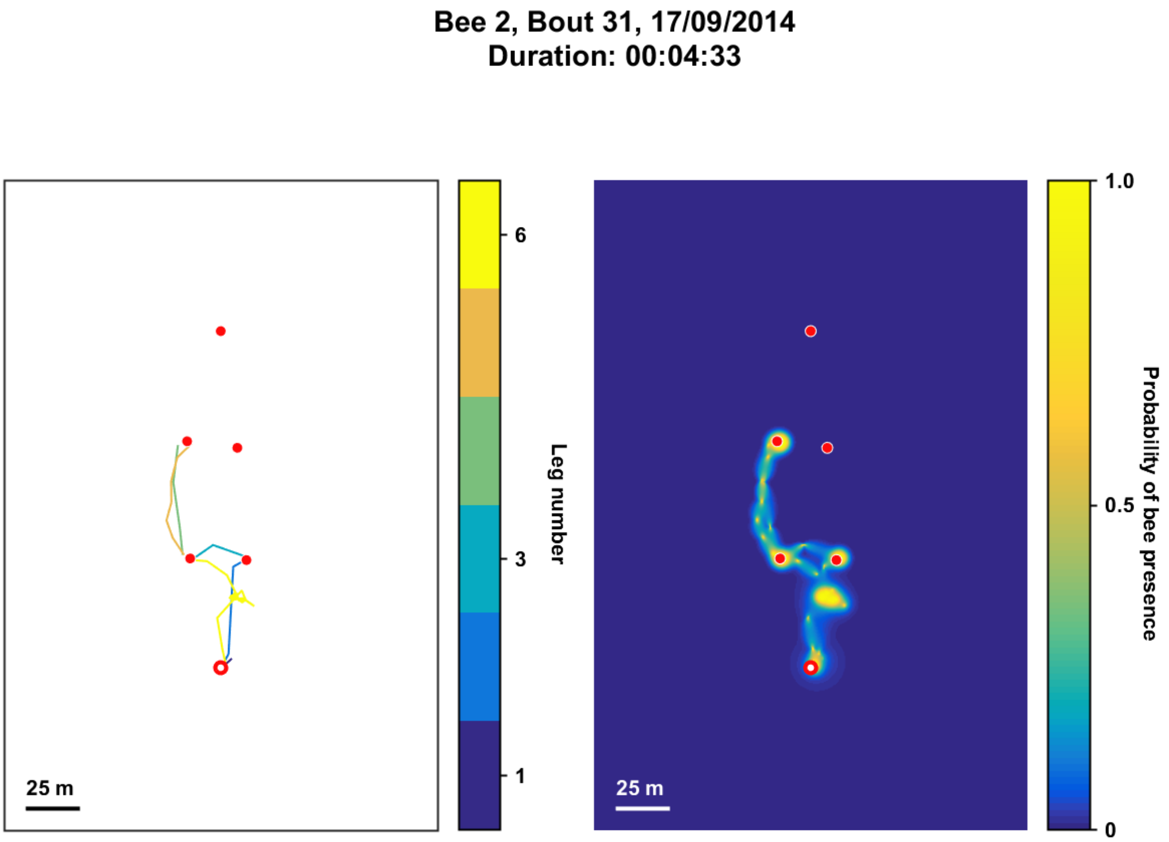

Bee 2, Bout 31, 17/09/2014
Duration: 00:04:33
25 m
6
3
1
Leg number
25 m
1.0
0.5
0
Probability of bee presence

Fig S79

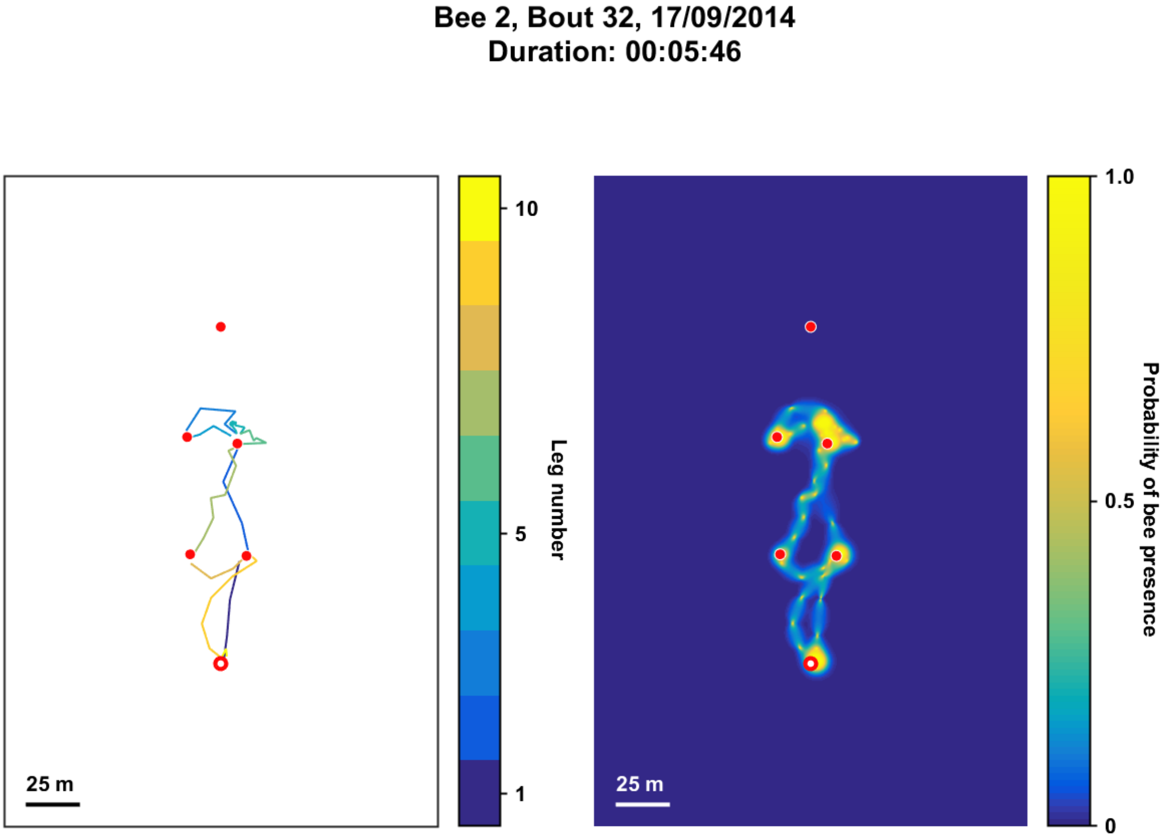

Bee 2, Bout 32, 17/09/2014
Duration: 00:05:46
25 m
10
5
1
Leg number
25 m
1.0
0.5
0
Probability of bee presence

Fig S80

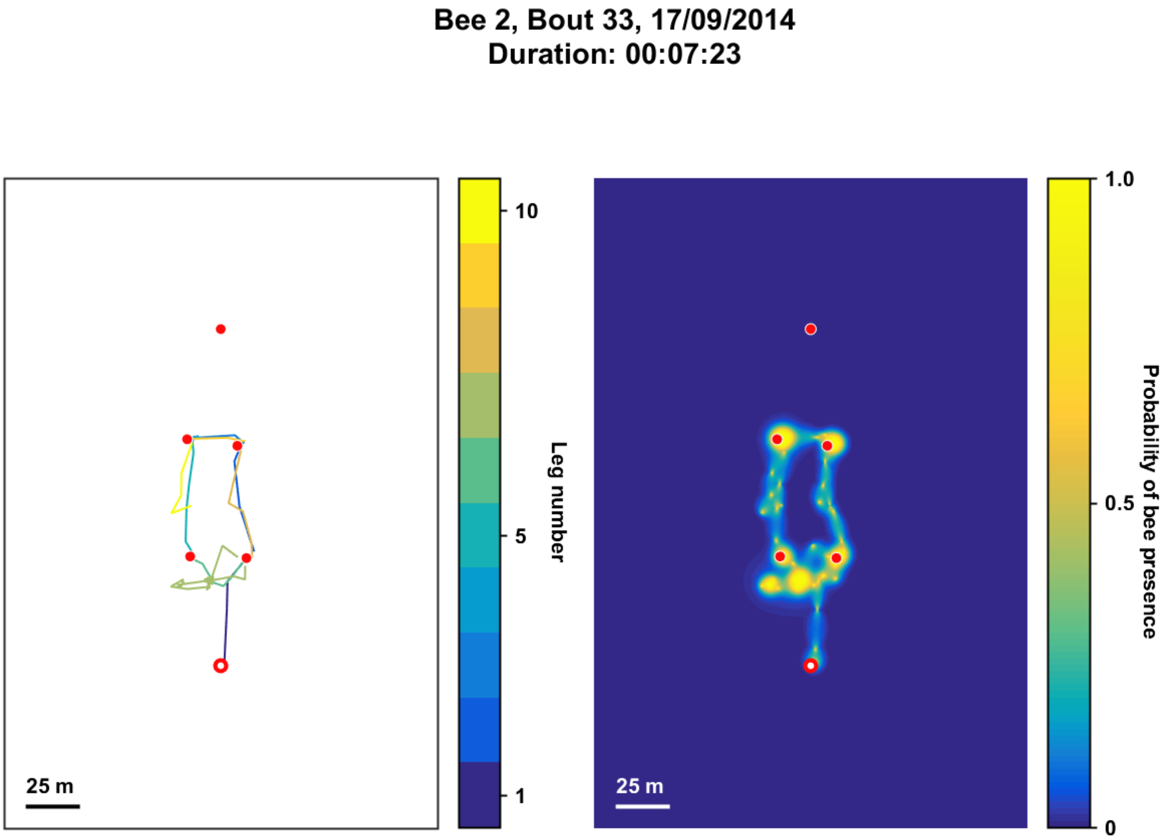

Bee 2, Bout 33, 17/09/2014
Duration: 00:07:23
25 m
10
5
1
Leg number
25 m
1.0
0.5
0
Probability of bee presence

Fig S81

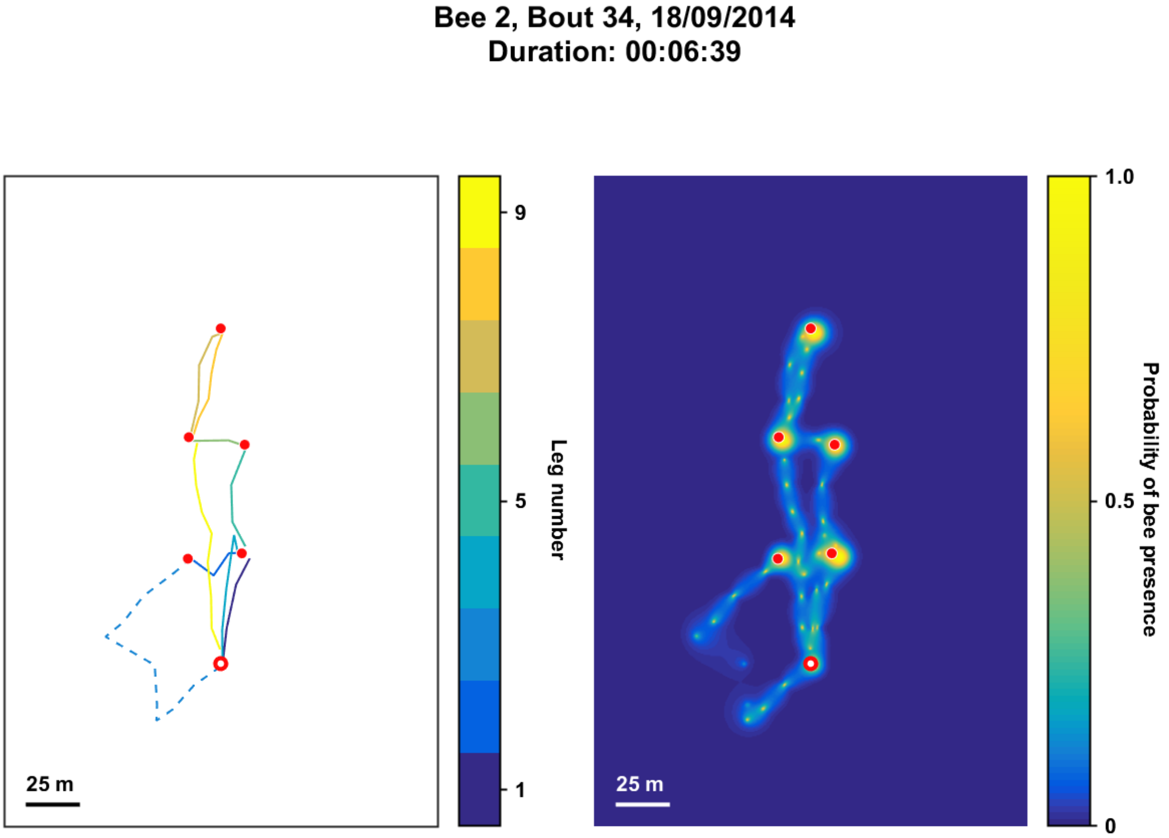
Bee 2, Bout 34, 18/09/2014
Duration: 00:06:39
25 m
9
5
1
Leg number
25 m
1.0
0.5
0
Probability of bee presence

Fig S82

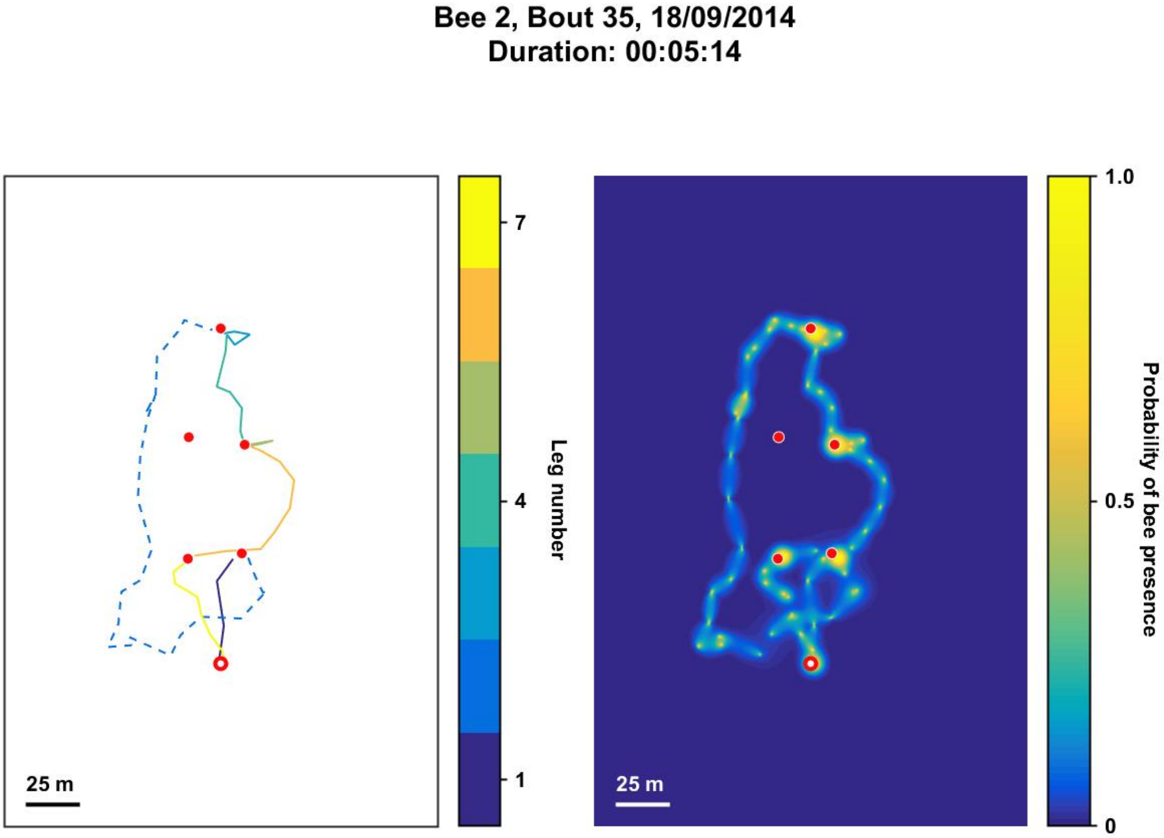

Bee 2, Bout 35, 18/09/2014
Duration: 00:05:14
25 m
Leg number
1
4
7
25 m
Probability of bee presence
0
0.5
1.0

Fig S83

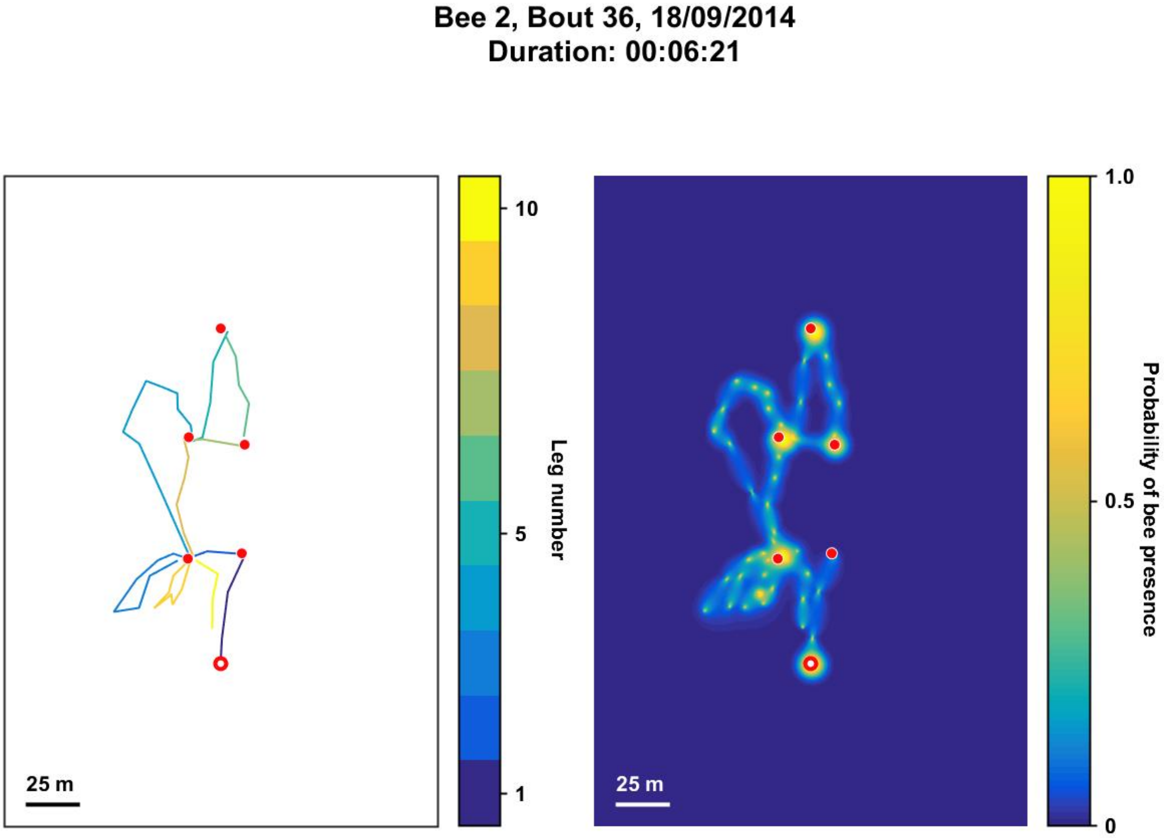

Bee 2, Bout 36, 18/09/2014
Duration: 00:06:21
25 m
Leg number
10
5
1
25 m
Probability of bee presence
1.0
0.5
0

Fig S84

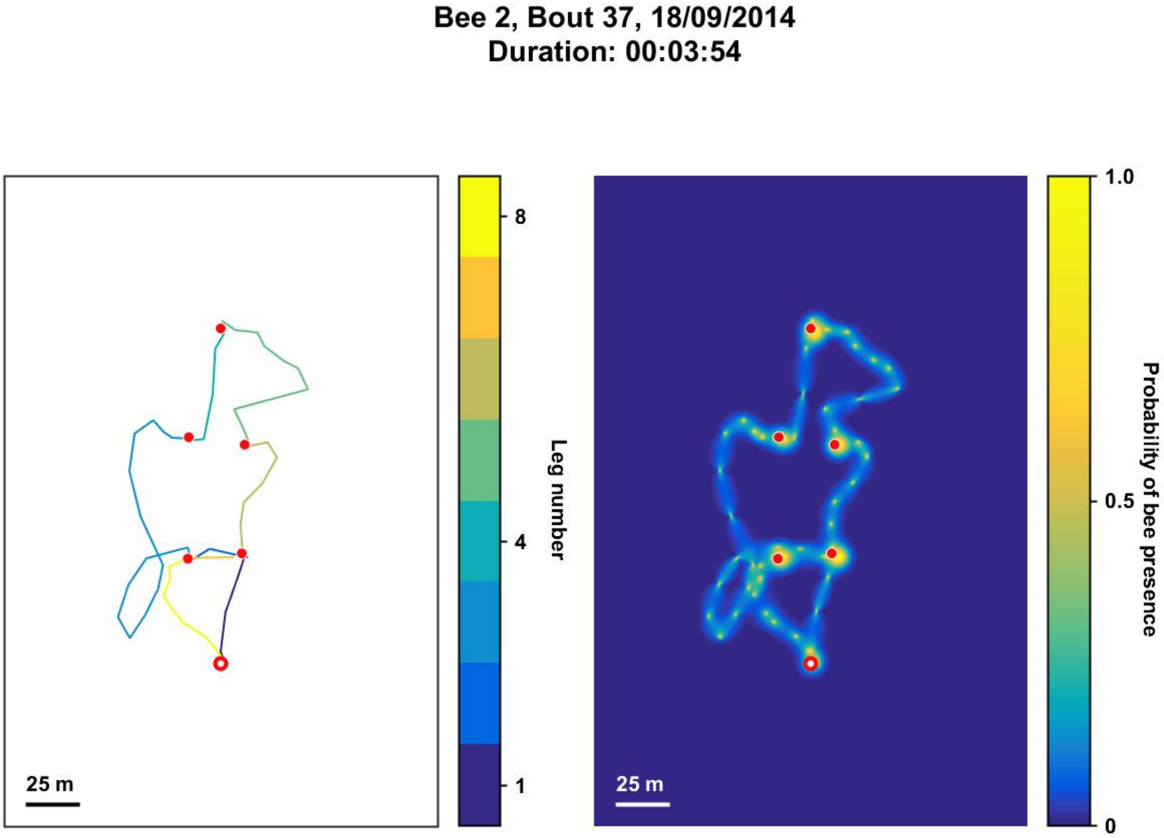
Bee 2, Bout 37, 18/09/2014
Duration: 00:03:54
25 m
8
4
1
Leg number
25 m
1.0
0.5
0
Probability of bee presence

Fig S85

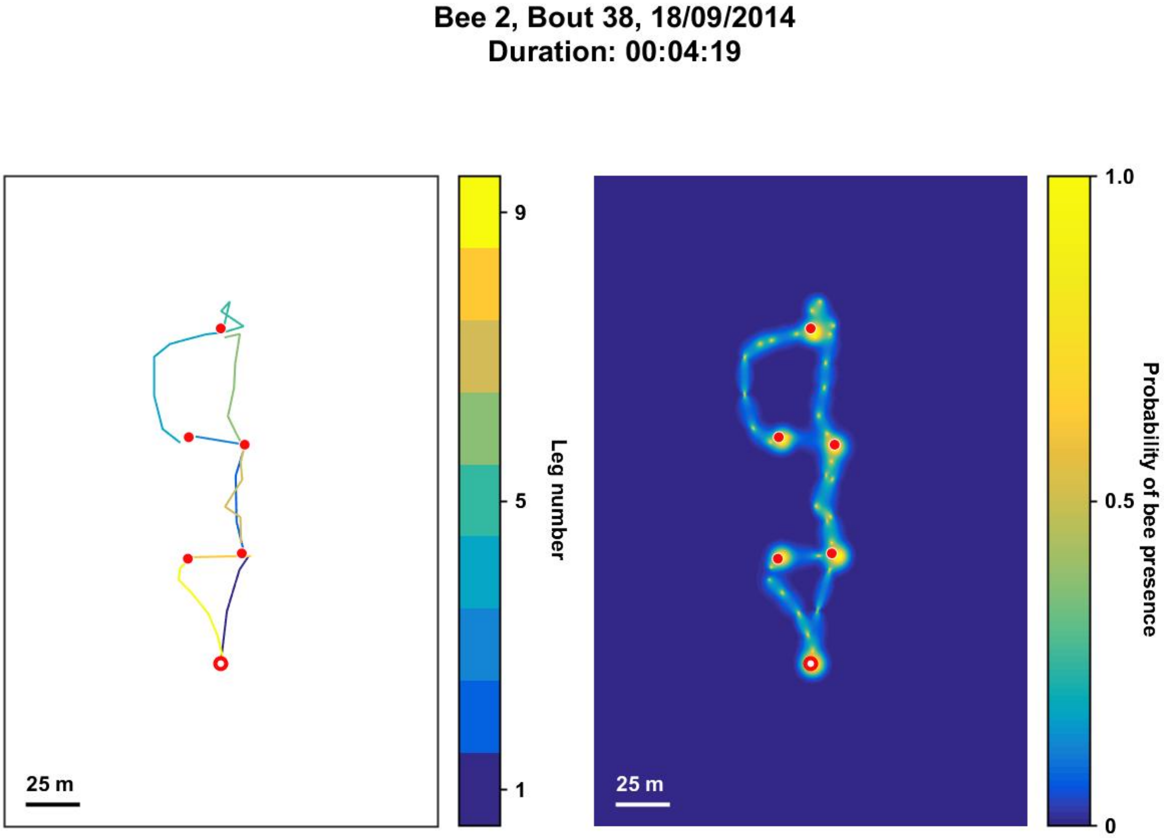

Bee 2, Bout 38, 18/09/2014
Duration: 00:04:19
25 m
9
5
1
Leg number
25 m
1.0
0.5
0
Probability of bee presence

Fig S86

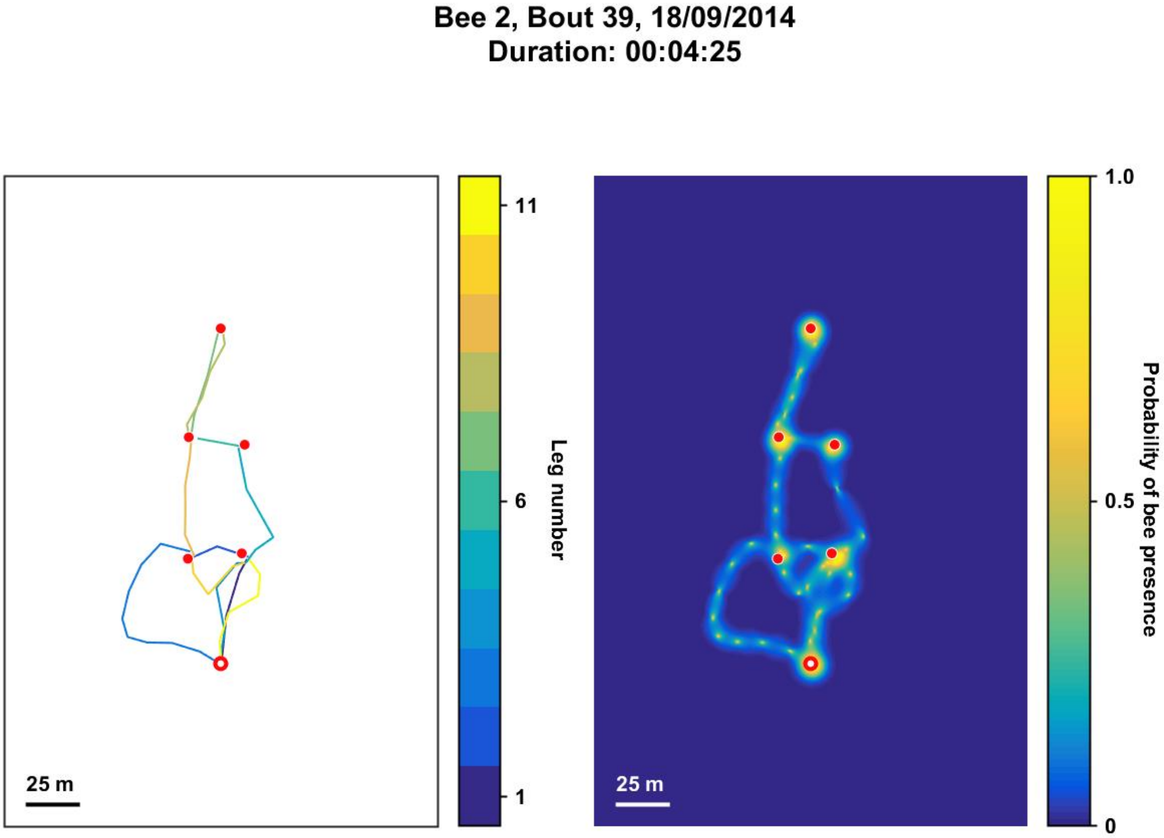

Bee 2, Bout 39, 18/09/2014
Duration: 00:04:25
25 m
Leg number
11
6
1
25 m
Probability of bee presence
1.0
0.5
0

Fig S87

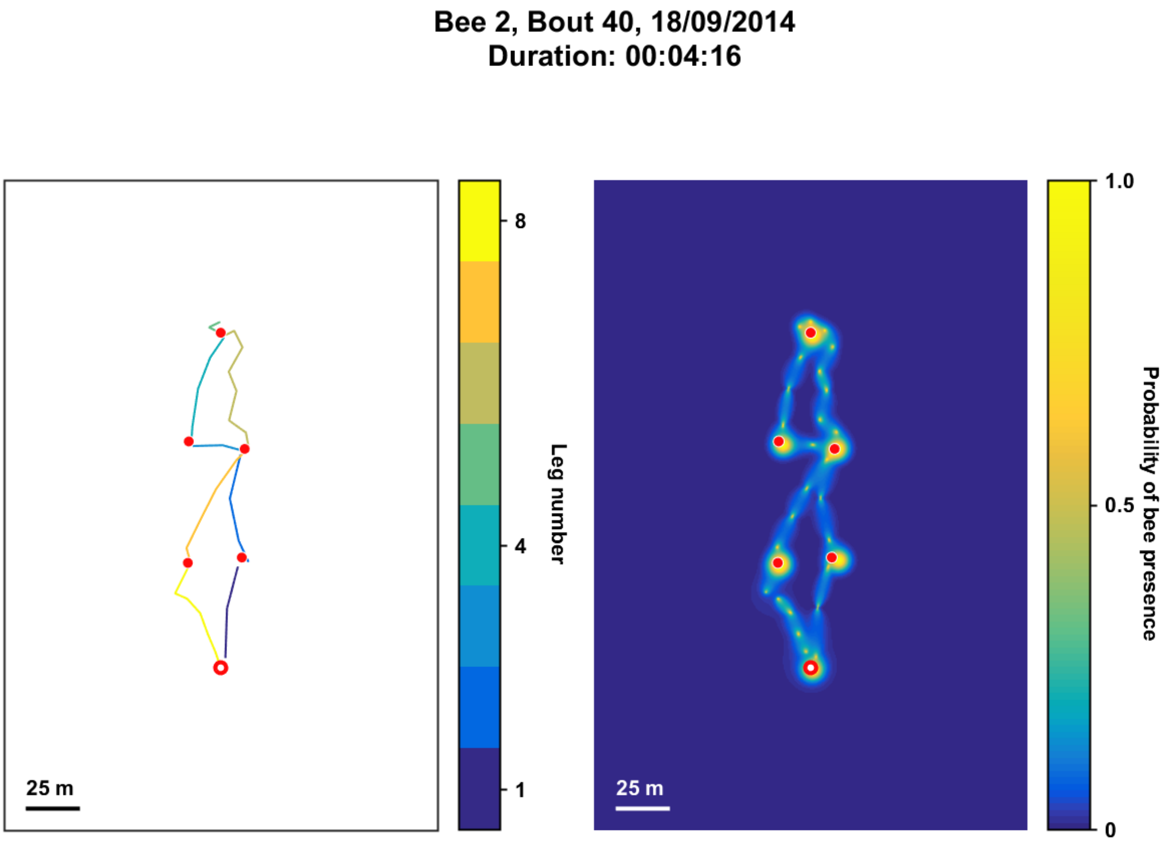
Bee 2, Bout 40, 18/09/2014
Duration: 00:04:16
25 m
8
4
1
Leg number
25 m
1.0
0.5
0
Probability of bee presence

Fig S88

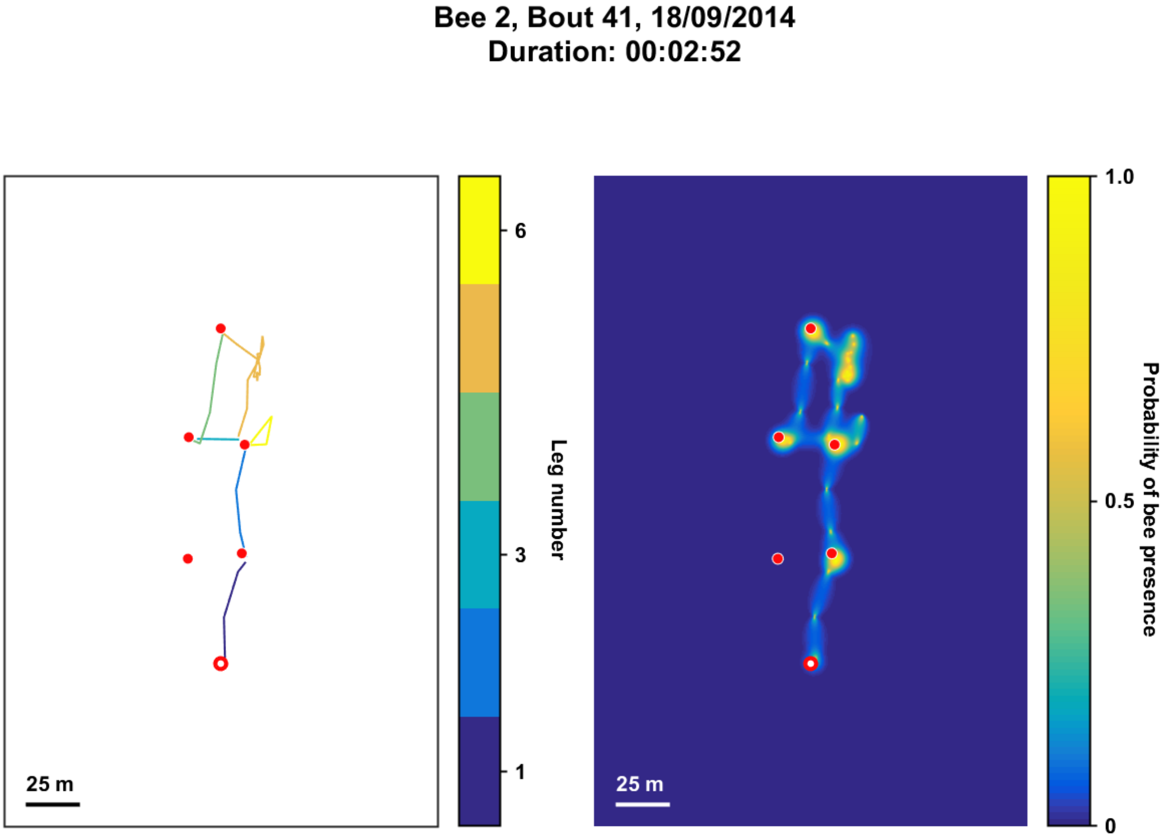

Bee 2, Bout 41, 18/09/2014
Duration: 00:02:52
25 m
6
3
1
Leg number
25 m
1.0
0.5
0
Probability of bee presence

Fig S89

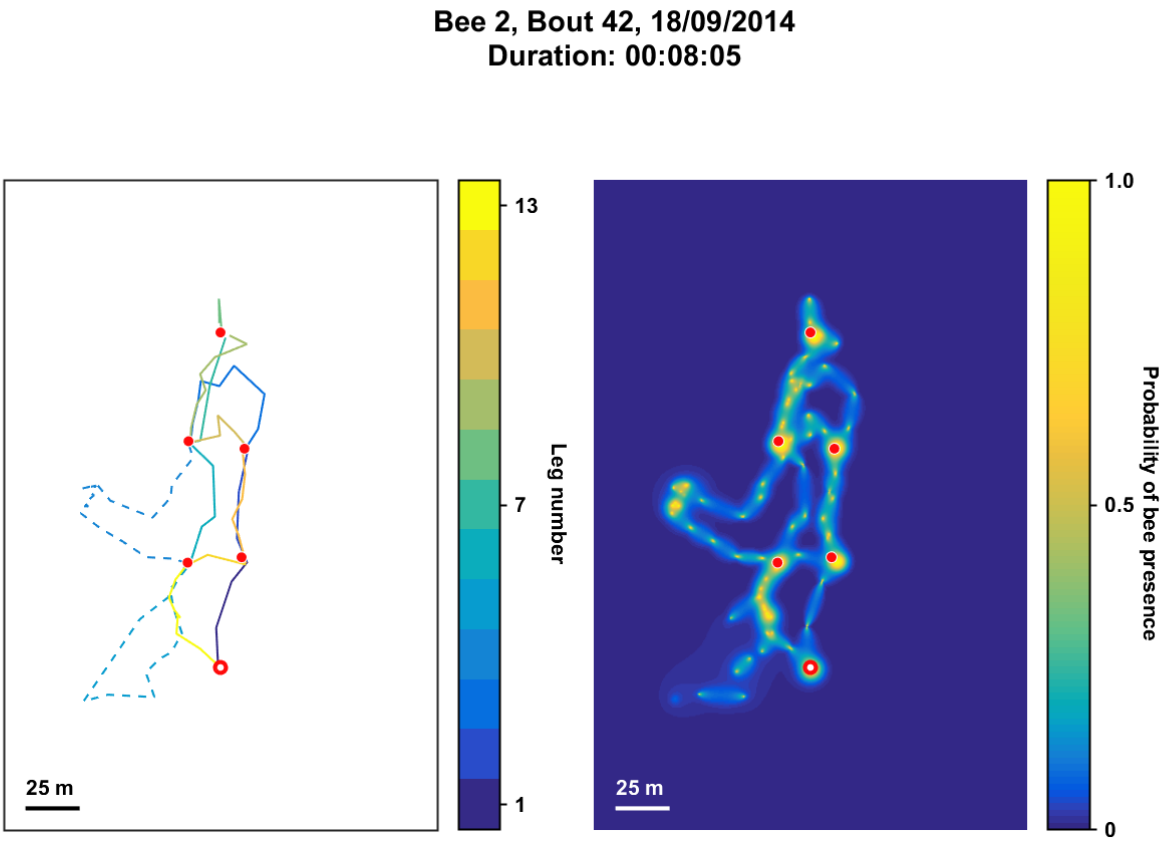
Bee 2, Bout 42, 18/09/2014
Duration: 00:08:05
25 m
13
7
1
Leg number
25 m
1.0
0.5
0
Probability of bee presence

Fig S90

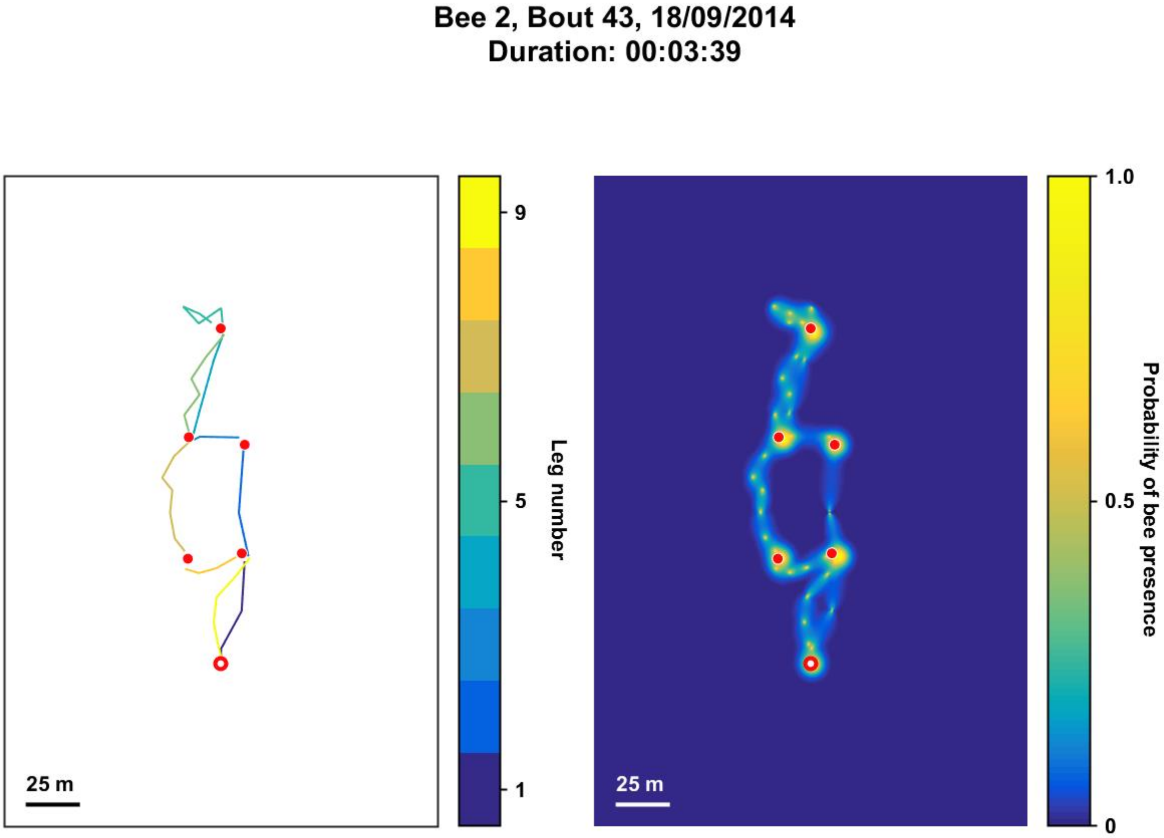

Bee 2, Bout 43, 18/09/2014
Duration: 00:03:39
25 m
Leg number
9
5
1
25 m
Probability of bee presence
1.0
0.5
0

Fig S91

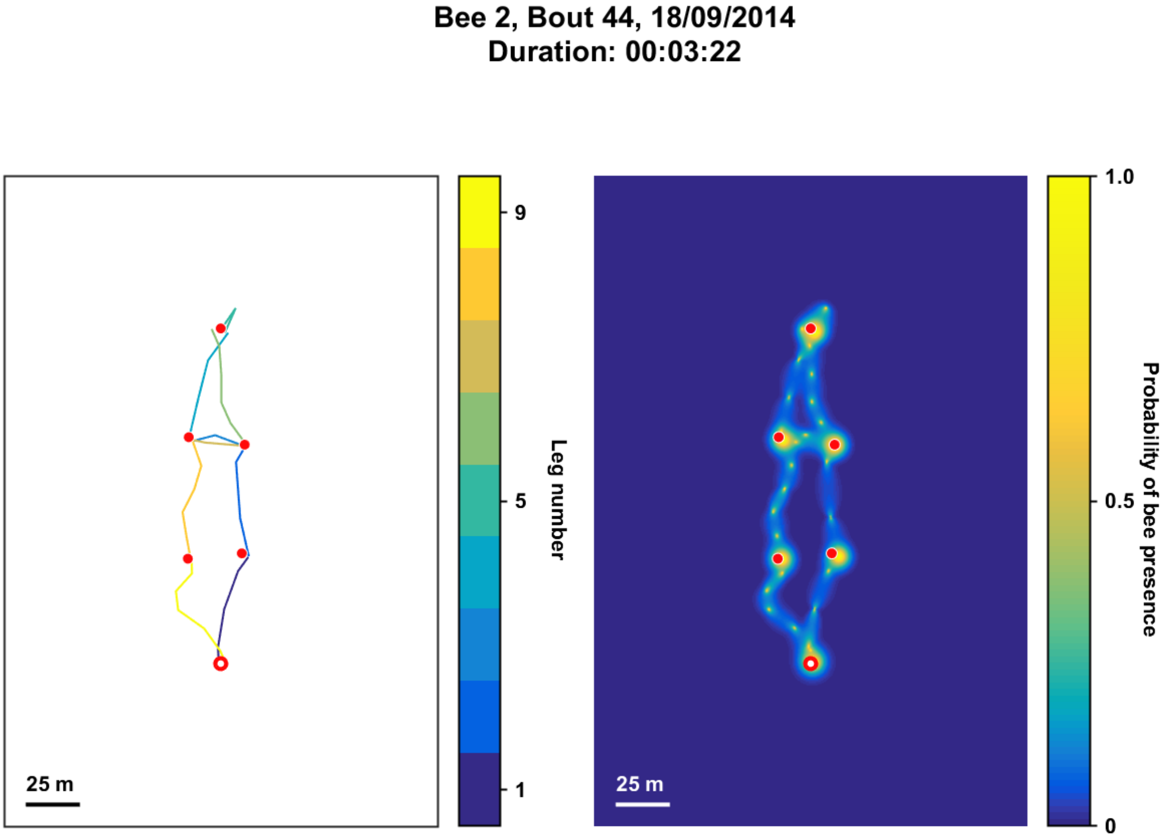

Bee 2, Bout 44, 18/09/2014
Duration: 00:03:22
25 m
Leg number
9
5
1
25 m
Probability of bee presence
1.0
0.5
0

Fig S92

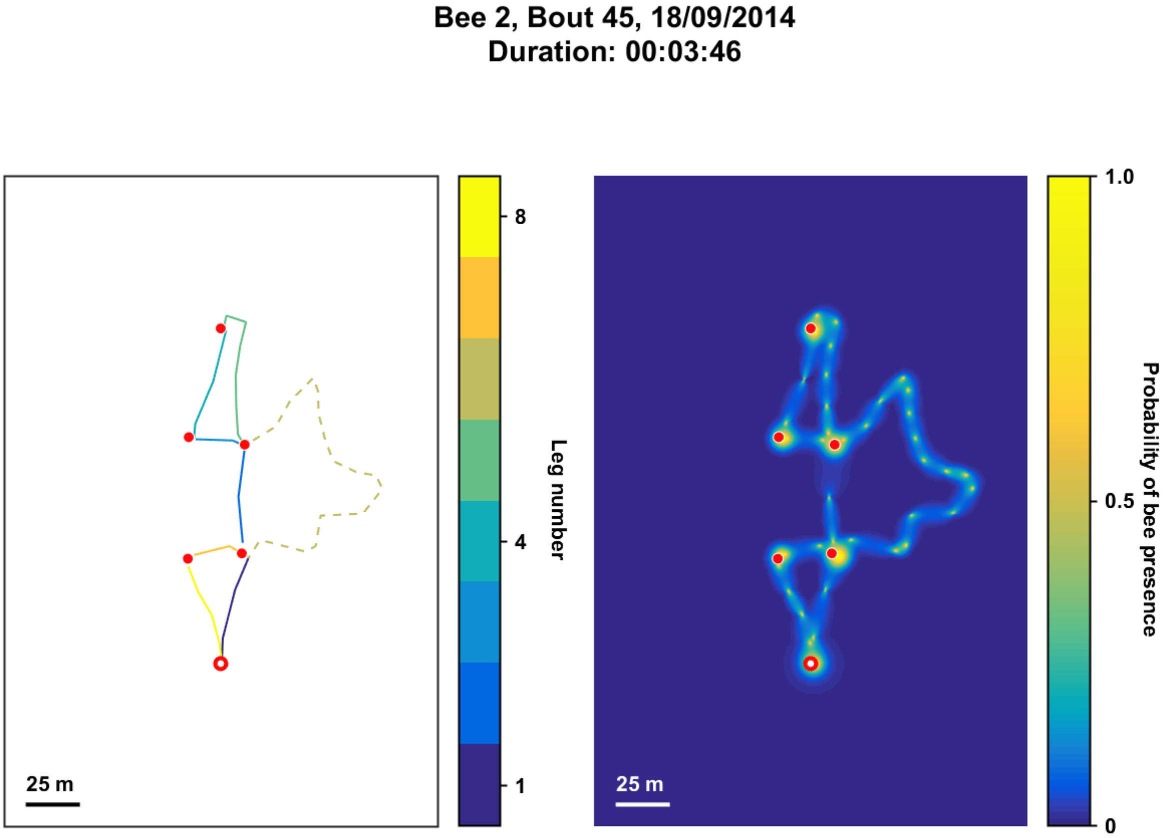
Bee 2, Bout 45, 18/09/2014
Duration: 00:03:46
25 m
8
4
1
Leg number
25 m
1.0
0.5
0
Probability of bee presence

Fig S93

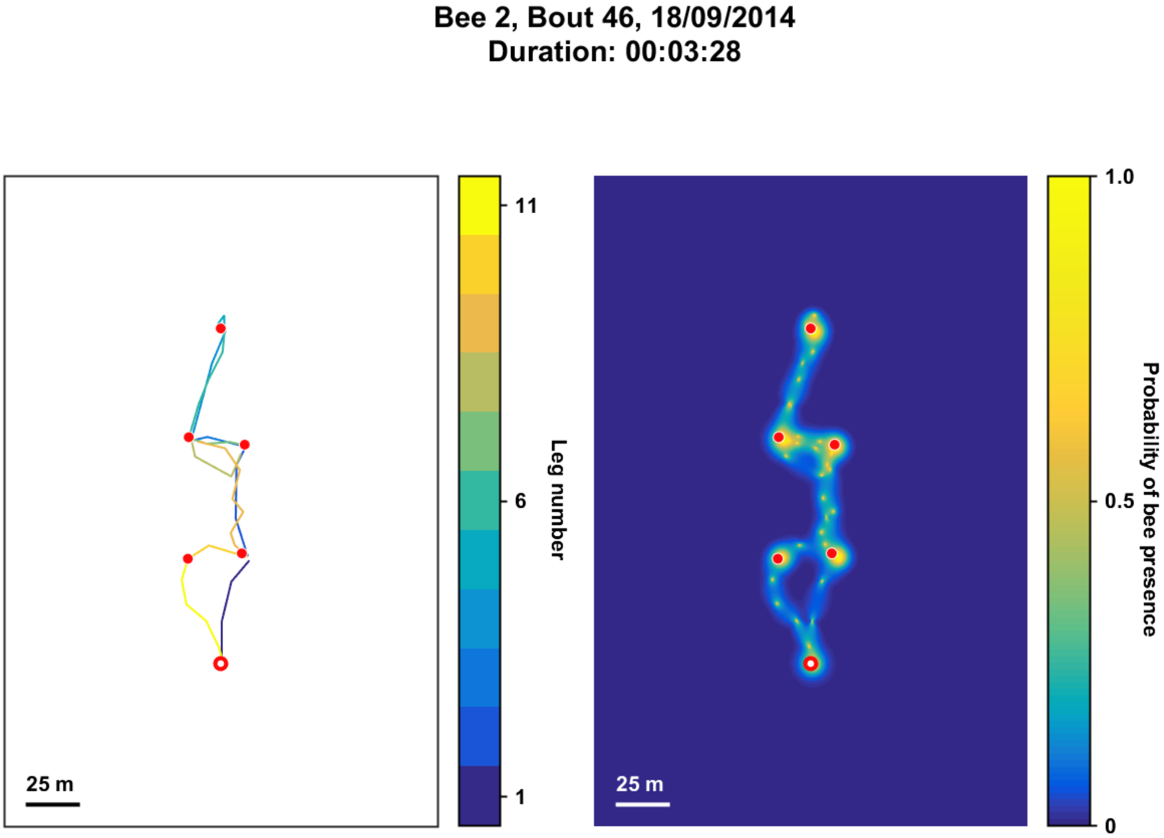

Bee 2, Bout 46, 18/09/2014
Duration: 00:03:28
25 m
11
6
1
Leg number
25 m
1.0
0.5
0
Probability of bee presence

Fig S94

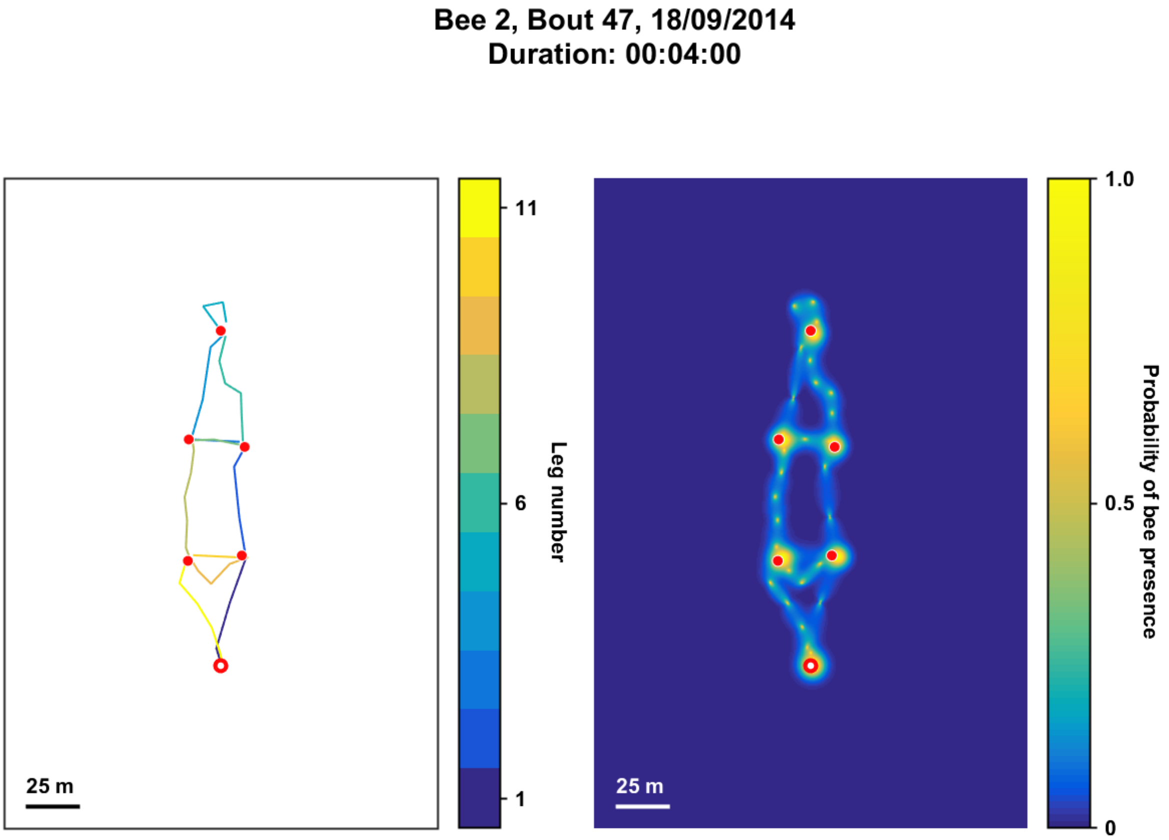
Bee 2, Bout 47, 18/09/2014
Duration: 00:04:00
25 m
11
6
1
Leg number
25 m
1.0
0.5
0
Probability of bee presence

Fig S95

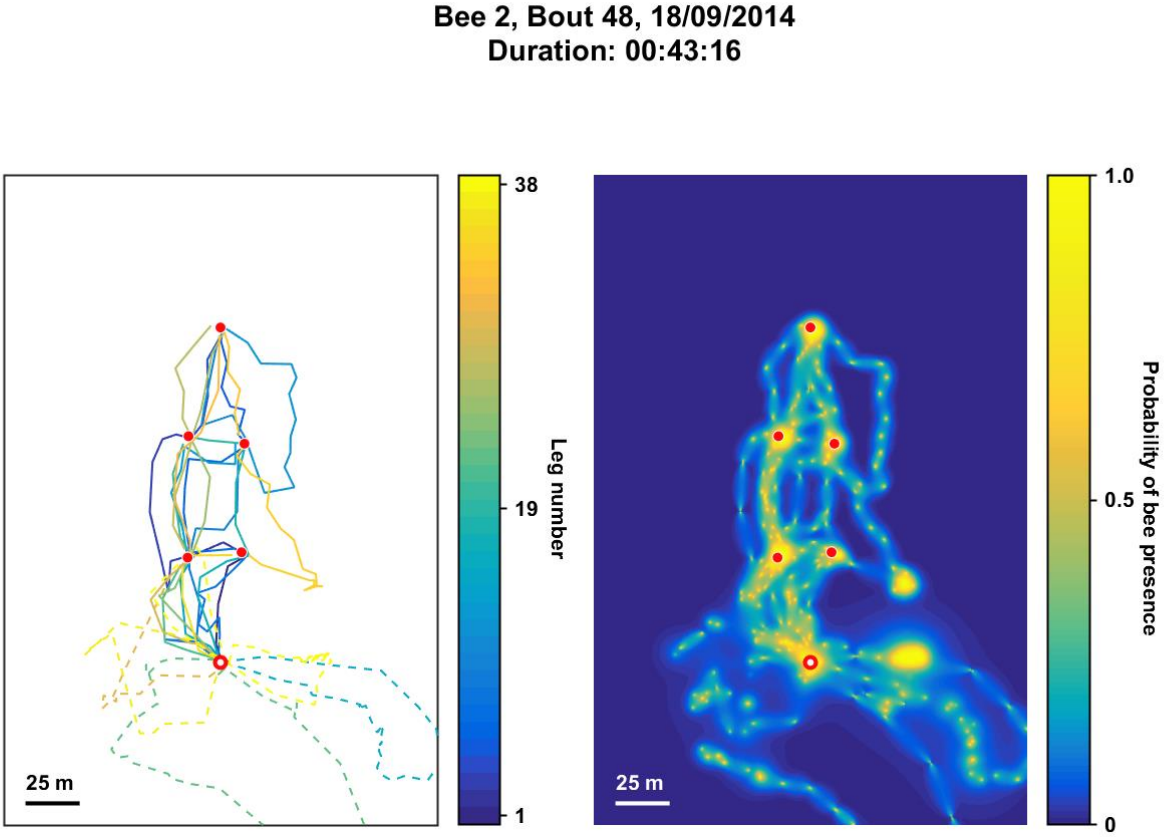
Bee 2, Bout 48, 18/09/2014
Duration: 00:43:16
25 m
38
19
1
Leg number
25 m
1.0
0.5
0
Probability of bee presence

Fig S96

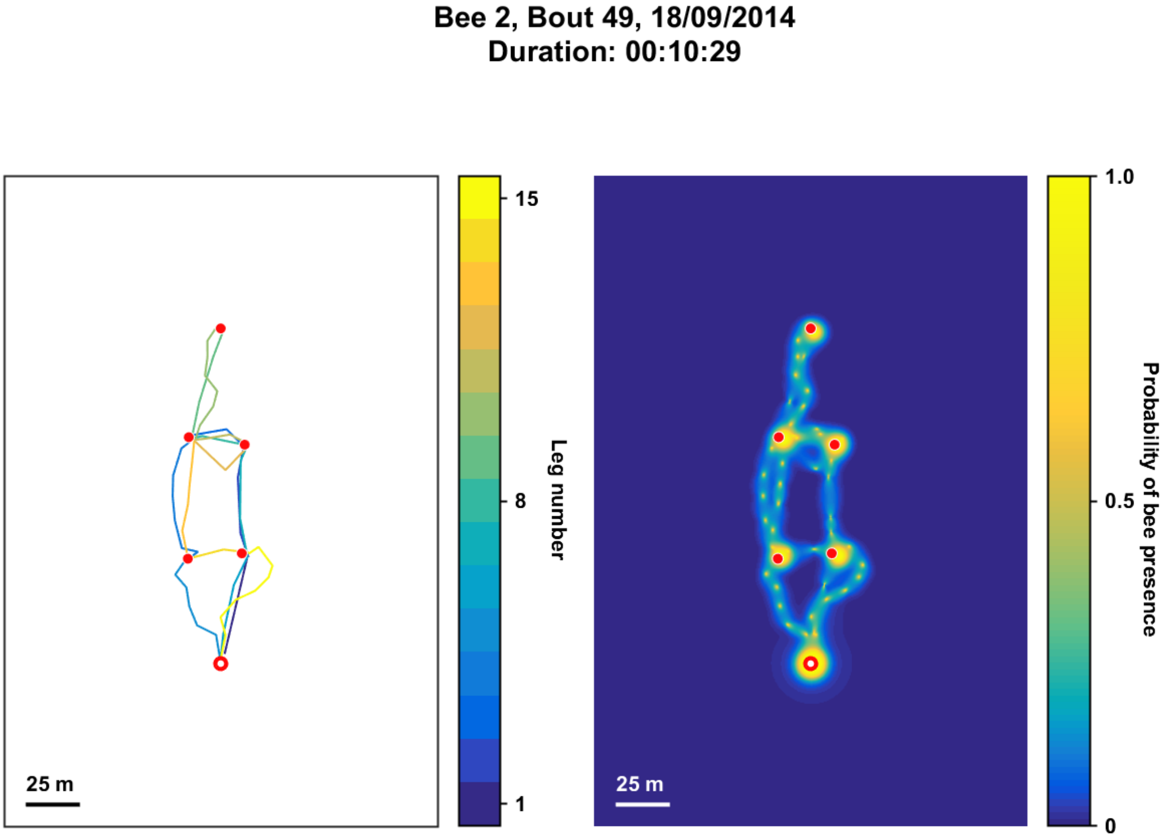
Bee 2, Bout 49, 18/09/2014
Duration: 00:10:29
25 m
15
8
1
Leg number
25 m
1.0
0.5
0
Probability of bee presence

Fig S97

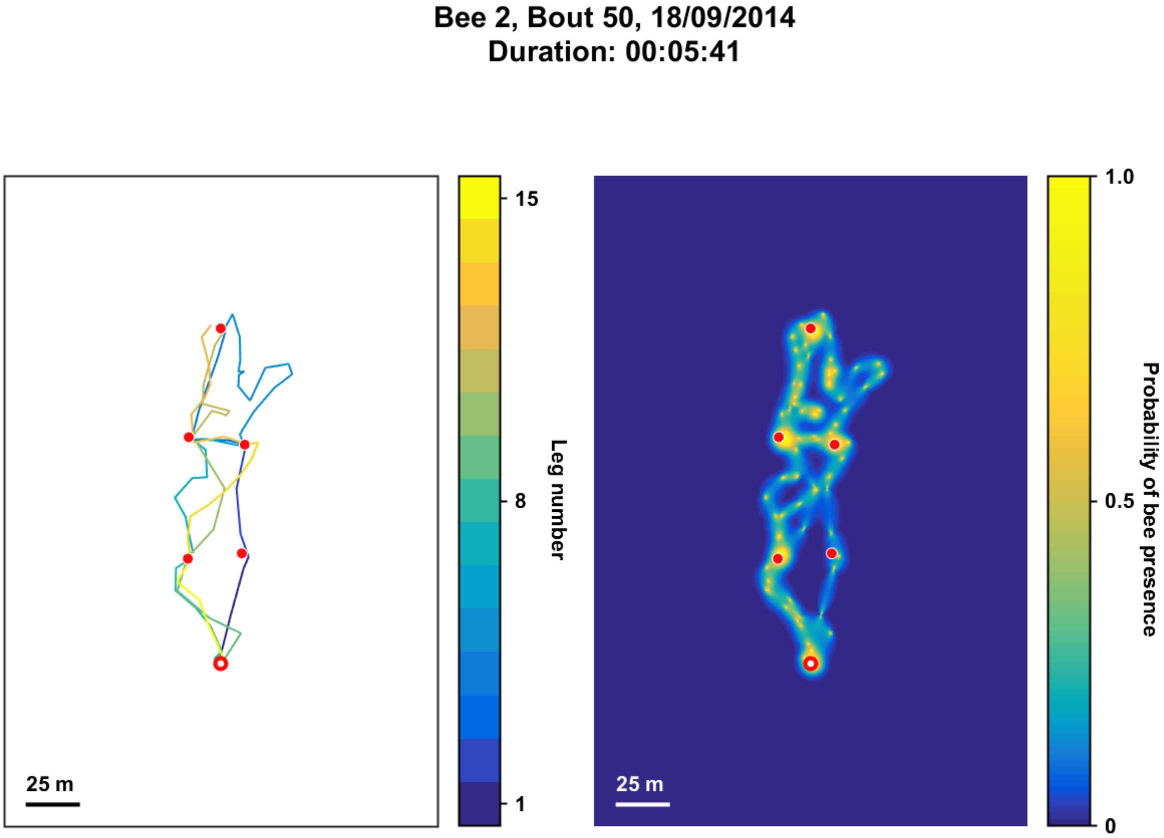

Bee 2, Bout 50, 18/09/2014
Duration: 00:05:41
15
8
1
Leg number
25 m
1.0
0.5
0
Probability of bee presence
25 m

Fig S98

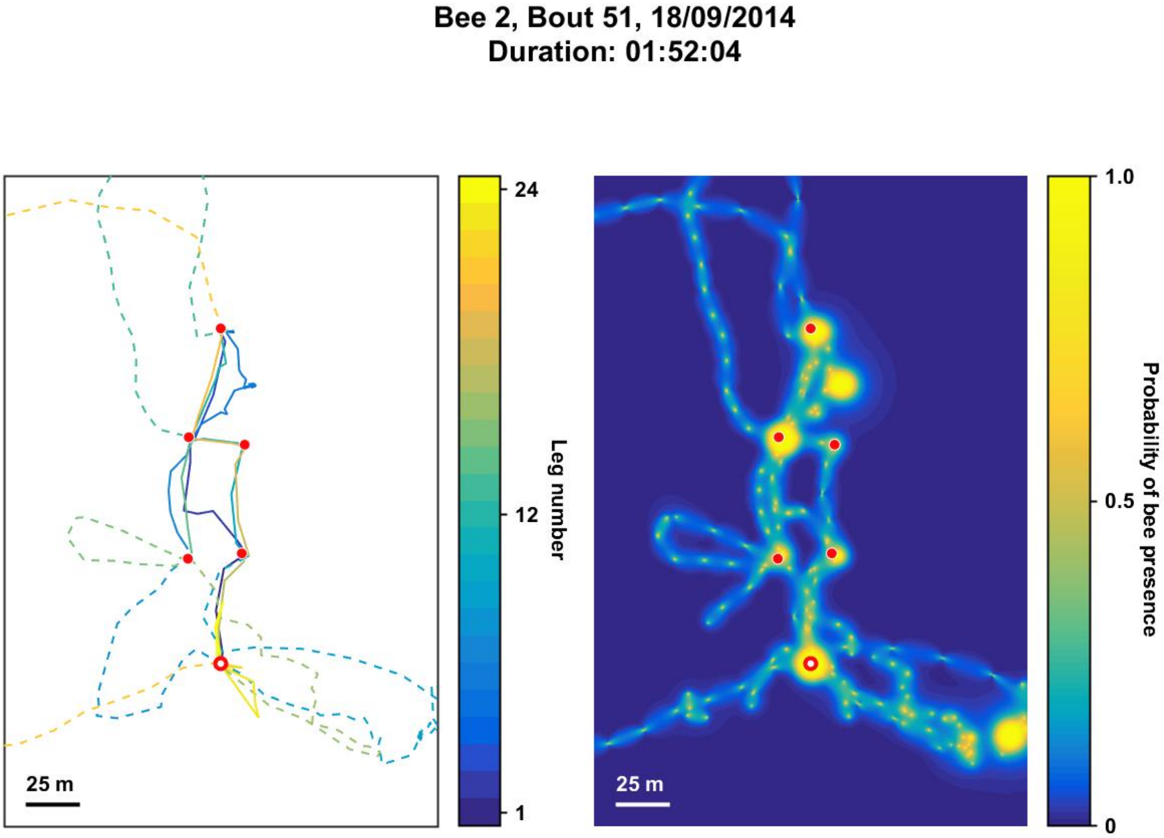

Bee 2, Bout 51, 18/09/2014
Duration: 01:52:04
25 m
24
12
1
Leg number
25 m
1.0
0.5
0
Probability of bee presence

Fig S99

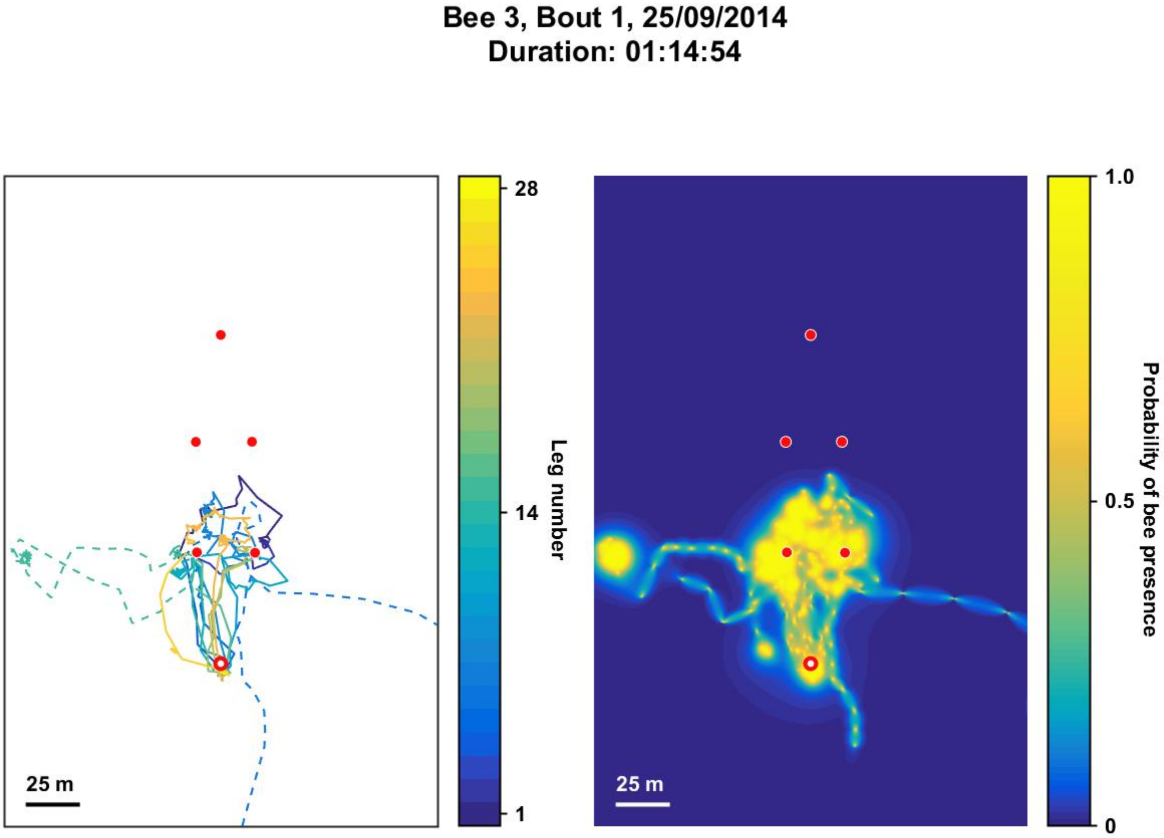

Bee 3, Bout 1, 25/09/2014
Duration: 01:14:54
25 m
28
14
1
Leg number
25 m
1.0
0.5
0
Probability of bee presence

Fig S100

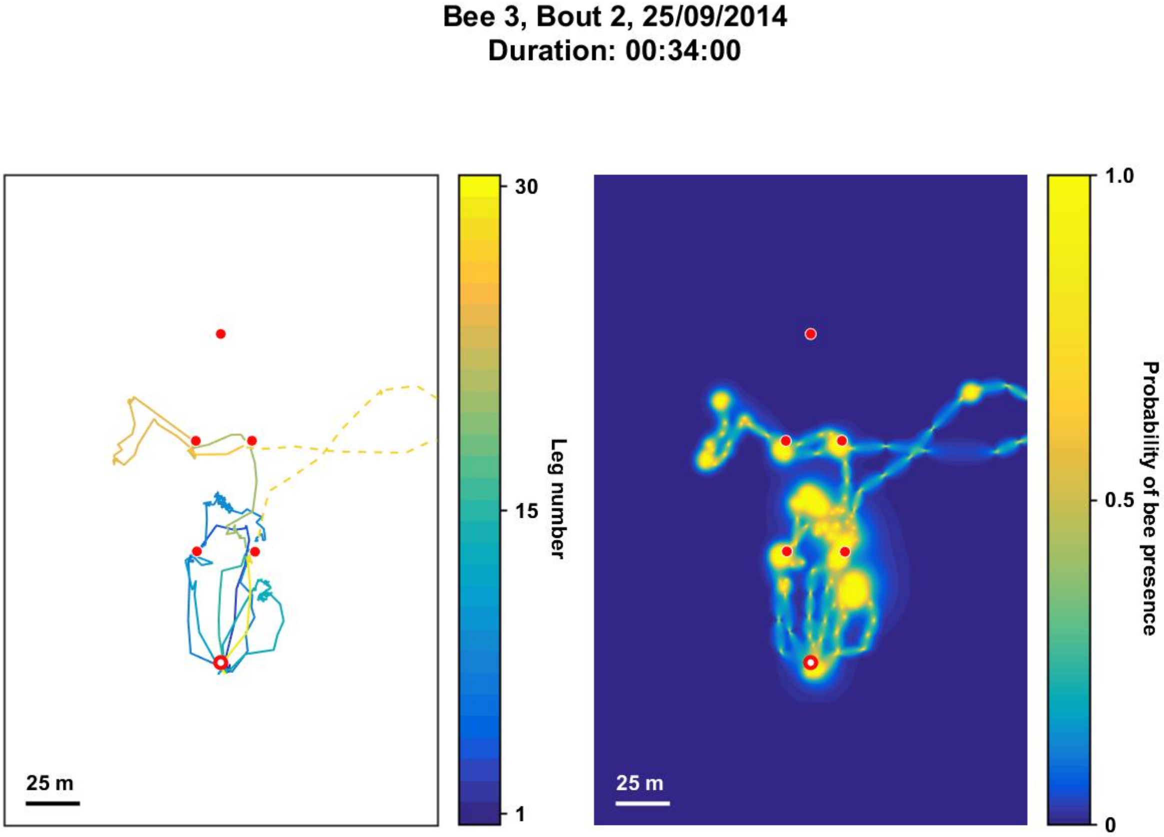
Bee 3, Bout 2, 25/09/2014
Duration: 00:34:00
25 m
30
15
1
Leg number
25 m
1.0
0.5
0
Probability of bee presence

Fig S101

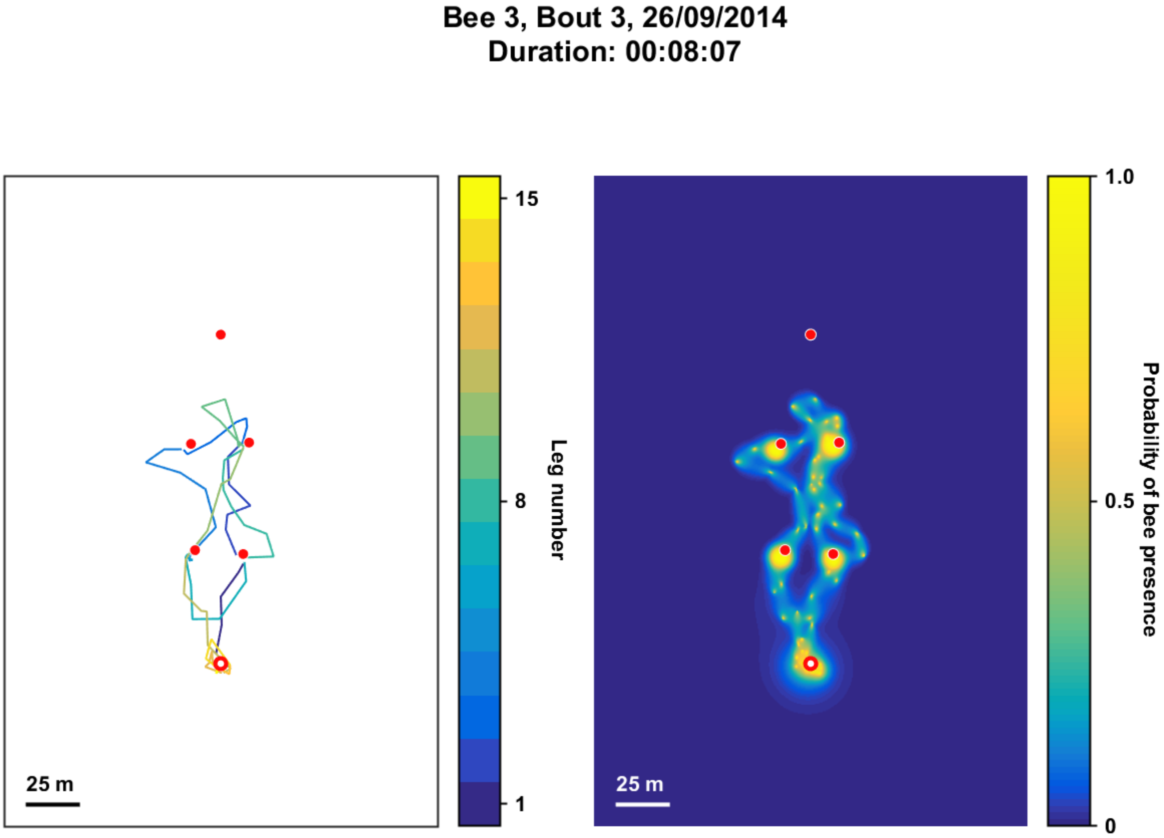

Bee 3, Bout 3, 26/09/2014
Duration: 00:08:07
25 m
Leg number
15
8
1
25 m
Probability of bee presence
1.0
0.5
0

Fig S102

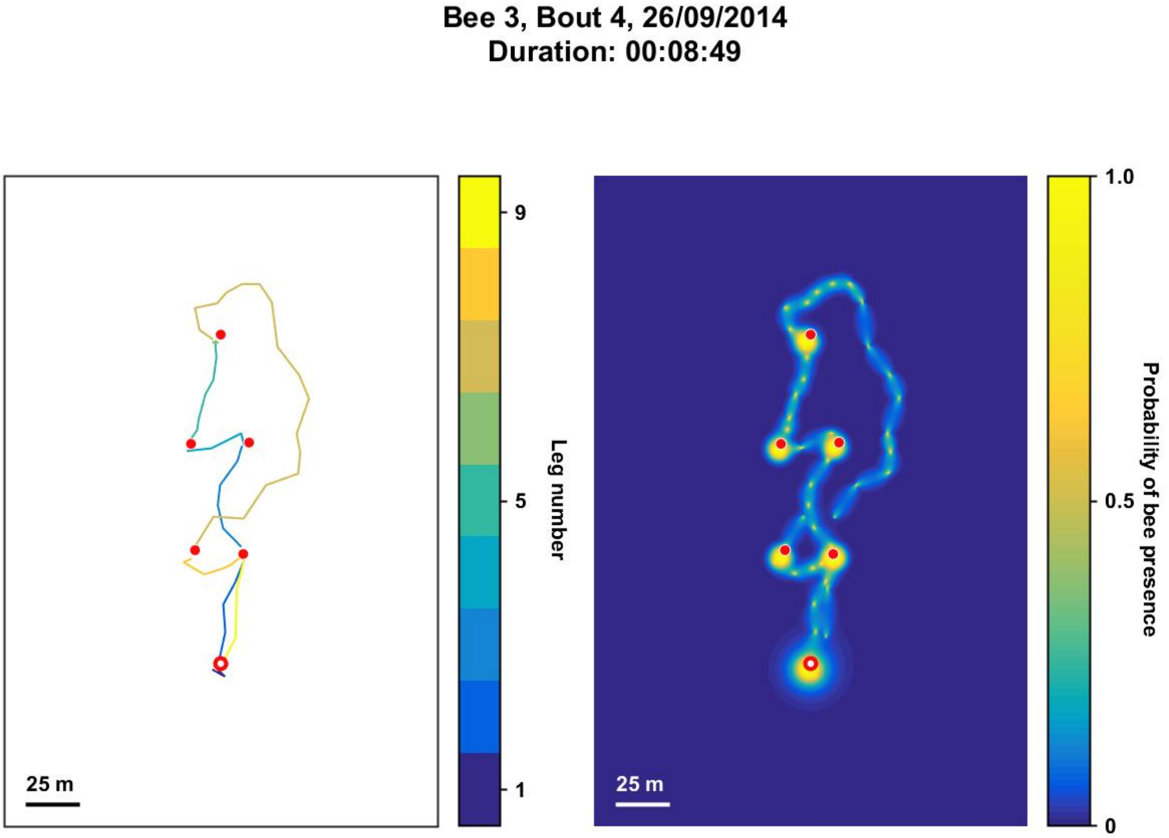
Bee 3, Bout 4, 26/09/2014
Duration: 00:08:49
25 m
9
5
1
Leg number
25 m
1.0
0.5
0
Probability of bee presence

Fig S103

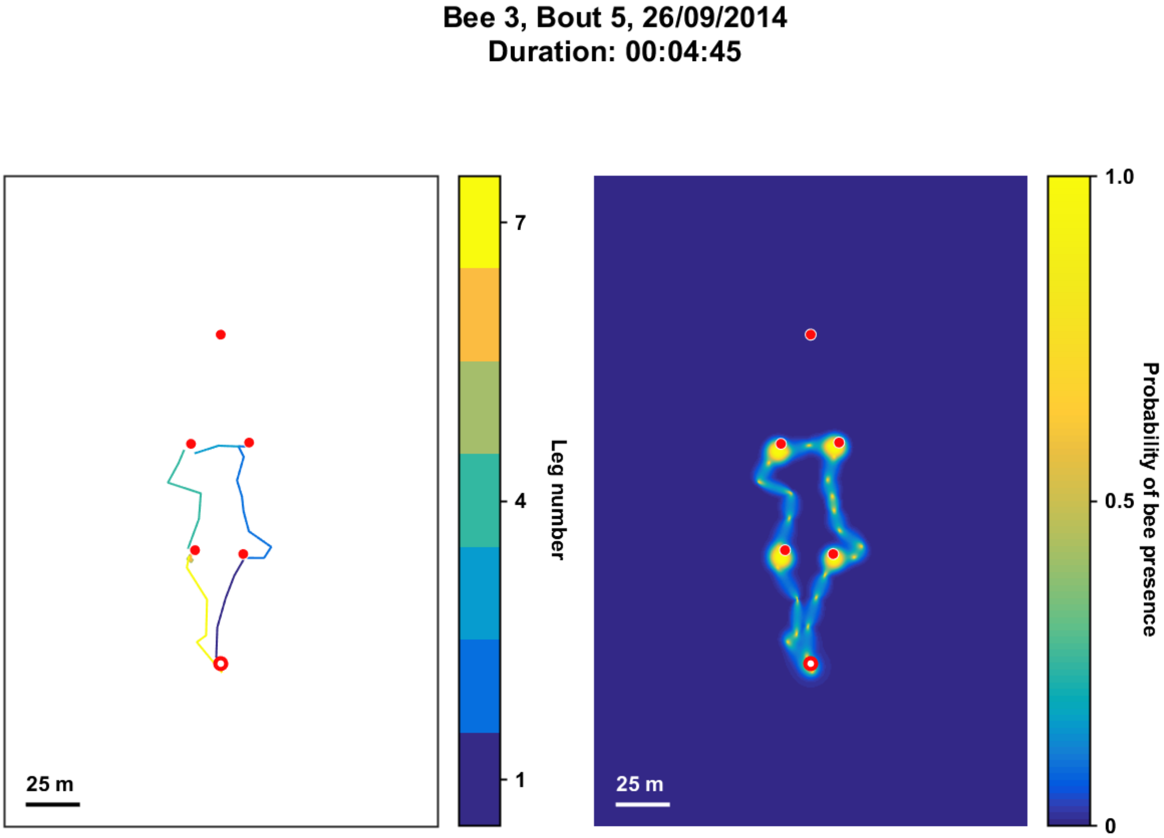

Bee 3, Bout 5, 26/09/2014
Duration: 00:04:45
25 m
7
4
1
Leg number
25 m
1.0
0.5
0
Probability of bee presence

Fig S104

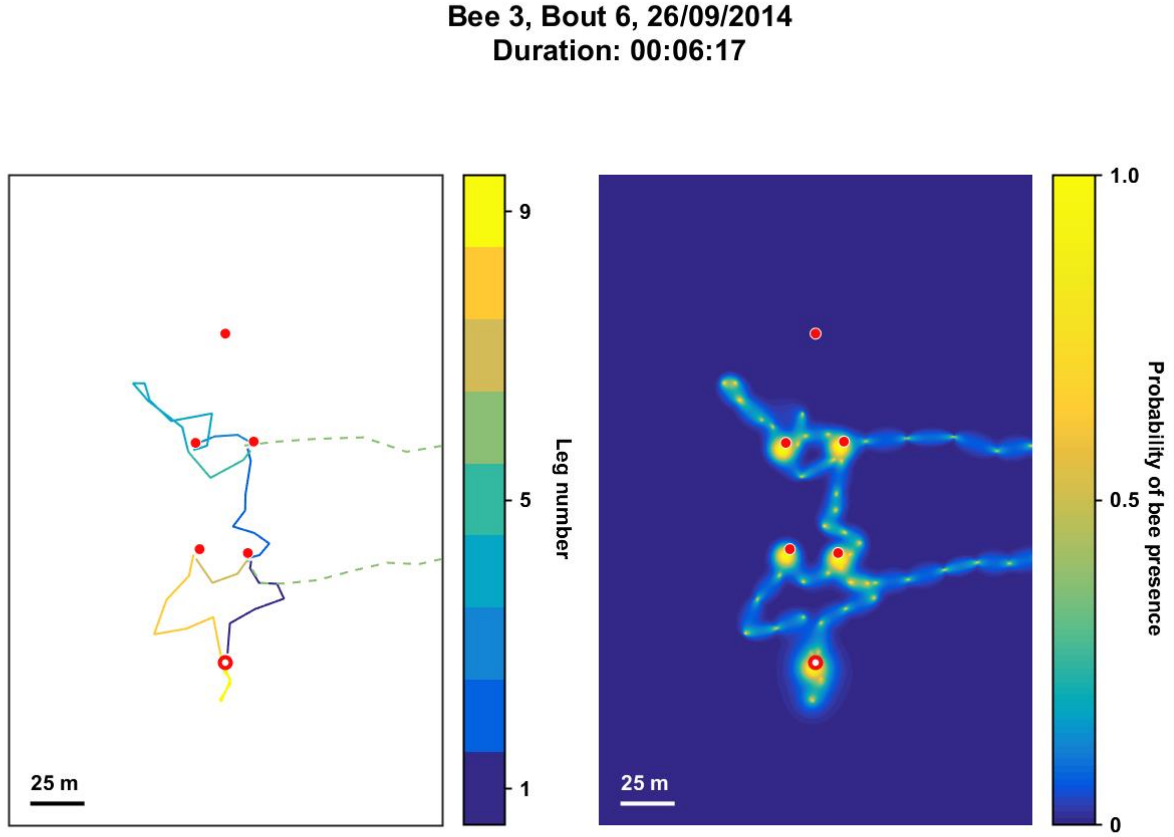

Bee 3, Bout 6, 26/09/2014
Duration: 00:06:17
25 m
9
5
1
Leg number
25 m
1.0
0.5
0
Probability of bee presence

Fig S105

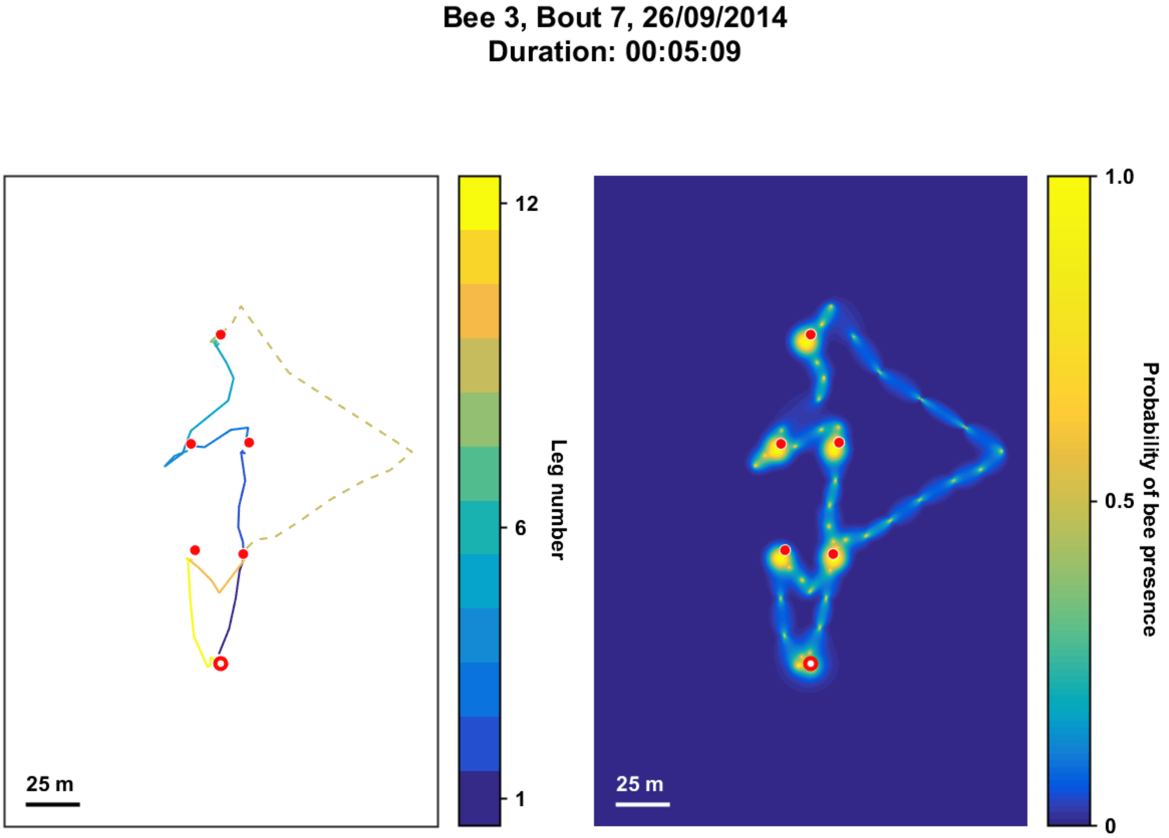

Bee 3, Bout 7, 26/09/2014
Duration: 00:05:09
25 m
12
6
1
Leg number
25 m
1.0
0.5
0
Probability of bee presence

Fig S106

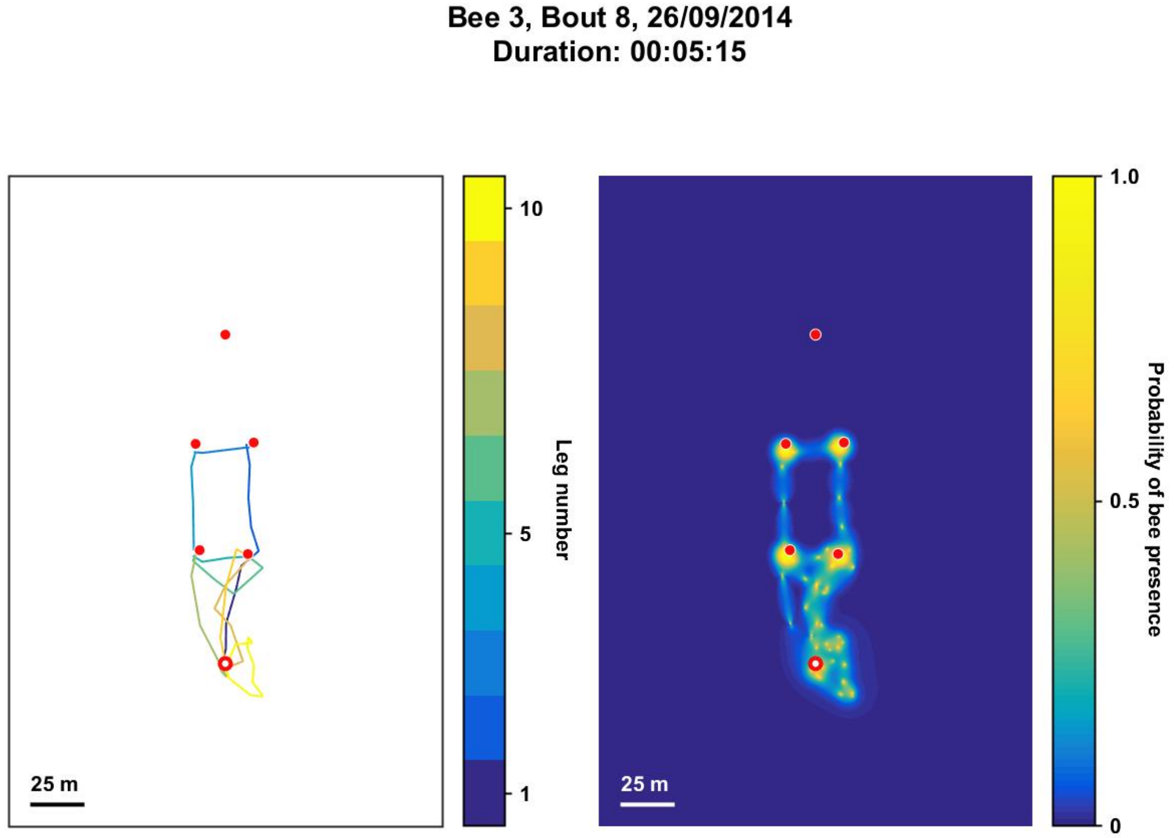

Bee 3, Bout 8, 26/09/2014
Duration: 00:05:15
10
5
1
Leg number
25 m
25 m
1.0
0.5
0
Probability of bee presence

Fig S107

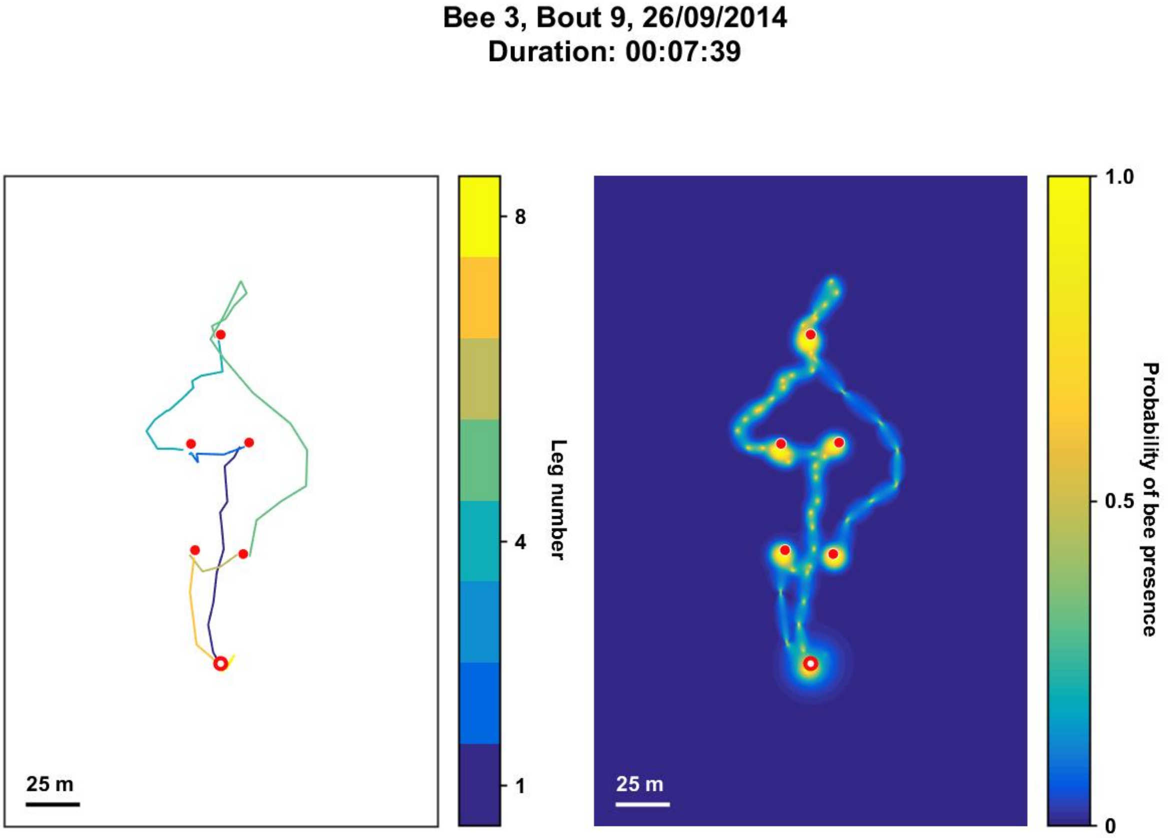

Bee 3, Bout 9, 26/09/2014
Duration: 00:07:39
25 m
Leg number
8
4
1
25 m
Probability of bee presence
1.0
0.5
0

Fig S108

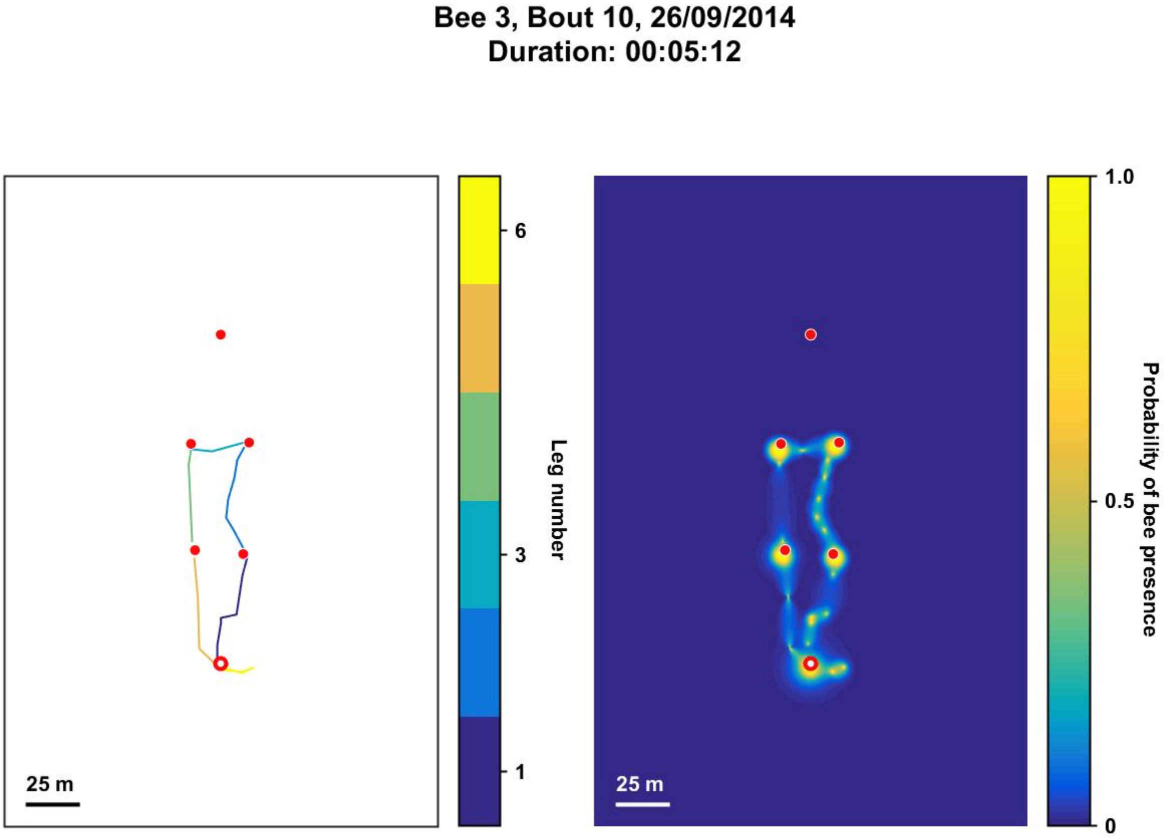
Bee 3, Bout 10, 26/09/2014
Duration: 00:05:12
25 m
Leg number
6
3
1
25 m
Probability of bee presence
1.0
0.5
0

Fig S109

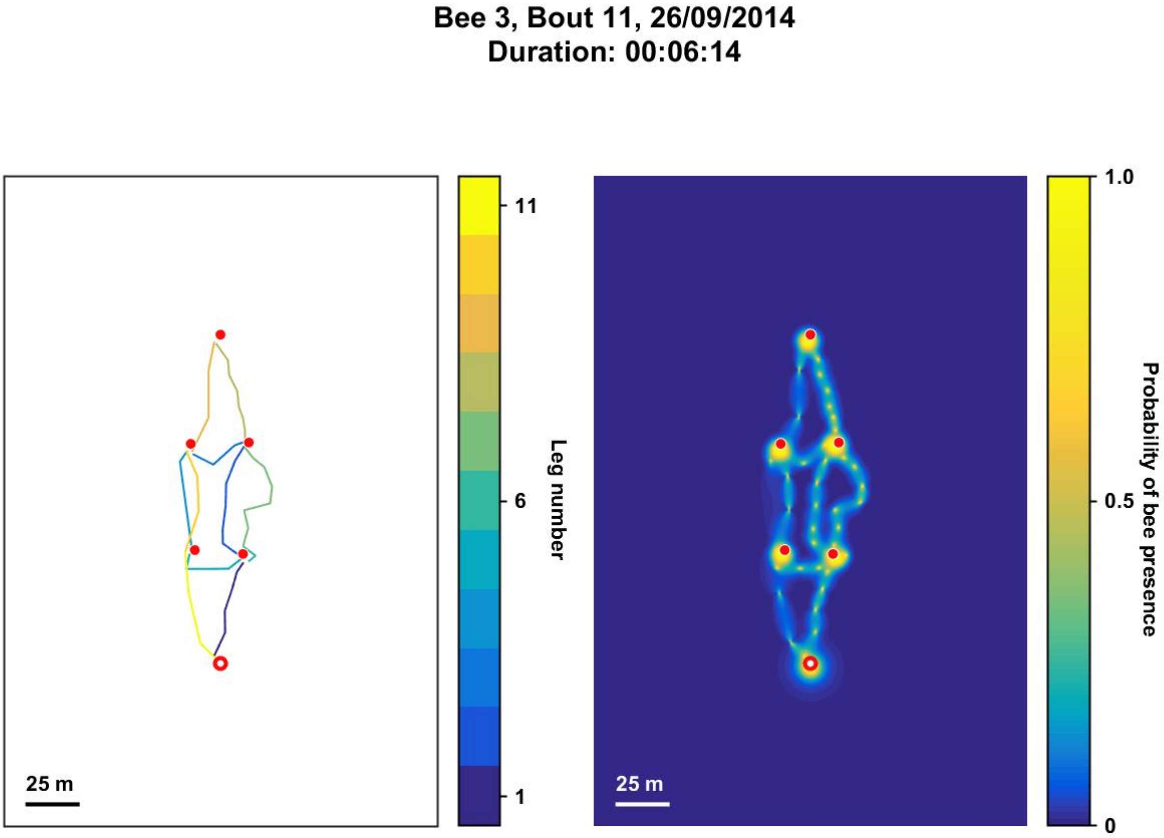
Bee 3, Bout 11, 26/09/2014
Duration: 00:06:14
25 m
11
6
1
Leg number
25 m
1.0
0.5
0
Probability of bee presence

Fig S110

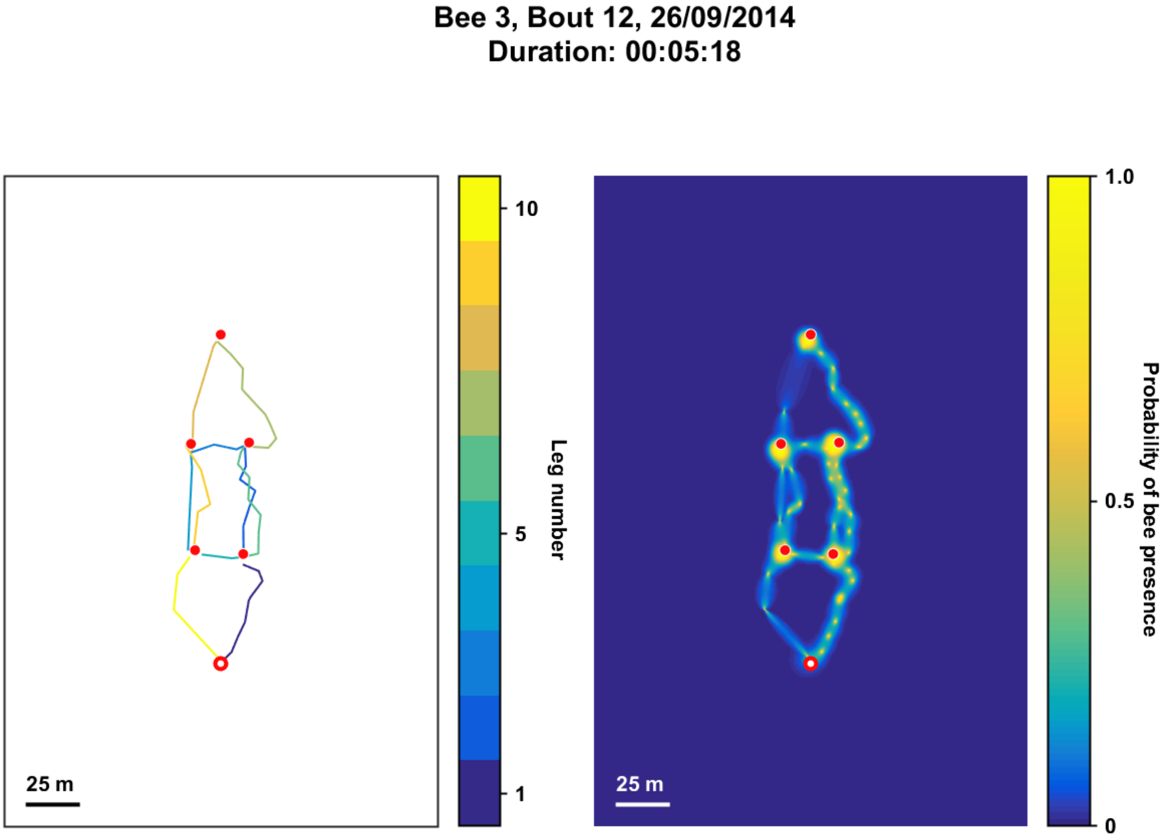

Bee 3, Bout 12, 26/09/2014
Duration: 00:05:18
25 m
10
5
1
Leg number
25 m
1.0
0.5
0
Probability of bee presence

Fig S111

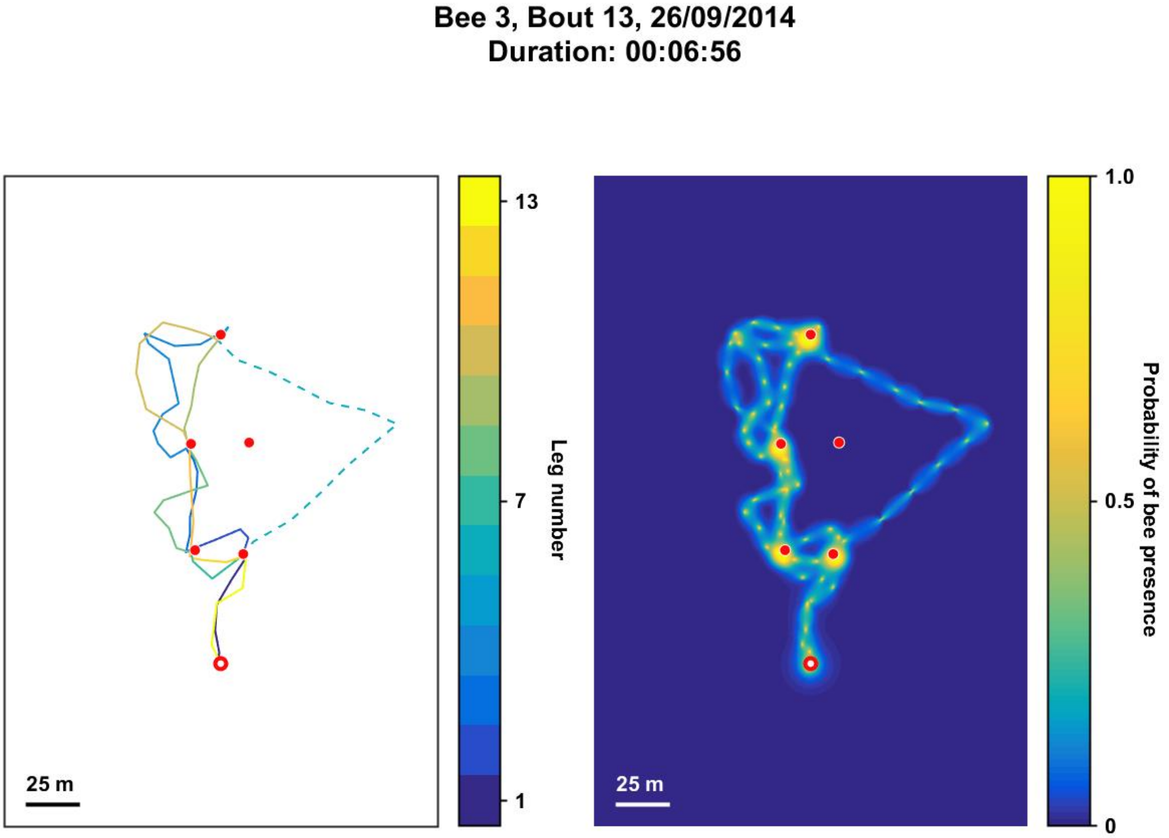

Bee 3, Bout 13, 26/09/2014
Duration: 00:06:56
13
7
1
Leg number
25 m
1.0
0.5
0
Probability of bee presence
25 m

Fig S112

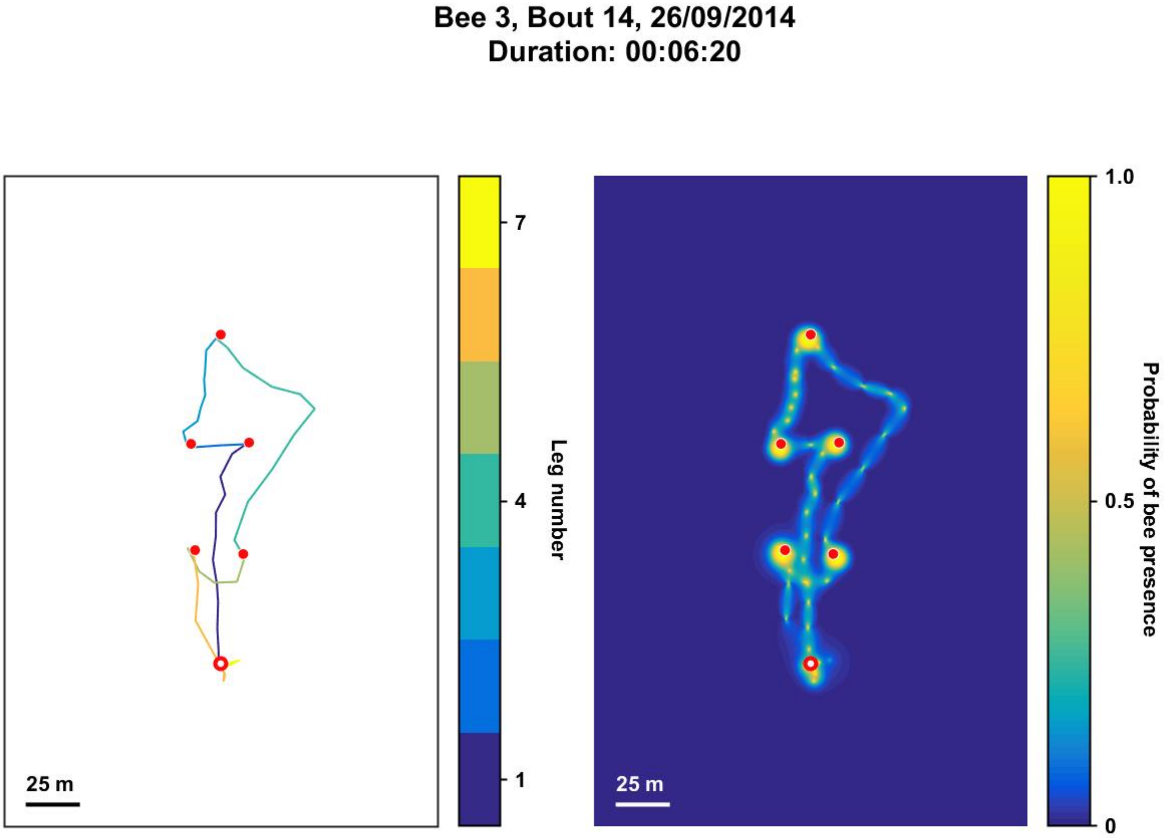
Bee 3, Bout 14, 26/09/2014
Duration: 00:06:20
25 m
Leg number
1
4
7
25 m
Probability of bee presence
0
0.5
1.0

Fig S113

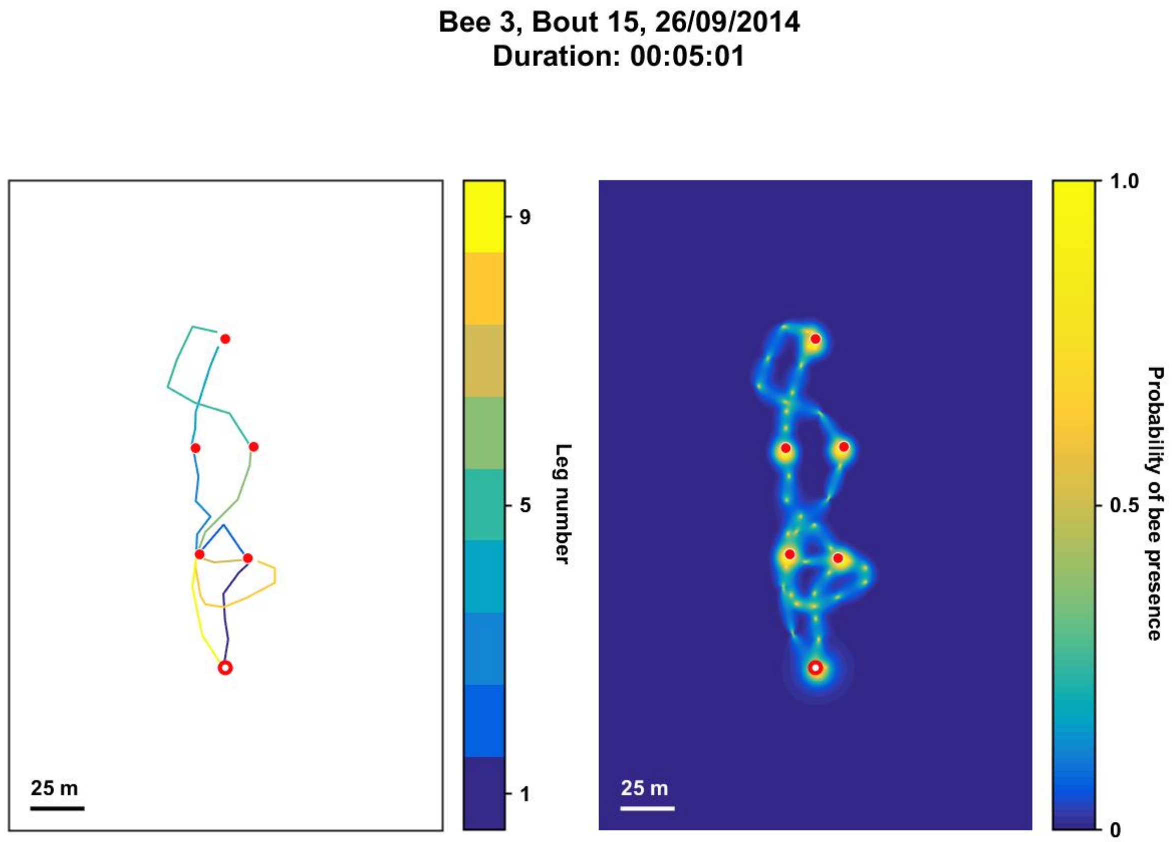
Bee 3, Bout 15, 26/09/2014
Duration: 00:05:01
25 m
Leg number
9
5
1
25 m
Probability of bee presence
1.0
0.5
0

Fig S114

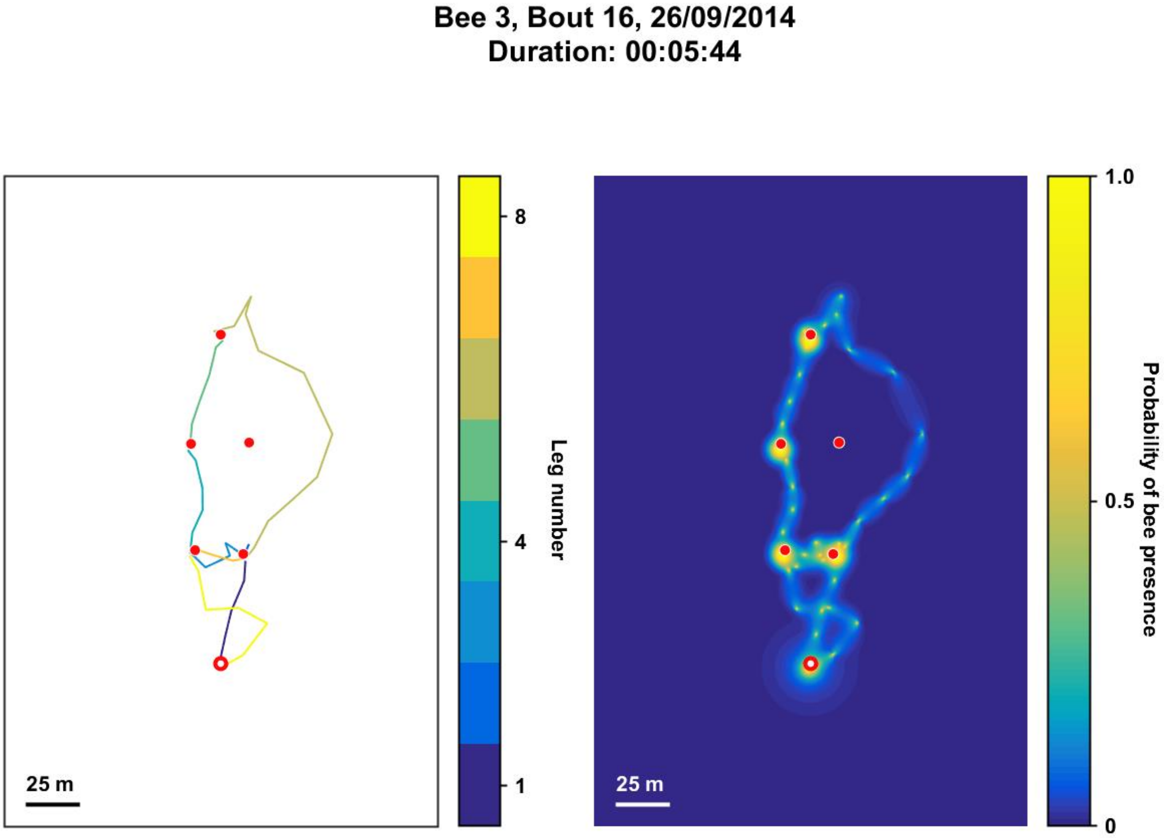

Bee 3, Bout 16, 26/09/2014
Duration: 00:05:44
25 m
Leg number
8
4
1
25 m
Probability of bee presence
1.0
0.5
0

Fig S115

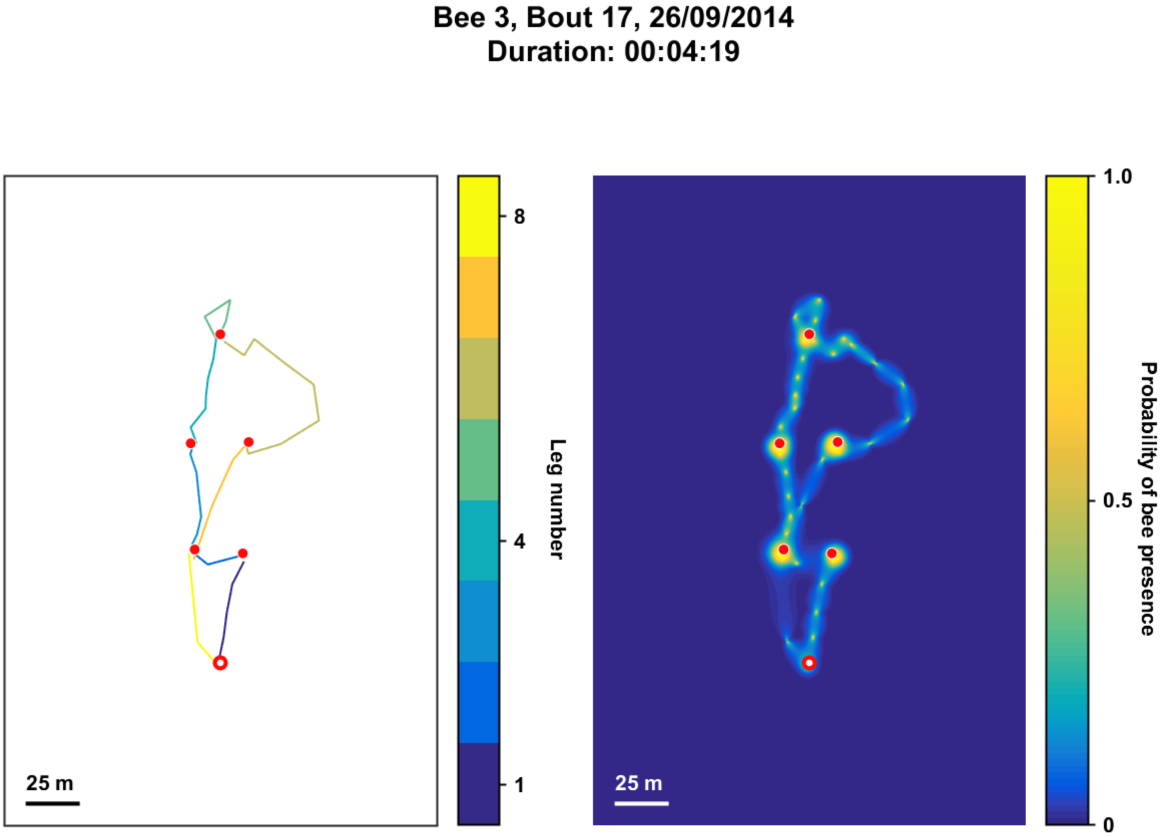
Bee 3, Bout 17, 26/09/2014
Duration: 00:04:19
25 m
8
4
1
Leg number
25 m
1.0
0.5
0
Probability of bee presence

Fig S116

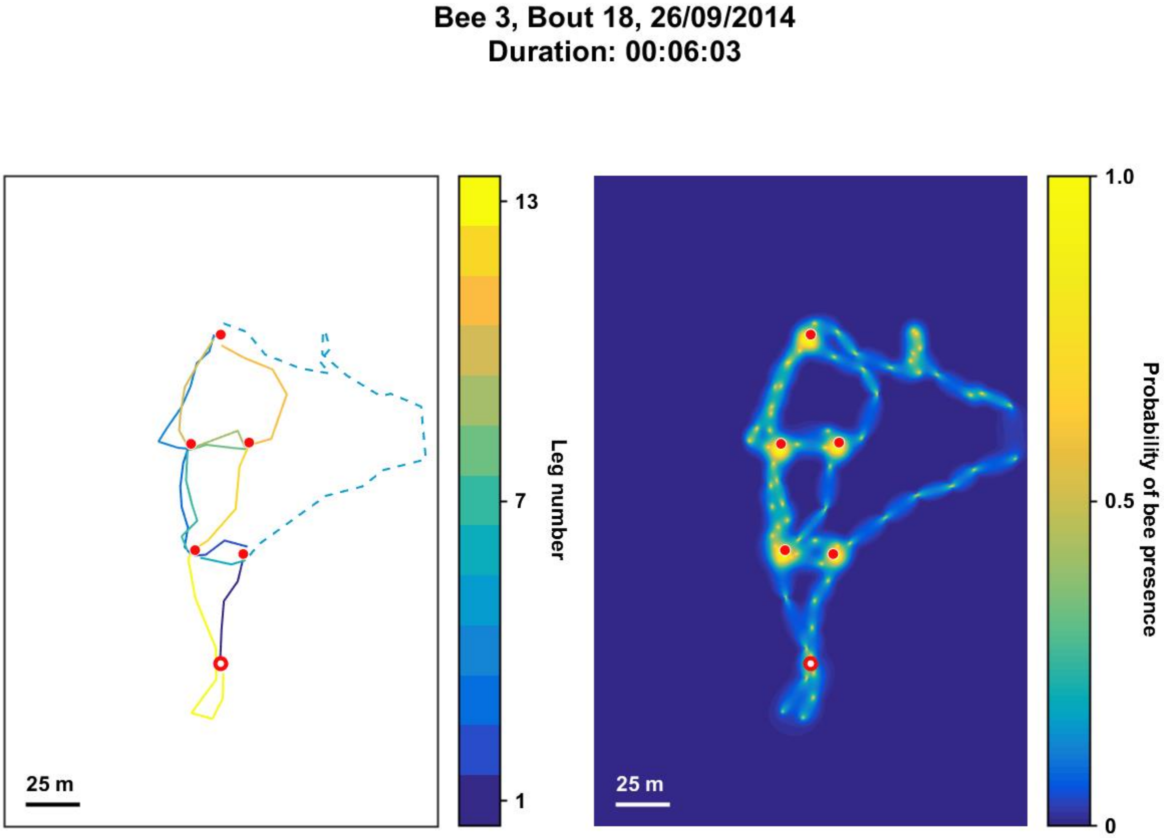

Bee 3, Bout 18, 26/09/2014
Duration: 00:06:03
25 m
13
7
1
Leg number
25 m
1.0
0.5
0
Probability of bee presence

Fig S117

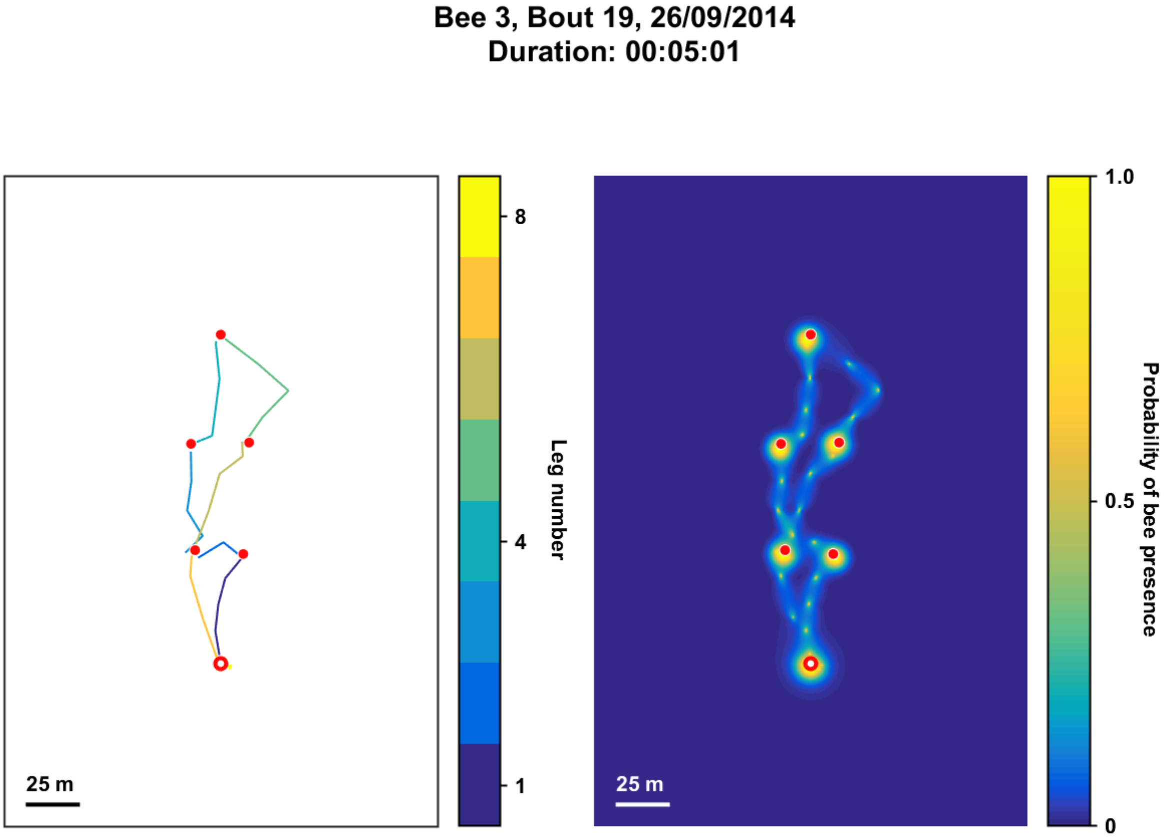

Bee 3, Bout 19, 26/09/2014
Duration: 00:05:01
25 m
8
4
1
Leg number
25 m
1.0
0.5
0
Probability of bee presence

Fig S118

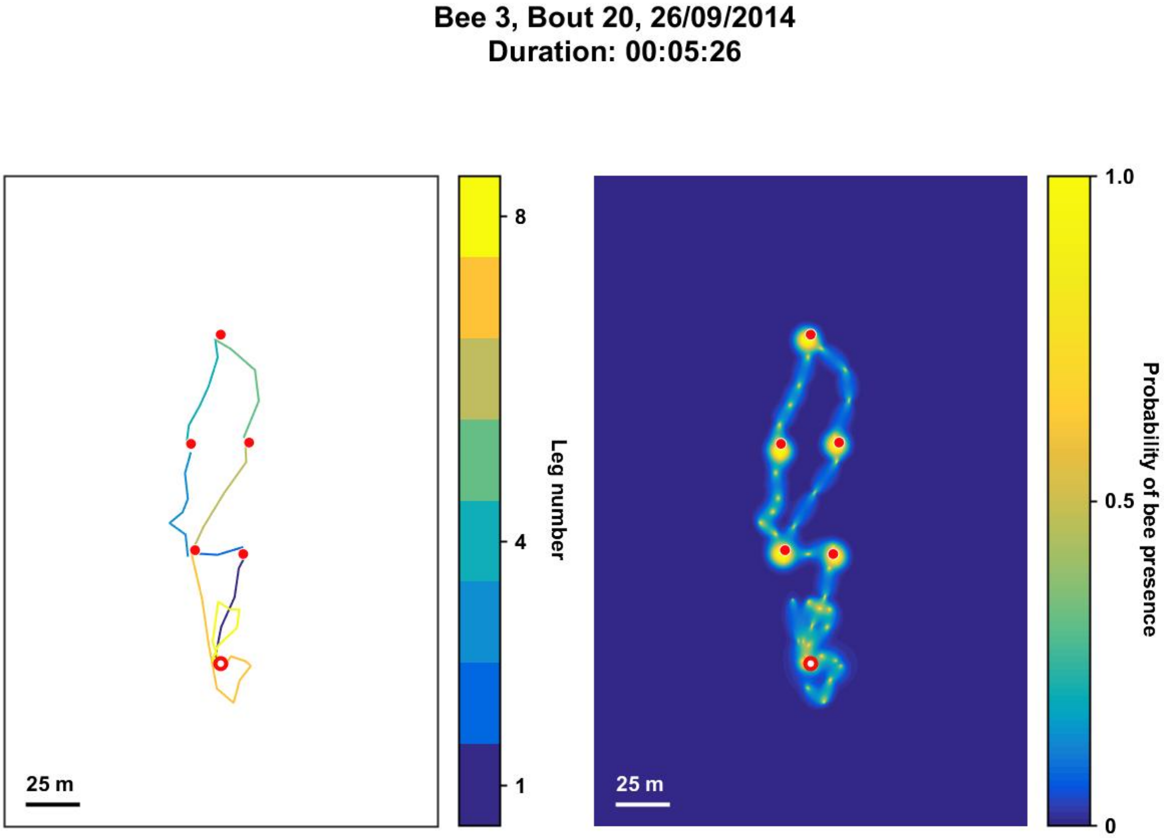

Bee 3, Bout 20, 26/09/2014
Duration: 00:05:26
25 m
8
4
1
Leg number
25 m
1.0
0.5
0
Probability of bee presence

Fig S119

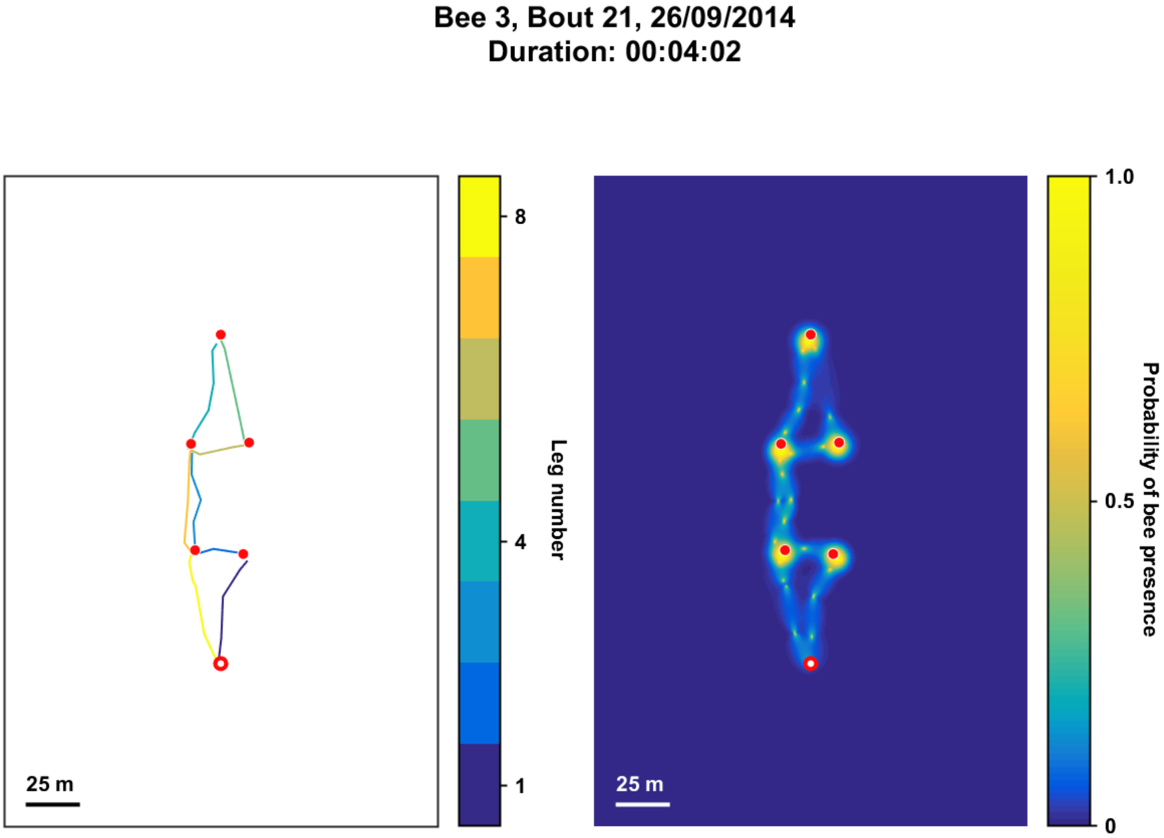
Bee 3, Bout 21, 26/09/2014
Duration: 00:04:02
25 m
8
4
1
Leg number
25 m
1.0
0.5
0
Probability of bee presence

Fig S120

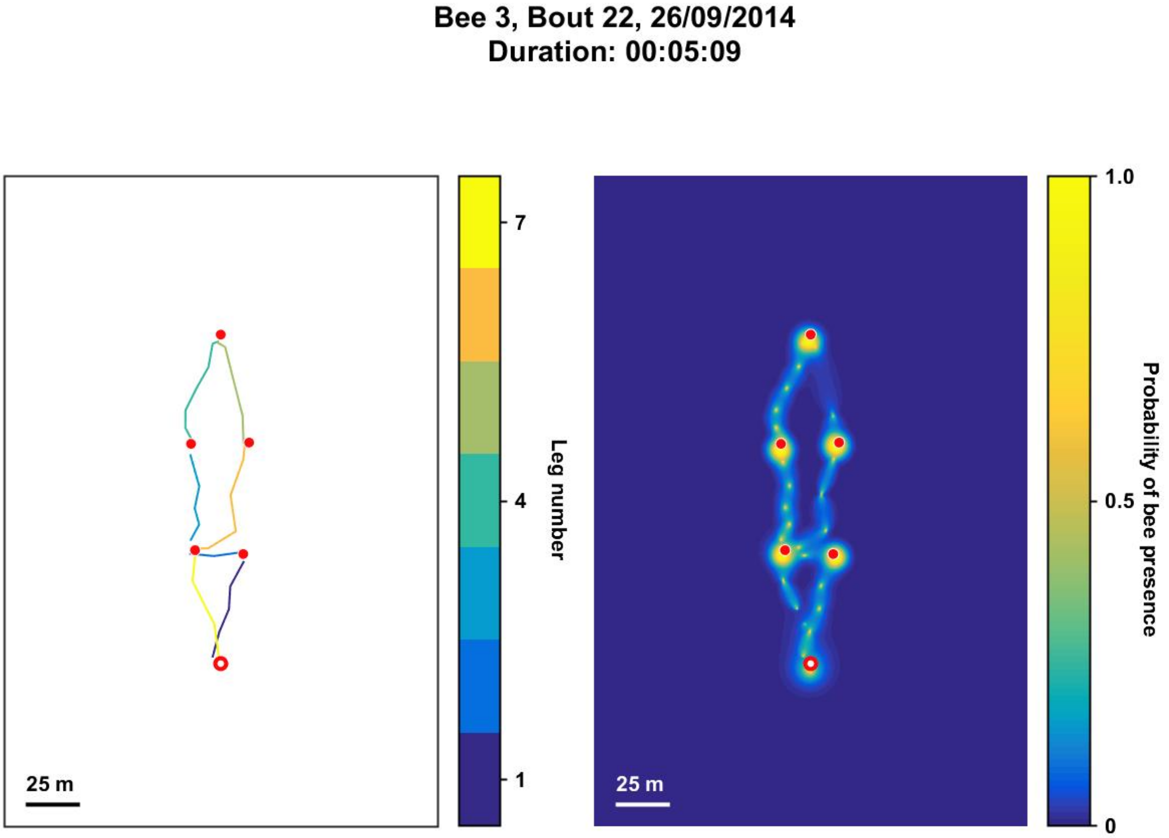

Bee 3, Bout 22, 26/09/2014
Duration: 00:05:09
25 m
Leg number
7
4
1
25 m
Probability of bee presence
1.0
0.5
0

Fig S121

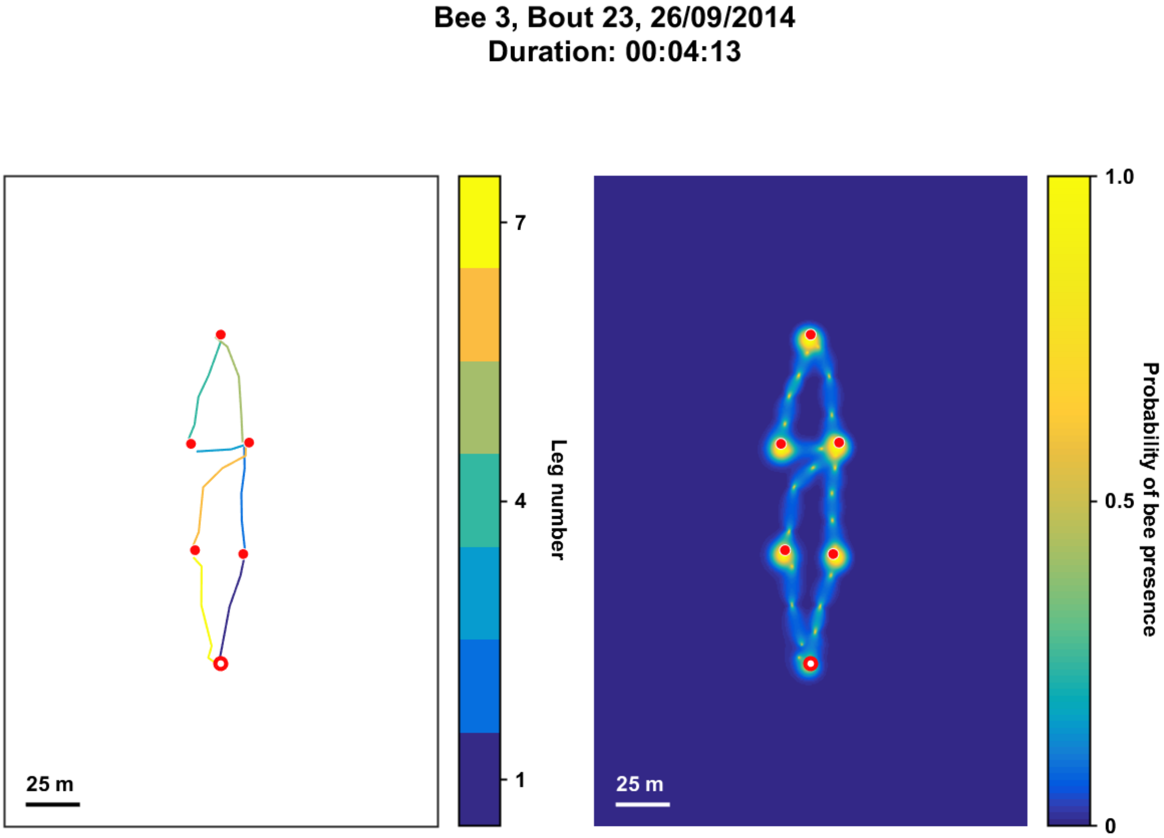
Bee 3, Bout 23, 26/09/2014
Duration: 00:04:13
25 m
7
4
1
Leg number
25 m
1.0
0.5
0
Probability of bee presence

Fig S122

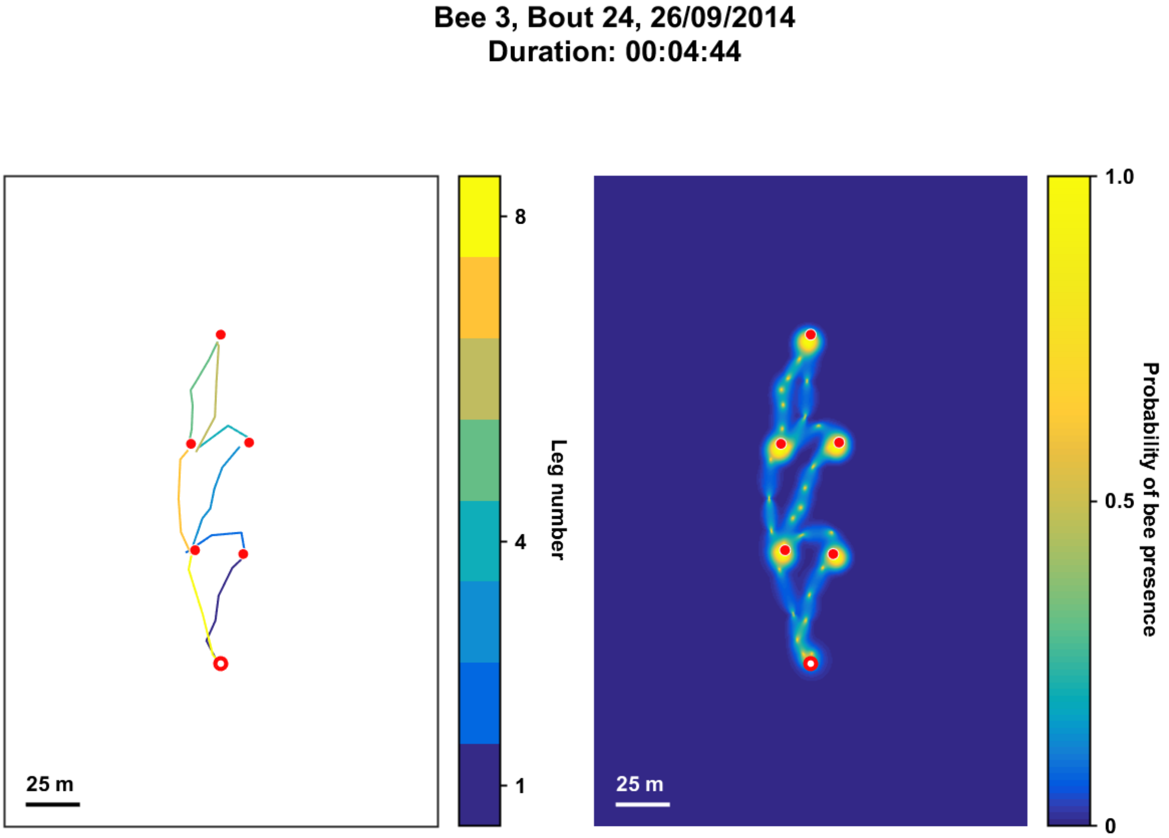
Bee 3, Bout 24, 26/09/2014
Duration: 00:04:44
25 m
8
4
1
Leg number
25 m
1.0
0.5
0
Probability of bee presence

Fig S123

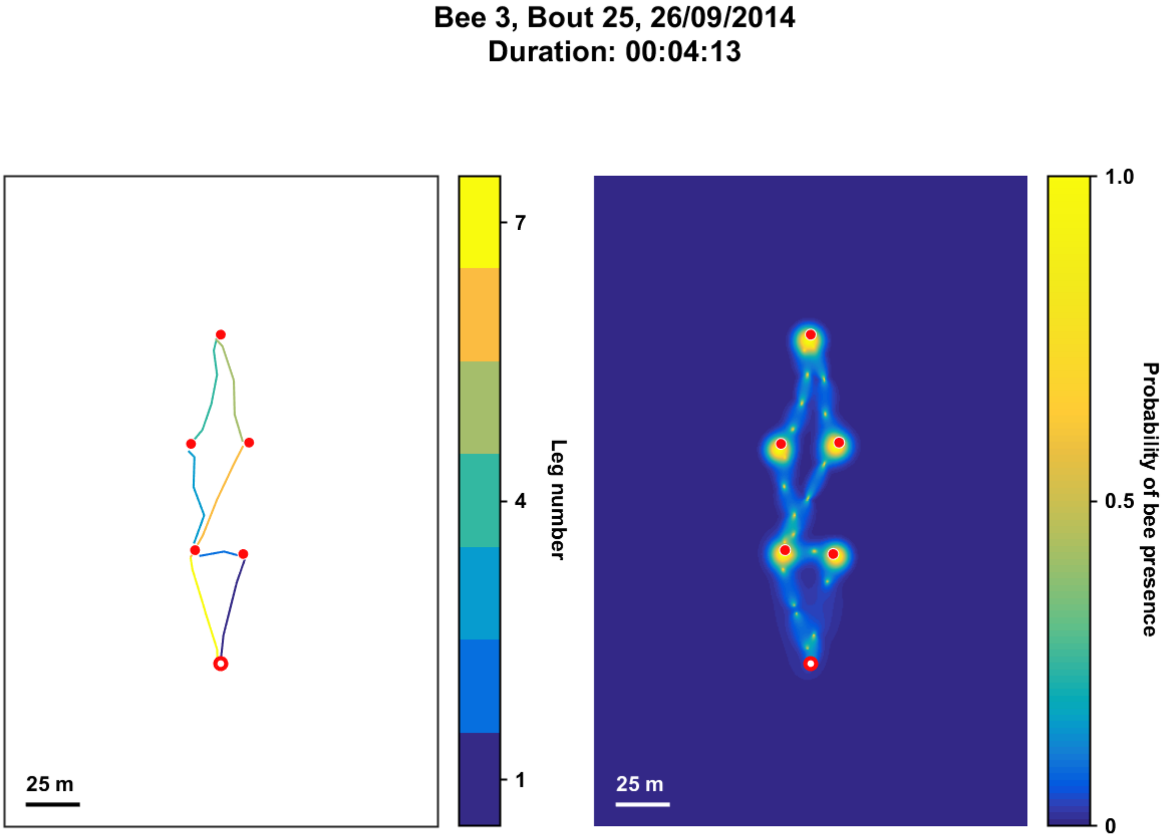

Bee 3, Bout 25, 26/09/2014
Duration: 00:04:13
25 m
7
4
1
Leg number
25 m
1.0
0.5
0
Probability of bee presence

Fig S124

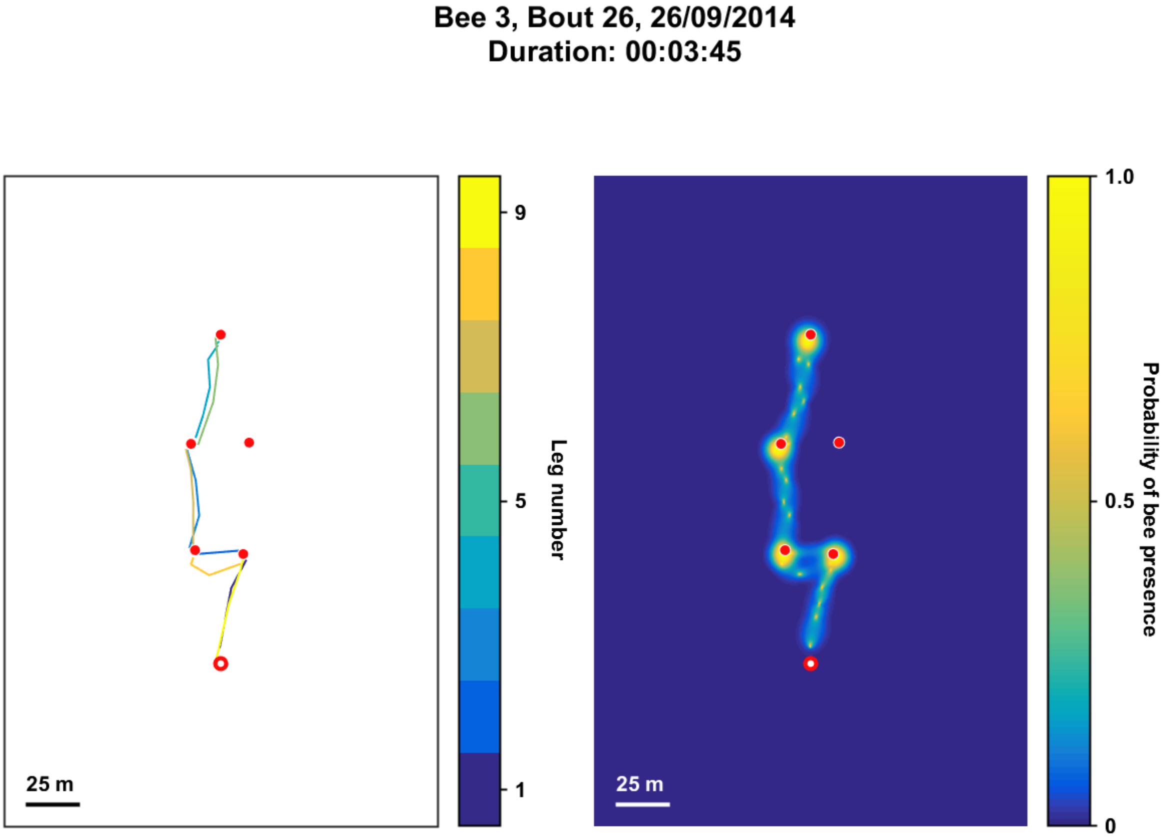

Bee 3, Bout 26, 26/09/2014
Duration: 00:03:45
25 m
9
5
1
Leg number
25 m
1.0
0.5
0
Probability of bee presence

Fig S125

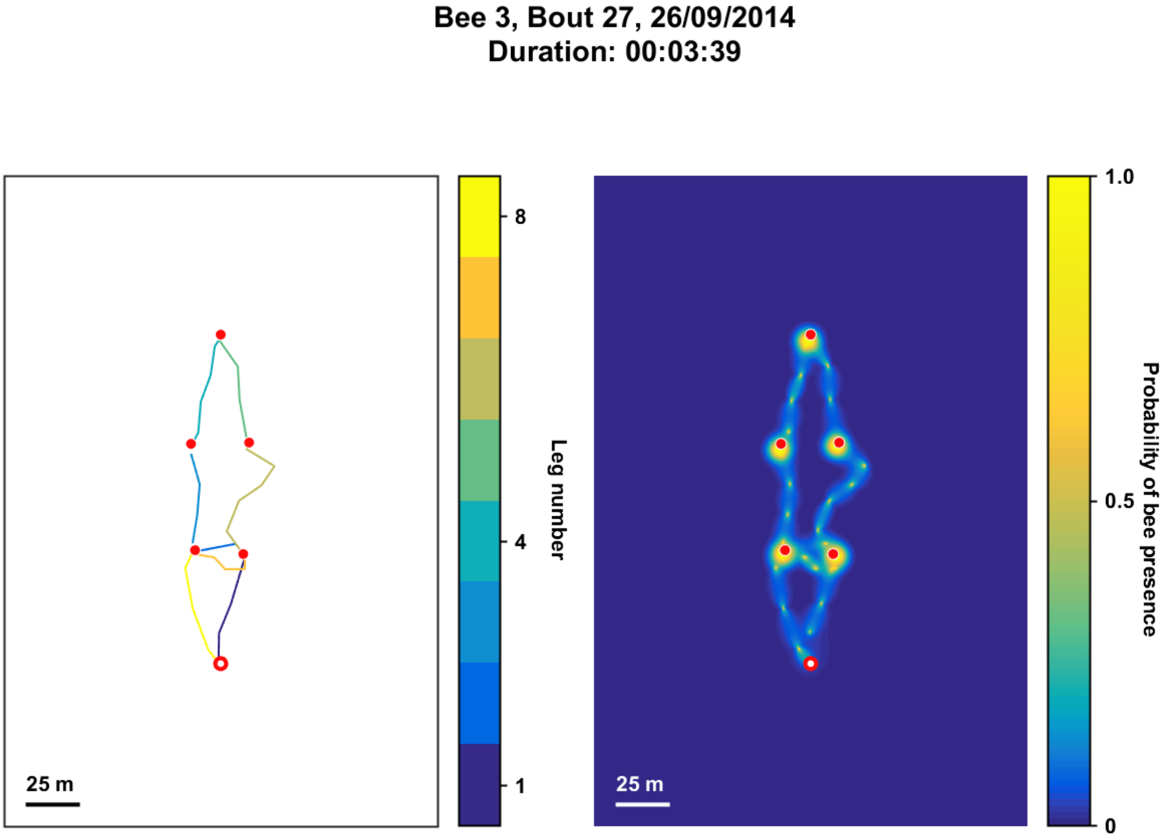

Bee 3, Bout 27, 26/09/2014
Duration: 00:03:39
8
4
1
Leg number
25 m
25 m
1.0
0.5
0
Probability of bee presence

Fig S126

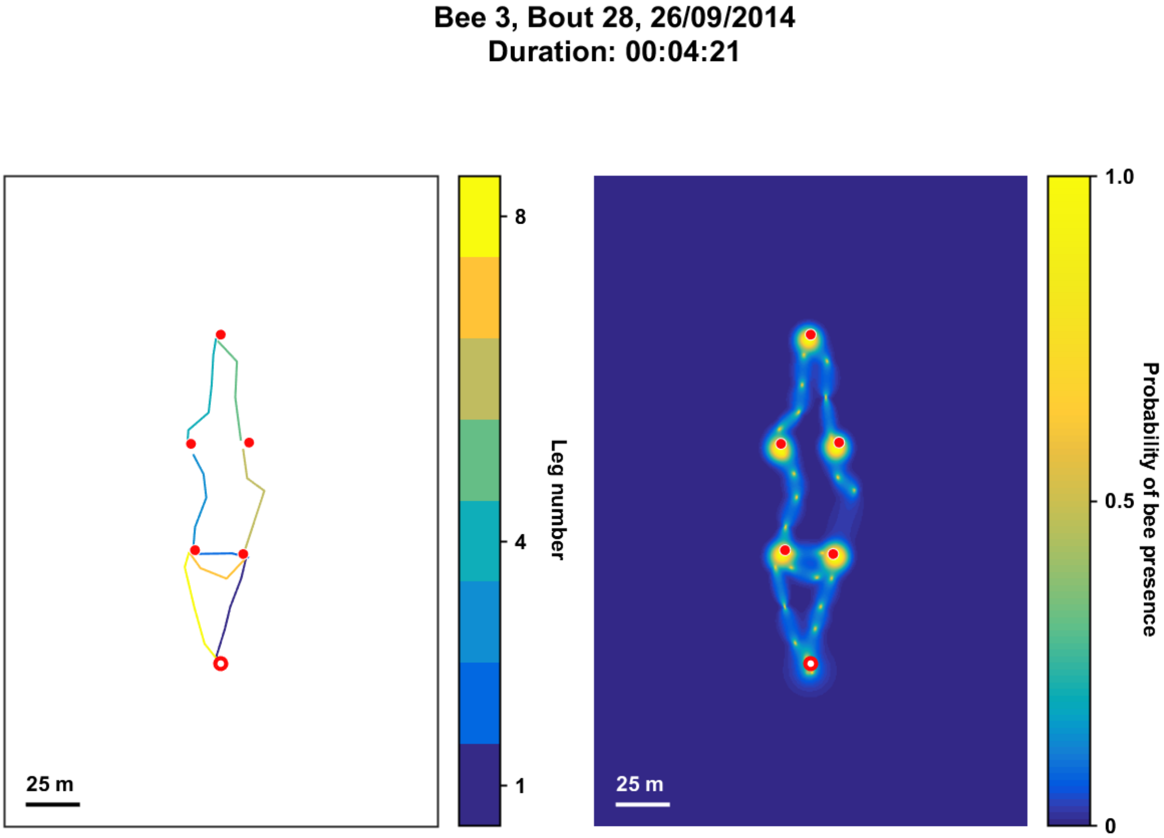

Bee 3, Bout 28, 26/09/2014
Duration: 00:04:21
25 m
8
4
1
Leg number
25 m
1.0
0.5
0
Probability of bee presence

Fig S127

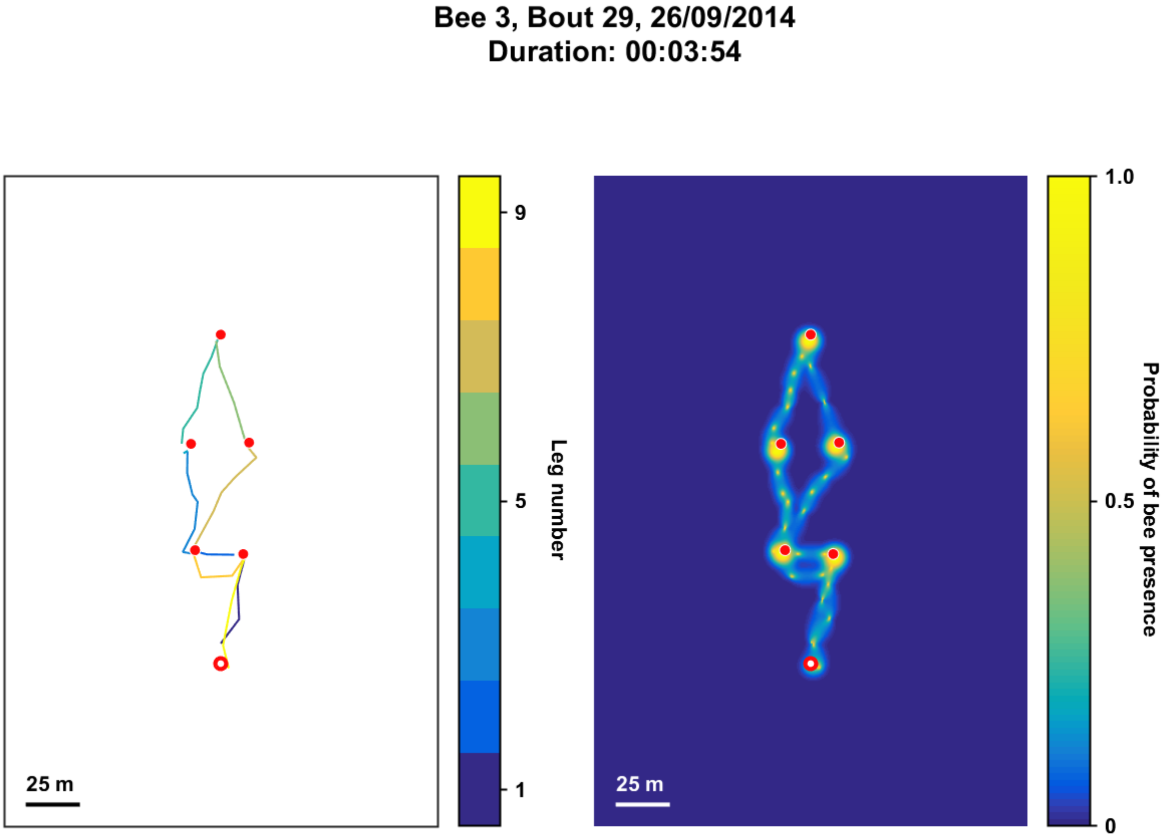

Bee 3, Bout 29, 26/09/2014
Duration: 00:03:54
25 m
9
5
1
Leg number
25 m
1.0
0.5
0
Probability of bee presence

Fig S128

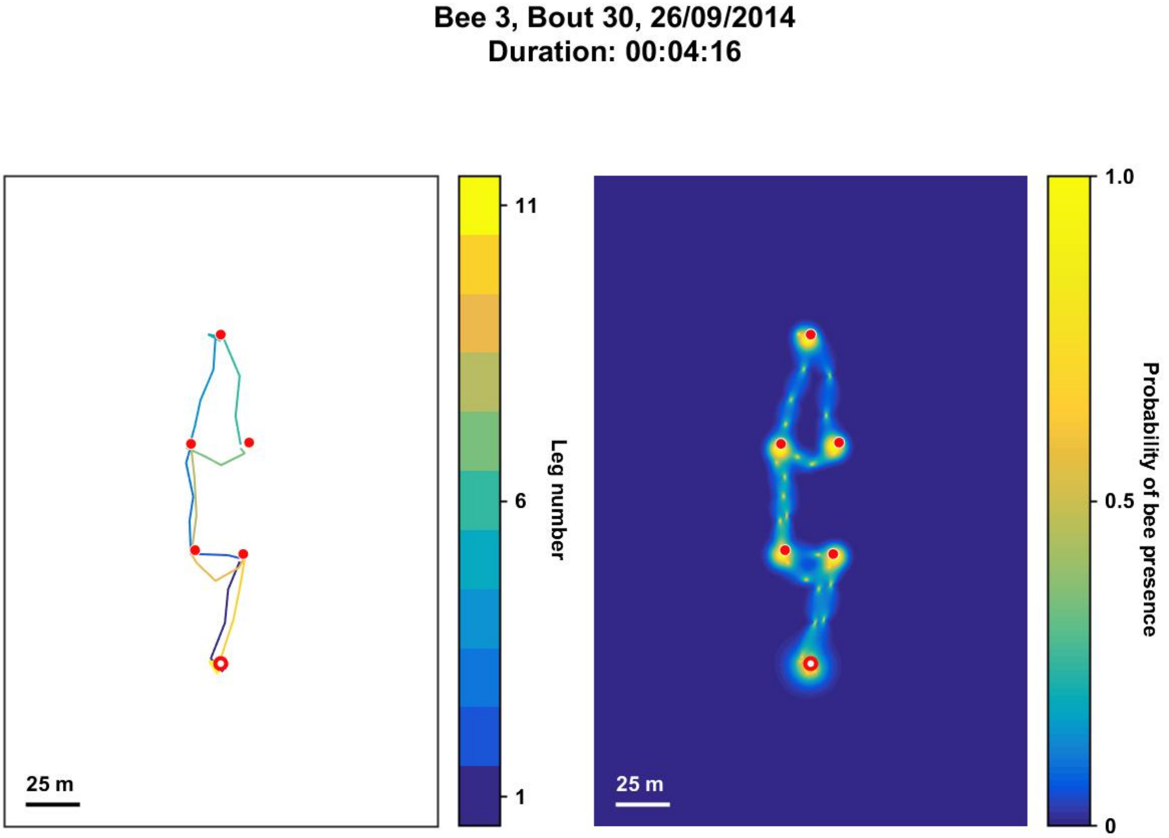
Bee 3, Bout 30, 26/09/2014
Duration: 00:04:16
25 m
Leg number
1
6
11
25 m
Probability of bee presence
0
0.5
1.0

Fig S129

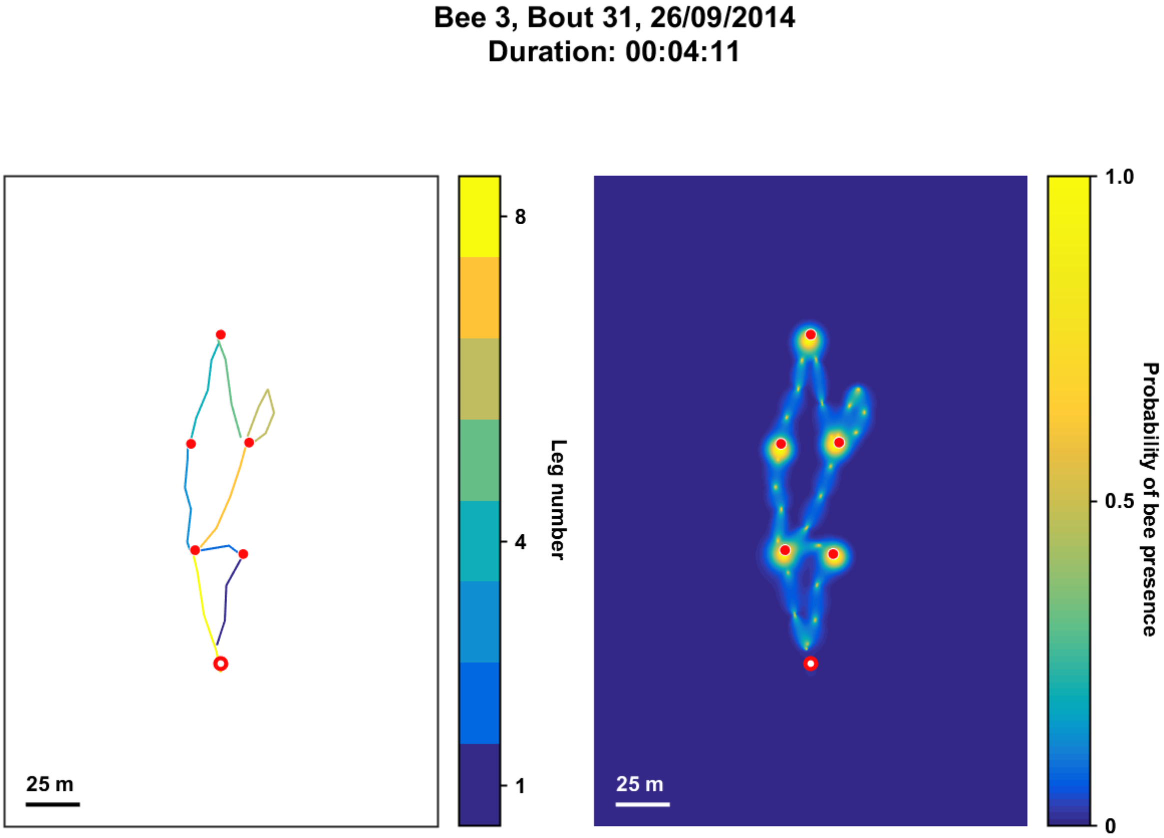

Bee 3, Bout 31, 26/09/2014
Duration: 00:04:11
25 m
8
4
1
Leg number
25 m
1.0
0.5
0
Probability of bee presence

Fig S130

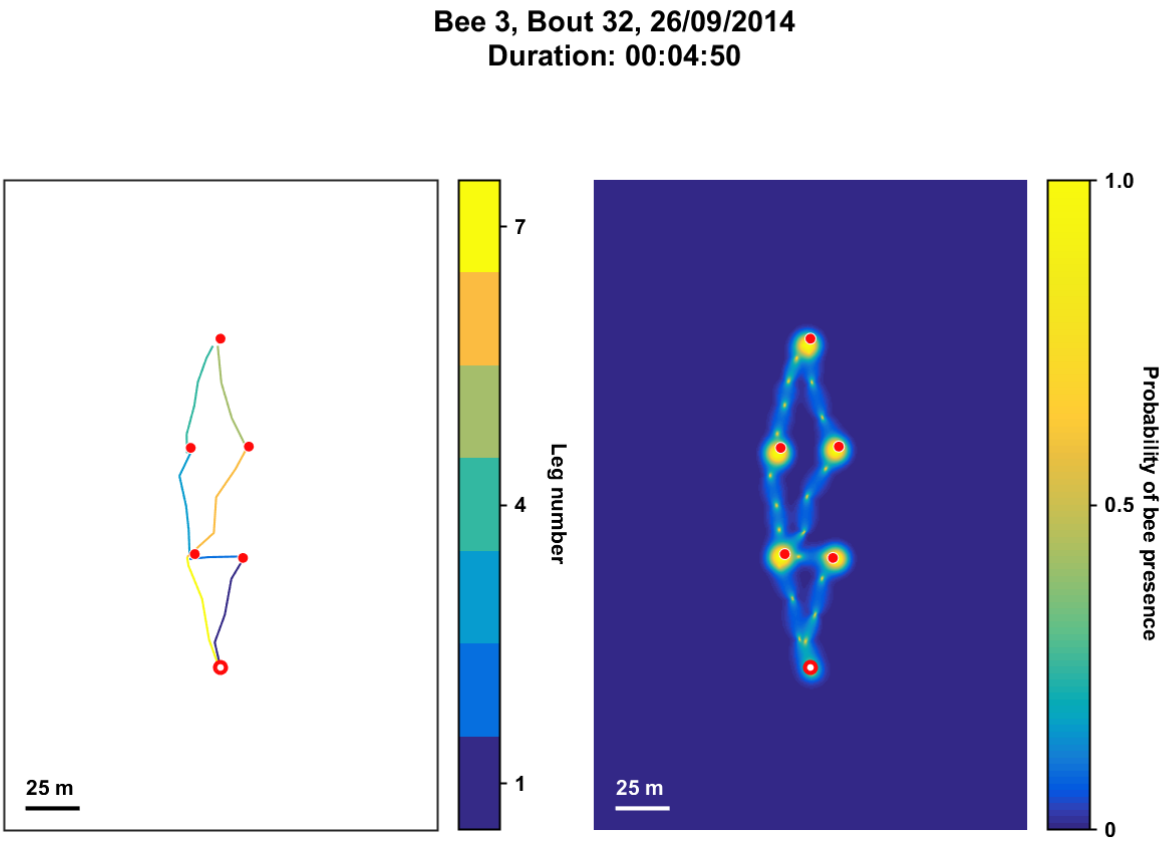
Bee 3, Bout 32, 26/09/2014
Duration: 00:04:50
25 m
7
4
1
Leg number
25 m
1.0
0.5
0
Probability of bee presence

Fig S131

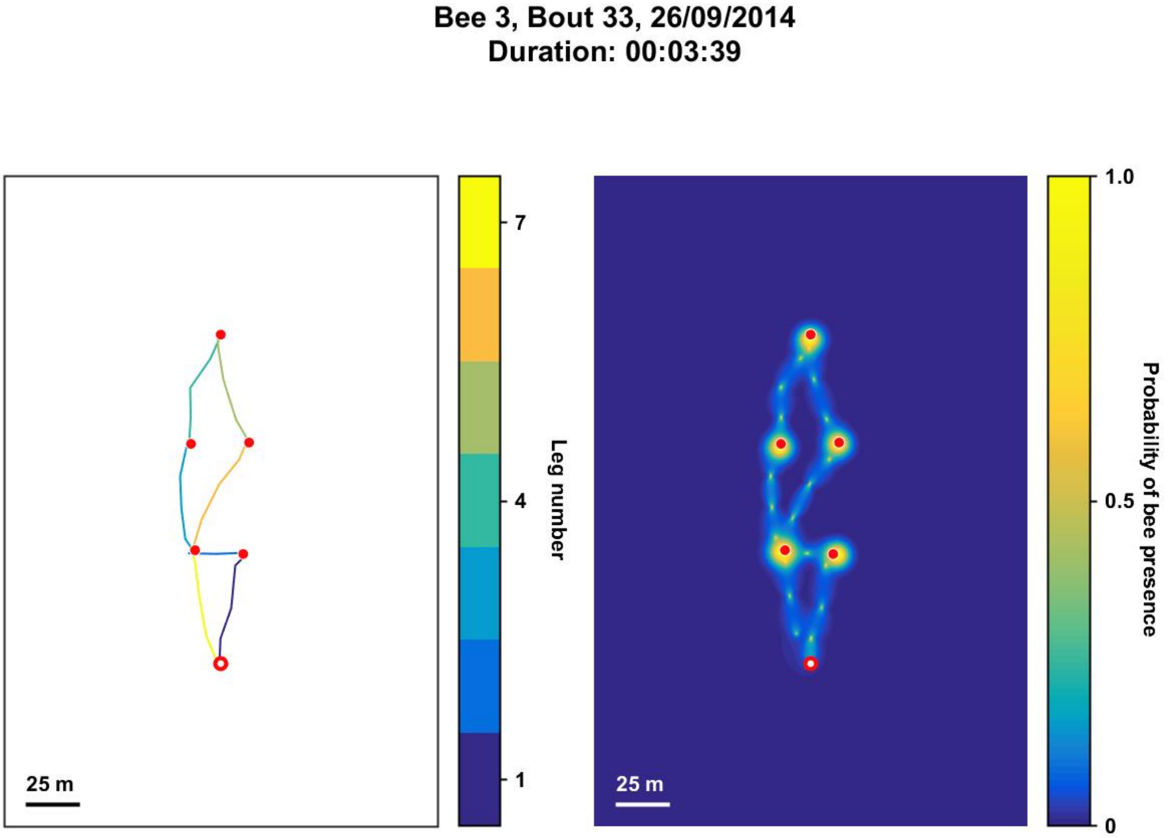
Bee 3, Bout 33, 26/09/2014
Duration: 00:03:39
25 m
7
4
1
Leg number
25 m
1.0
0.5
0
Probability of bee presence

Fig S132

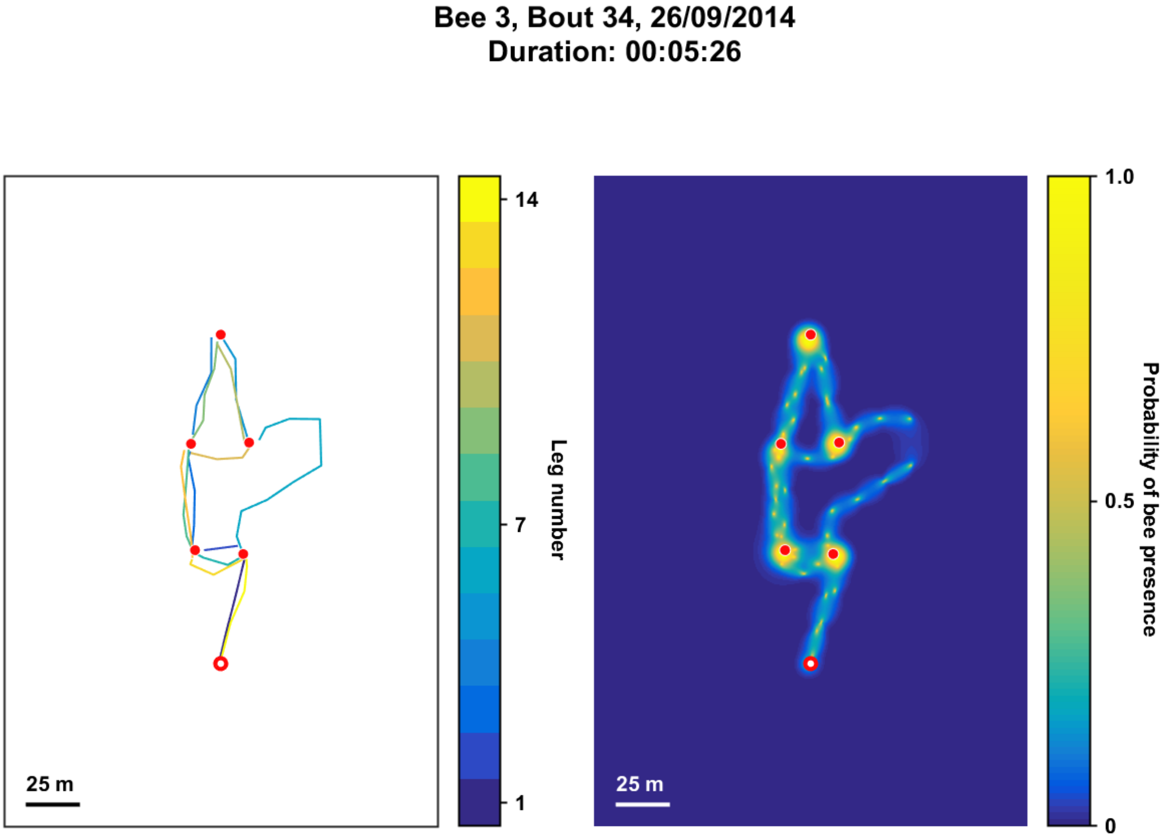
Bee 3, Bout 34, 26/09/2014
Duration: 00:05:26
25 m
14
7
1
Leg number
25 m
1.0
0.5
0
Probability of bee presence

Fig S133

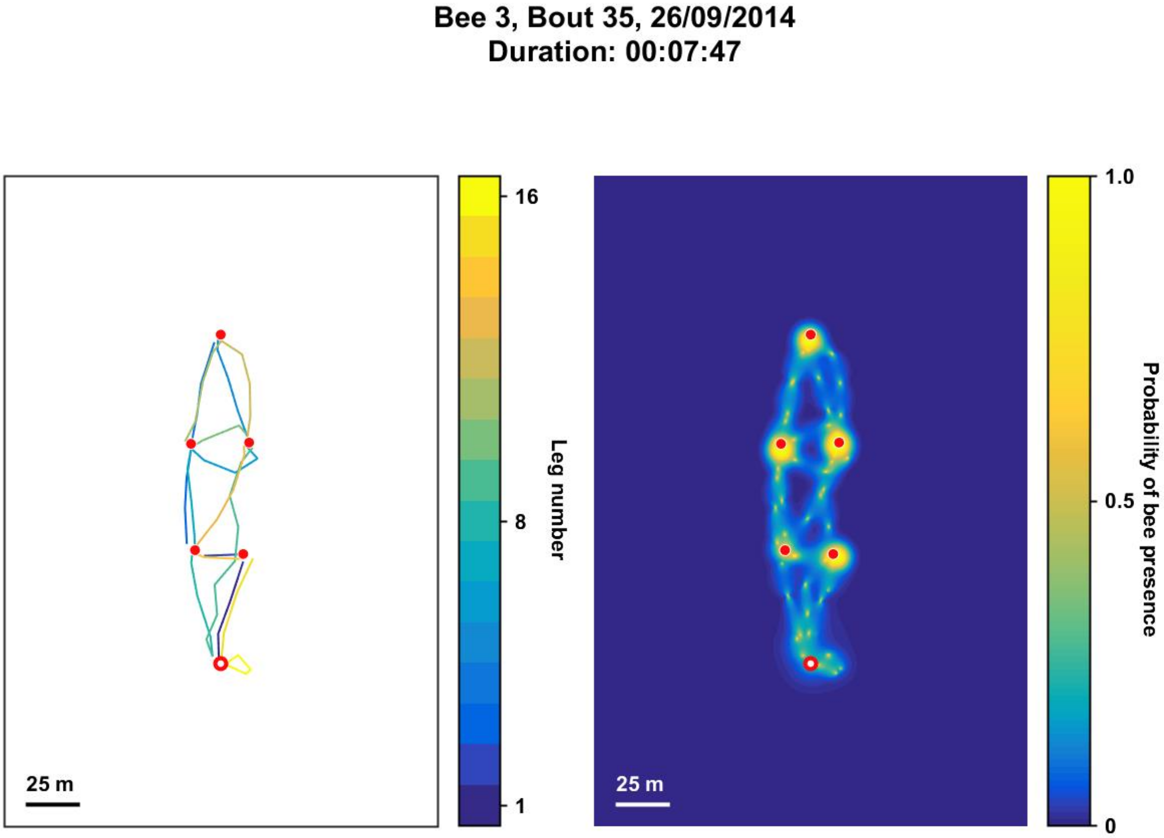
Bee 3, Bout 35, 26/09/2014
Duration: 00:07:47
16
8
1
Leg number
25 m
1.0
0.5
0
Probability of bee presence
25 m

Fig S134

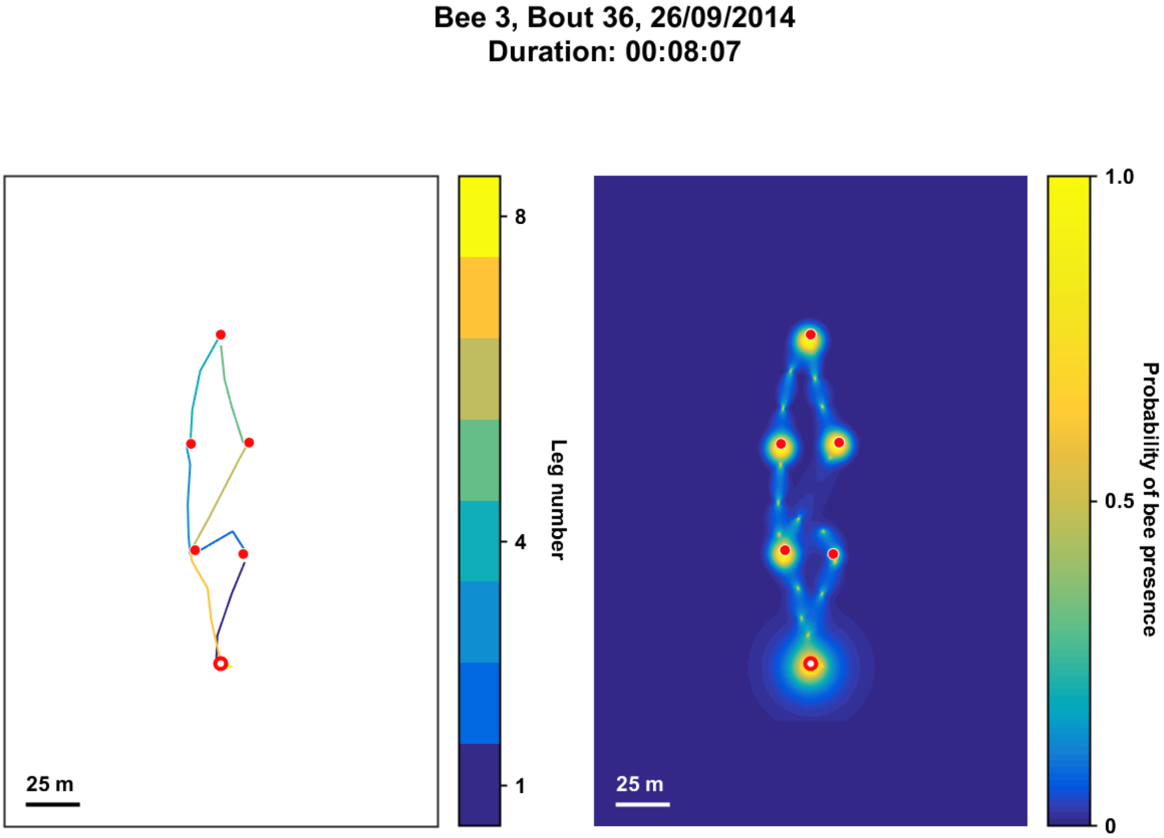

Bee 3, Bout 36, 26/09/2014
Duration: 00:08:07
25 m
8
4
1
Leg number
25 m
1.0
0.5
0
Probability of bee presence

Fig S135

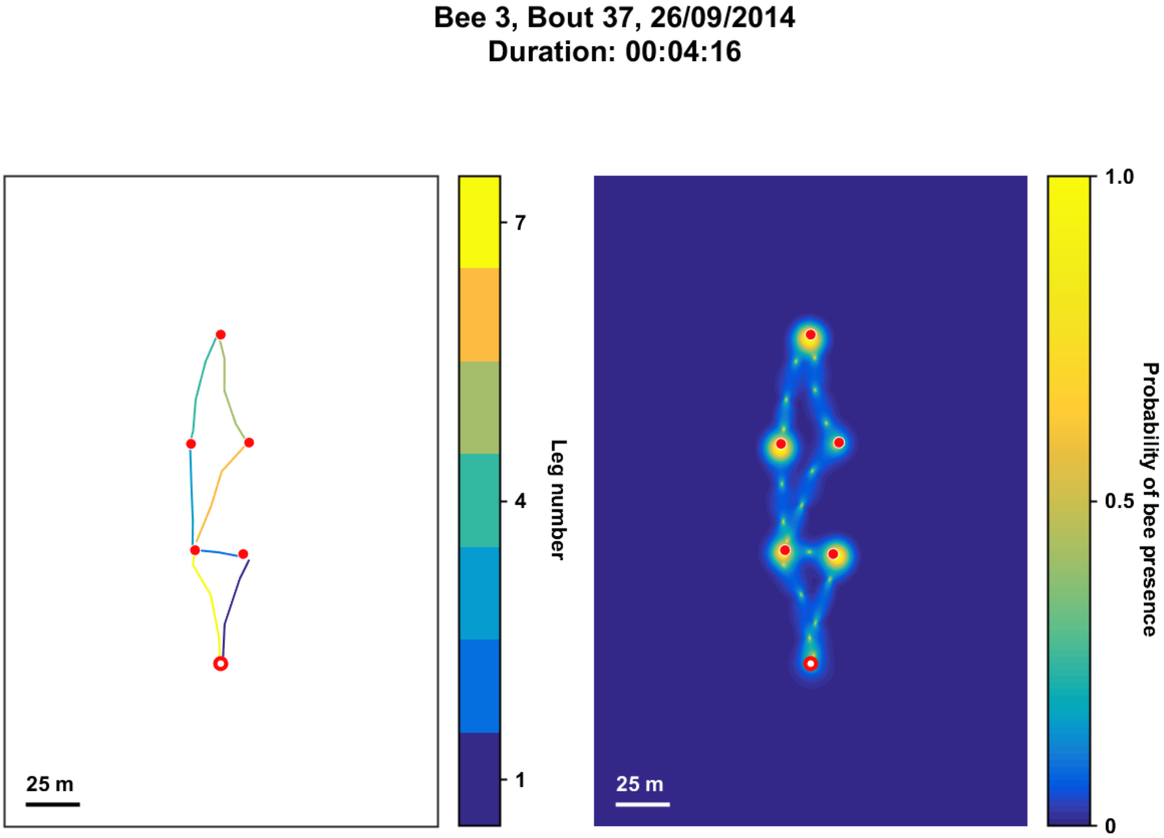

Bee 3, Bout 37, 26/09/2014
Duration: 00:04:16
25 m
Leg number
7
4
1
25 m
Probability of bee presence
1.0
0.5
0

Fig S136

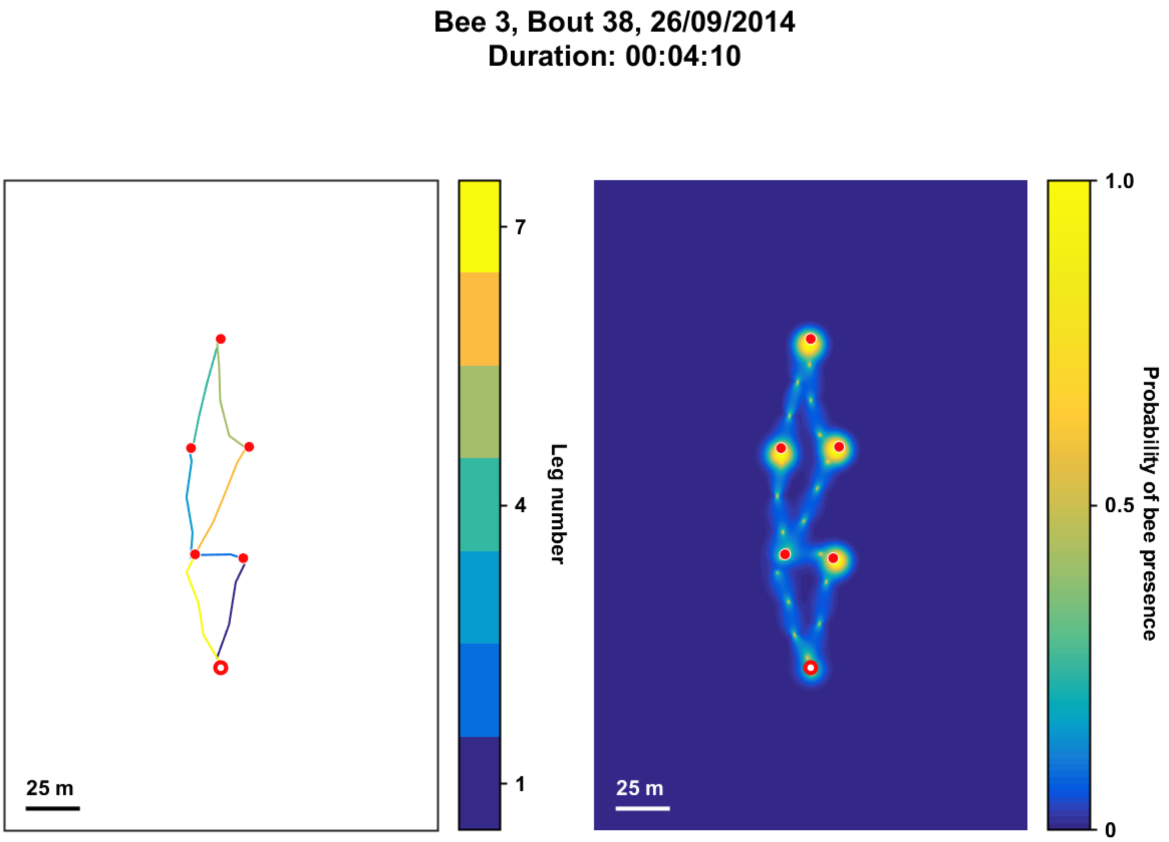

Bee 3, Bout 38, 26/09/2014
Duration: 00:04:10
25 m
Leg number
7
4
1
25 m
1.0
0.5
0
Probability of bee presence

Fig S137

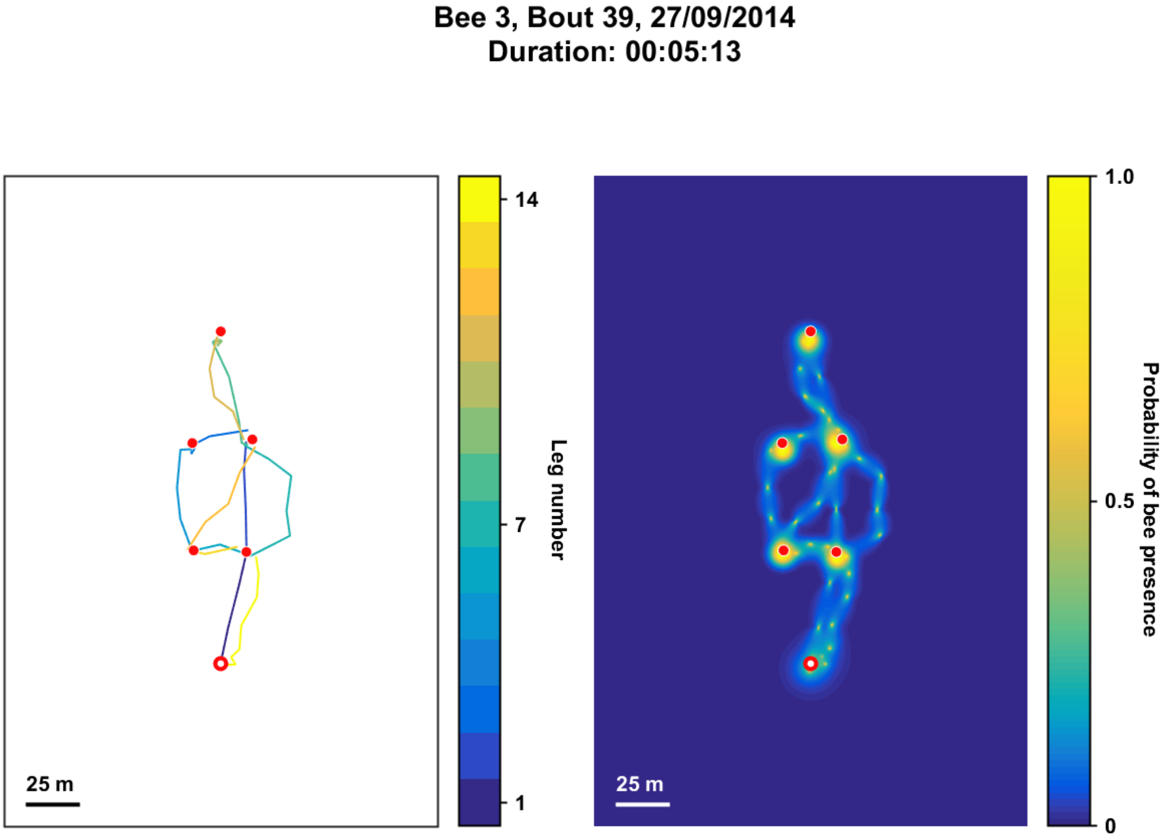

Bee 3, Bout 39, 27/09/2014
Duration: 00:05:13
25 m
14
7
1
Leg number
25 m
1.0
0.5
0
Probability of bee presence

Fig S138

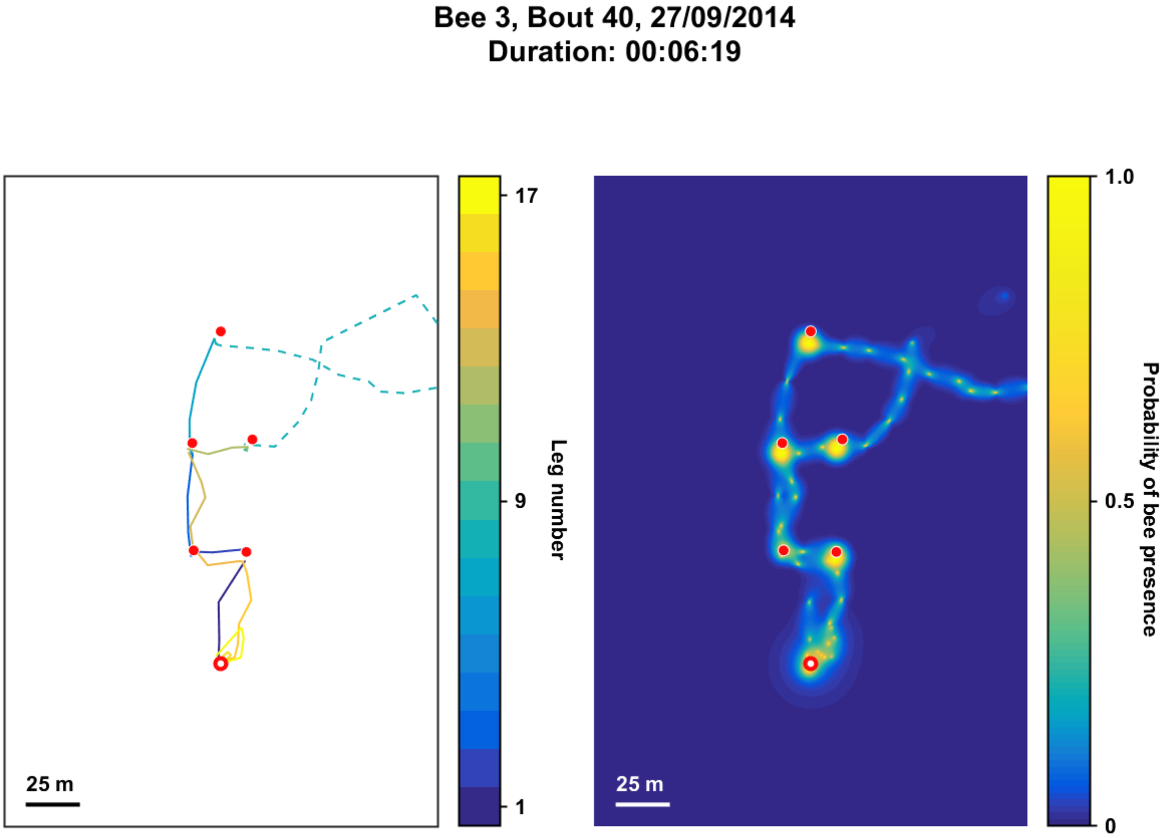
Bee 3, Bout 40, 27/09/2014
Duration: 00:06:19
25 m
17
9
1
Leg number
25 m
1.0
0.5
0
Probability of bee presence

Fig S139

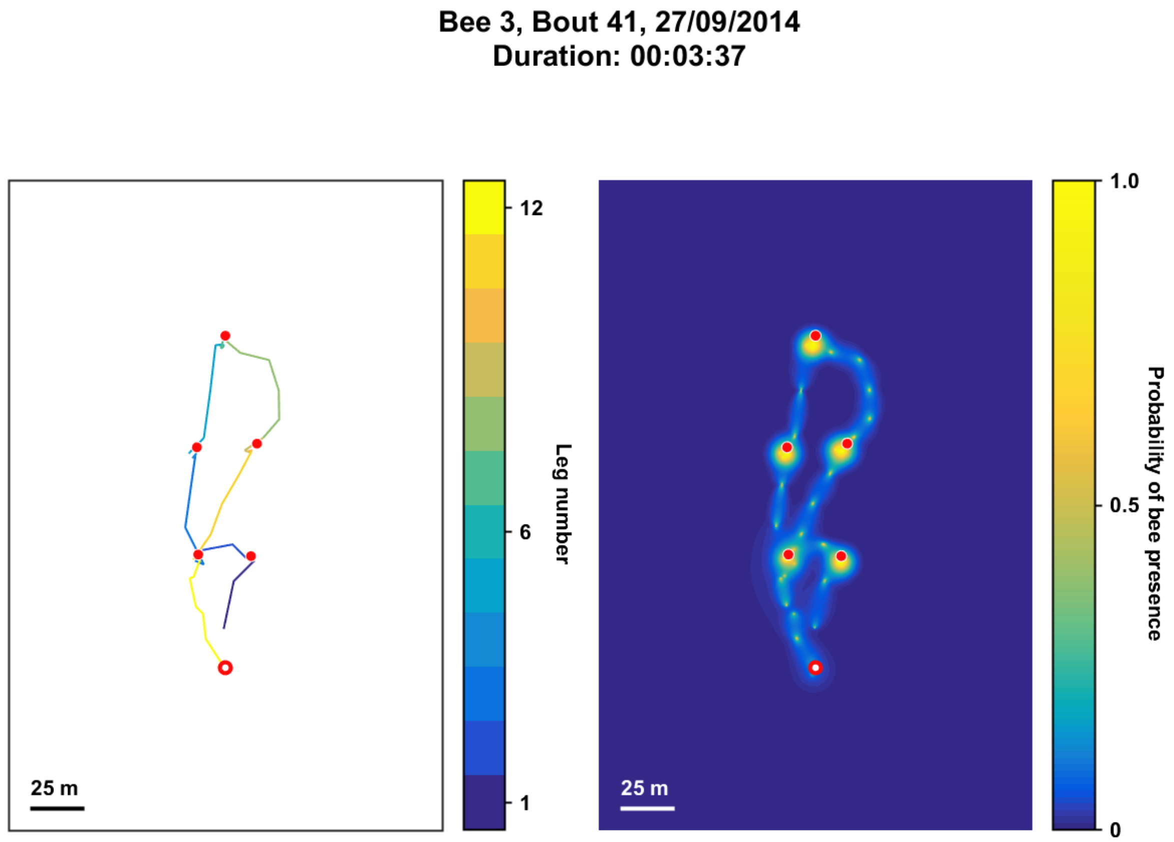

Bee 3, Bout 41, 27/09/2014
Duration: 00:03:37
25 m
12
6
1
Leg number
25 m
1.0
0.5
0
Probability of bee presence

Fig S140

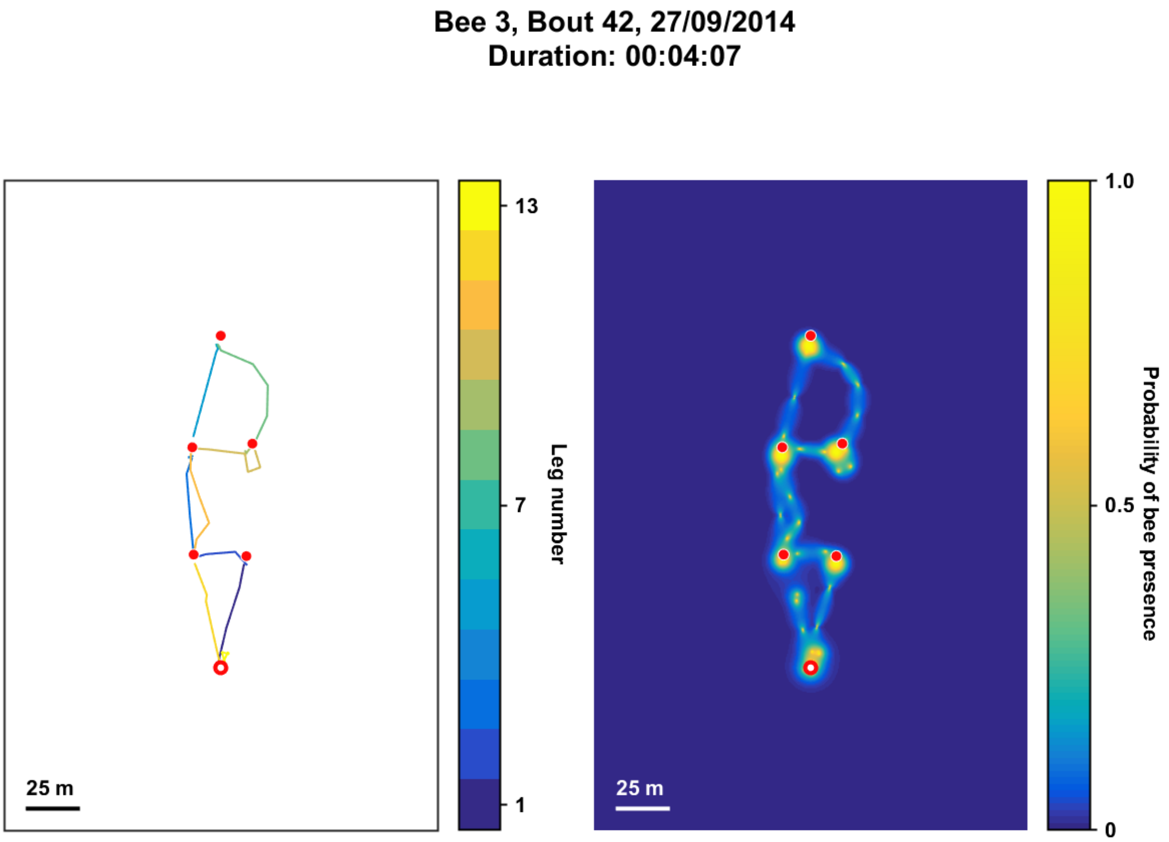
Bee 3, Bout 42, 27/09/2014
Duration: 00:04:07
25 m
13
7
1
Leg number
25 m
1.0
0.5
0
Probability of bee presence

Fig S141

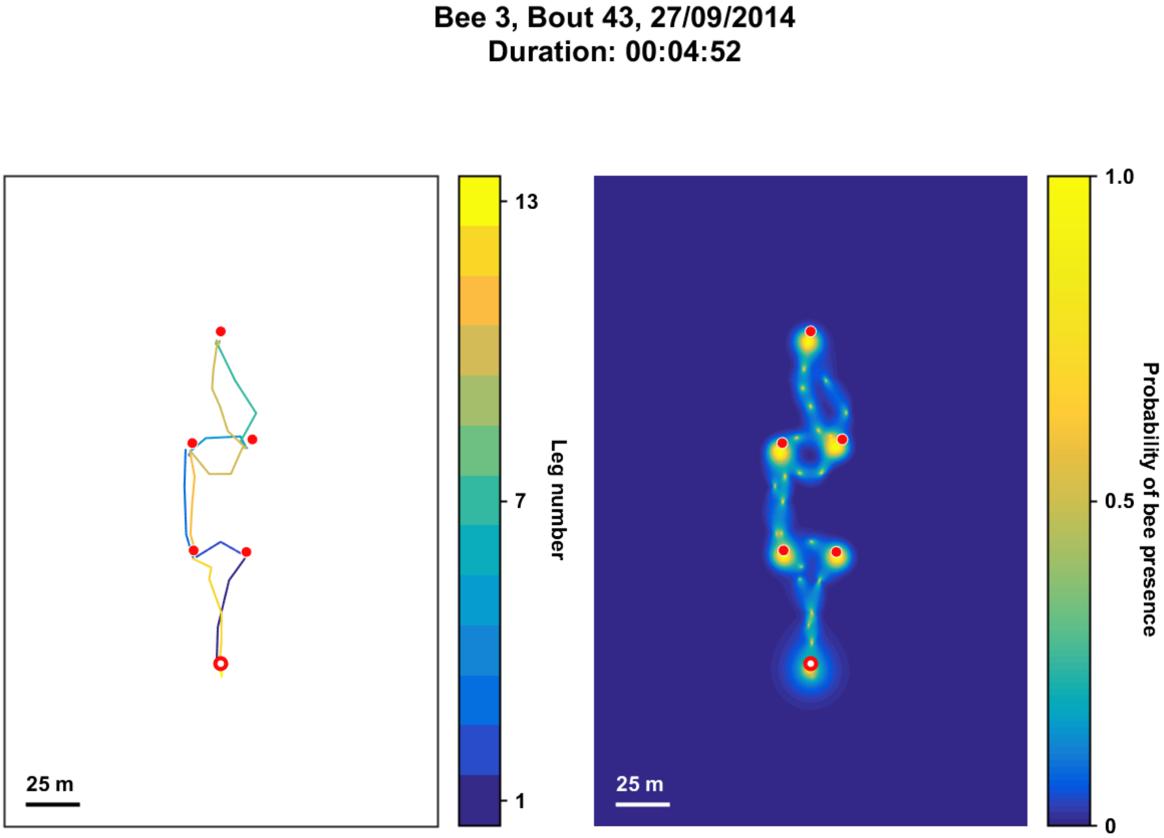
Bee 3, Bout 43, 27/09/2014
Duration: 00:04:52
25 m
13
7
1
Leg number
25 m
1.0
0.5
0
Probability of bee presence

Fig S142

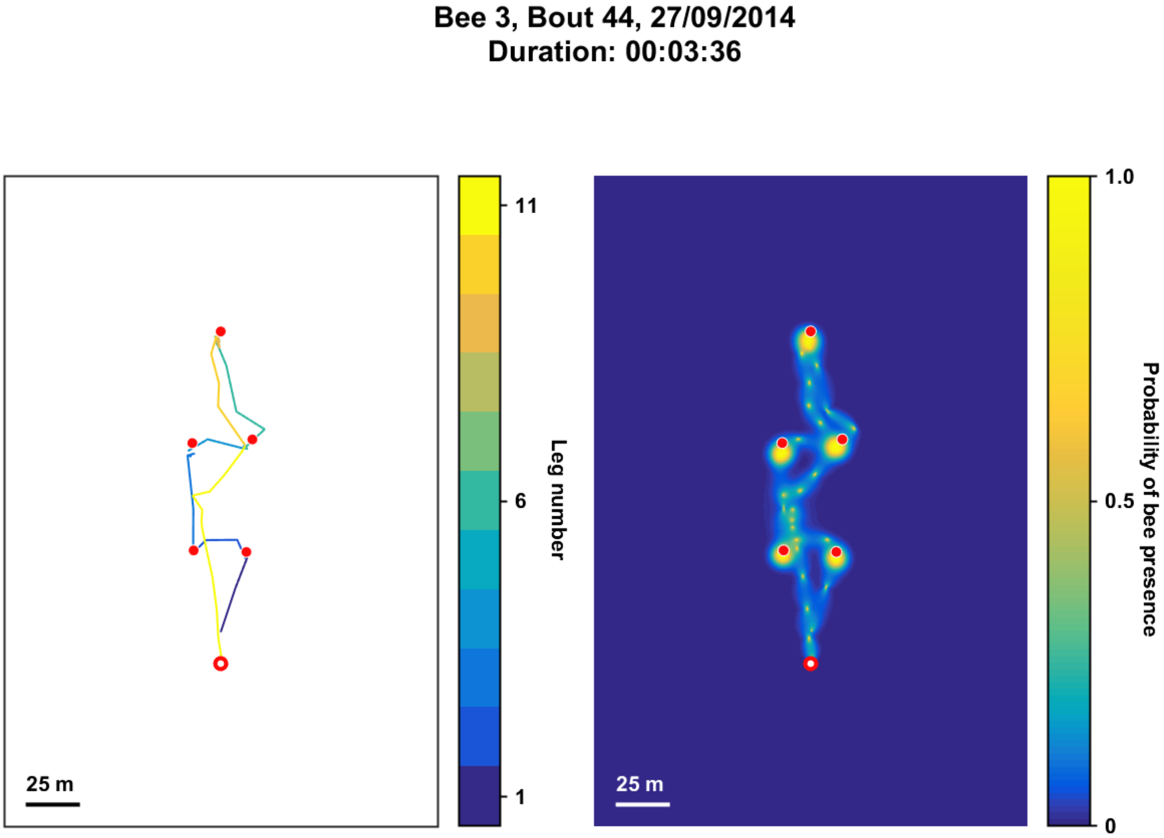
Bee 3, Bout 44, 27/09/2014
Duration: 00:03:36
25 m
11
6
1
Leg number
25 m
1.0
0.5
0
Probability of bee presence

Fig S143

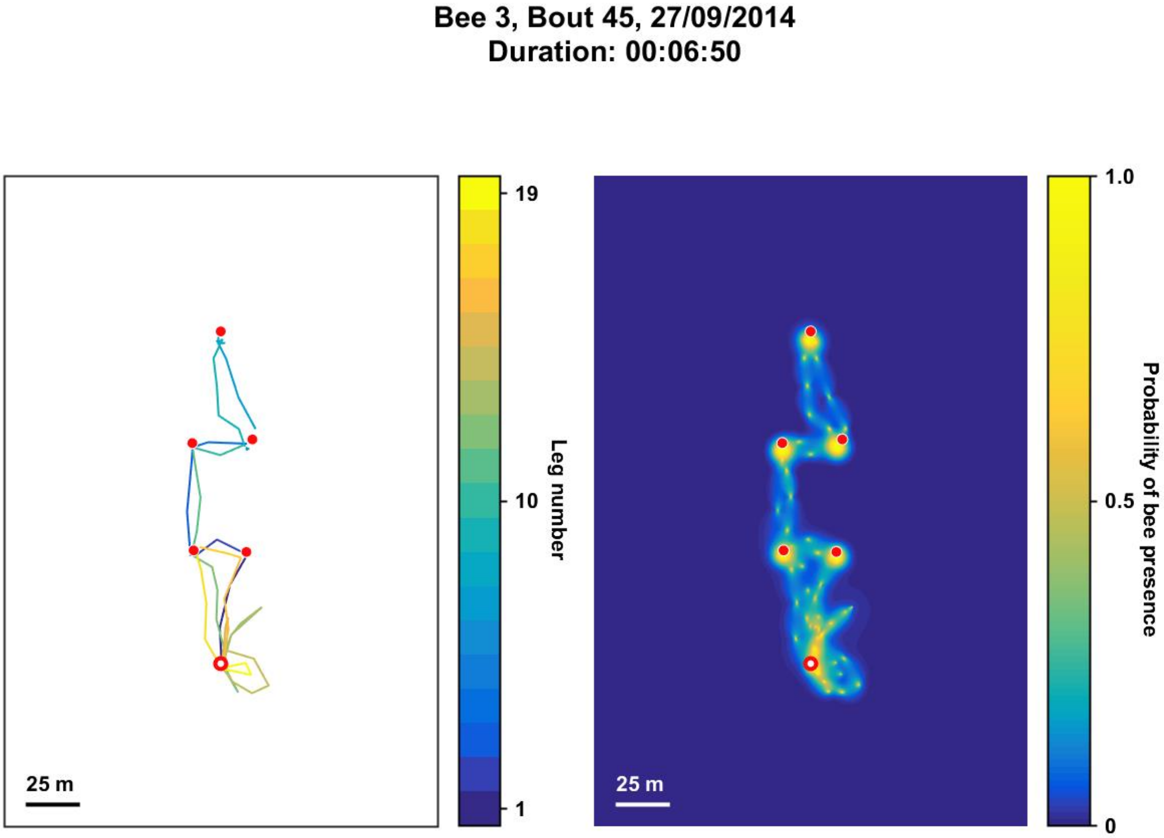

Bee 3, Bout 45, 27/09/2014
Duration: 00:06:50
25 m
19
10
1
Leg number
25 m
1.0
0.5
0
Probability of bee presence

Fig S144

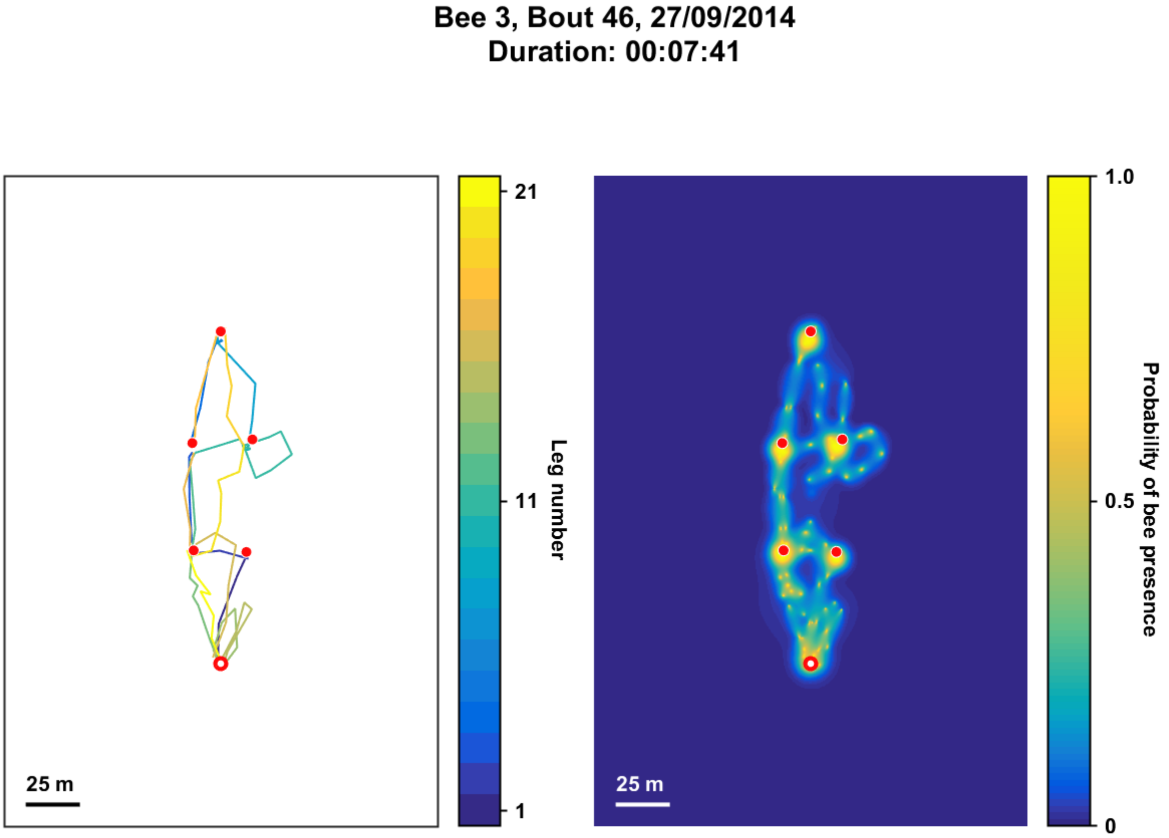

Bee 3, Bout 46, 27/09/2014
Duration: 00:07:41
25 m
21
11
1
Leg number
25 m
1.0
0.5
0
Probability of bee presence

Fig S145

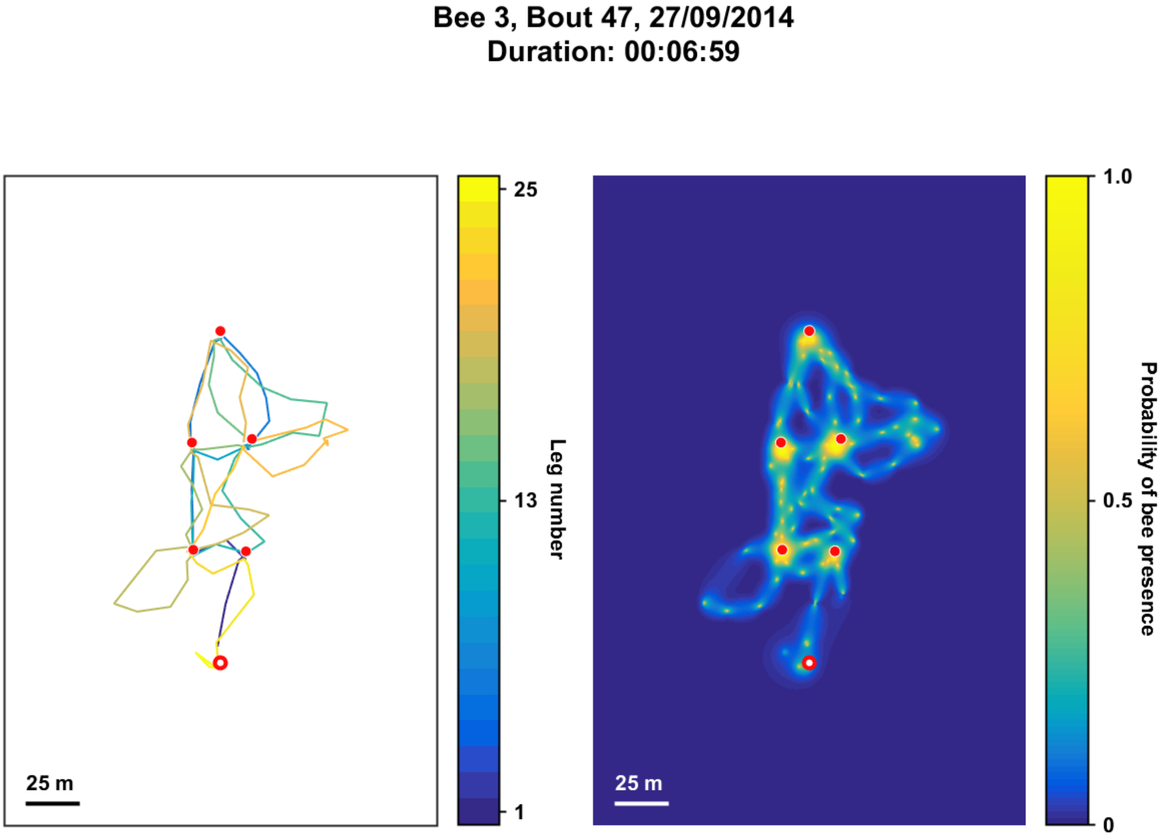

Bee 3, Bout 47, 27/09/2014
Duration: 00:06:59
25 m
25
13
1
Leg number
25 m
1.0
0.5
0
Probability of bee presence

Fig S146

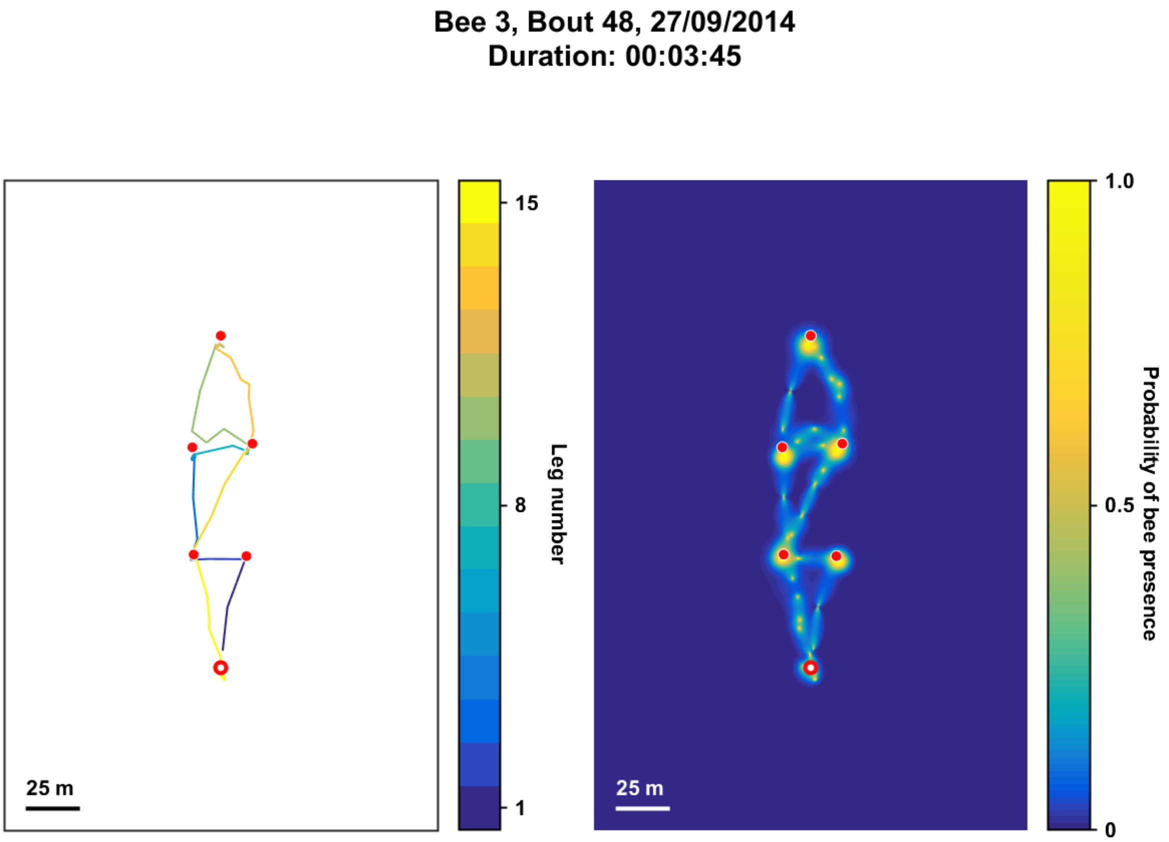

Bee 3, Bout 48, 27/09/2014
Duration: 00:03:45
25 m
15
8
1
Leg number
25 m
1.0
0.5
0
Probability of bee presence

Fig S147

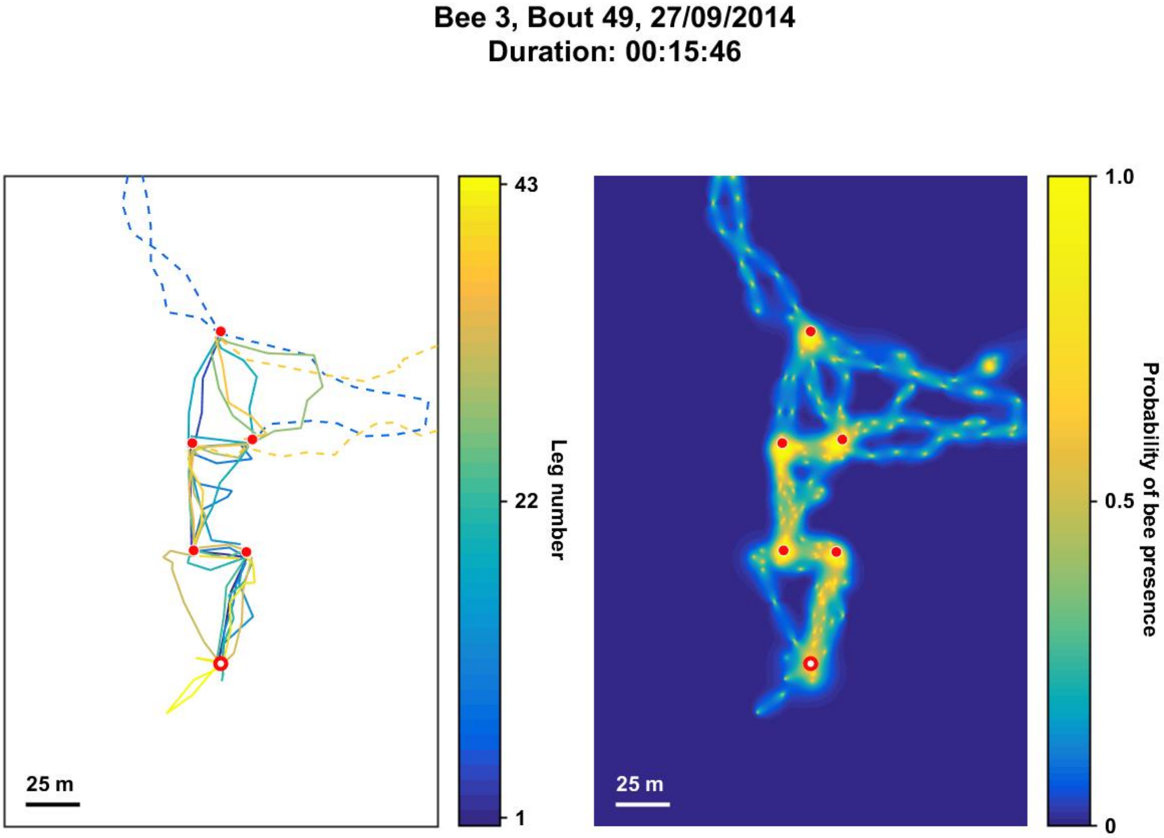

Bee 3, Bout 49, 27/09/2014
Duration: 00:15:46
25 m
43
22
1
Leg number
25 m
1.0
0.5
0
Probability of bee presence

Fig S148

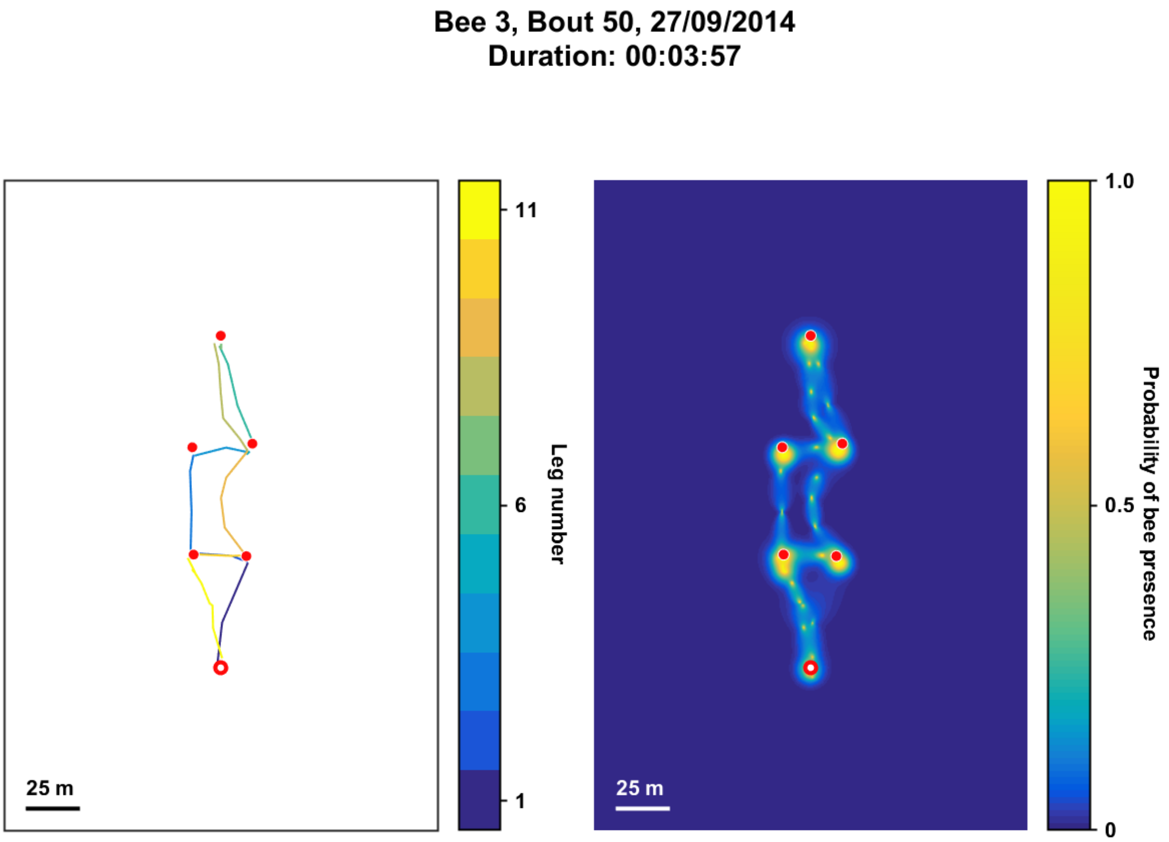
Bee 3, Bout 50, 27/09/2014
Duration: 00:03:57
25 m
11
6
1
Leg number
25 m
1.0
0.5
0
Probability of bee presence

Fig S149

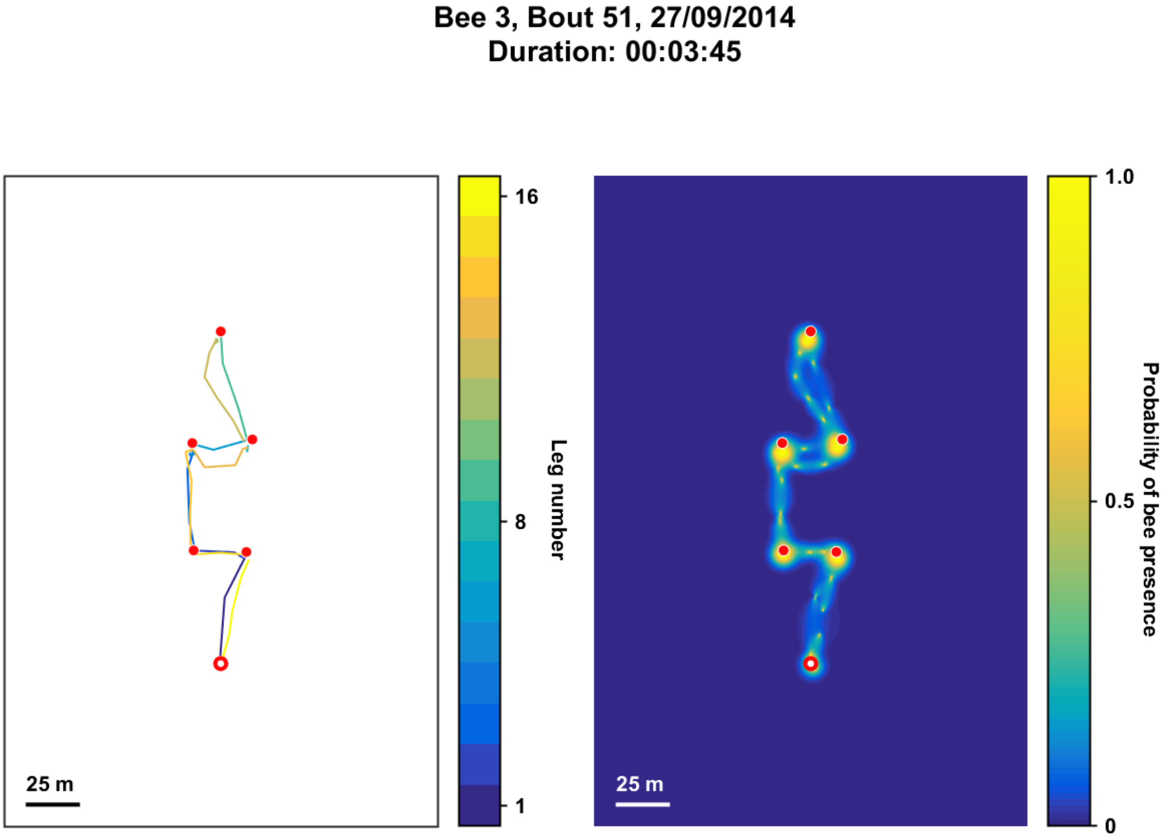

Bee 3, Bout 51, 27/09/2014
Duration: 00:03:45
25 m
16
8
1
Leg number
25 m
1.0
0.5
0
Probability of bee presence

Fig S150

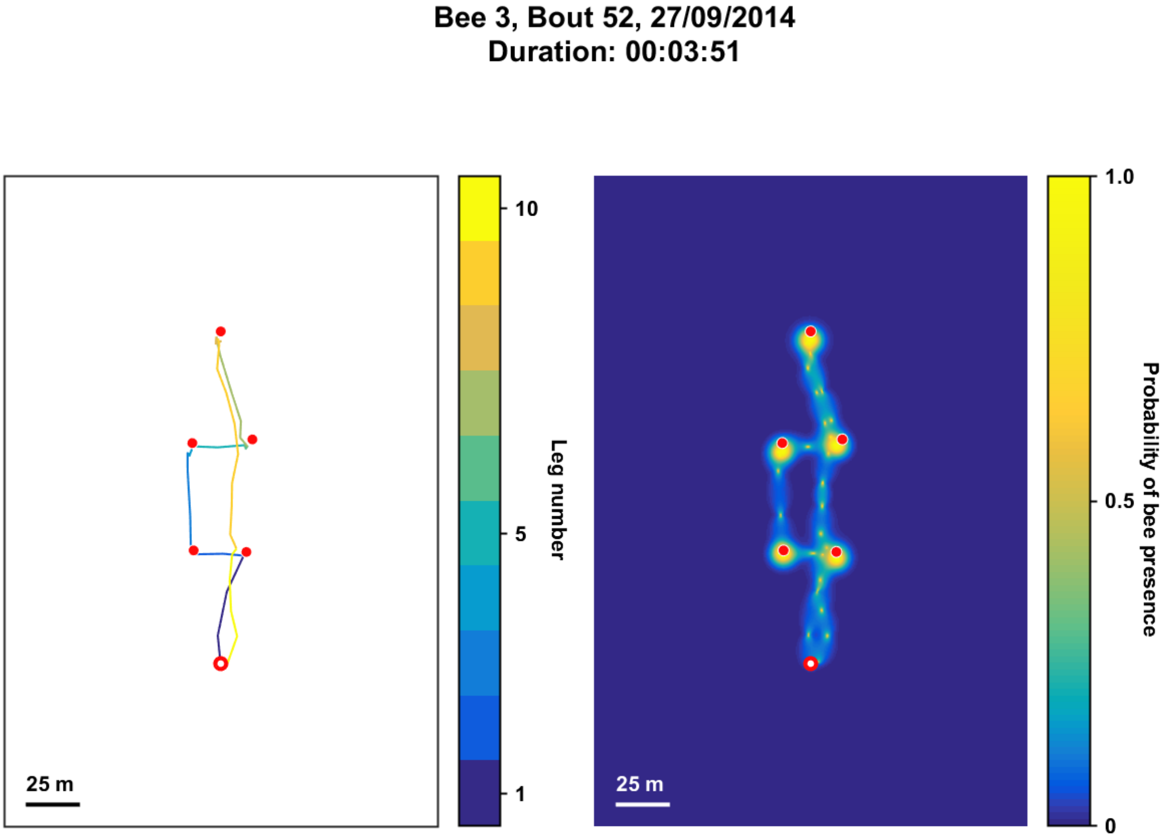

Bee 3, Bout 52, 27/09/2014
Duration: 00:03:51
25 m
10
5
1
Leg number
25 m
1.0
0.5
0
Probability of bee presence

Fig S151

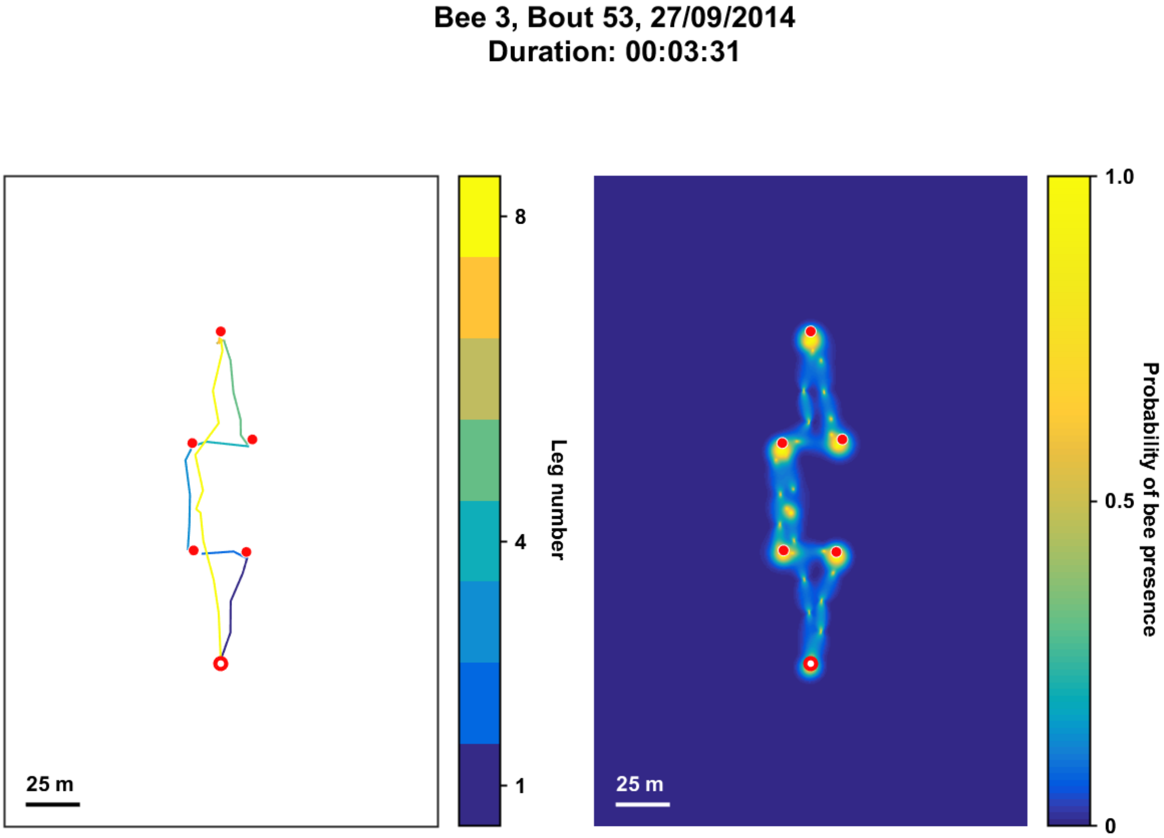
Bee 3, Bout 53, 27/09/2014
Duration: 00:03:31
25 m
8
4
1
Leg number
25 m
1.0
0.5
0
Probability of bee presence

Fig S152

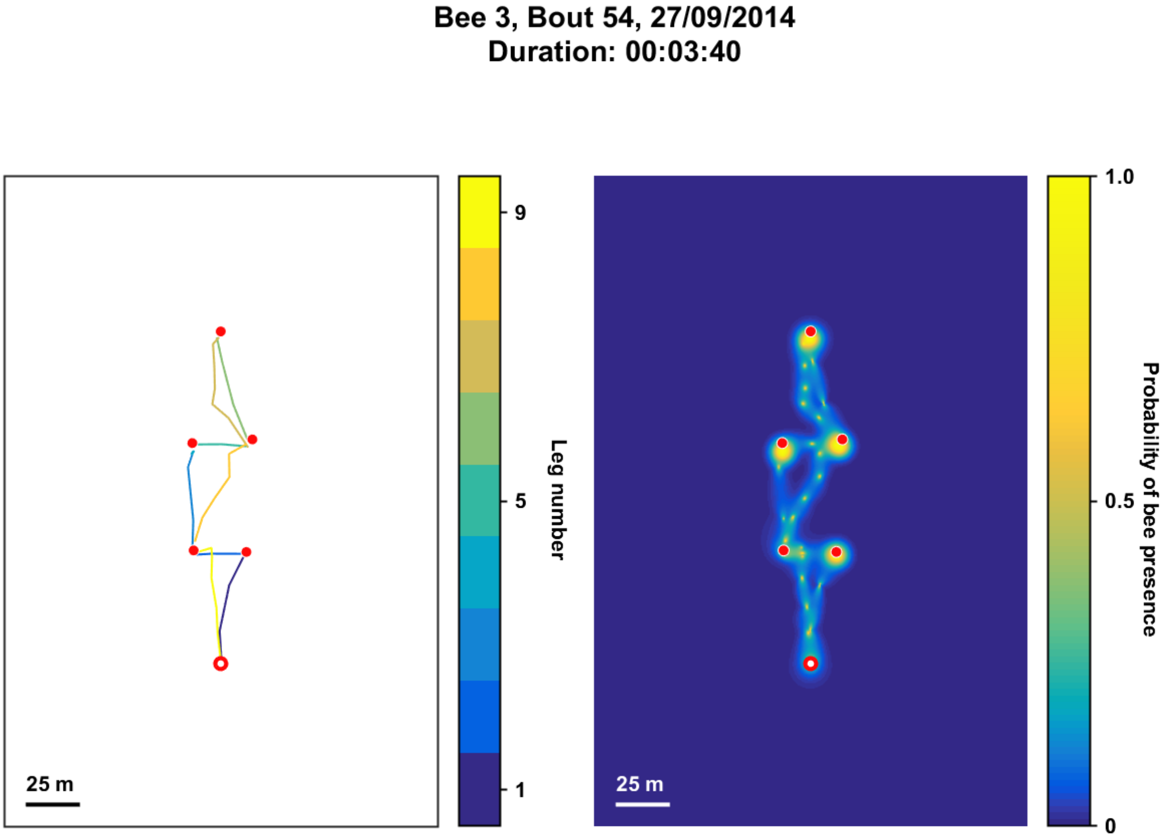
Bee 3, Bout 54, 27/09/2014
Duration: 00:03:40
25 m
9
5
1
Leg number
25 m
1.0
0.5
0
Probability of bee presence

Fig S153

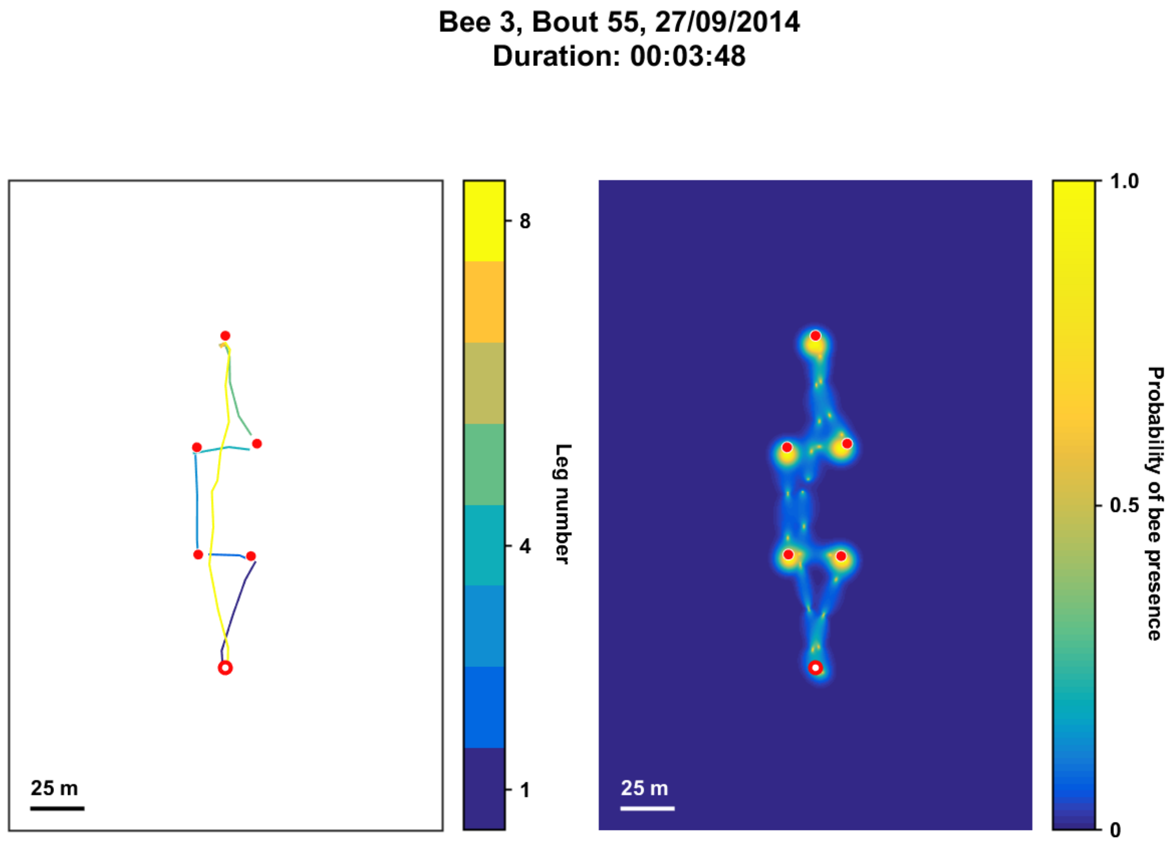
Bee 3, Bout 55, 27/09/2014
Duration: 00:03:48
8
4
1
Leg number
25 m
1.0
0.5
0
Probability of bee presence
25 m

Fig S154

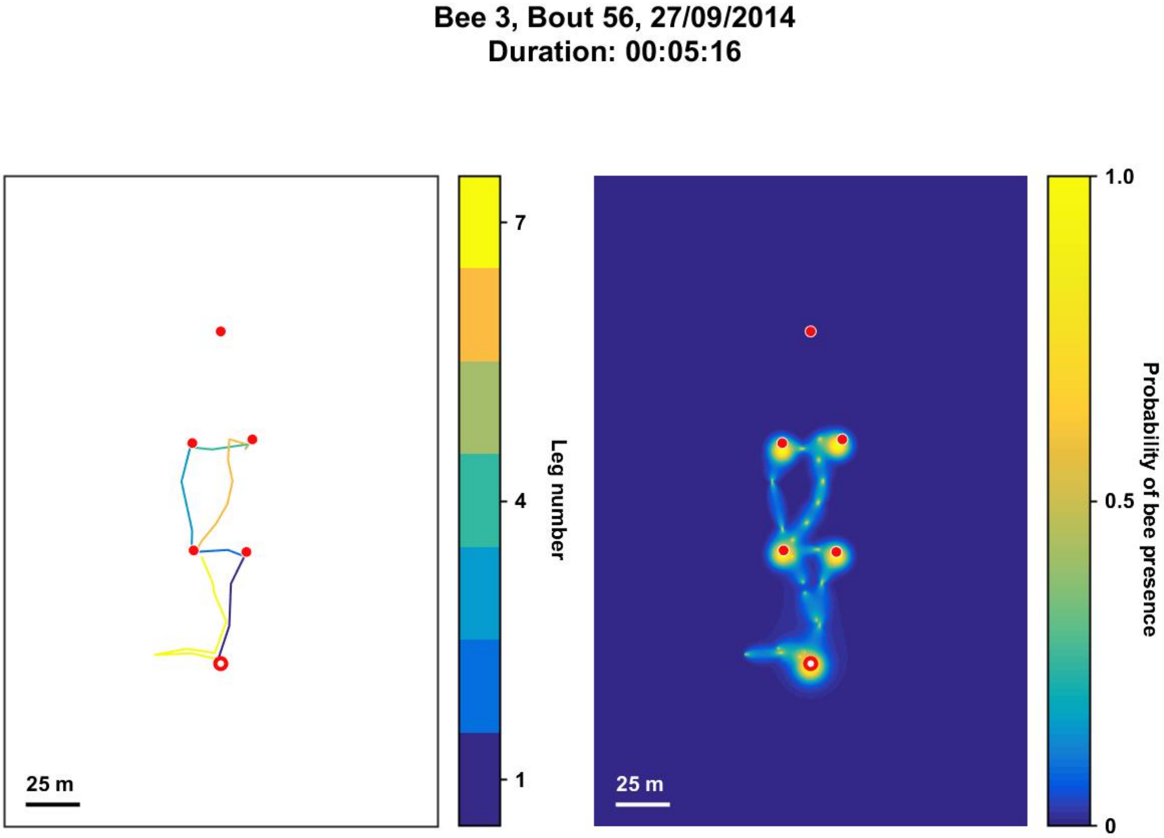

Bee 3, Bout 56, 27/09/2014
Duration: 00:05:16
25 m
7
4
1
Leg number
25 m
1.0
0.5
0
Probability of bee presence

Fig S155

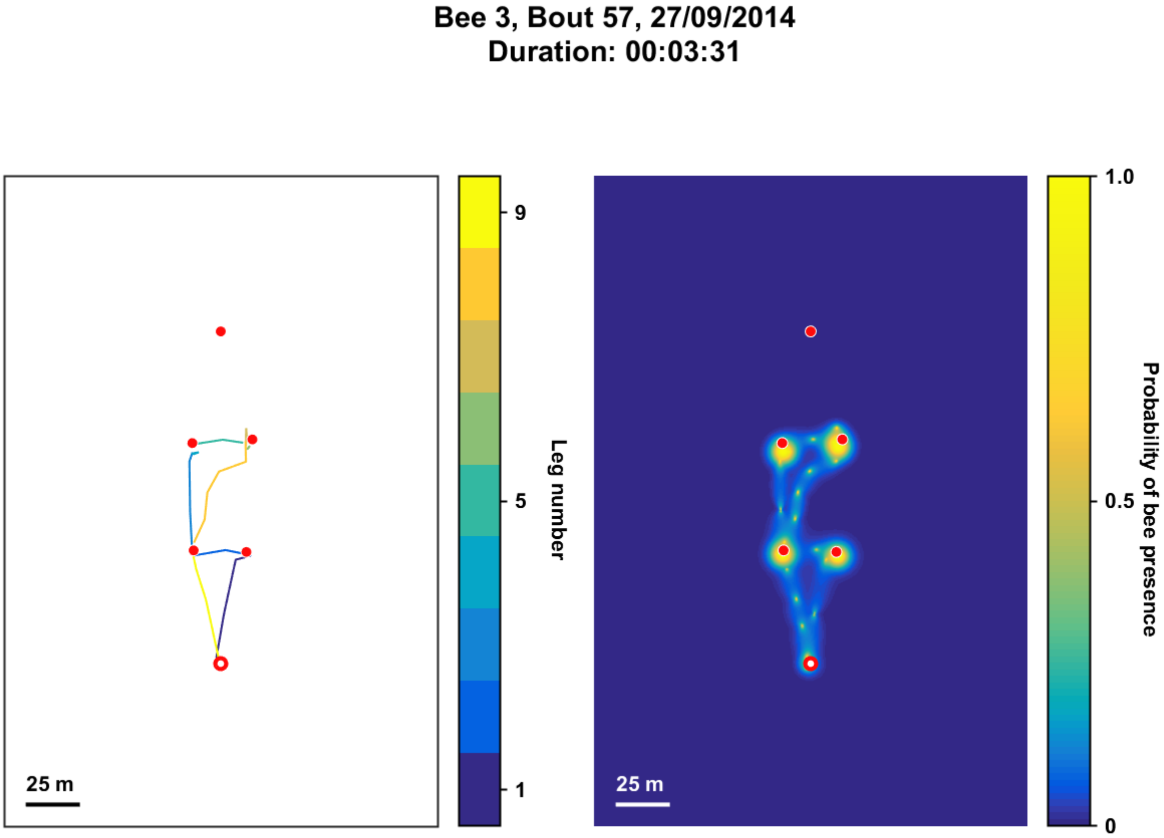
Bee 3, Bout 57, 27/09/2014
Duration: 00:03:31
25 m
9
5
1
Leg number
25 m
1.0
0.5
0
Probability of bee presence

Fig S156

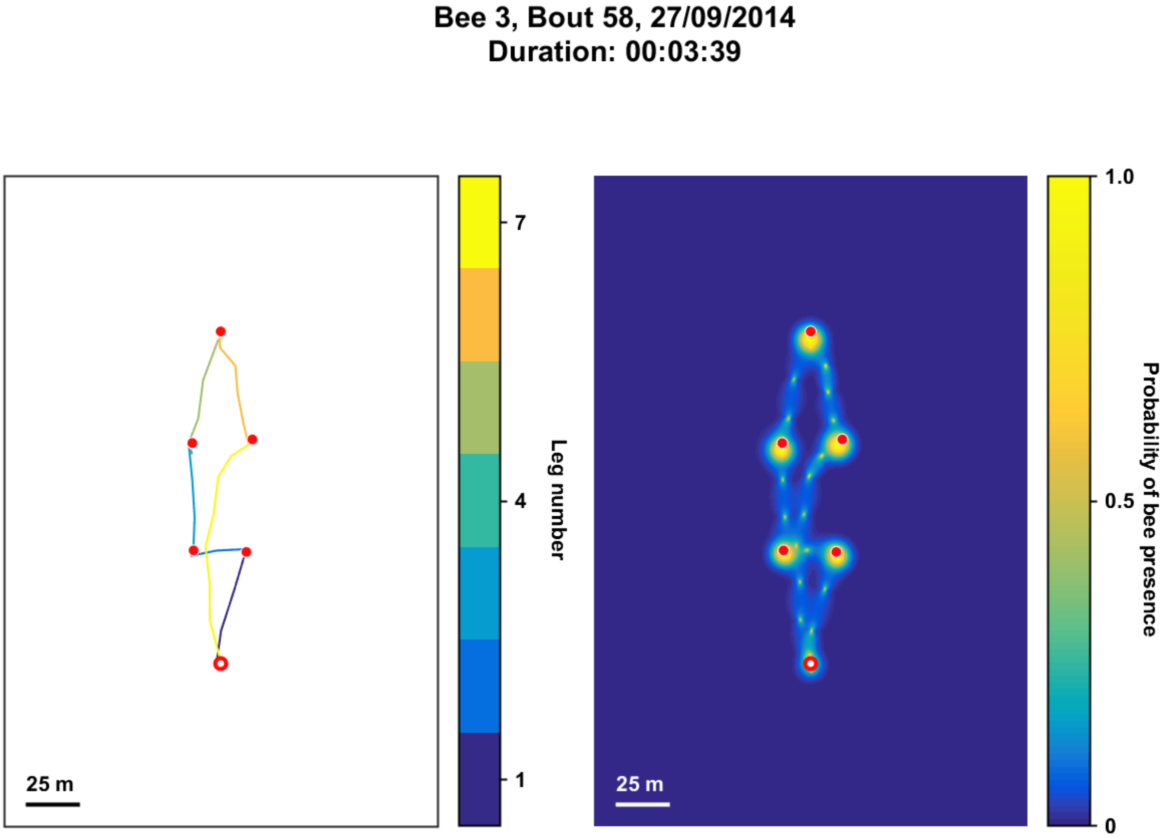

Bee 3, Bout 58, 27/09/2014
Duration: 00:03:39
25 m
7
4
1
Leg number
25 m
1.0
0.5
0
Probability of bee presence

Fig S157

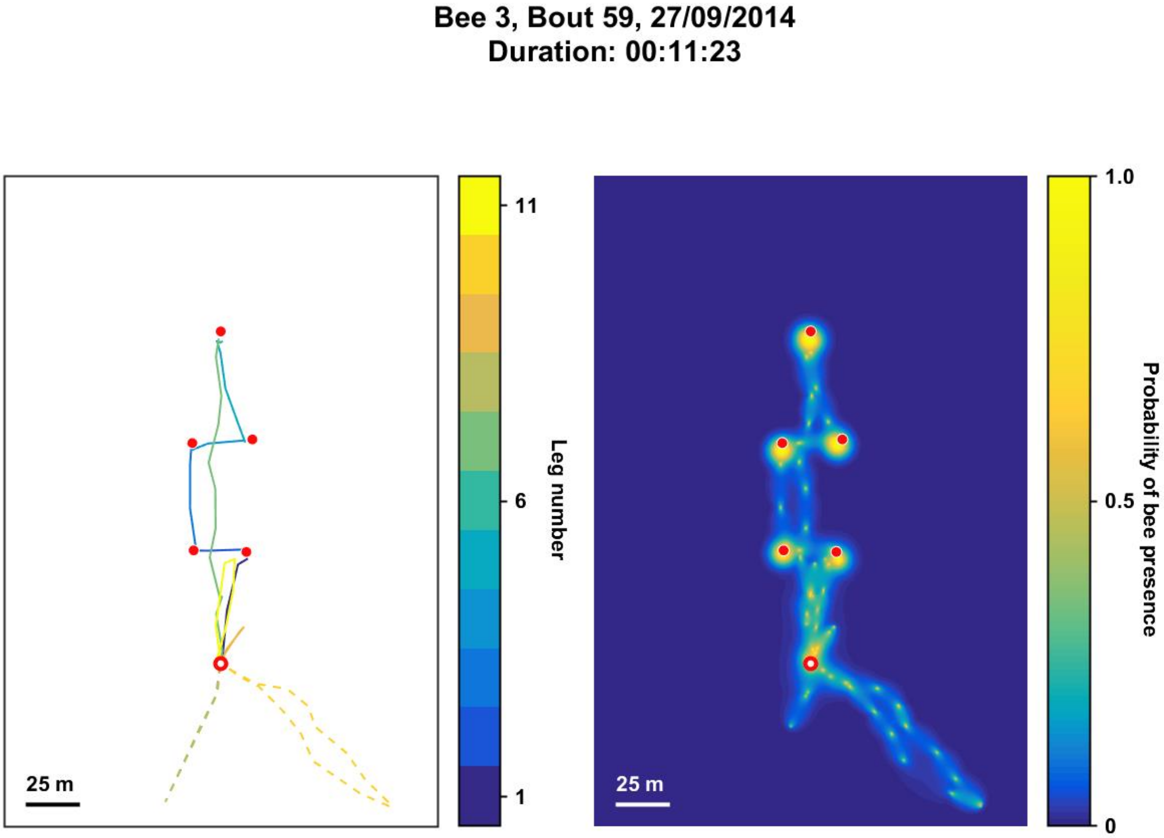

Bee 3, Bout 59, 27/09/2014
Duration: 00:11:23
25 m
11
6
1
Leg number
25 m
1.0
0.5
0
Probability of bee presence

Fig S158

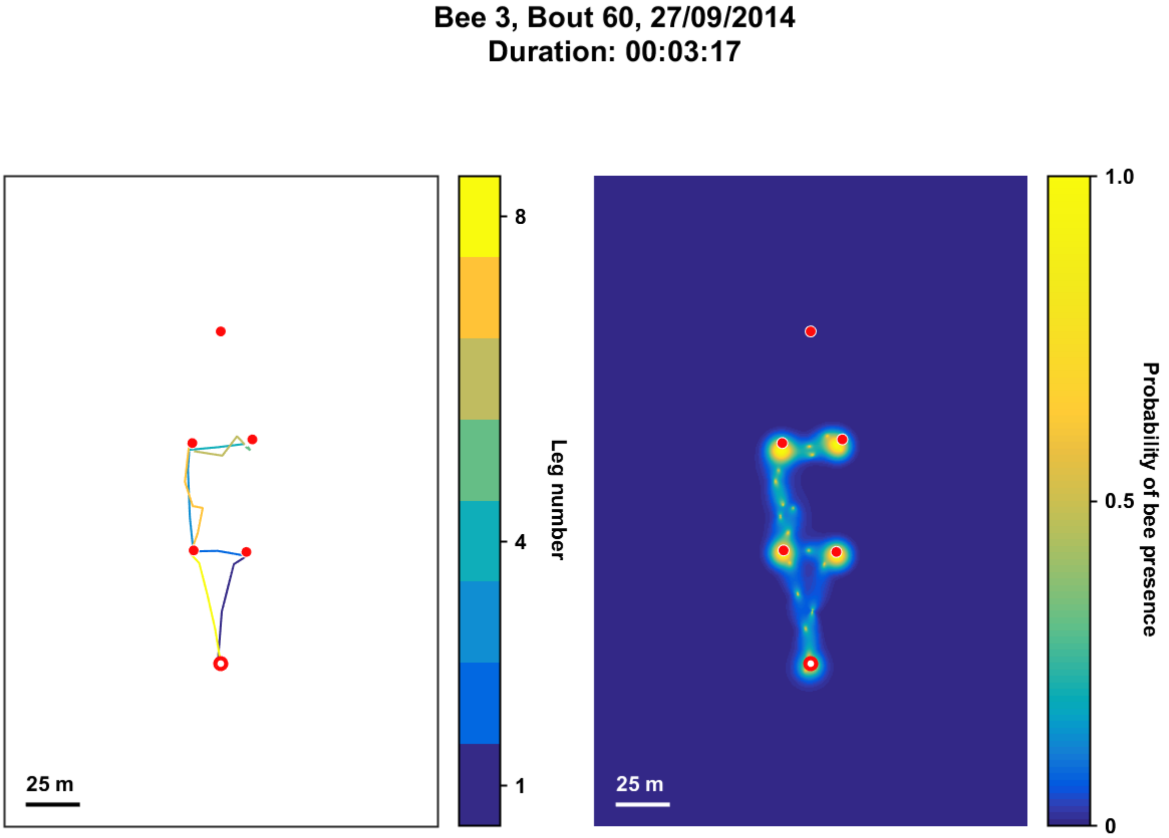

Bee 3, Bout 60, 27/09/2014
Duration: 00:03:17
25 m
8
4
1
Leg number
25 m
1.0
0.5
0
Probability of bee presence

Fig S159

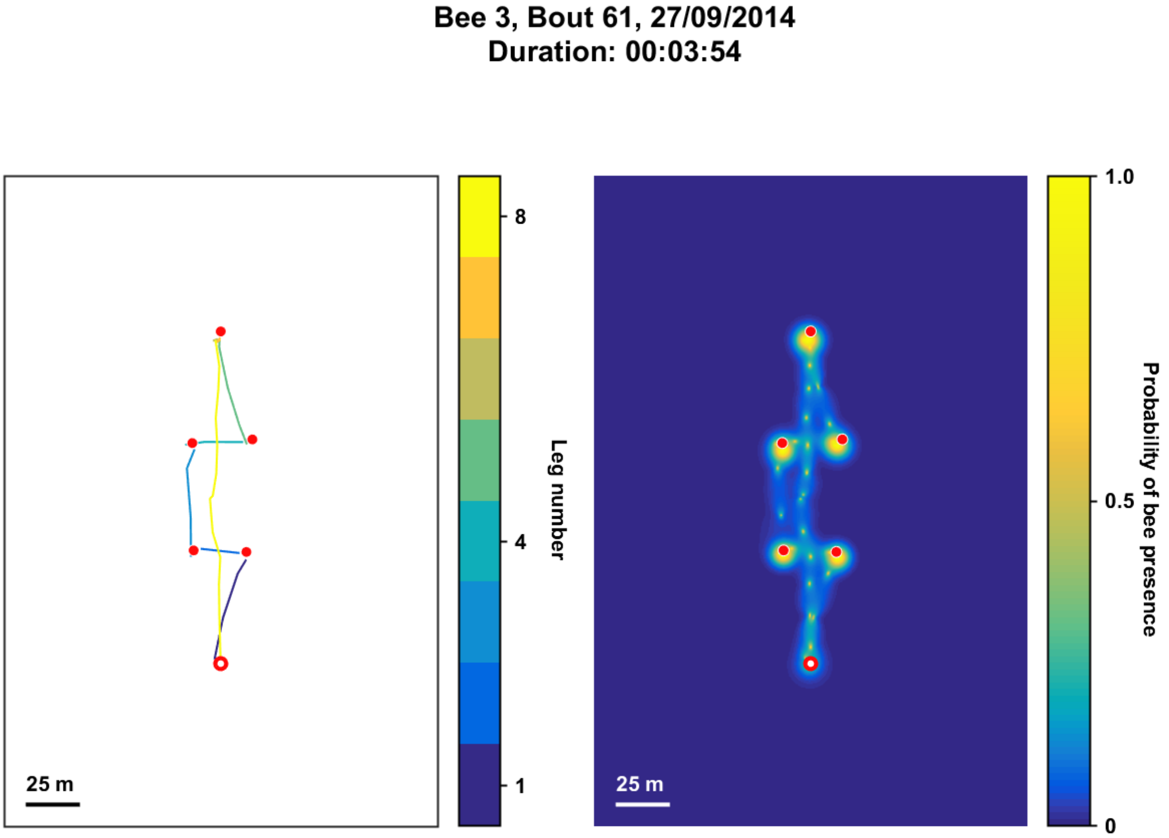
Bee 3, Bout 61, 27/09/2014
Duration: 00:03:54
25 m
Leg number
8
4
1
25 m
Probability of bee presence
1.0
0.5
0

Fig S160

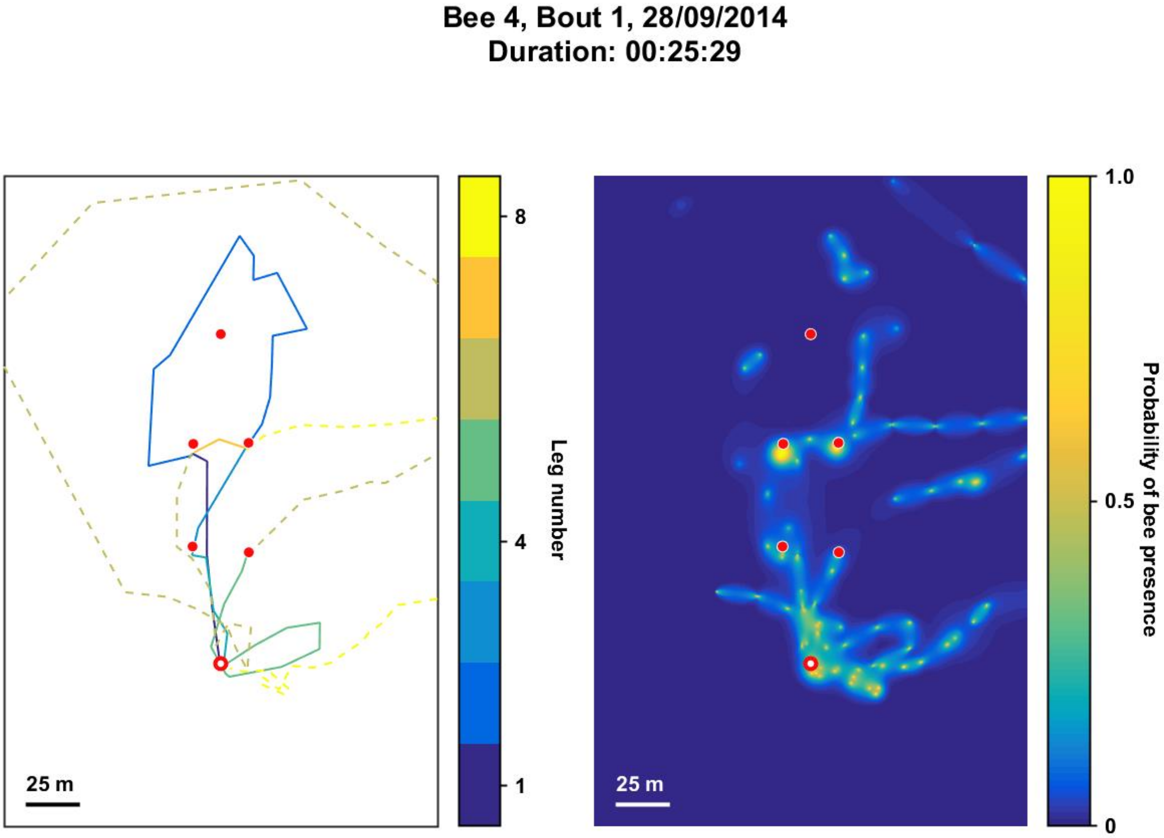
Bee 4, Bout 1, 28/09/2014
Duration: 00:25:29
25 m
Leg number
8
4
1
25 m
Probability of bee presence
1.0
0.5
0

Fig S162

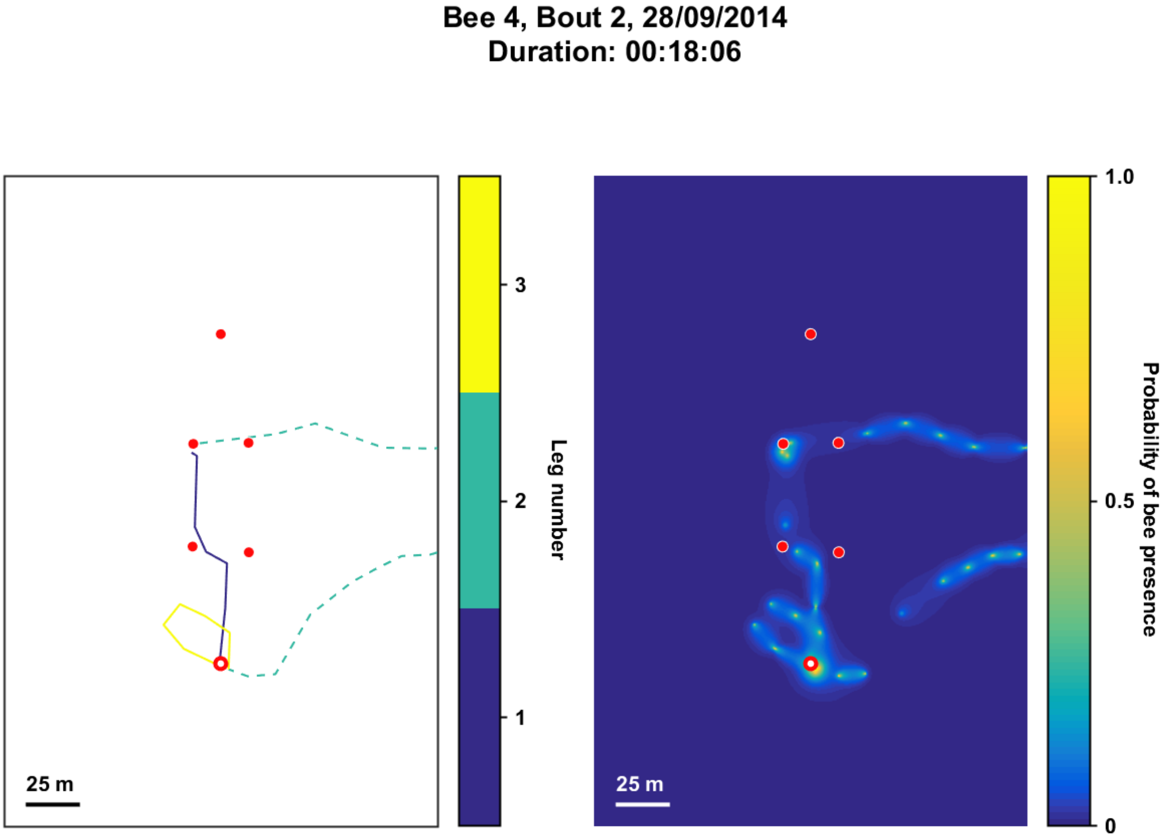

Bee 4, Bout 2, 28/09/2014
Duration: 00:18:06
25 m
Leg number
1
2
3
25 m
Probability of bee presence
0
0.5
1.0

Fig S163

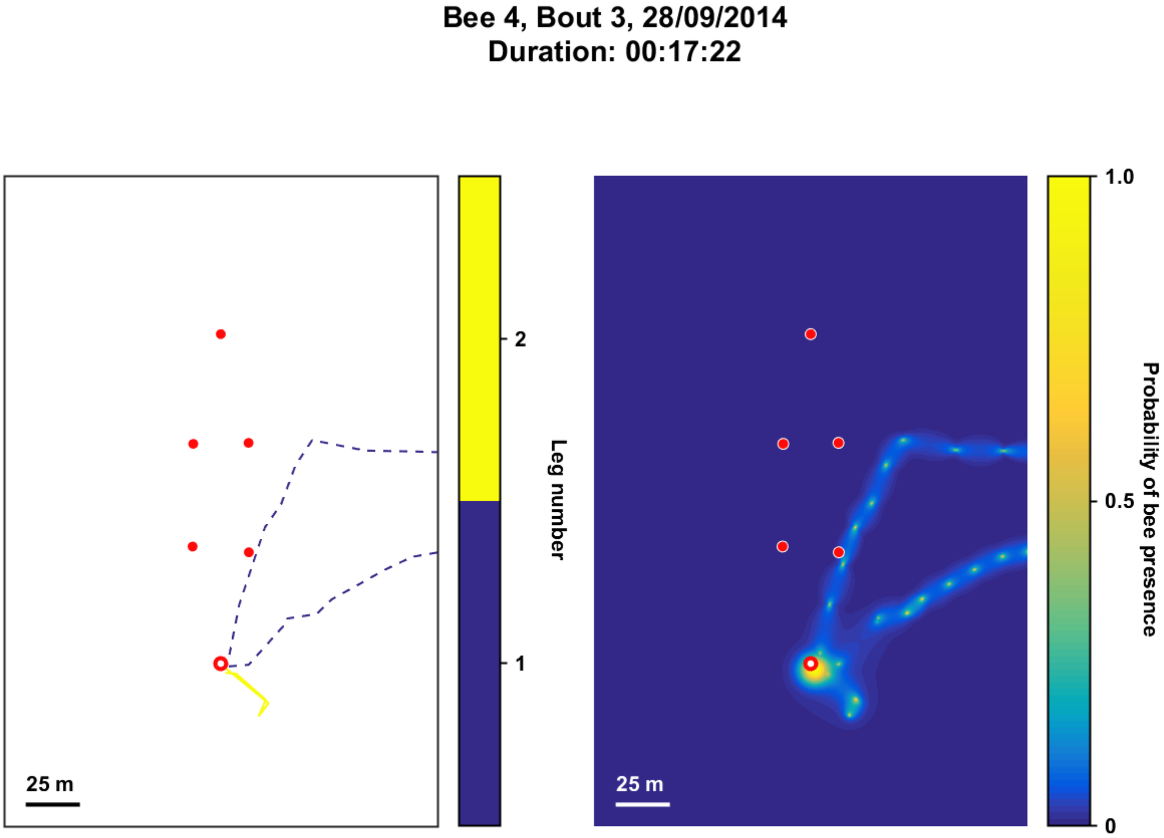
Bee 4, Bout 3, 28/09/2014
Duration: 00:17:22
25 m
2
1
Leg number
25 m
1.0
0.5
0
Probability of bee presence

Fig S164

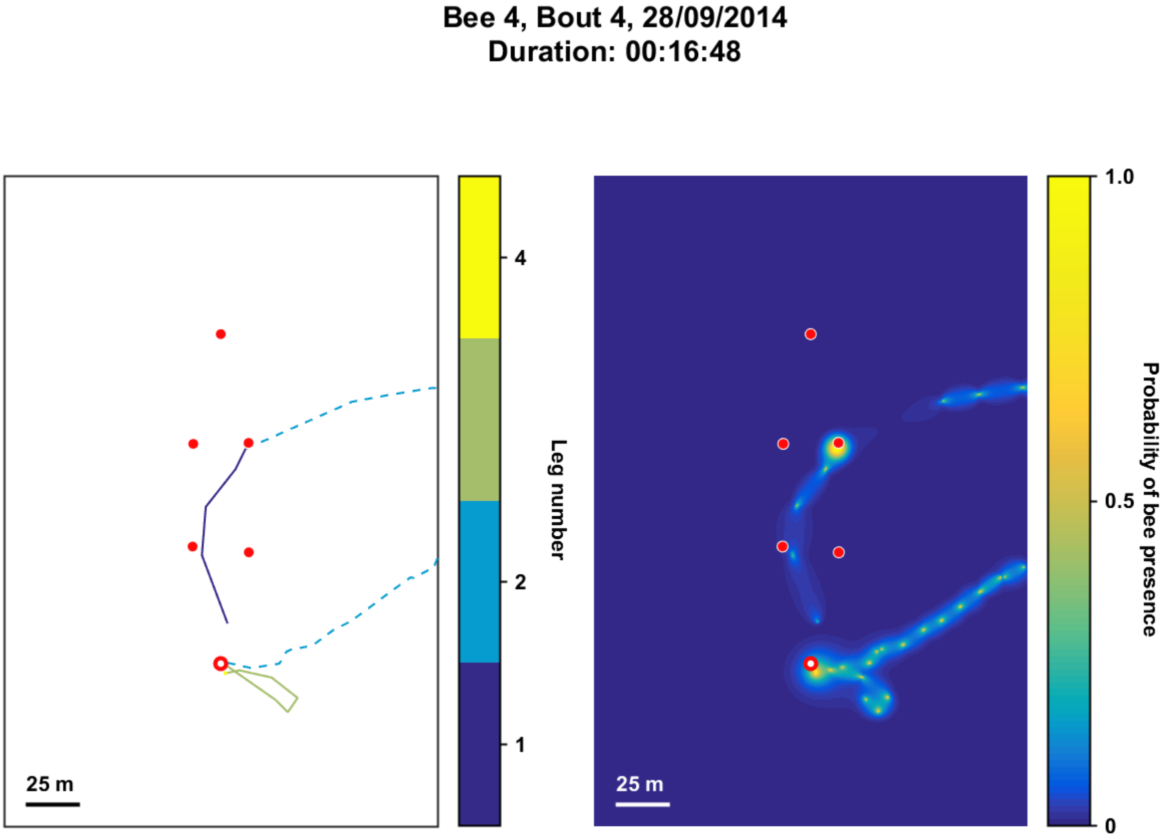

Bee 4, Bout 4, 28/09/2014
Duration: 00:16:48
25 m
Leg number
4
2
1
25 m
1.0
0.5
0
Probability of bee presence

Fig S165

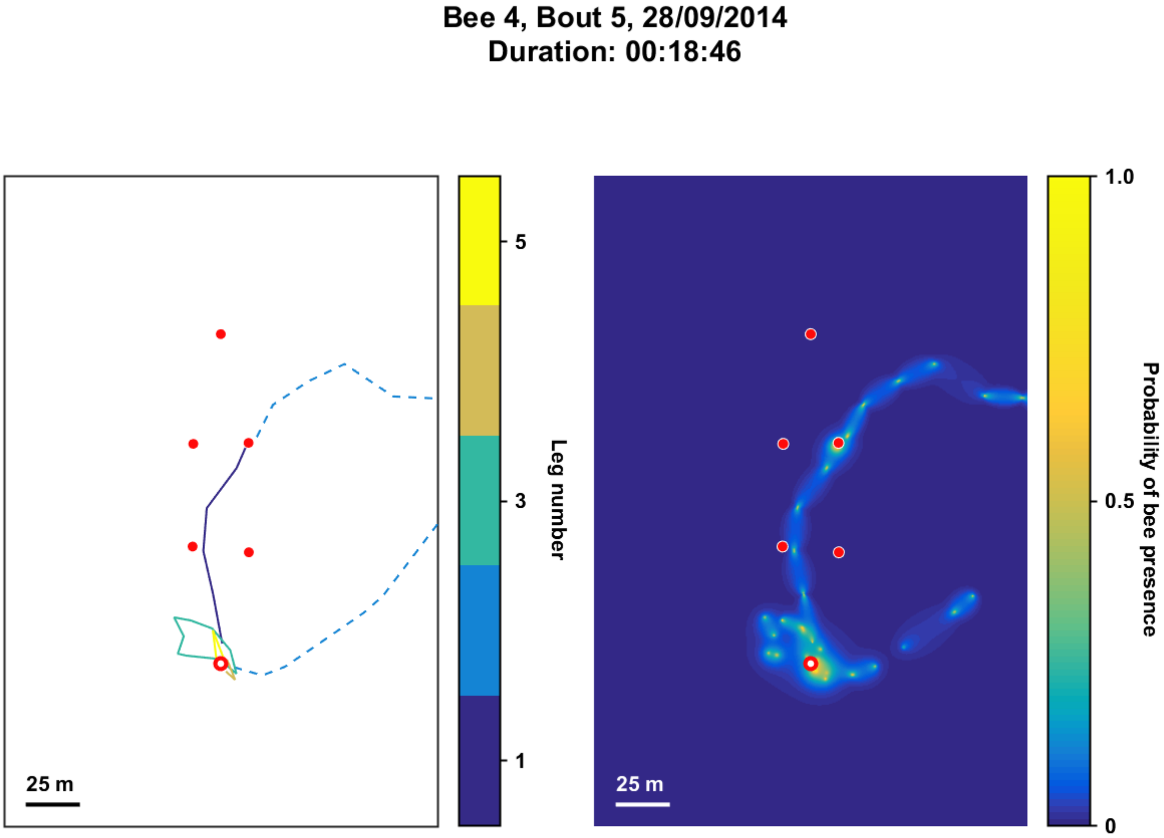

Bee 4, Bout 5, 28/09/2014
Duration: 00:18:46
25 m
Leg number
1
3
5
25 m
Probability of bee presence
0
0.5
1.0

Fig S166

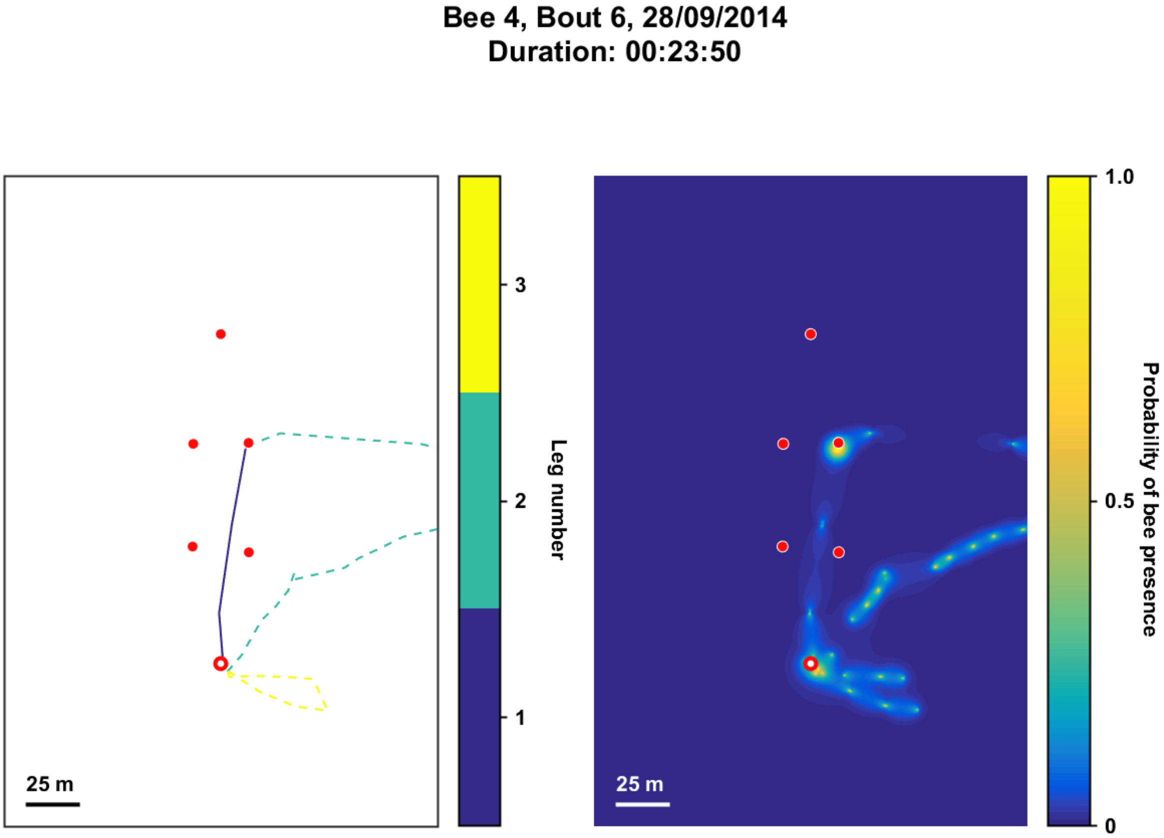

Bee 4, Bout 6, 28/09/2014
Duration: 00:23:50
25 m
Leg number
1
2
3
25 m
Probability of bee presence
0
0.5
1.0

Fig S167

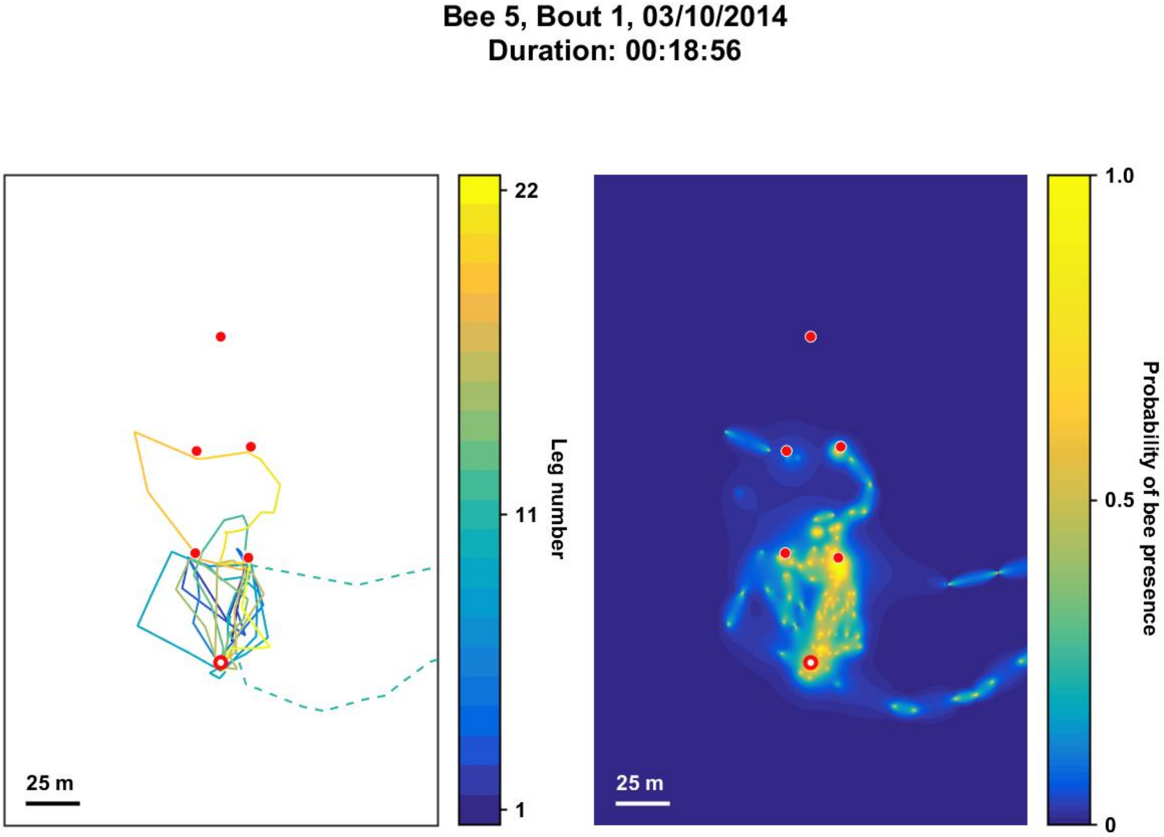
Bee 5, Bout 1, 03/10/2014
Duration: 00:18:56
22
11
1
Leg number
25 m
1.0
0.5
0
Probability of bee presence
25 m

Fig S168

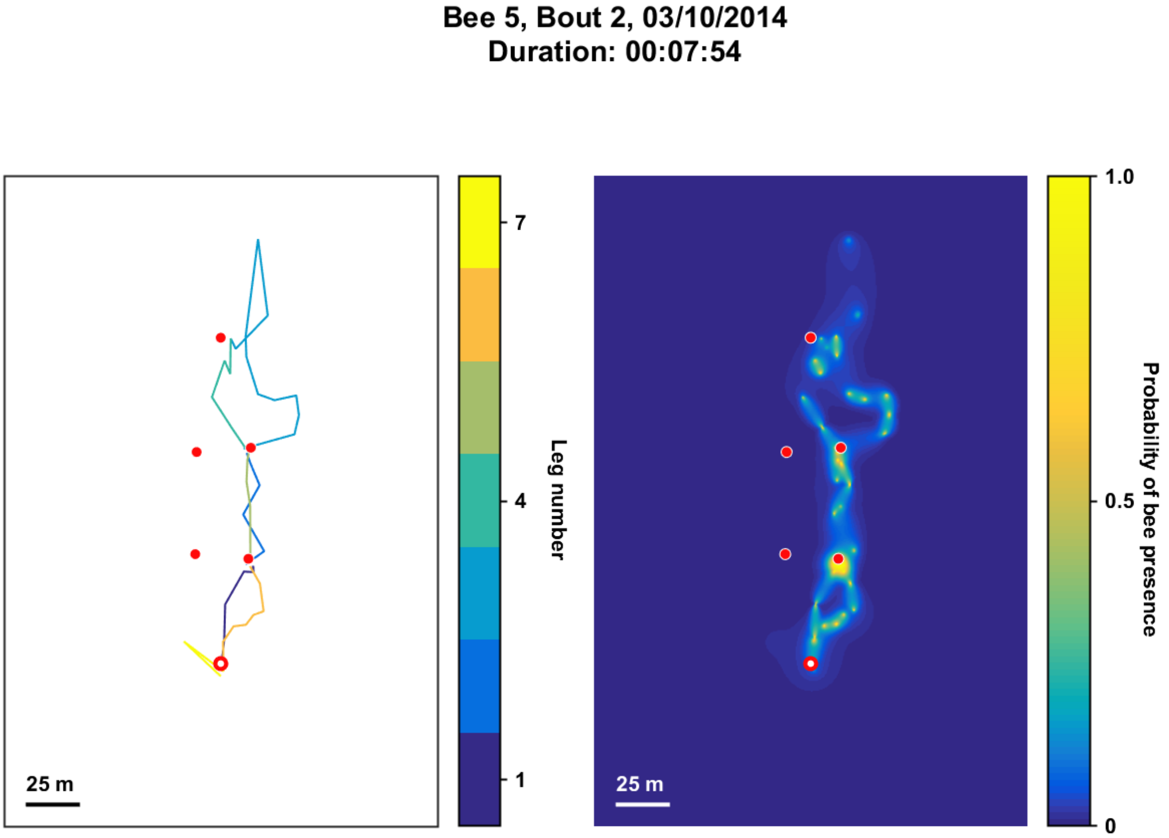
Bee 5, Bout 2, 03/10/2014
Duration: 00:07:54
25 m
7
4
1
Leg number
25 m
1.0
0.5
0
Probability of bee presence

Fig S169

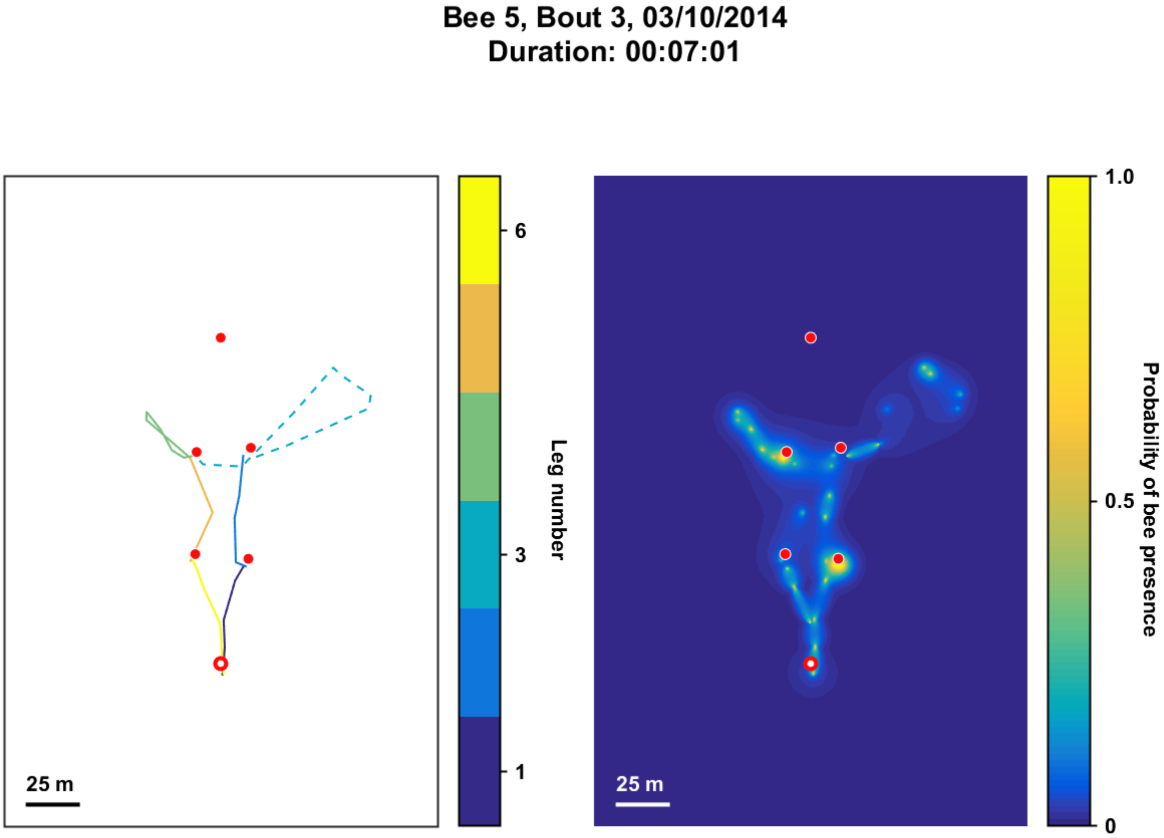
Bee 5, Bout 3, 03/10/2014
Duration: 00:07:01
25 m
6
3
1
Leg number
25 m
1.0
0.5
0
Probability of bee presence

Fig S170

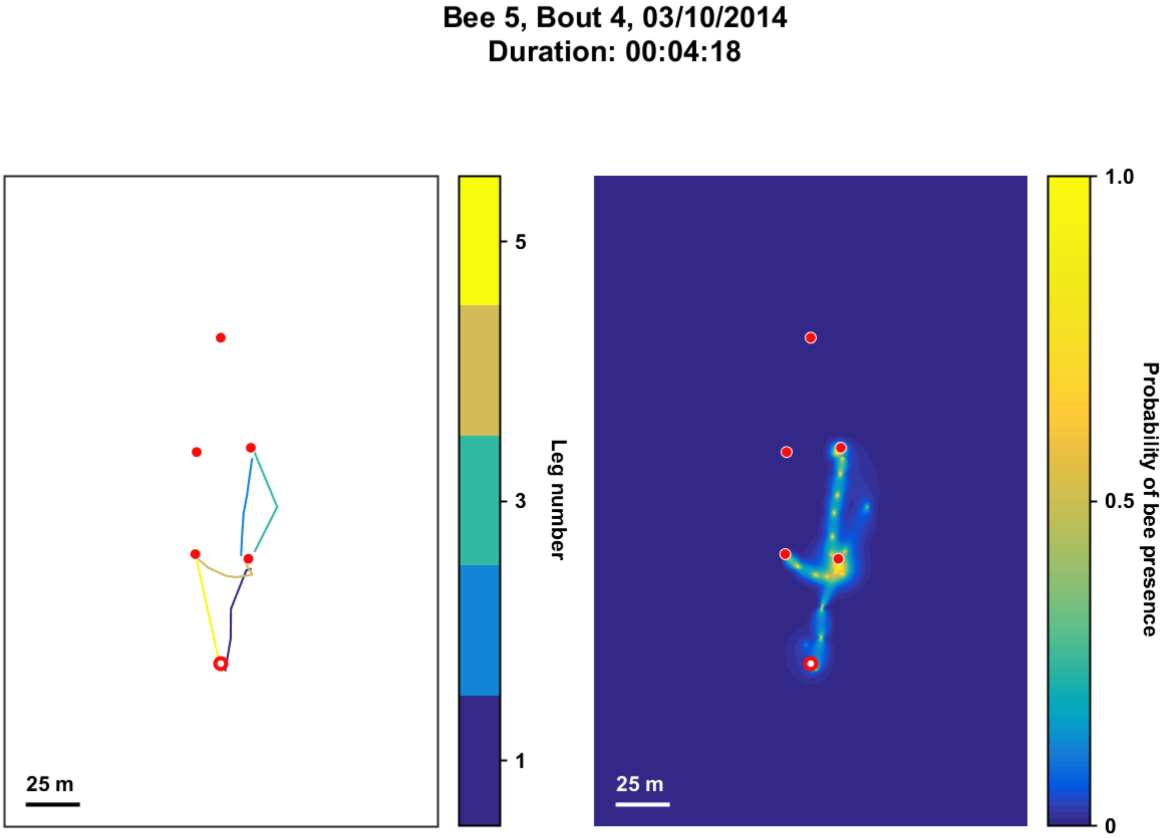

Bee 5, Bout 4, 03/10/2014
Duration: 00:04:18
25 m
Leg number
5
3
1
25 m
Probability of bee presence
1.0
0.5
0

Fig S171

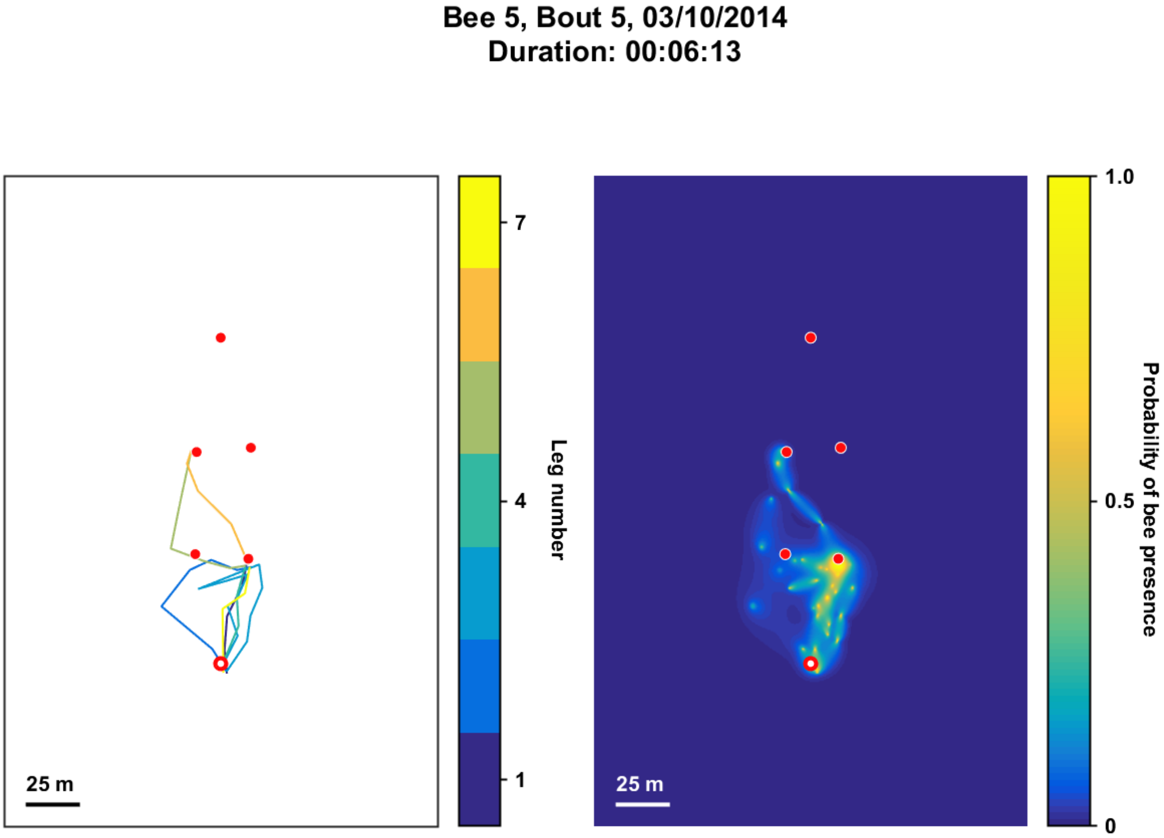
Bee 5, Bout 5, 03/10/2014
Duration: 00:06:13
25 m
7
4
1
Leg number
25 m
1.0
0.5
0
Probability of bee presence

Fig S172

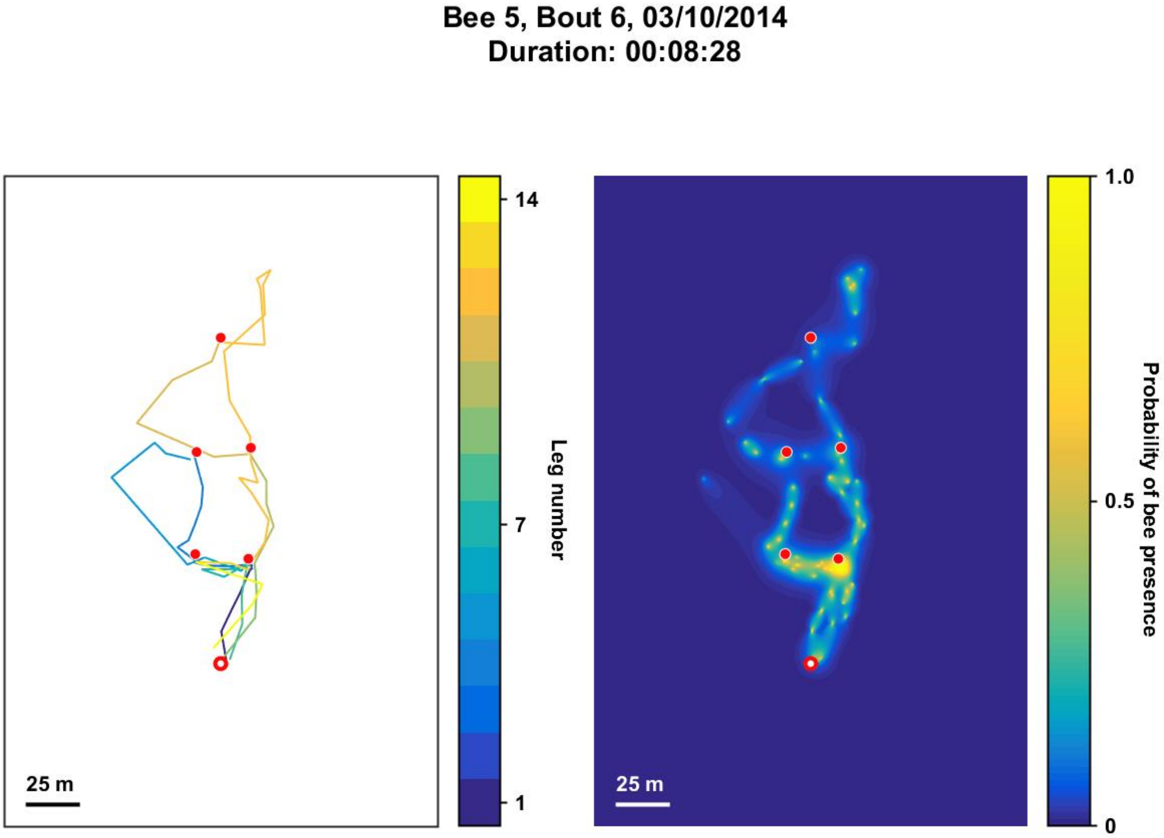
Bee 5, Bout 6, 03/10/2014
Duration: 00:08:28
25 m
Leg number
14
7
1
25 m
Probability of bee presence
1.0
0.5
0

Fig S173

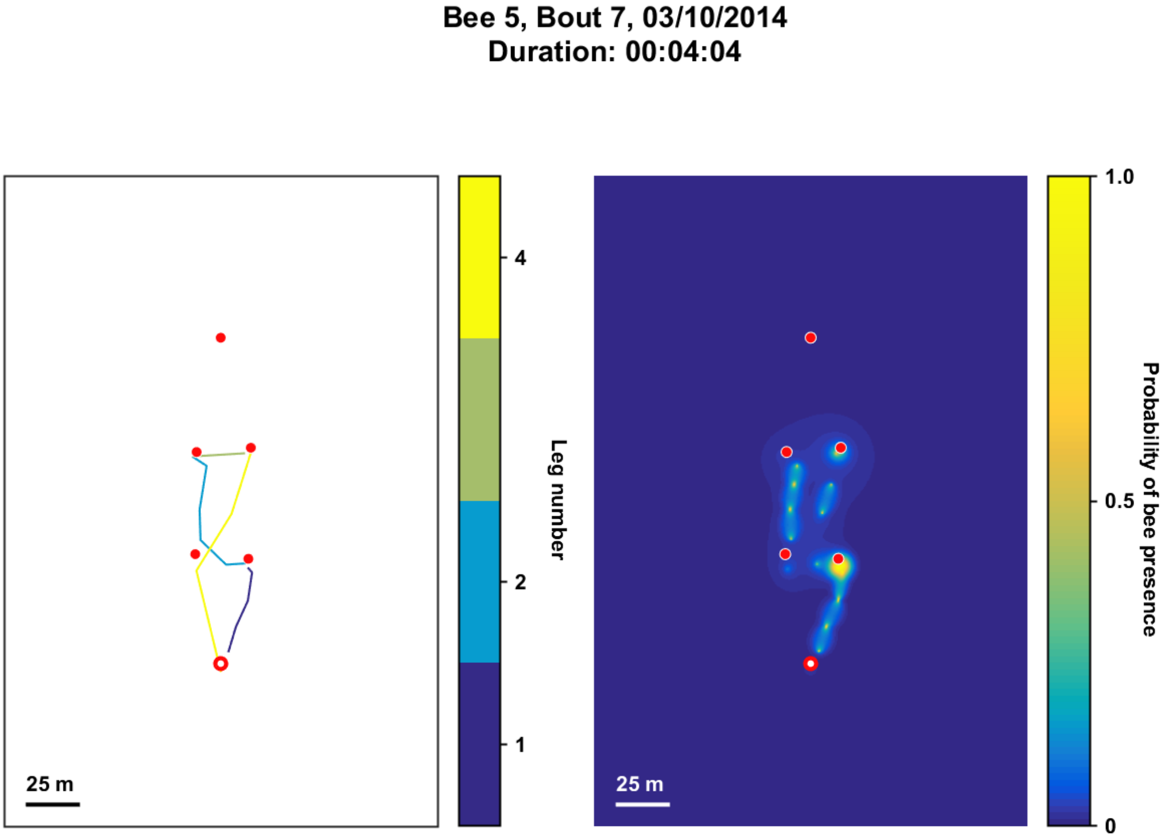

Bee 5, Bout 7, 03/10/2014
Duration: 00:04:04
25 m
4
2
1
Leg number
25 m
1.0
0.5
0
Probability of bee presence

Fig S174

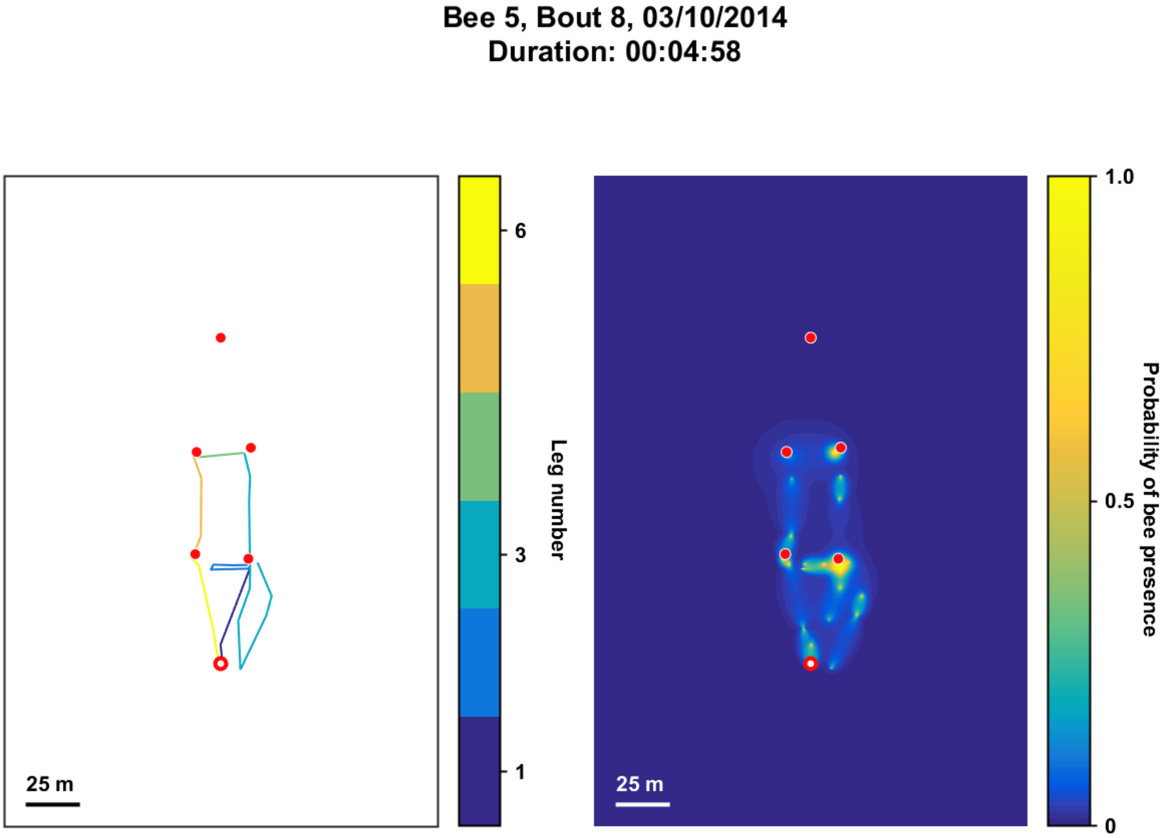

Bee 5, Bout 8, 03/10/2014
Duration: 00:04:58
25 m
Leg number
1
3
6
25 m
Probability of bee presence
0
0.5
1.0

Fig S175

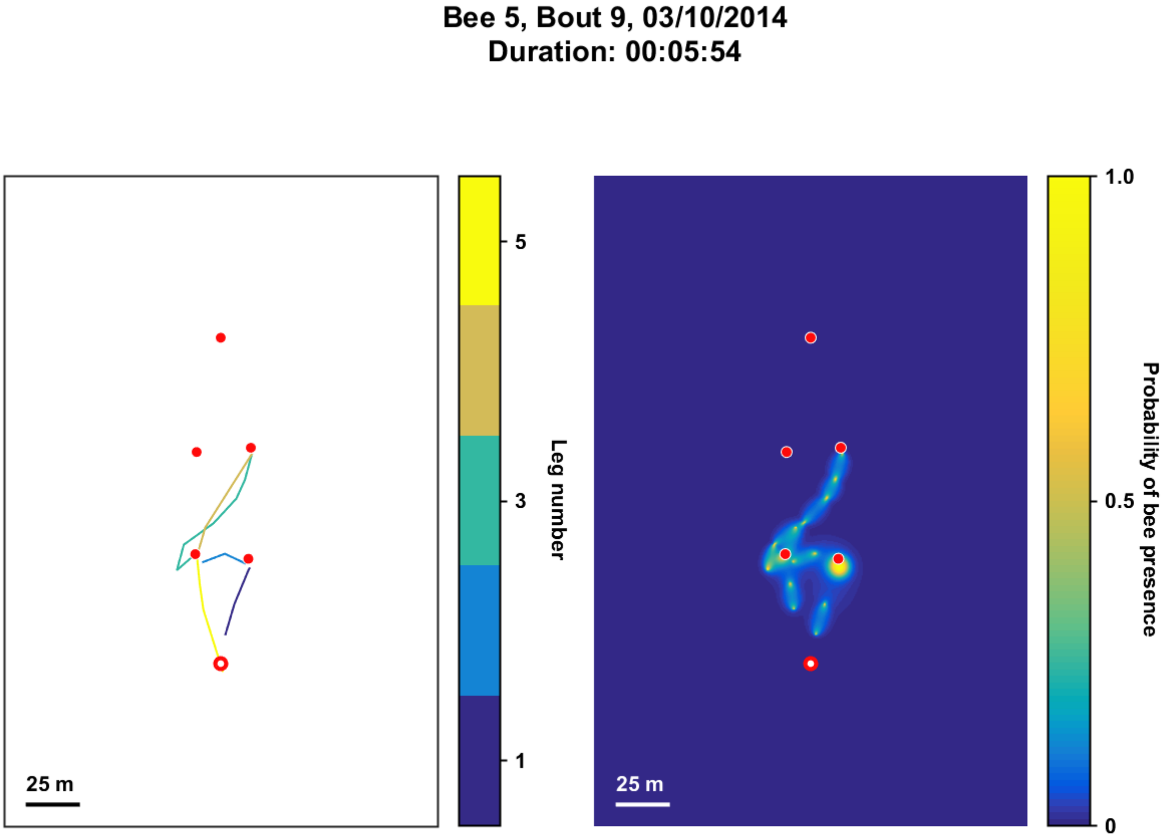
Bee 5, Bout 9, 03/10/2014
Duration: 00:05:54
25 m
5
3
1
Leg number
25 m
1.0
0.5
0
Probability of bee presence

Fig S176

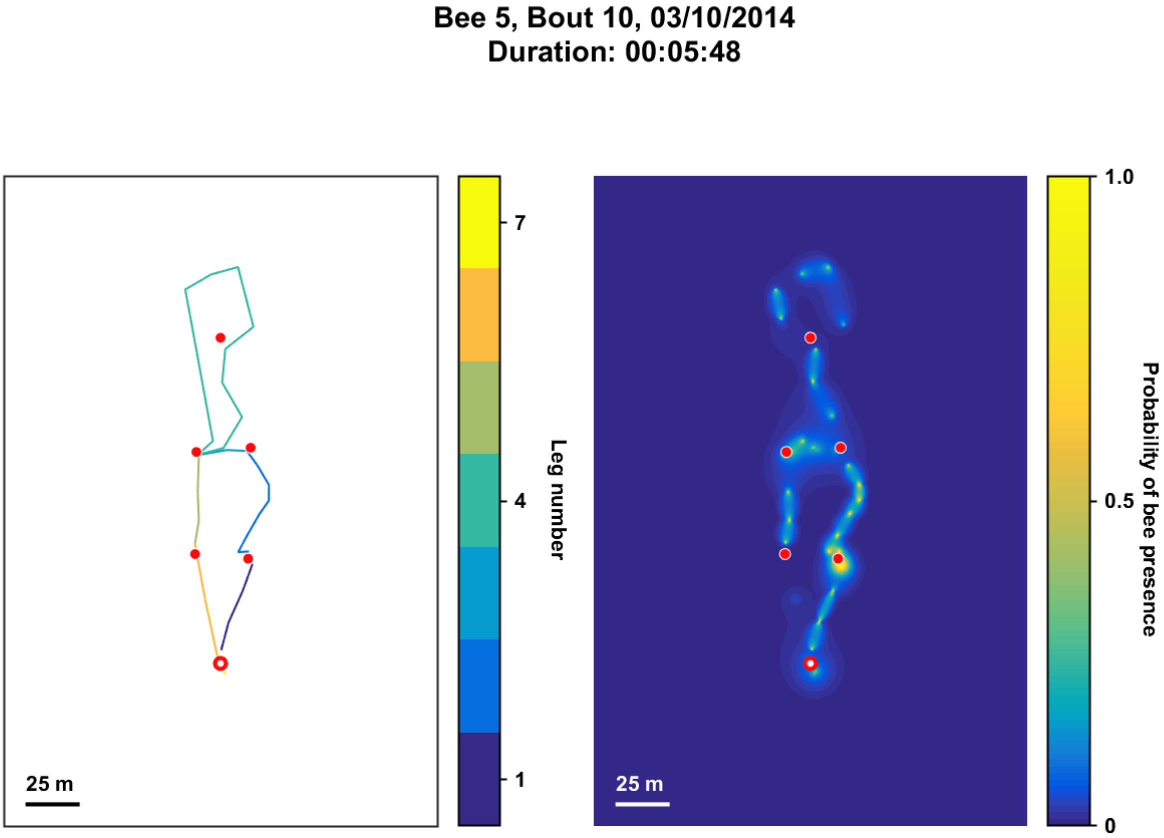

Bee 5, Bout 10, 03/10/2014
Duration: 00:05:48
25 m
7
4
1
Leg number
25 m
1.0
0.5
0
Probability of bee presence

Fig S177

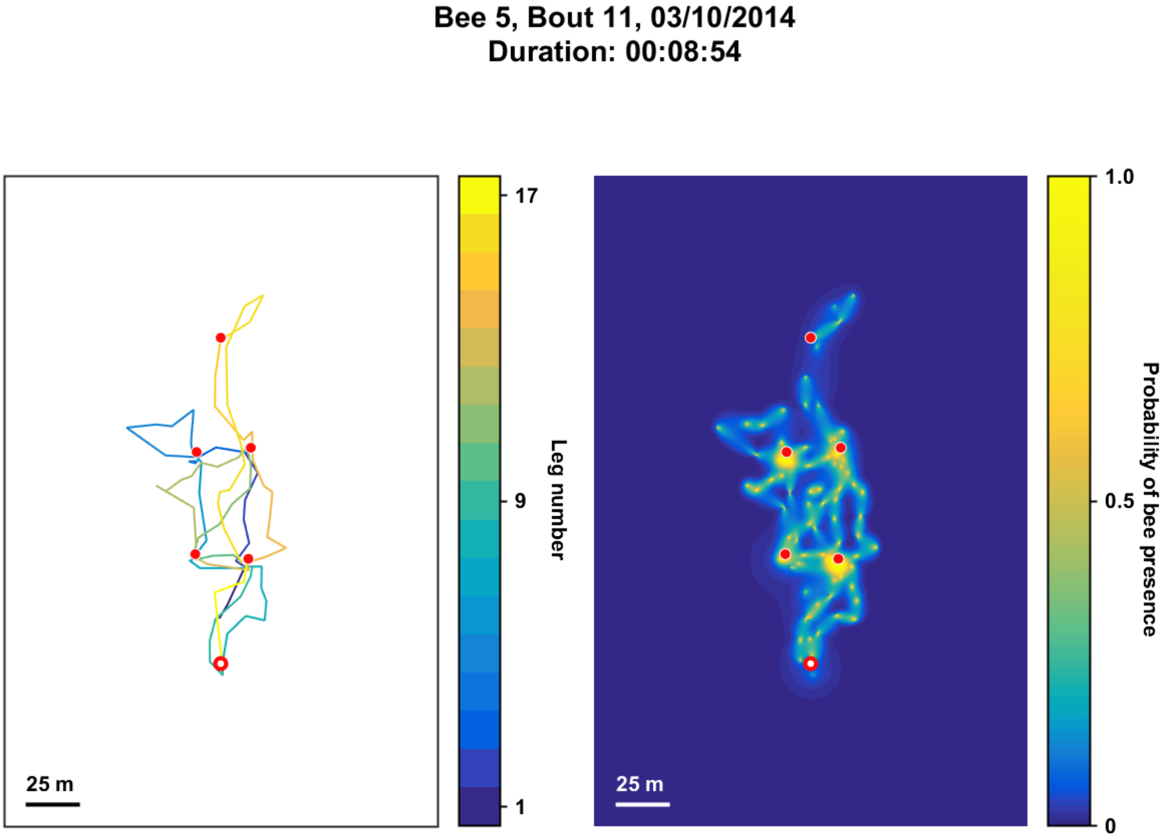

Bee 5, Bout 11, 03/10/2014
Duration: 00:08:54
25 m
17
9
1
Leg number
25 m
1.0
0.5
0
Probability of bee presence

Fig S178

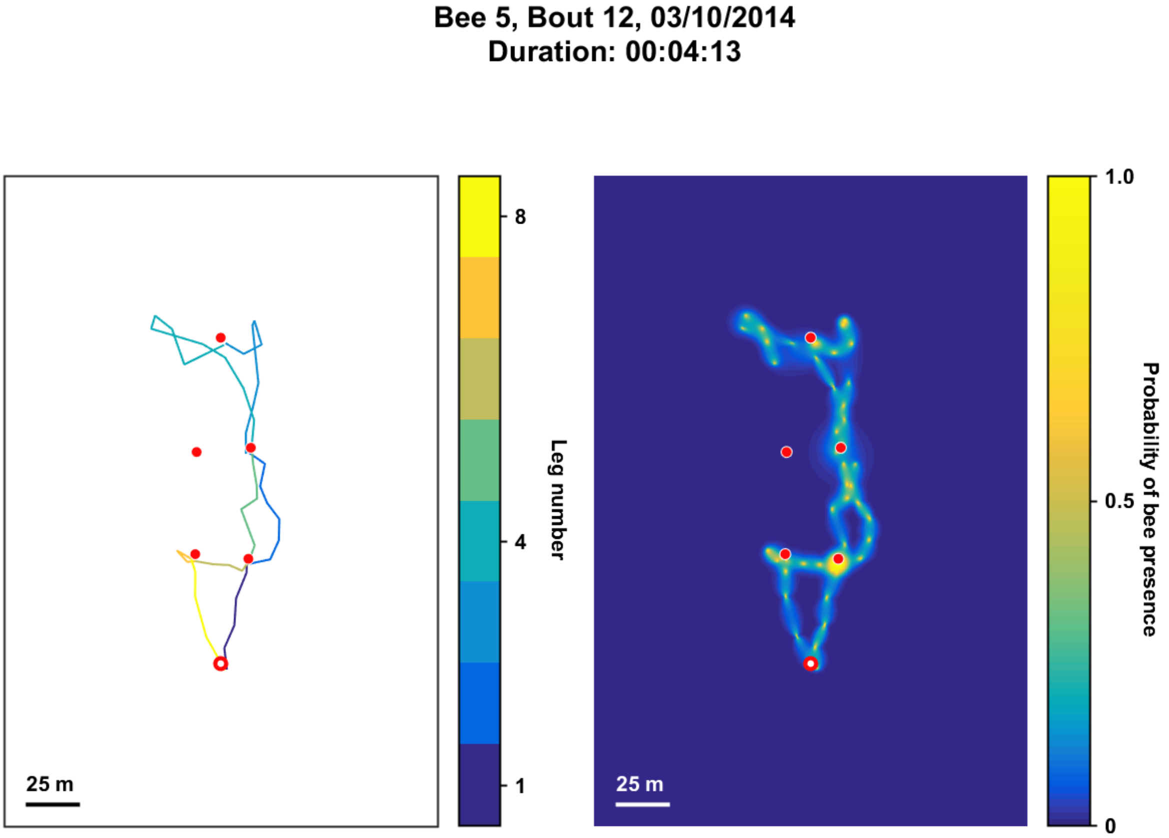
Bee 5, Bout 12, 03/10/2014
Duration: 00:04:13
25 m
8
4
1
Leg number
25 m
1.0
0.5
0
Probability of bee presence

Fig S179

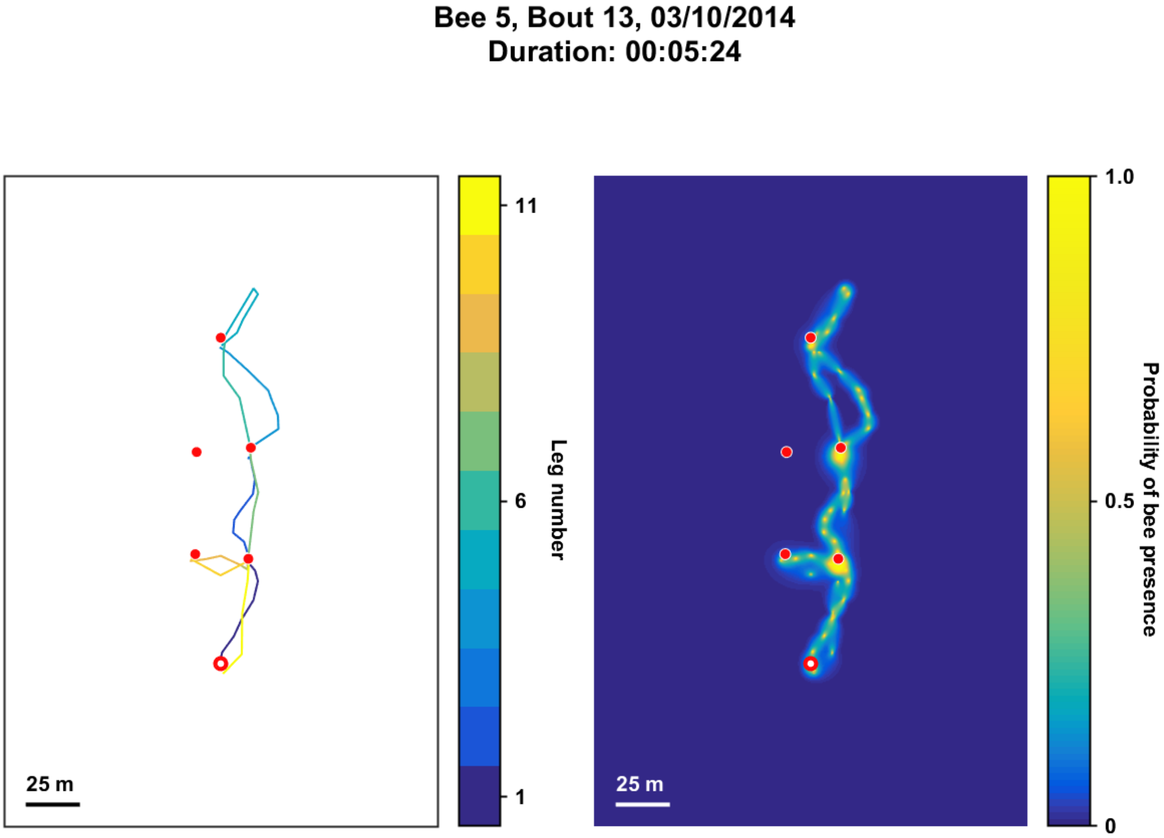
Bee 5, Bout 13, 03/10/2014
Duration: 00:05:24
25 m
11
6
1
Leg number
25 m
1.0
0.5
0
Probability of bee presence

Fig S180

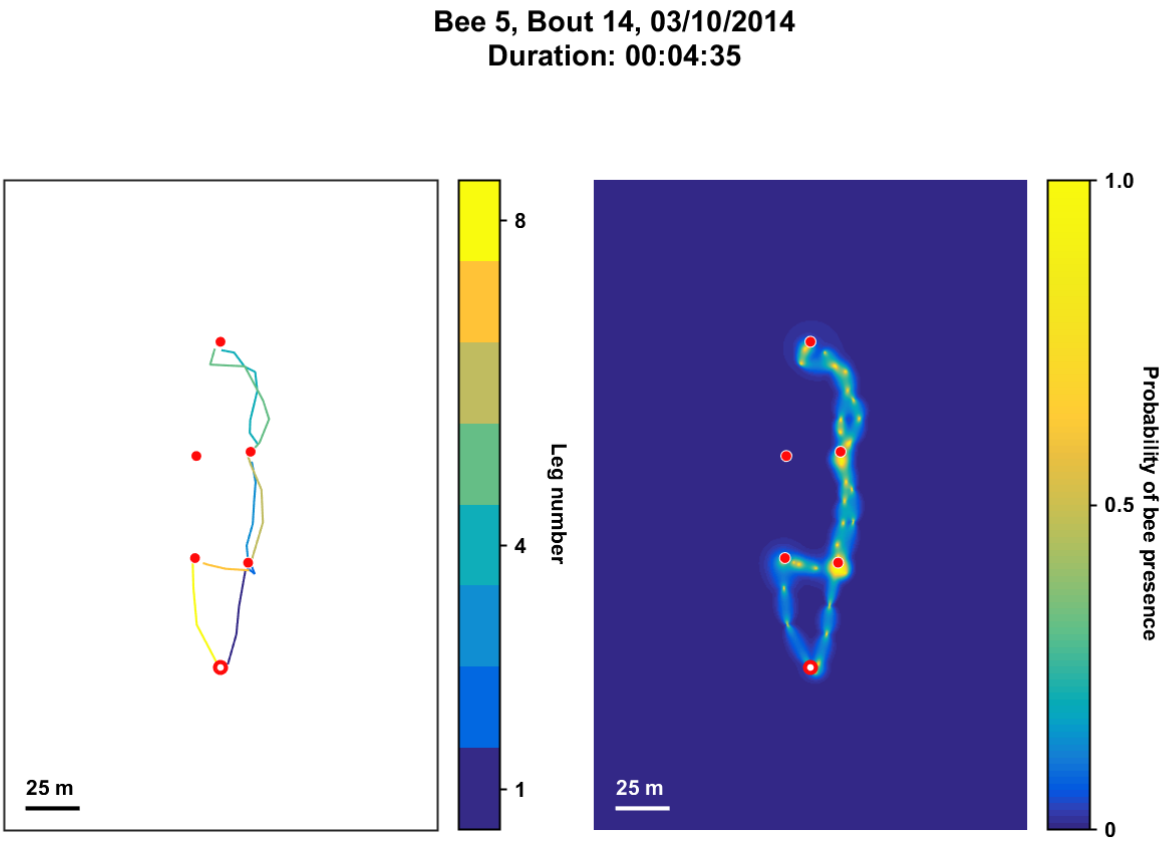

Bee 5, Bout 14, 03/10/2014
Duration: 00:04:35
25 m
Leg number
8
4
1
25 m
Probability of bee presence
1.0
0.5
0

Fig S181

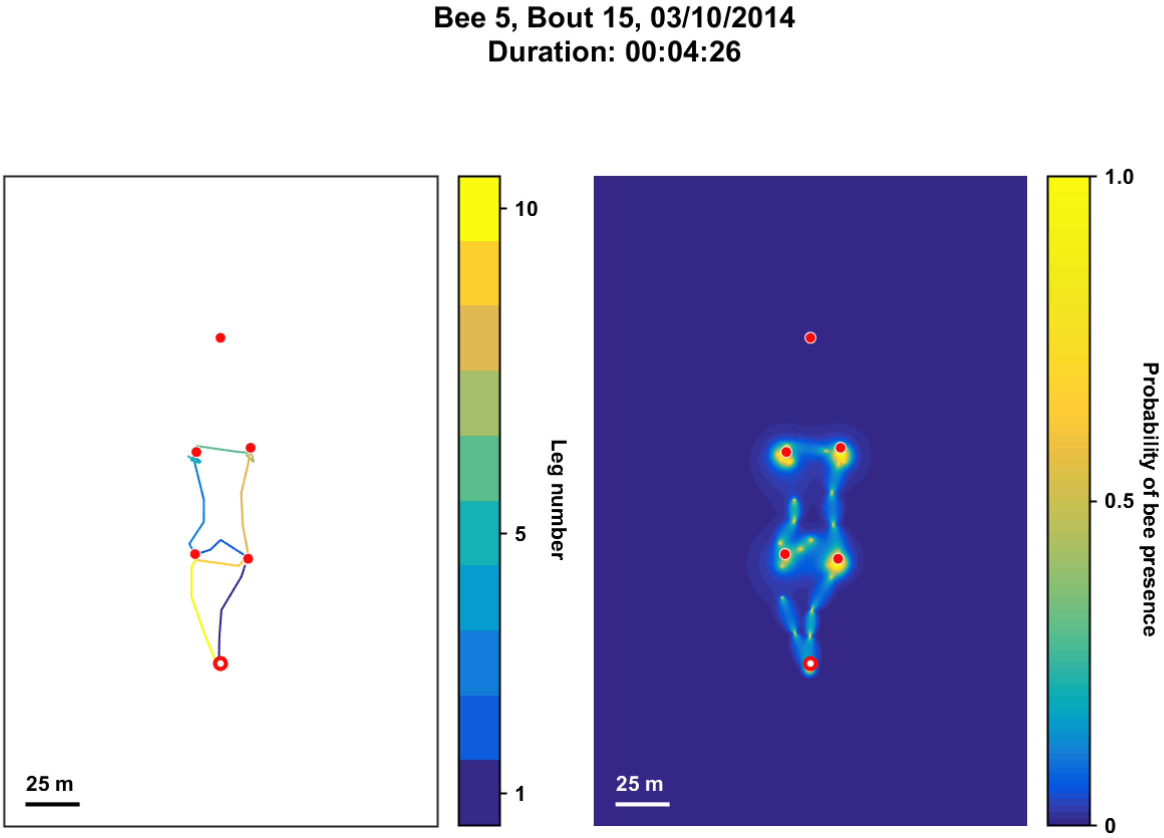
Bee 5, Bout 15, 03/10/2014
Duration: 00:04:26
25 m
10
5
1
Leg number
25 m
1.0
0.5
0
Probability of bee presence

Fig S182

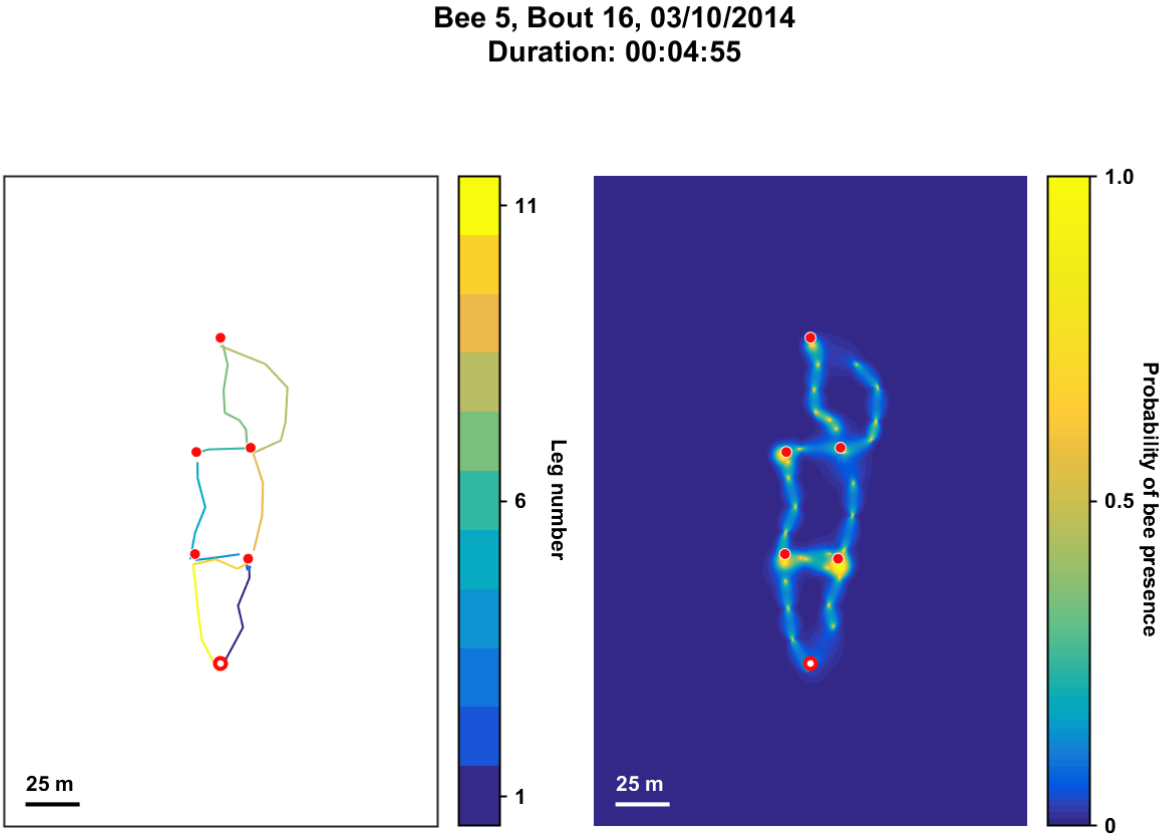

Bee 5, Bout 16, 03/10/2014
Duration: 00:04:55
25 m
11
6
1
Leg number
25 m
1.0
0.5
0
Probability of bee presence

Fig S183

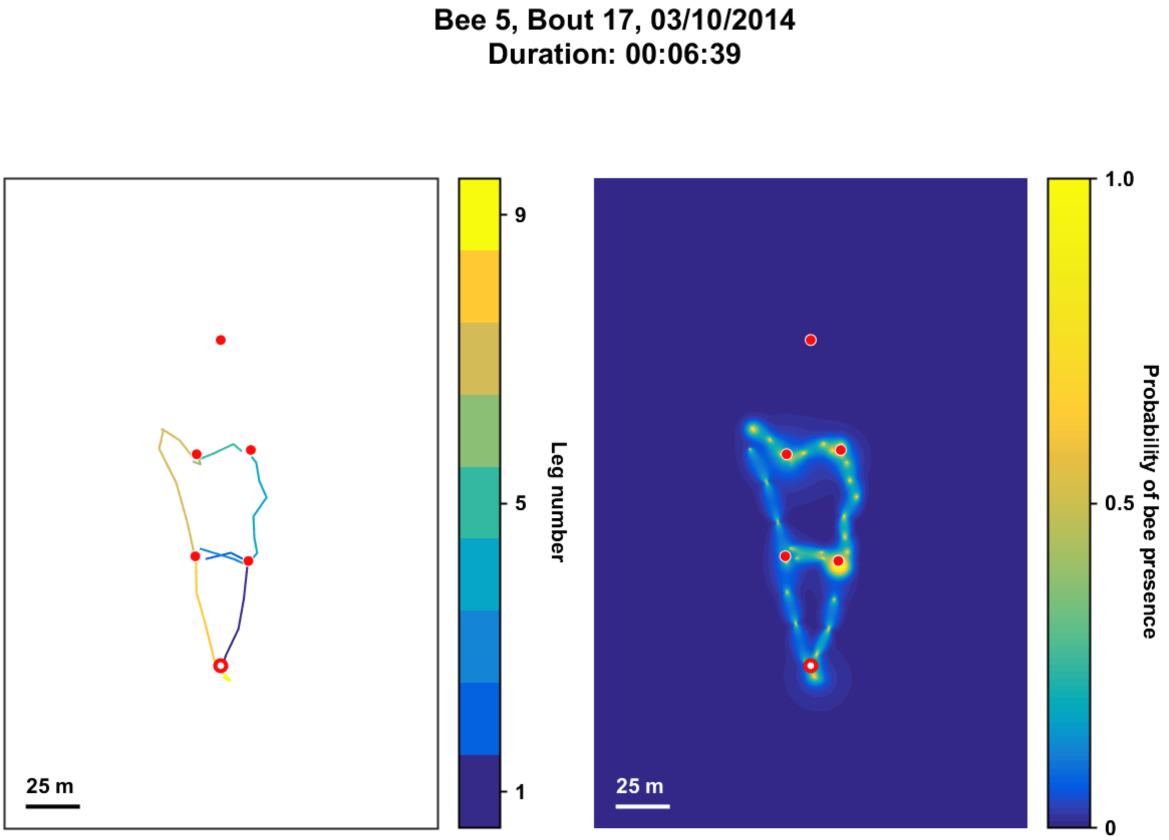

Bee 5, Bout 17, 03/10/2014
Duration: 00:06:39
25 m
9
5
1
Leg number
25 m
1.0
0.5
0
Probability of bee presence

Fig S184

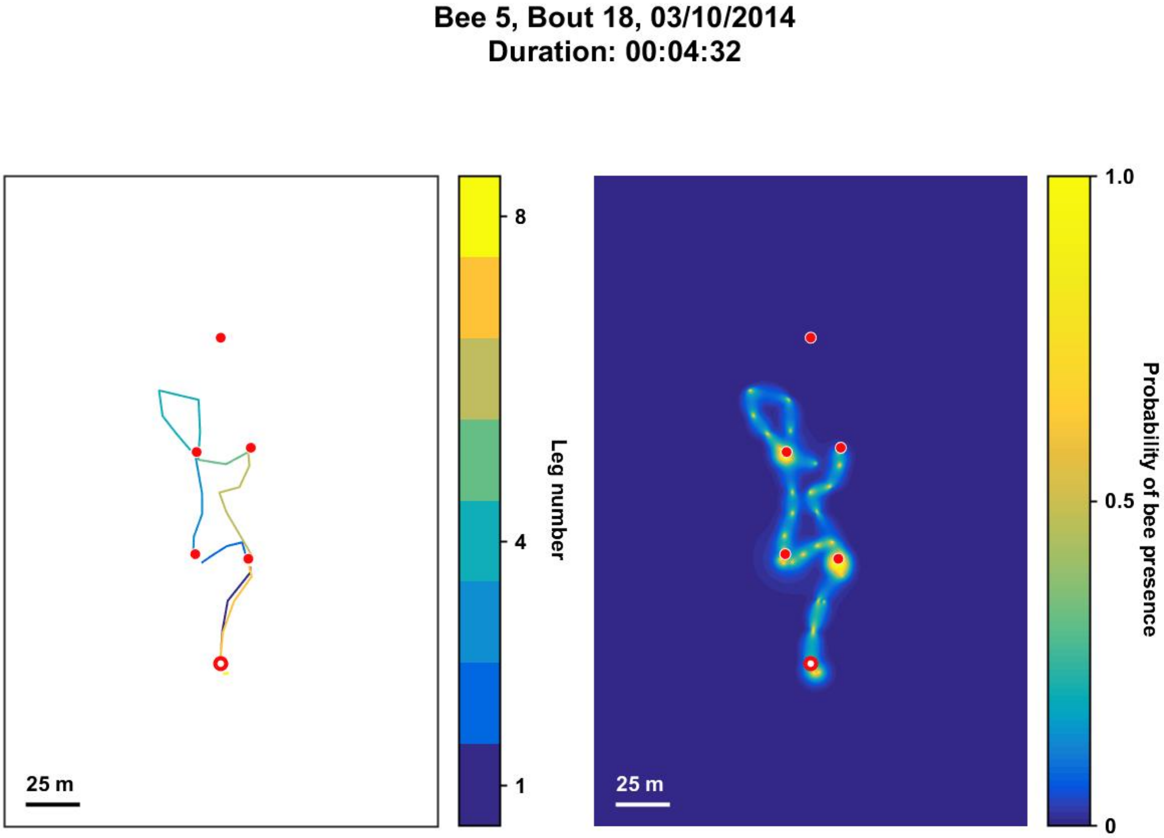

Bee 5, Bout 18, 03/10/2014
Duration: 00:04:32
25 m
Leg number
8
4
1
25 m
Probability of bee presence
1.0
0.5
0

Fig S185

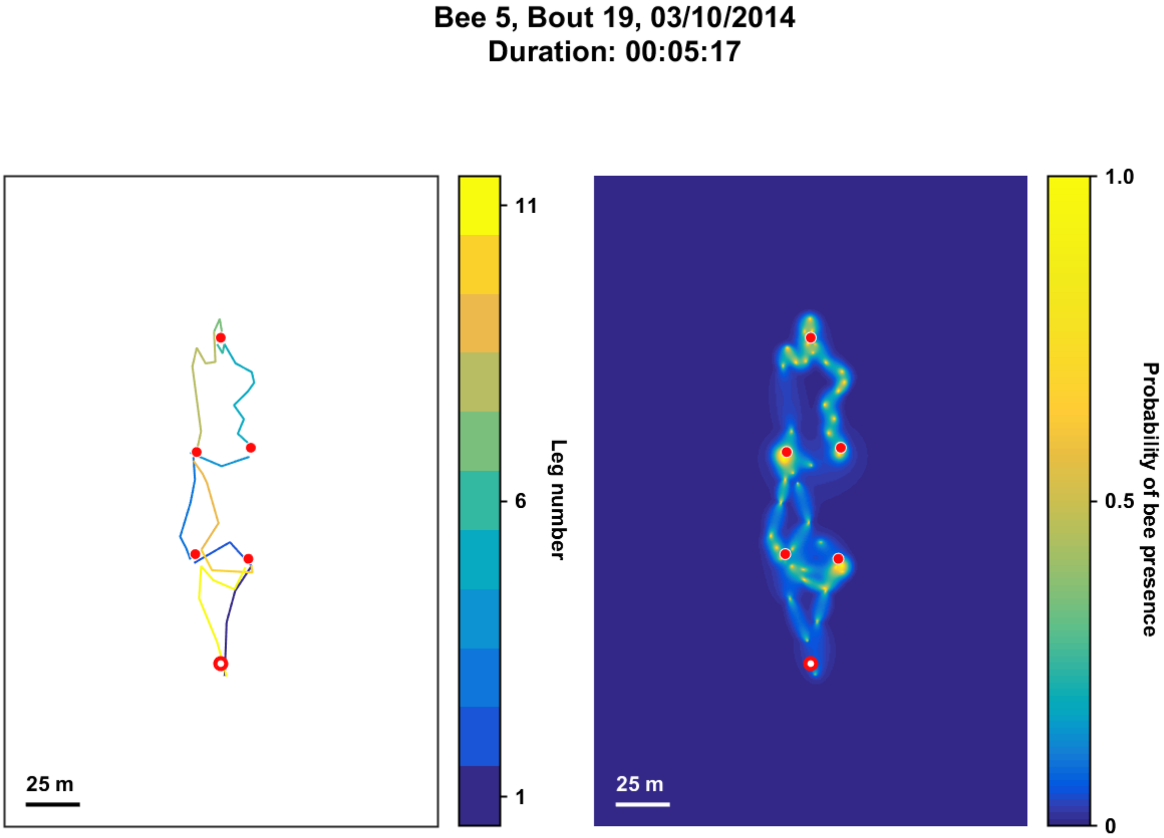
Bee 5, Bout 19, 03/10/2014
Duration: 00:05:17
25 m
11
6
1
Leg number
25 m
1.0
0.5
0
Probability of bee presence

Fig S186

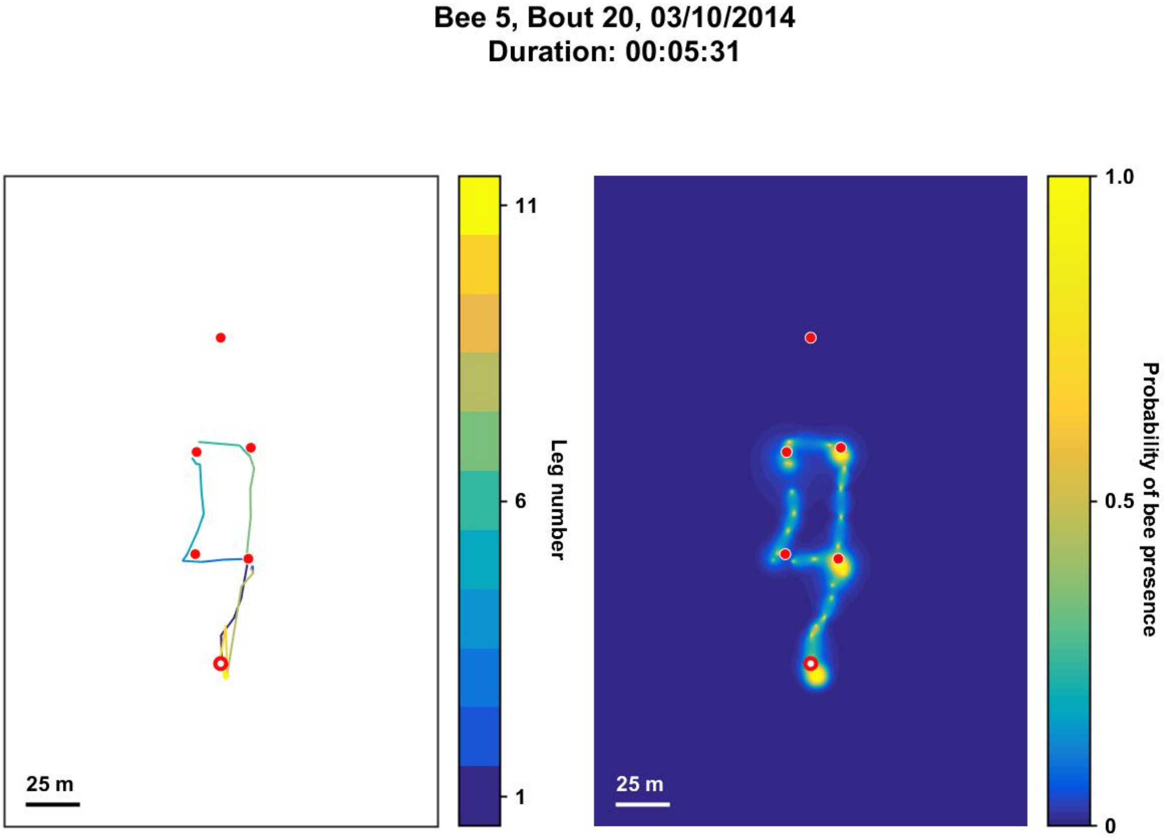

Bee 5, Bout 20, 03/10/2014
Duration: 00:05:31
25 m
Leg number
11
6
1
25 m
Probability of bee presence
1.0
0.5
0

Fig S187

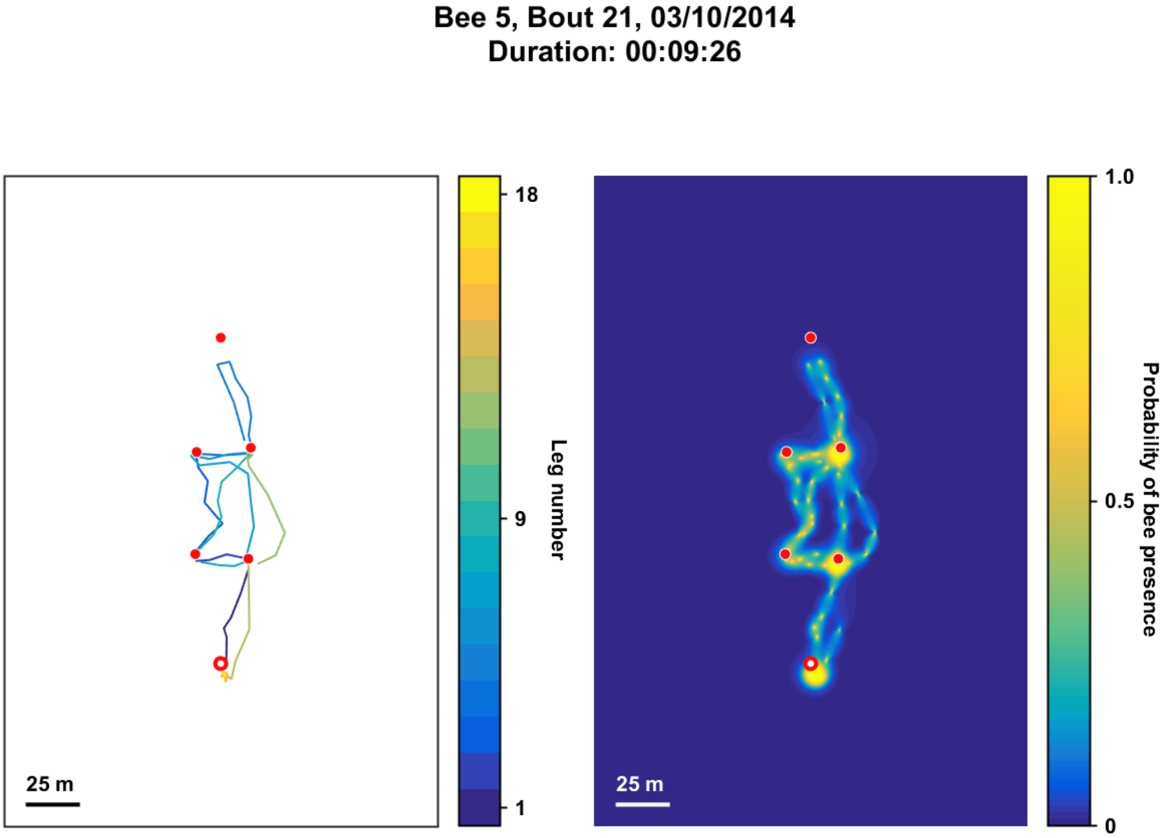
Bee 5, Bout 21, 03/10/2014
Duration: 00:09:26
18
9
1
Leg number
25 m
1.0
0.5
0
Probability of bee presence
25 m

Fig S188

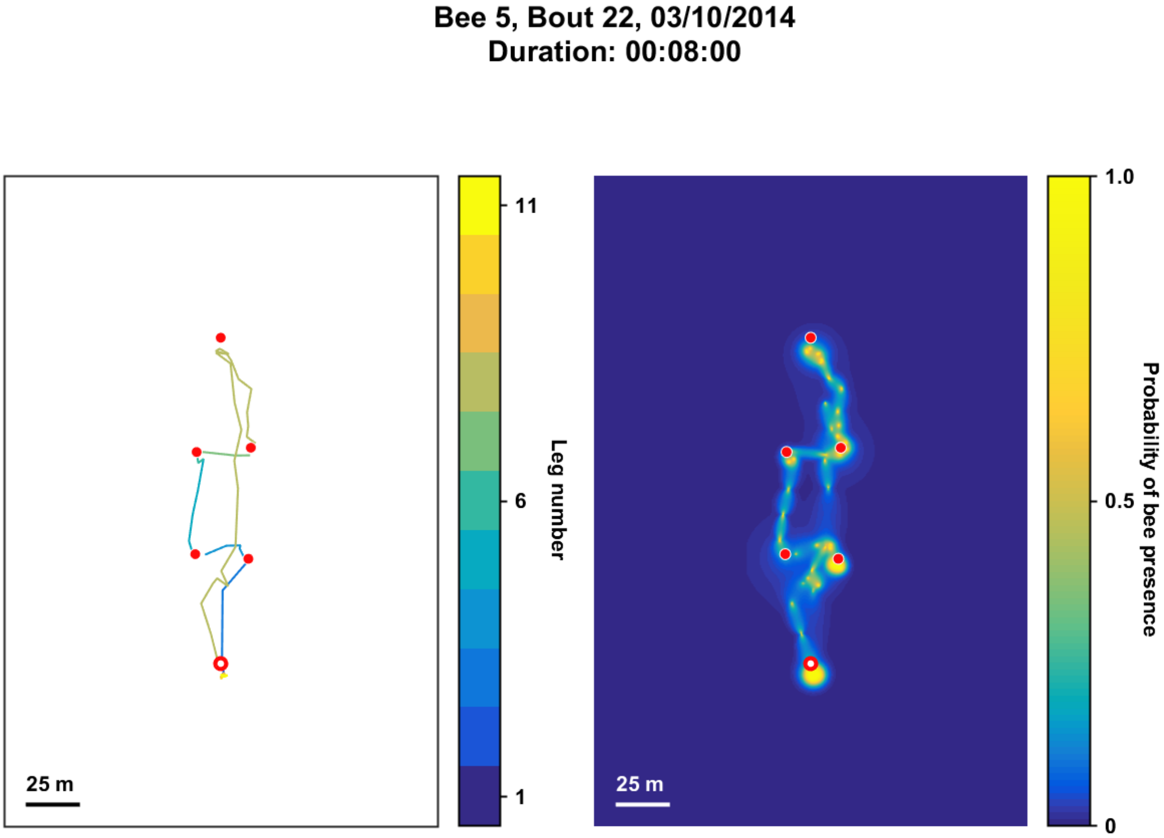

Bee 5, Bout 22, 03/10/2014
Duration: 00:08:00
25 m
11
6
1
Leg number
25 m
1.0
0.5
0
Probability of bee presence

Fig S189

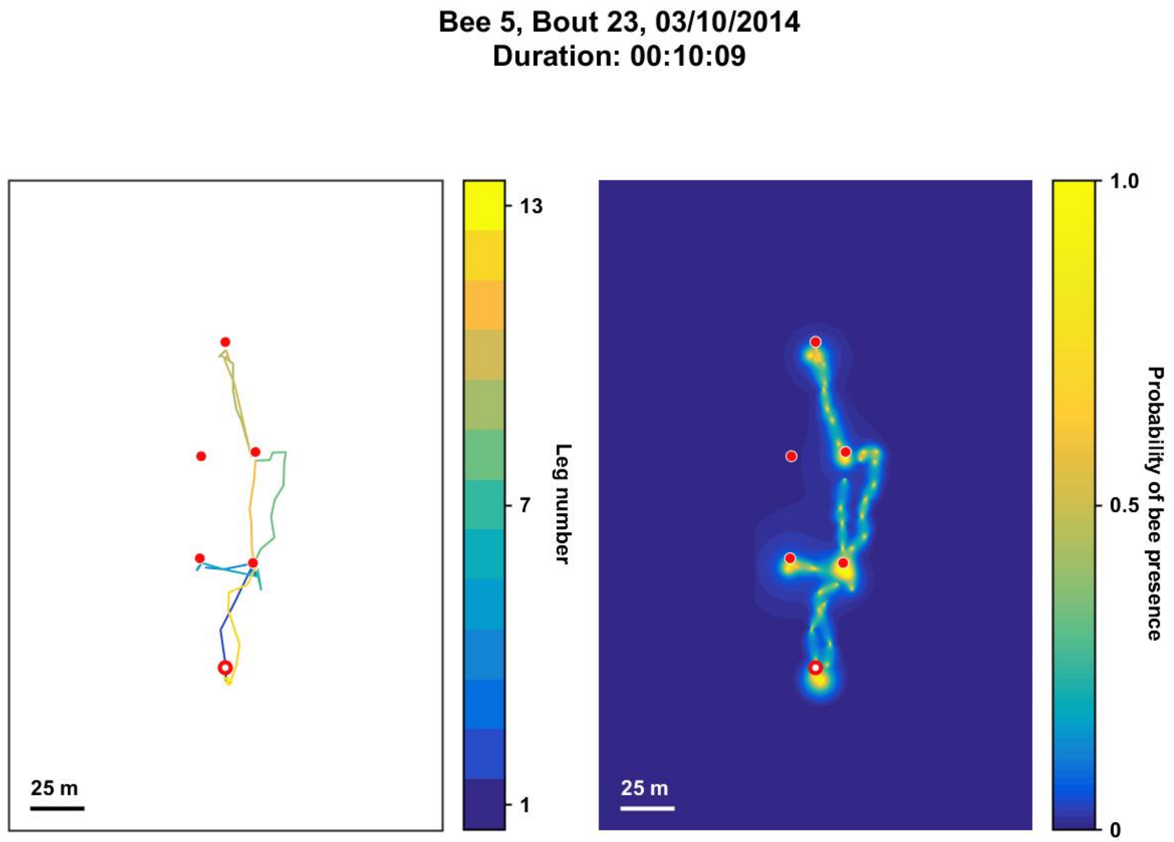

Bee 5, Bout 23, 03/10/2014
Duration: 00:10:09
25 m
Leg number
13
7
1
25 m
Probability of bee presence
1.0
0.5
0

Fig S190

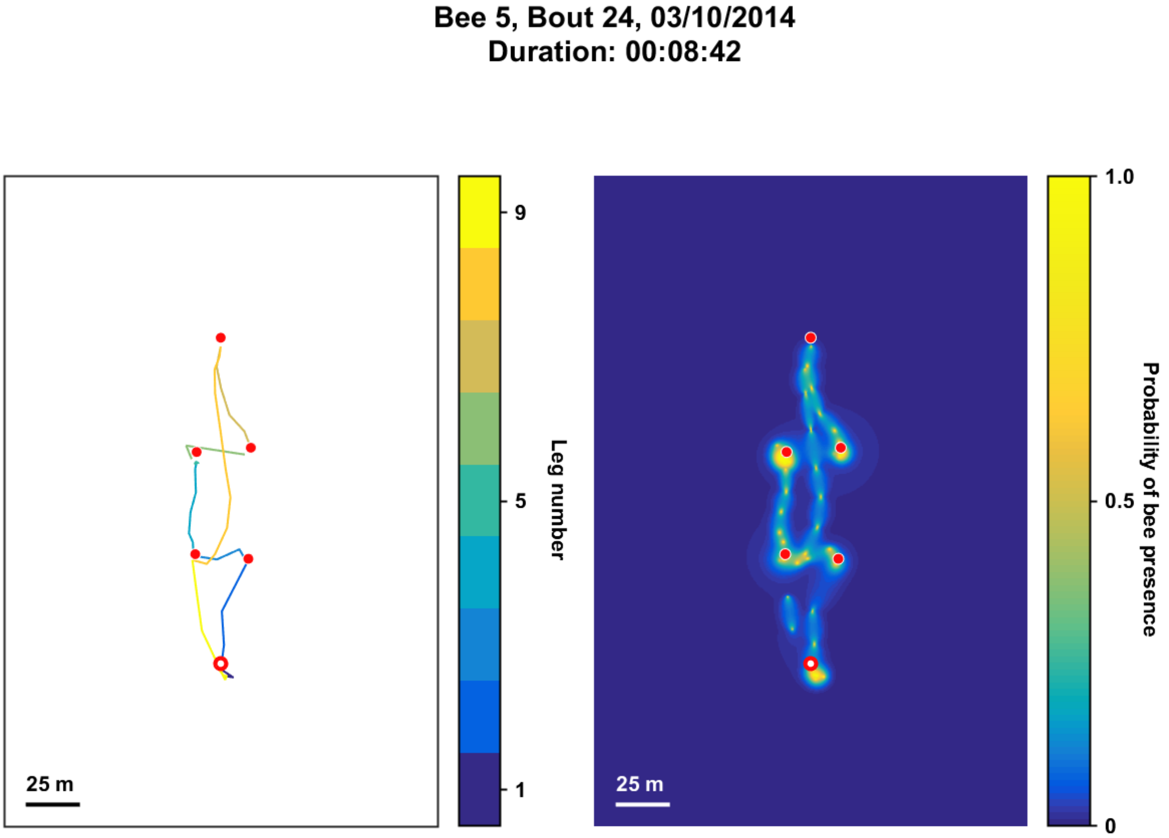
Bee 5, Bout 24, 03/10/2014
Duration: 00:08:42
25 m
9
5
1
Leg number
25 m
1.0
0.5
0
Probability of bee presence

Fig S191

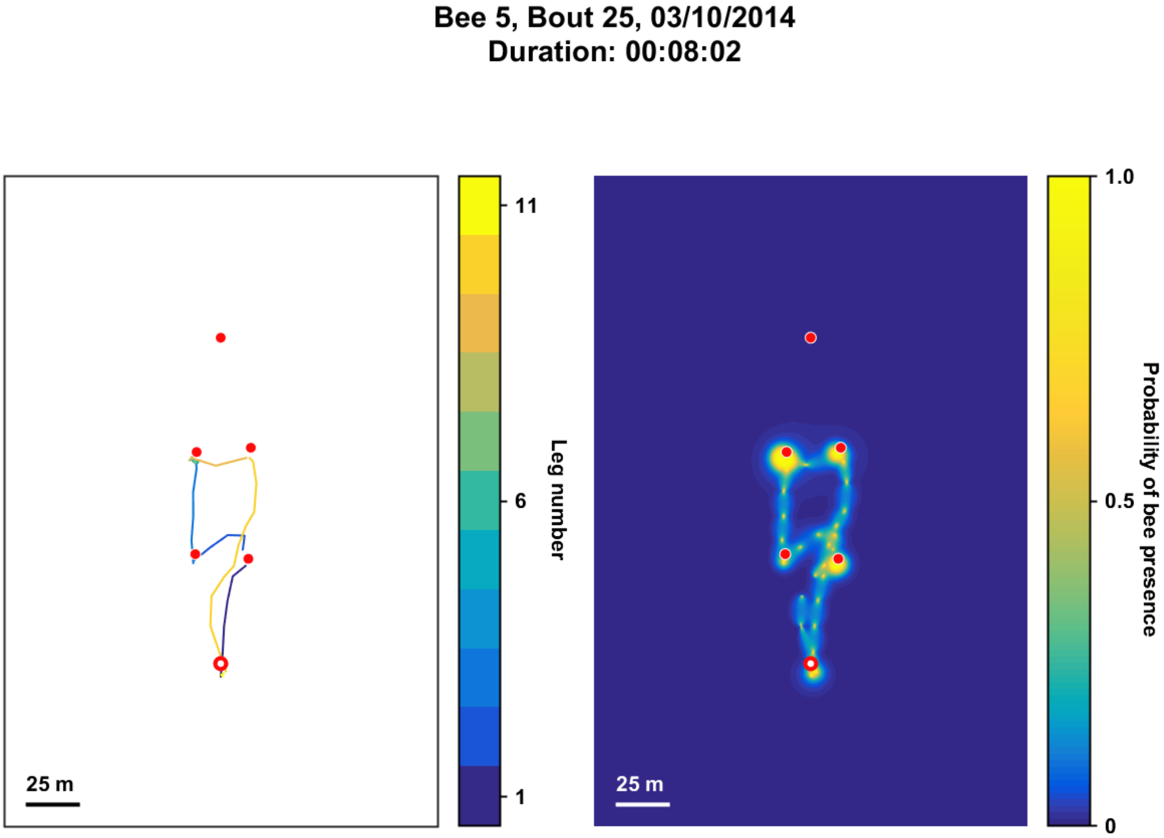

Bee 5, Bout 25, 03/10/2014
Duration: 00:08:02
25 m
11
6
1
Leg number
25 m
1.0
0.5
0
Probability of bee presence

Fig S192

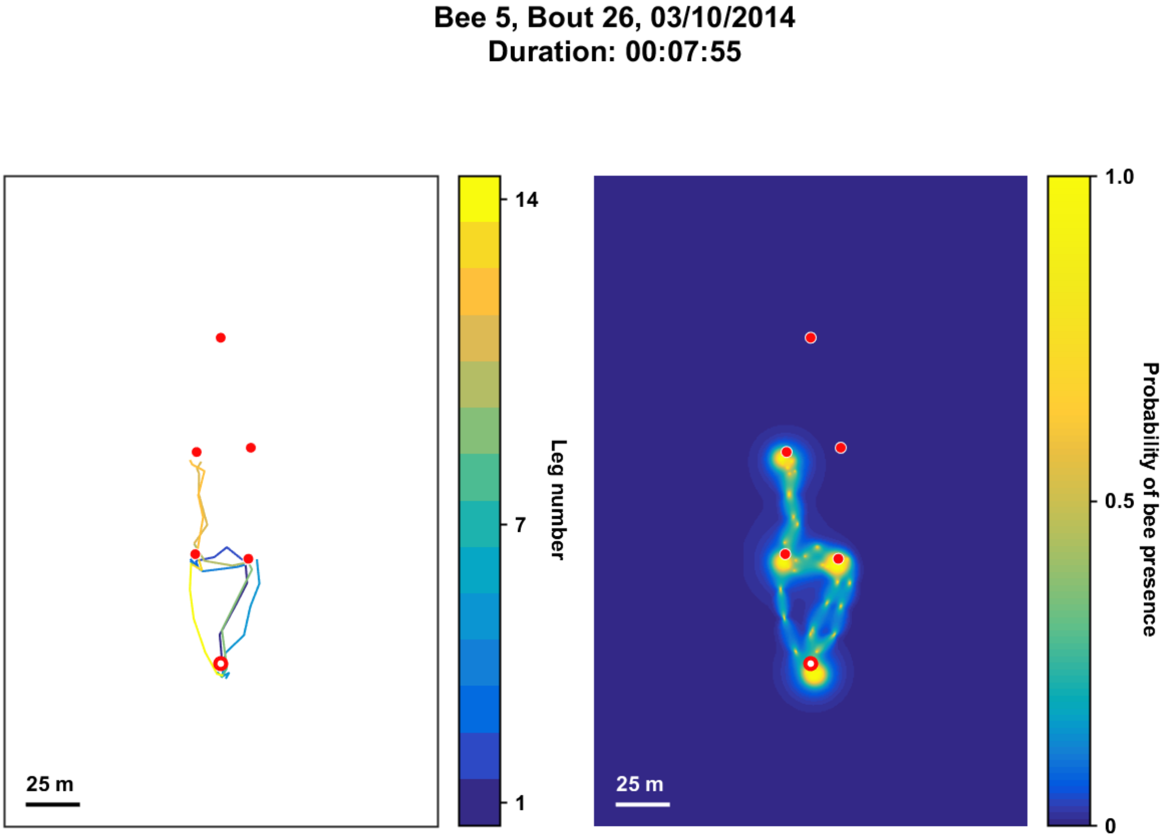

Bee 5, Bout 26, 03/10/2014
Duration: 00:07:55
25 m
14
7
1
Leg number
25 m
1.0
0.5
0
Probability of bee presence

Fig S193

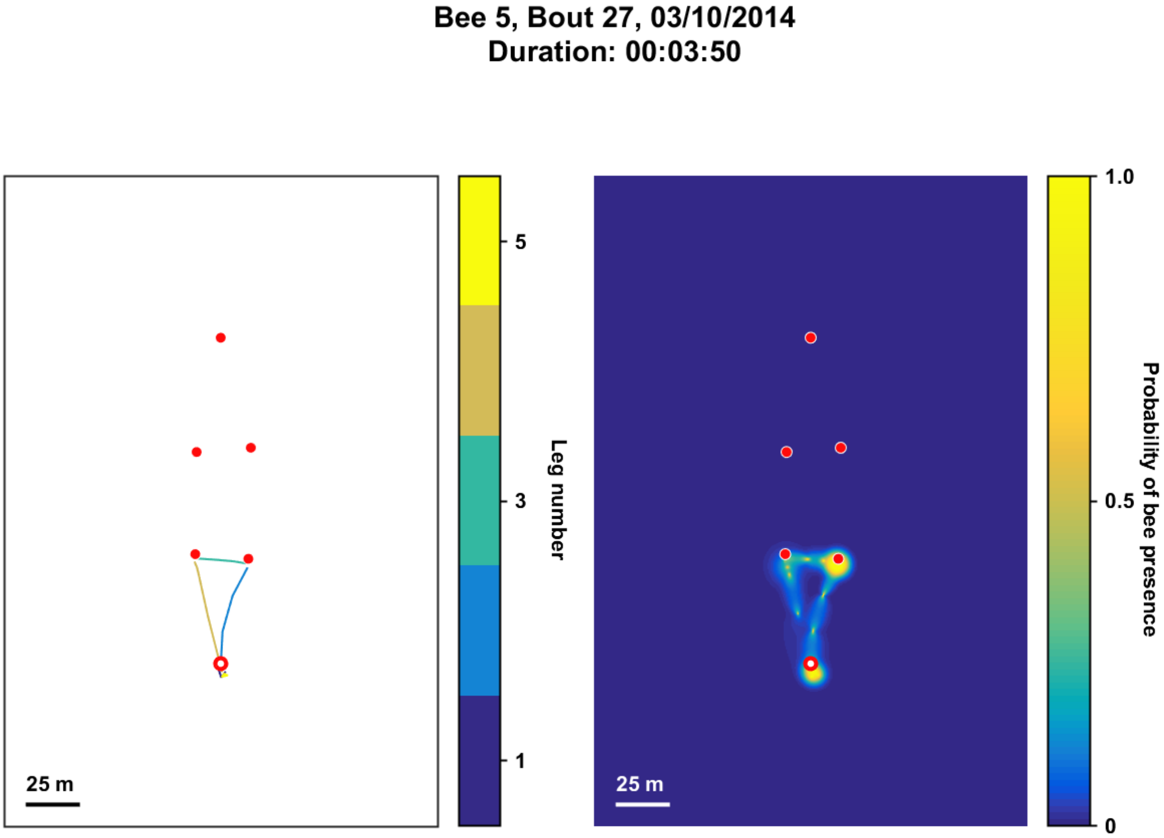
Bee 5, Bout 27, 03/10/2014
Duration: 00:03:50
25 m
Leg number
1
3
5
25 m
Probability of bee presence
0
0.5
1.0

Fig S194

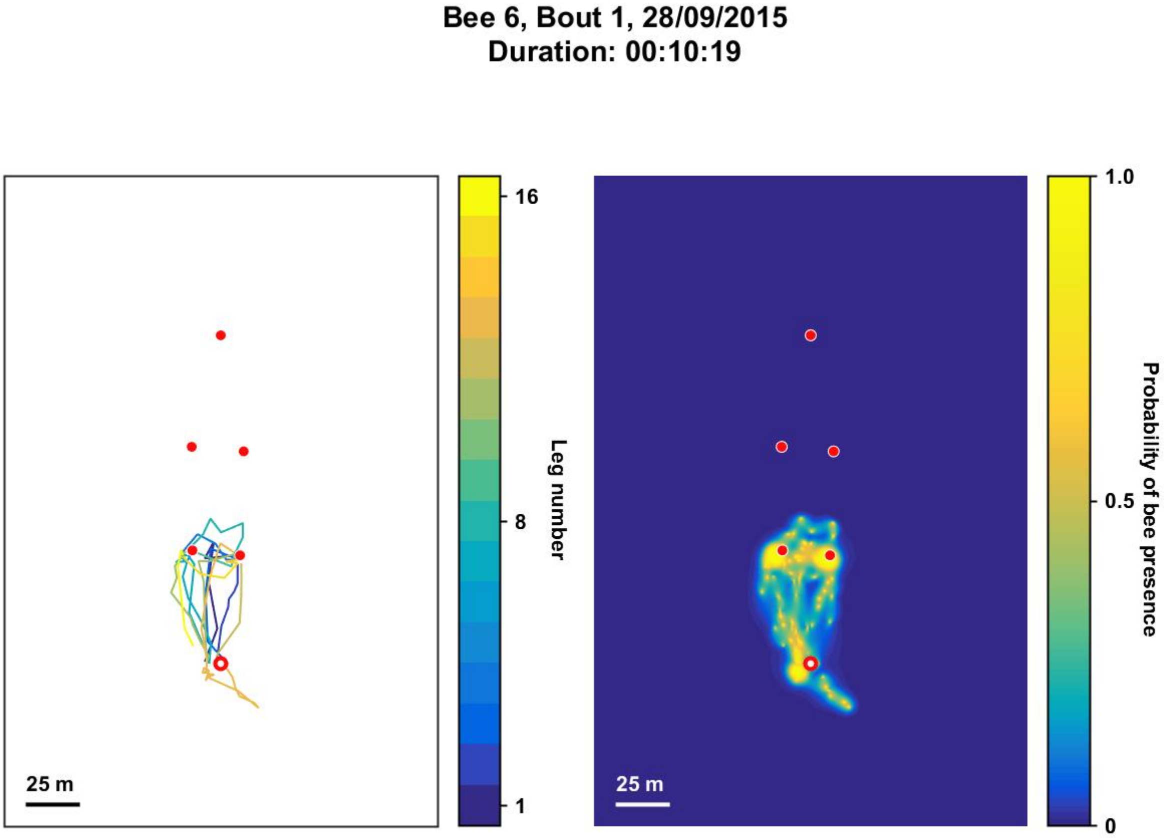

Bee 6, Bout 1, 28/09/2015
Duration: 00:10:19
25 m
16
8
1
Leg number
25 m
1.0
0.5
0
Probability of bee presence

Fig S195

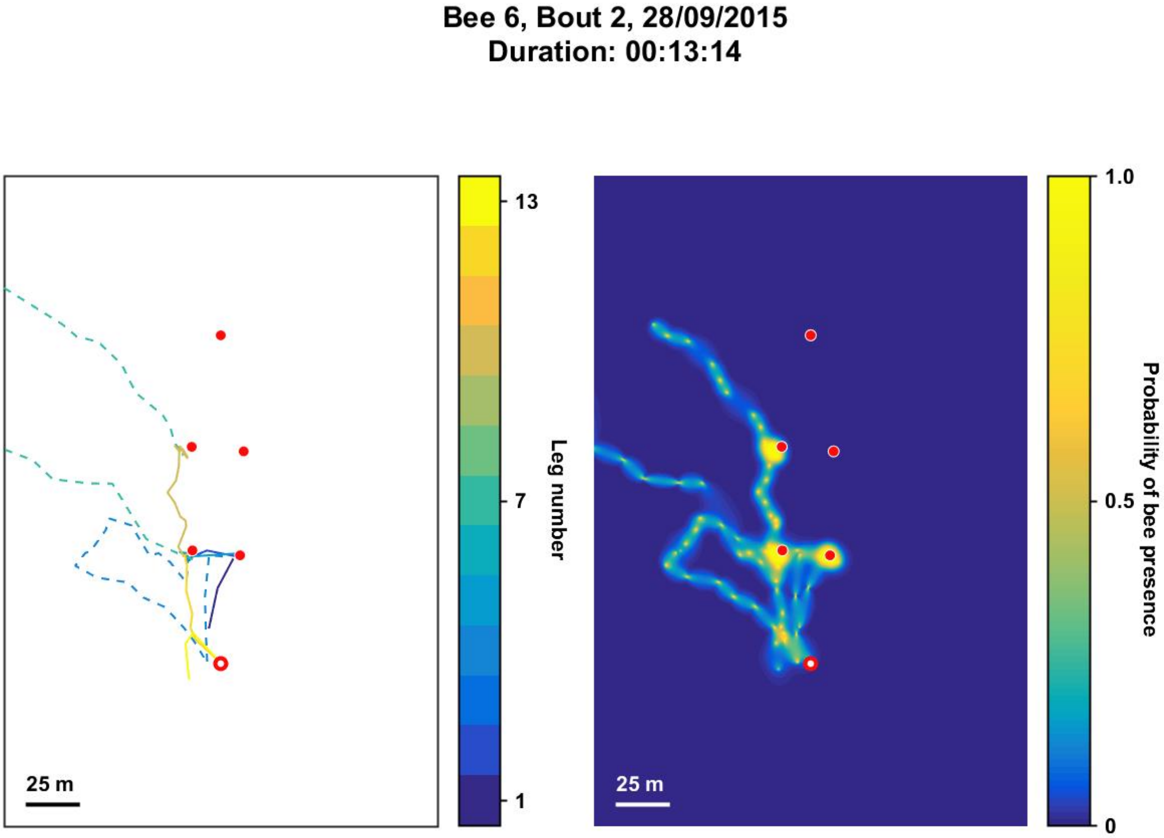

Bee 6, Bout 2, 28/09/2015
Duration: 00:13:14
25 m
13
7
1
Leg number
25 m
1.0
0.5
0
Probability of bee presence

Fig S196

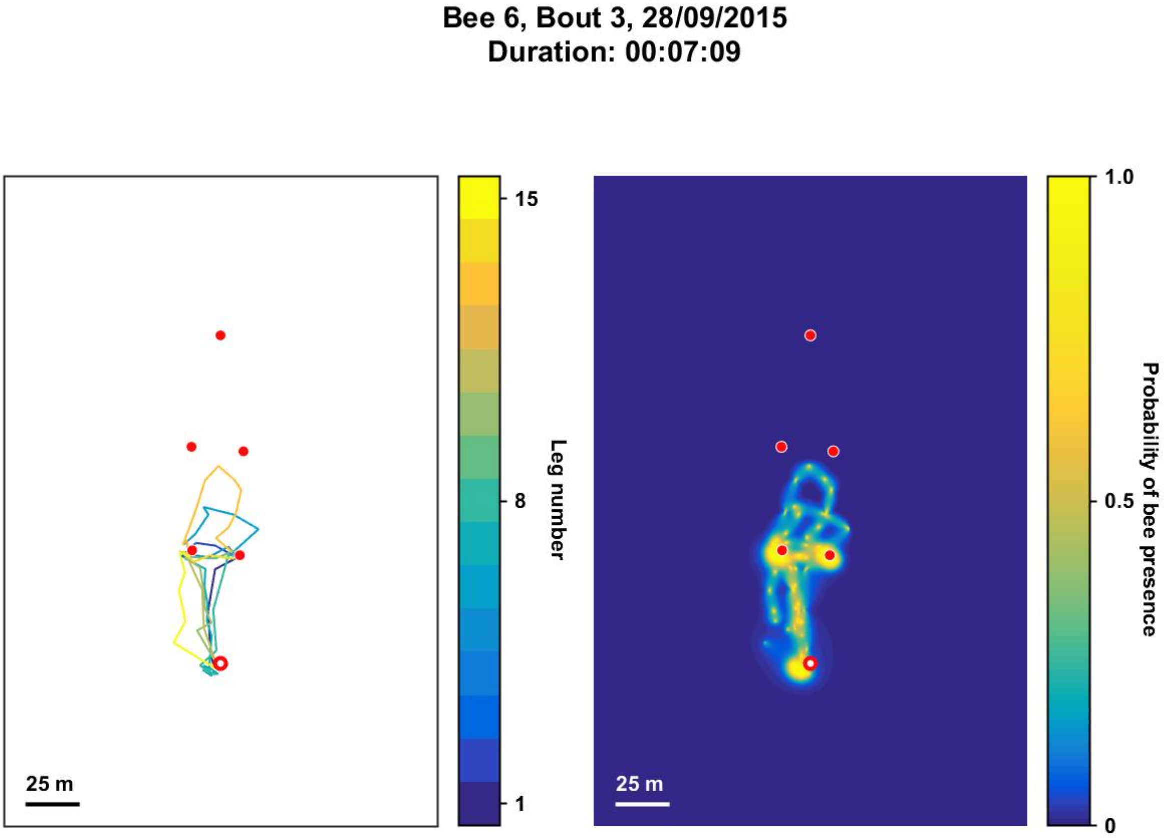

Bee 6, Bout 3, 28/09/2015
Duration: 00:07:09
25 m
Leg number
15
8
1
25 m
Probability of bee presence
1.0
0.5
0

Fig S197

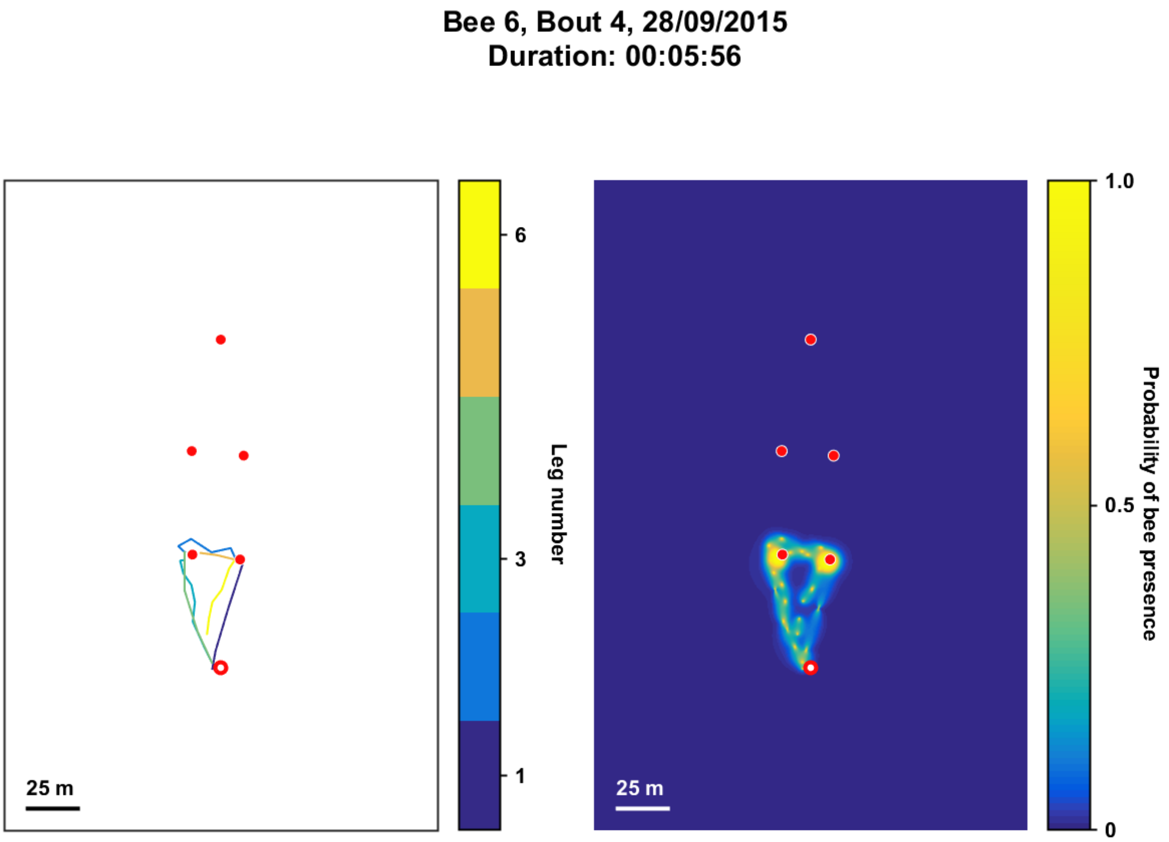
Bee 6, Bout 4, 28/09/2015
Duration: 00:05:56
25 m
6
3
1
Leg number
25 m
1.0
0.5
0
Probability of bee presence

Fig S198

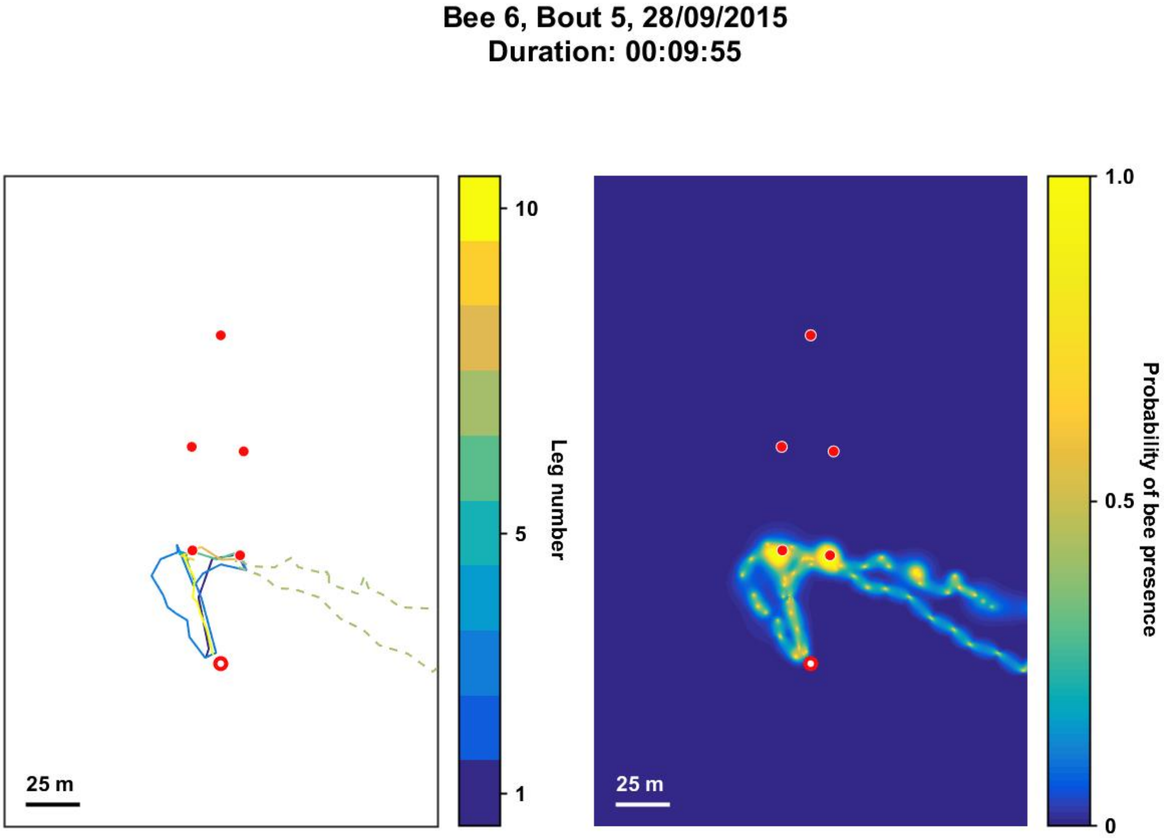

Bee 6, Bout 5, 28/09/2015
Duration: 00:09:55
25 m
Leg number
10
5
1
25 m
Probability of bee presence
1.0
0.5
0

Fig S199

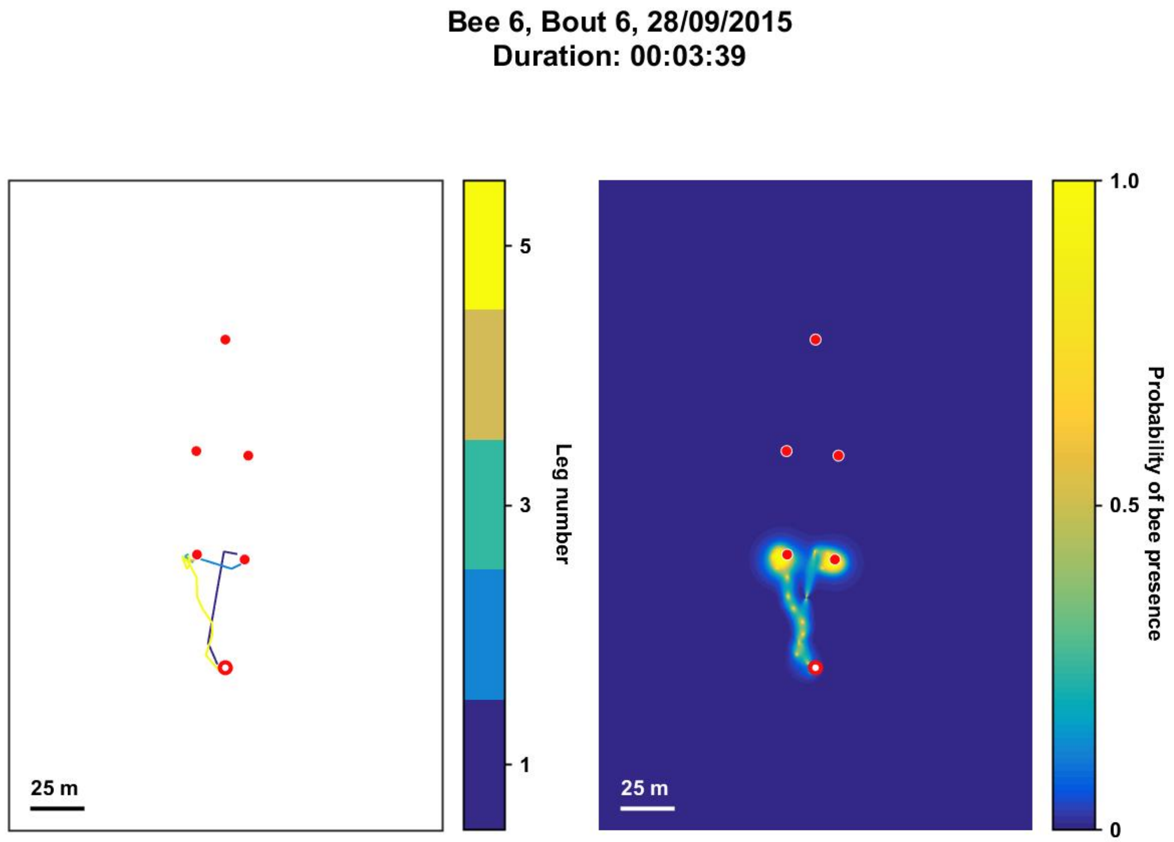
Bee 6, Bout 6, 28/09/2015
Duration: 00:03:39
25 m
Leg number
1
3
5
25 m
Probability of bee presence
0
0.5
1.0

Fig S200

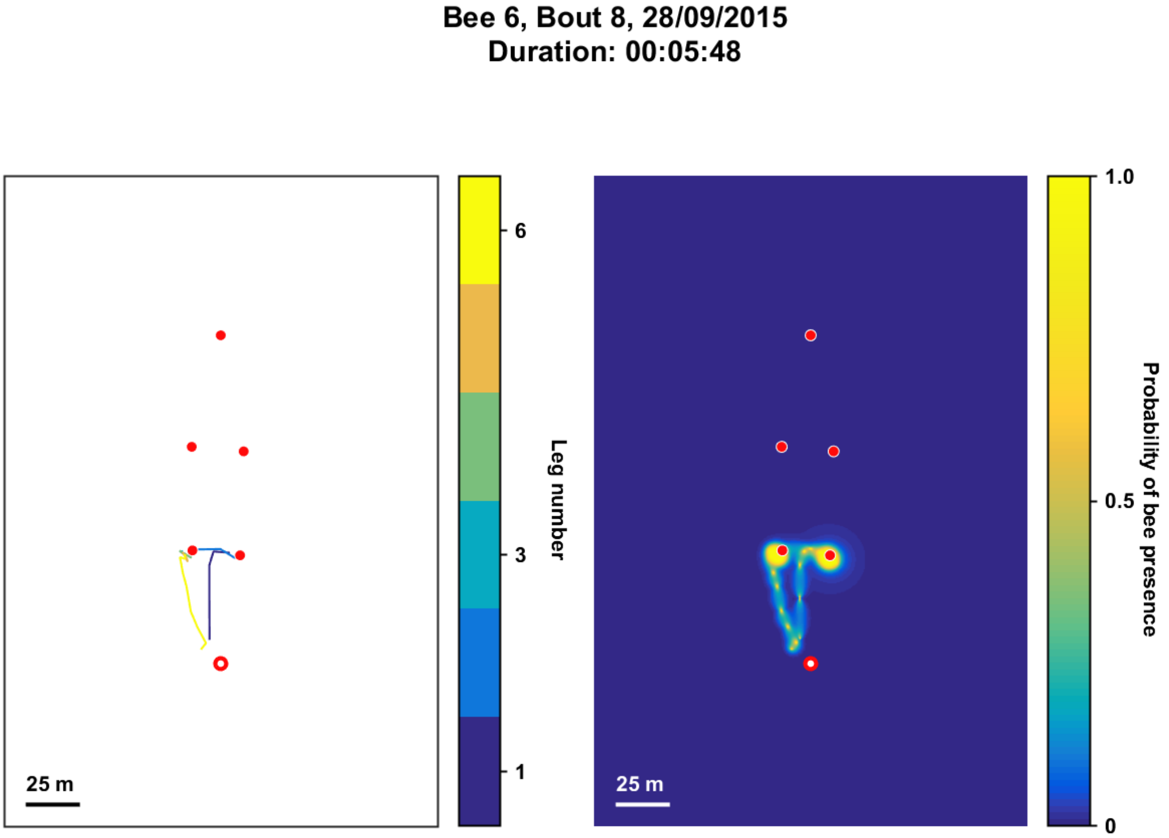

Bee 6, Bout 8, 28/09/2015
Duration: 00:05:48
25 m
6
3
1
Leg number
25 m
1.0
0.5
0
Probability of bee presence

Fig S201

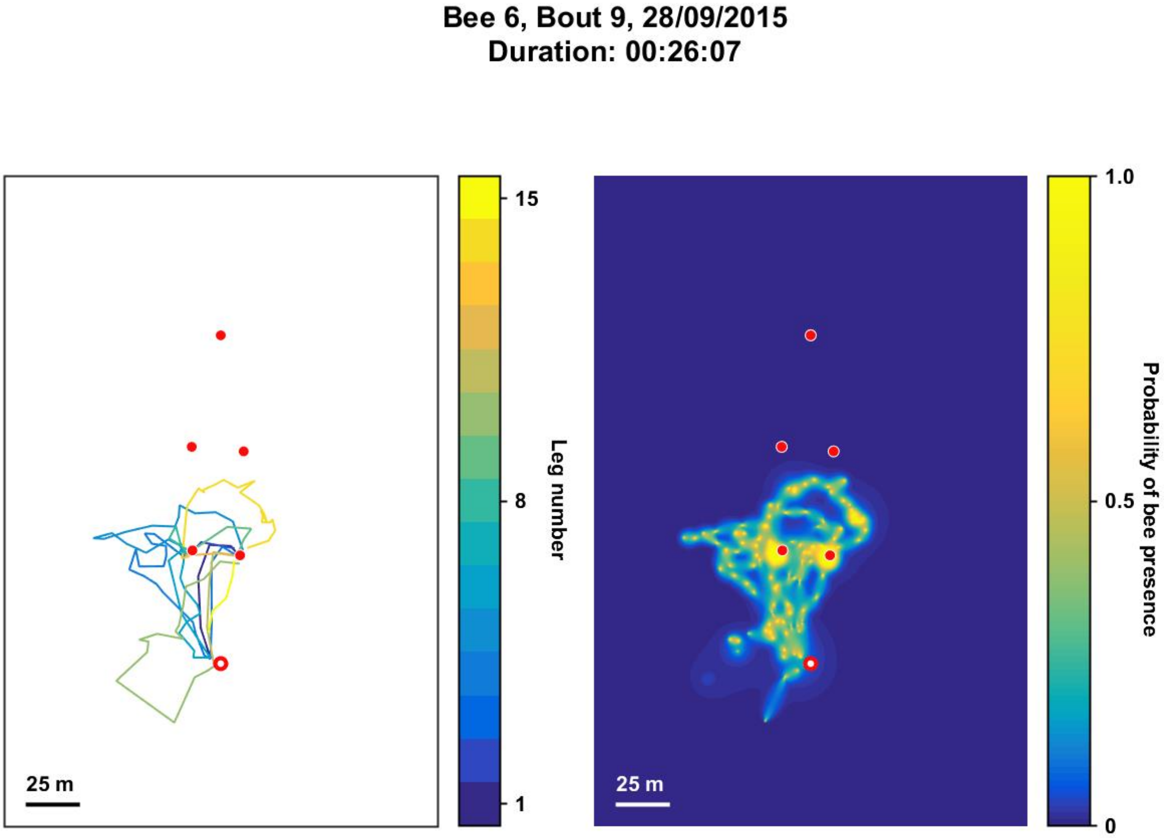

Bee 6, Bout 9, 28/09/2015
Duration: 00:26:07
25 m
Leg number
15
8
1
25 m
Probability of bee presence
1.0
0.5
0

Supplementary Fig S202: Heat maps showing the early foraging bouts of two bees. Bees 4 and 6 completed only 6 and 9 bouts respectively, too few to assess how their routes changed over time as in Fig 2. Their initial 5 bouts on the array are shown here for comparison with Fig 2 a-d. Nest position: open circle; positions of feeders: closed circles. The colour of each pixel represents the probability that the bee passed over that point in the landscape as estimated from the radar tracks using a Brownian bridges technique (described in Methods). Each pixel represents an area 5 x 5m. Scale bars represent a distance of 25m. Each panel represents the cumulative probability map of one bee's location over its first 5 bouts of foraging on the feeder array: a) Bee 4. b) Bee 6.

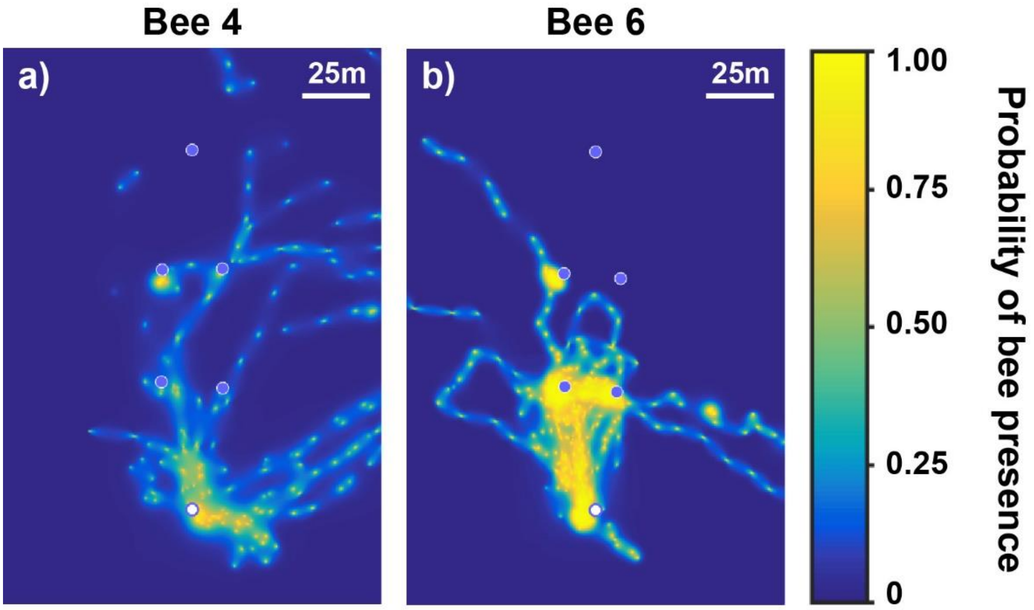

Supplementary Fig S203: Conditions under which exploration legs take place. Individual legs of each foraging bout were categorised as 'explorations' if the bee travelled further than 50m from the nest and all feeders it had previously visited (i.e. the bee was further from all known feeders than the feeders were from one another). All panels show the combined data from all bees 1-6. a) Histogram showing how many exploration legs started at each feeder (1-5 indicate feeders #1-#5, 6 indicates the nest). Explorations started from all feeders but most commonly originated at the nest. b) Histogram showing how many feeders had been discovered and fed from at the point each exploration leg began. Explorations rarely occurred before the bee had fed at all and were most common when the bee had visited four out of five feeders. c) Histogram showing how many times a bee had revisited empty feeders before beginning an exploration leg. Explorations commonly occurred without any revisits to empty feeders. d) Comparison of the straightness of the flight paths during legs in which the bee remained within the array to exploration legs. Exploration legs are significantly less straight than those that remained within the array.

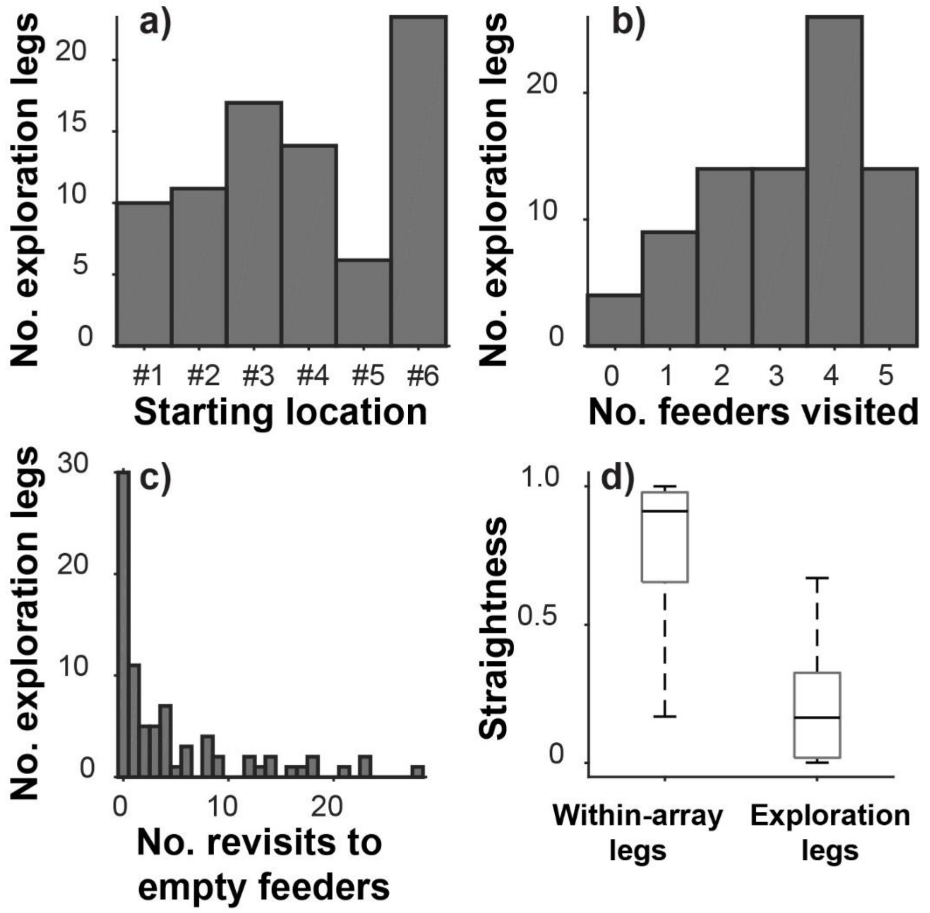

Supplementary Fig S204: Path length and duration of foraging bouts of two bees. Bees 4 and 6 completed only 6 and 9 bouts respectively, too few to assess how path lengths and duration of their flights changed over time as in Fig 3; data are shown here for comparison. X-axis of each plot shows the cumulative number of foraging bouts by each bee. a-b) Path length of each bout by bees 4 and 6 respectively. Distances were estimated as the cumulative length of a series of straight lines joining every pair of consecutive radar observations of the bees' positions. Total distance flown is broken down into two categories: blue bars = distance flown in legs that remained within 50m of a feeder; yellow bars = distance flown during 'exploration' legs (those in which the bee travels further than 50m from all feeders it has previously visited). Horizontal dashed grey line indicates minimum possible length of an optimal route visiting all feeders. Red dots indicate straight-line path length, the theoretical distance of a route composed of straight lines that visits the feeders in the order observed in each bout. c-d) Duration of each bout flown by bees 4 and 6 respectively. Total duration is broken down into three categories: blue bars = time spent within 5m of a feeder; green bars = total duration of legs that remained within 50m of a feeder; yellow bars = total duration of 'exploration' legs.

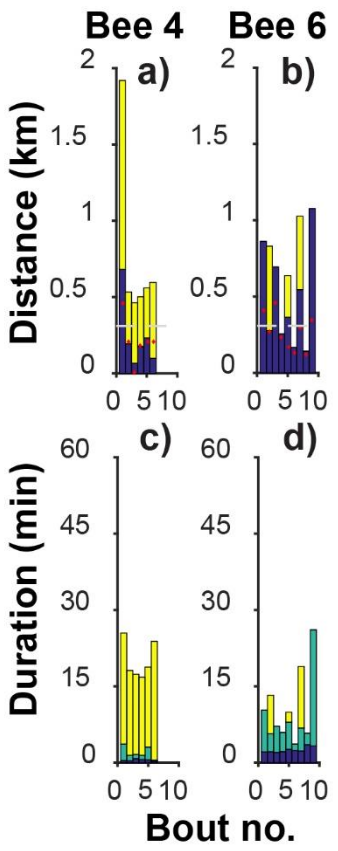

Supplementary Fig S205: Proportion of total flight distance attributable to visit sequence. The red diamonds represent straight-line path length, the theoretical minimal distance required to visit the feeders in the order observed during each bout. This distance is expressed as a proportion of the total distance actually travelled by the bee in each bout. The background to each plot shows the proportion of the total distance flown by the bee which can be attributed to legs within the array (darker grey bars) and to 'exploration' legs (those in which the bee travels further than 50 m from all feeders it has previously visited; pale grey bars). Each panel represents every foraging bout flown by one bee. The x-axis of each plot shows the cumulative number of foraging bouts experienced by each bee. a-f) Bees 1-6 respectively. As bees gained experience on the feeder array an increasing proportion of the distance they flew could be attributed to the chosen visit sequence rather than inefficiency in the flight paths.

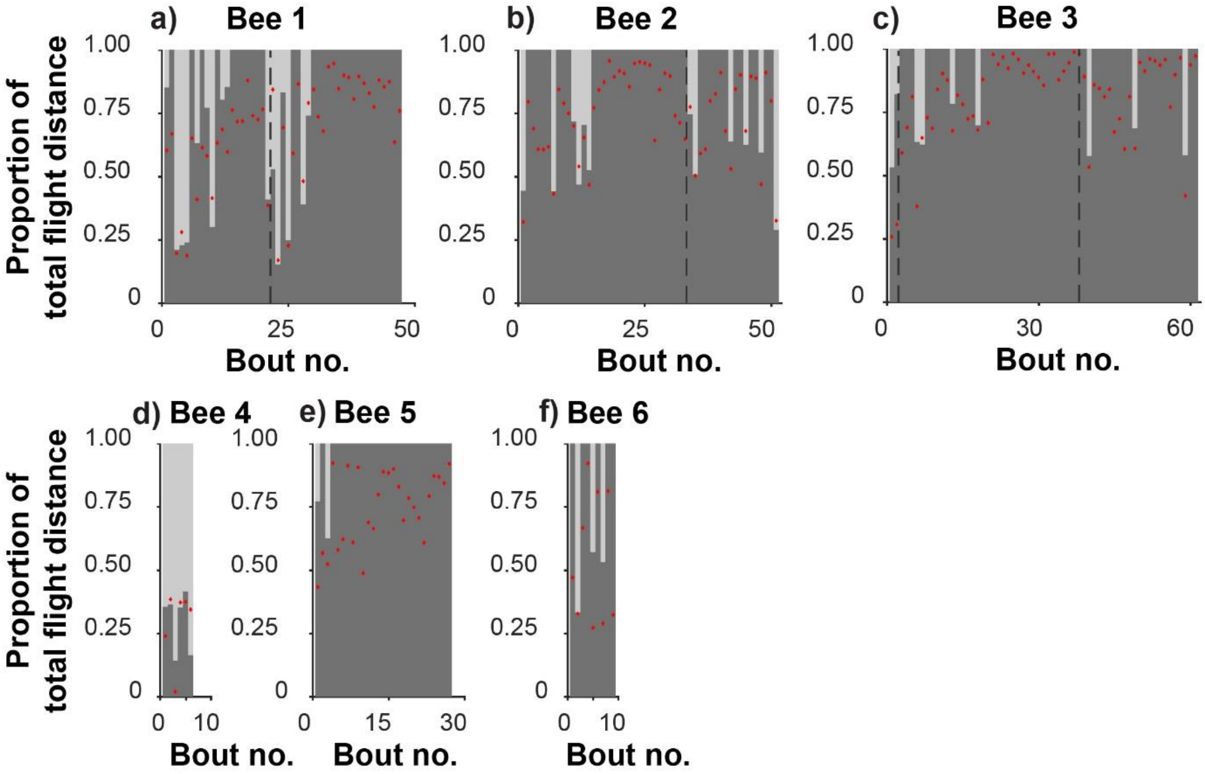
a) Bee 1
b) Bee 2
c) Bee 3
d) Bee 4
e) Bee 5
f) Bee 6
Proportion of total flight distance
Bout no.
1.00
0.75
0.50
0.25
0
0
25
50
30
60
10
15

Supplementary Fig S206: Optimality of visit sequence and straightness of flights for two bees. Bees 4 and 6 completed only 6 and 9 bouts respectively, too few to assess how the efficiency of their flights changed over time as in Fig 4; data are shown here for comparison. Each panel represents every foraging bout flown by one bee. The x-axis of each plot shows the cumulative number of foraging bouts experienced by each bee. a-b) Optimality score for the feeder visit sequence in each bout by bees 4 and 6 respectively. c-d) Mean straightness of the flight paths of all legs in each bout by bees 4 and respectively.

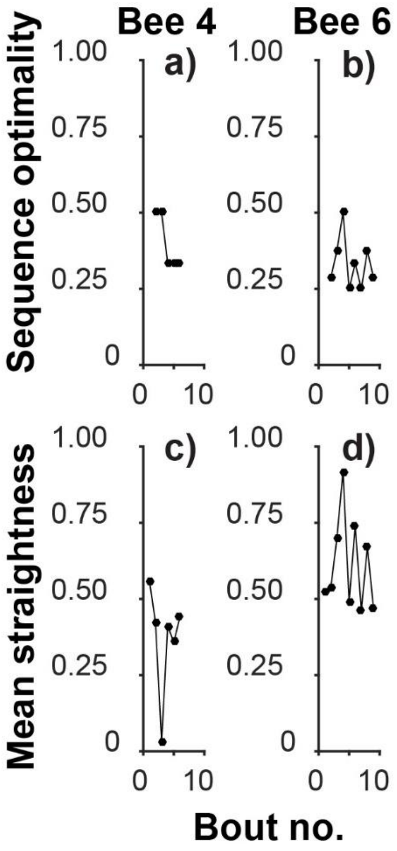

Supplementary Fig S207: Mean distance ranks show legs largely involve transitions to nearby feeders. Each panel represents every foraging bout flown by one bee. The x-axis of each plot shows the cumulative number of foraging bouts experienced by each bee. The vertical dashed lines indicate overnight breaks between foraging bouts. a-f) For bees 1-6 respectively, the mean distance rank of all legs in each bout. Each leg is assigned a rank reflecting how far the destination location is from the starting location. There was little change in these ranks with experience.

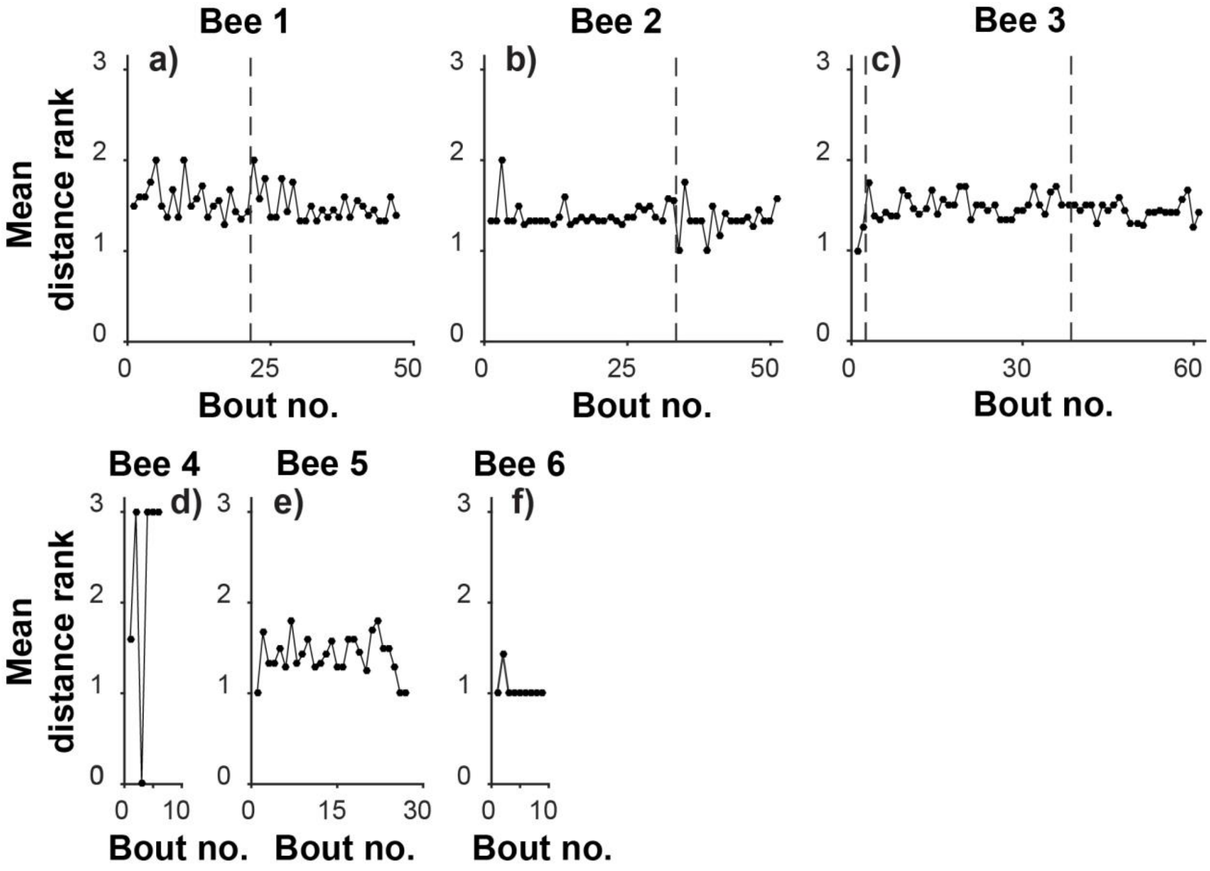

Supplementary Fig S208: Repeatability of visit sequence and flight path for two bees. Bees 4 and 6 completed only 6 and 9 bouts respectively, too few to assess how the stability of their flights changed over time as in Fig 5; data are shown here for comparison. Each panel represents every foraging bout flown by one bee. The x-axis of each plot shows the cumulative number of foraging bouts experienced by each bee. a-b) Self-similarity score for the feeder visit sequence in each bout by bees 4 and 6 respectively (similarity of the sequence of feeder visits in each bout to that of the previous bout undertaken by the same bee). c-d) Self-similarity score comparing the probability map of the bee's location during each bout to that of the previous bout by bees 4 and respectively. e-f) For bees 4 and 6 respectively, the mean similarity of the individual legs of each bout to the most recent occasion on which the same bee made the same feeder-to-feeder transition.

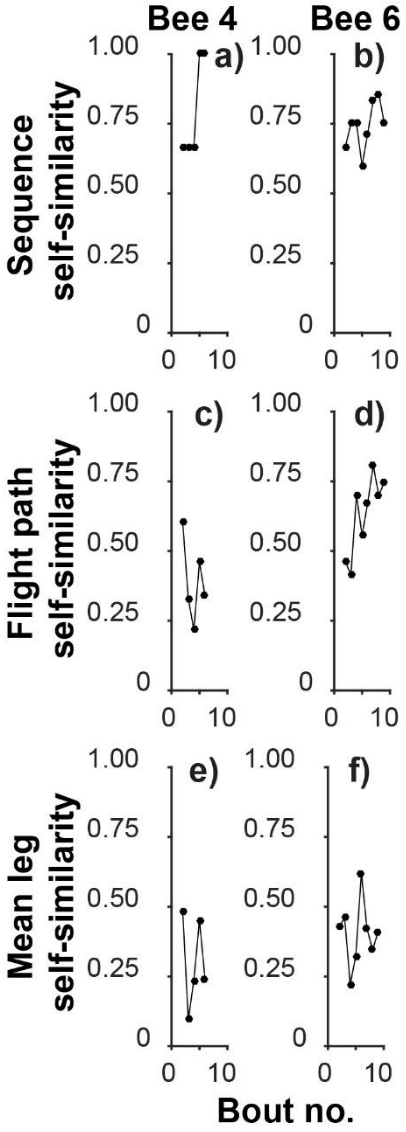

Supplementary Fig S209: Flight paths become more repeatable with experience at multiple timescales. Each panel represents every foraging bout flown by one bee. The x-axis of each plot shows the cumulative number of foraging bouts experienced by each bee. The vertical dashed lines indicate overnight breaks between foraging bouts. a-d) Self-similarity score for bees 1-3 and 5 respectively, comparing the probability map of the bees' location during each bout to that recorded two bouts previously. e-h) Self-similarity score comparing each bout's flight path to that recorded three bouts previously. i-l) Self-similarity score comparing each bout's flight path to that recorded four bouts previously. Similar figures comparing each bout to that recorded one bout previously are shown in Fig 5e-h. At all timescales there is a tendency toward increasing similarity scores over time.

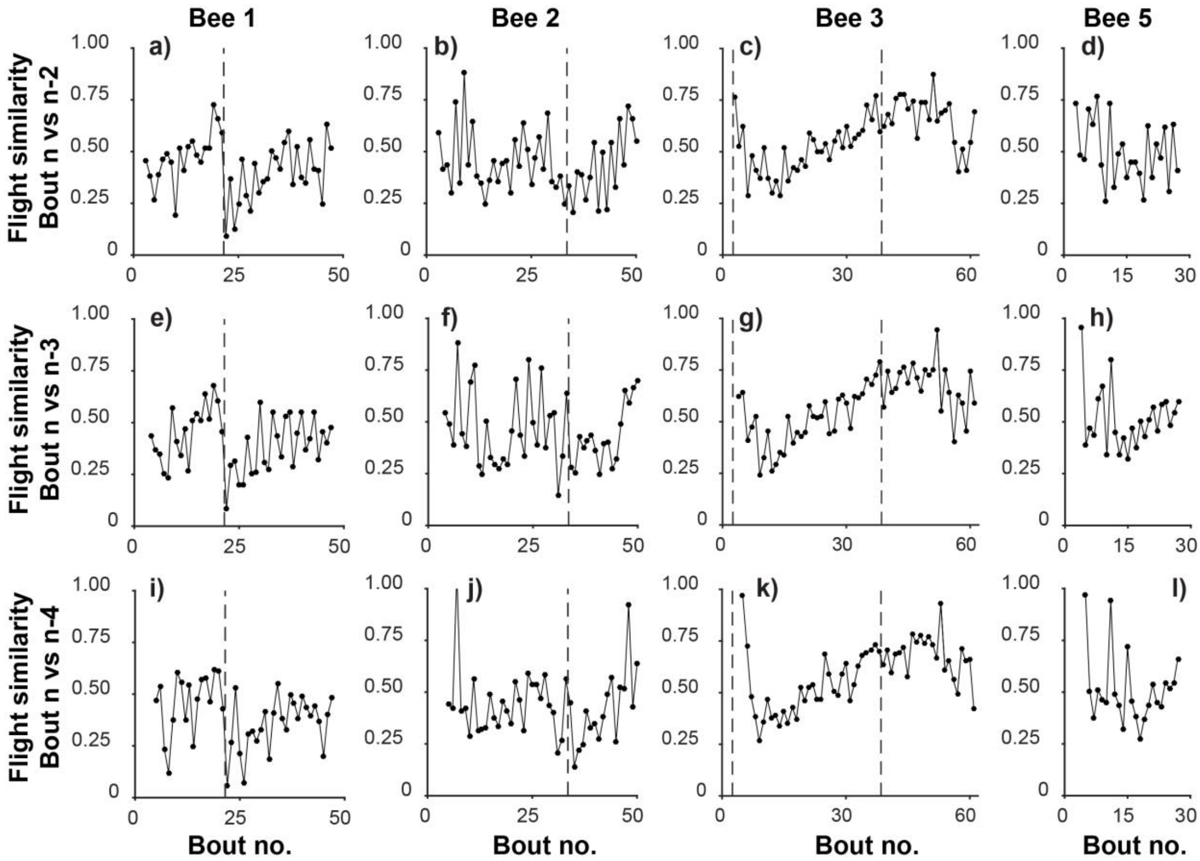

Supplementary Fig S210: Repeatability of individual legs of a foraging route for two bees. Bees 4 and 6 completed only 6 and 9 bouts respectively, too few to assess how the stability of their flights changed over time as in Fig 6; the data are shown here for comparison. Each panel represents every individual leg (transitions from any feeder to another) of every foraging bout flown by one bee. The x-axis of each plot shows the cumulative number of foraging bouts experienced by each bee. The y-axis shows the cumulative number of legs within each bout. The vertical dashed lines indicate overnight breaks between foraging bouts. a-b) For bees 4 and 6 respectively, the colour of each dot represents the identity of the destination of each leg of the route (i.e. the feeders 1-5 or the nest). The presence of horizontal rows of the same colour demonstrates that the bee in question visited the same destination at the same stage during several consecutive foraging bouts. c-d) For bees 4 and 6, the colour of each dot represents the self-similarity score comparing the probability map of the bee's location during each leg of the bout to that of the same leg in the previous bout. i-l) For bees 4 and 6, the colour of each dot represents the similarity of each legs within each bout to the most recent occasion on which the same bee made the same feeder-to-feeder transition.

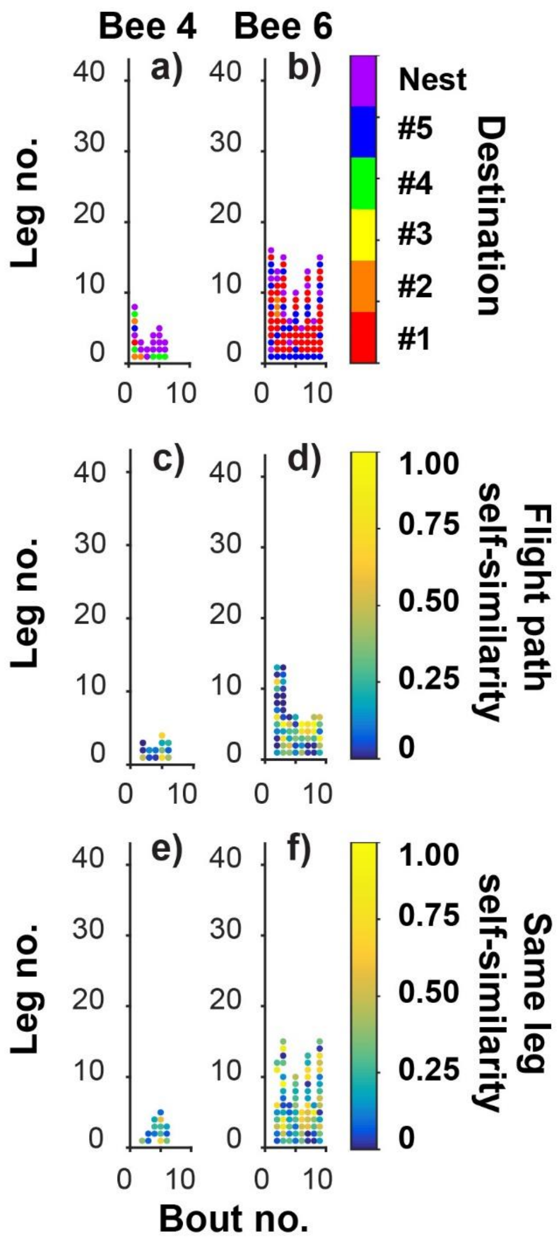
Bee 4
Bee 6
a)
b)
c)
d)
e)
f)
Leg no.
Bout no.
Nest
#5
#4
#3
#2
#1
Destination
Flight path self-similarity
Same leg self-similarity
1.00
0.75
0.50
0.25
0

Supplementary Fig S211: Probability of segments of flight of different lengths occurring within a foraging bout. The feeder visitation sequence for each bout was broken into shorter segments of two, three or four destinations and the probability was calculated of each unique sequence occurring in any given bout of a particular bee. Each panel combines the occurrences of each segment across all bouts flown by one bee. a-d) For bees 1-3 and 5 respectively, the descending rank commonness of each flight segment is plotted against the occurrence probability of that segment on a log-linear scale. Segments of length two are shown in red, those of length three in green, and those of length four in blue. The lines show how well the distributions fit an exponential distribution. The most commonly occurring segments fit the line poorly and occurred more commonly than predicted by an exponential distribution, implying the use of memory and decision making. The less common segments fit an exponential distribution well, implying that their presence within a route was generated by purely stochastic processes.

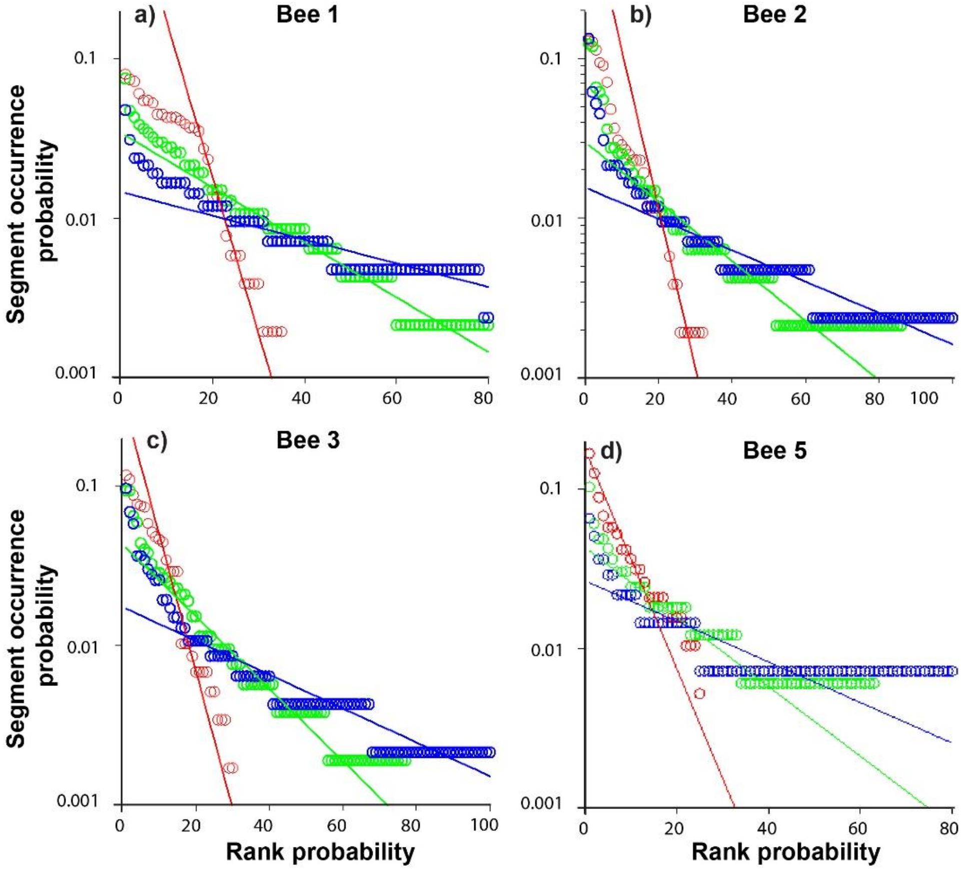

Legends for further supplementary material

Supplementary Videos S1-S6: Animated heat maps showing the development of foraging routes on the experimental feeder array. Each video graphically represents the flight path of a single bee over all of its foraging bouts on the experimental feeder array, using the same principles as Fig 2. The colour of each pixel represents the probability that the bee passed over that point in the landscape as estimated from the radar tracks using a technique derived from a Brownian Bridge movement model (described in the Methods). Each pixel represents an area 5 x 5m. Scale bars represent a distance of 20m. The colour of each pixel decays with time unless reinforced, in a manner analogous to an ant pheromone trail. Thus, abandoned segments of route gradually fade while those segments of the route used most frequently become reinforced. Videos play at 600x real speed and periods during which the bee's location did not change for more than 60s have been curtailed. Nest position: open circle; positions of feeders: closed circles.

Supplementary Dataset S1: Variables describing flight path of each bout flown by each bee. All data used in the analyses presented in the manuscript.
